# A ganglioside-based immune checkpoint enables senescent cells to evade immunosurveillance during aging

**DOI:** 10.1038/s43587-024-00776-z

**Published:** 2024-12-27

**Authors:** Charlène Iltis, Iryna Moskalevska, Antoine Debiesse, Laetitia Seguin, Christina Fissoun, Ludovic Cervera, Lyvia Moudombi, Maude Ardin, Anthony Ferrari, Coline Eliott, Didier Pisani, Alexandre Ottaviani, Manon Bourinet, Carmelo Luci, Philippe Gual, Gabriela Makulyte, David Bernard, Manon Durandy, Lou C. Duret, Tynhinane Hamidouche, Sarah Kunz, Olivier Croce, Clément Delannoy, Yann Guérardel, Fabrice Allain, Paul Hofman, Delphine Benarroch-Popivker, Laurence Bianchini, Berengère Dadone-Montaudie, Estelle Cosson, Julien Guglielmi, Thierry Pourcher, Véronique M. Braud, Marina Shkreli, Yves-Marie Pers, Christian Jorgensen, Jean-Marc Brondello, Chloé C. Féral, Marie-Cécile Michallet, Eric Gilson, Julien Cherfils-Vicini

**Affiliations:** 1 Université Côte d’Azur, Centre National de la Recherche Scientifique (CNRS) UMR7284, Institut National de la Santé et de la Recherche Médicale (INSERM) U1081, Institute for Research on Cancer and Aging, Nice (IRCAN), Nice, France; 2 Institut Hospitalo-Universitaire (IHU) RESPIRera and FHU OncoAge, CHU Nice, Nice, France; 3 Centre de Recherche en Cancérologie de Lyon (CRCL), Centre Léon Bérard, Université de Lyon, Université Claude Bernard Lyon 1, INSERM 1052, CNRS 5286, Équipe Labelisée la Ligue Contre le Cancer, Lyon, France; 4 Institute of Regenerative Medicine and Biotherapies (IRMB), INSERM U1183, University of Montpellier, Montpellier, France; 5 Université Côte d’Azur, Centre National de la Recherche Scientifique (CNRS) UMR7370, Laboratoire de PhysioMédecine Moléculaire (LP2M), Nice, France; 6 Université Côte d’Azur, Institut National de la Santé et de la Recherche Médicale (INSERM) U1065, Centre Méditerranéen de Médecine Moléculaire (C3M), Nice, France; 7 Université de Lille, Centre National de la Recherche Scientifique (CNRS) UMR 8576 - UGSF - Unité de Glycobiologie Structurale et Fonctionnelle, Lille, France; 8 Institute for Glyco-core Research (iGCORE), Gifu University, Gifu, Japan; 9 Laboratory of Clinical and Experimental Pathology and Biobank, CHU Nice, Pasteur Hospital, Nice, France; 10 Université Côte d’Azur, Centre National de la Recherche Scientifique (CNRS) UMR7275, Institut national de la santé et de la recherche U1323, Institut de Pharmacologie Moléculaire et Cellulaire, Valbonne, France; 11 Laboratory Transporter in Imaging and Radiotherapy in Oncology (TIRO), Direction de la Recherche Fondamentale (DRF), Institut des Sciences du Vivant Fréderic Joliot, Commissariat à l’Energie Atomique et aux Énergies Alternatives (CEA), University Côte d’Azur, Nice, France; 12 Clinical Immunology and Osteoarticular Diseases Therapeutic Unit, Montpellier University Hospital, Montpellier, France; 13 Department of Medical Genetics, CHU, Nice, France

**Keywords:** Senescence, Ageing, Innate immune cells

## Abstract

Although senescent cells can be eliminated by the immune system, they tend to accumulate with age in various tissues. Here we show that senescent cells can evade immune clearance by natural killer (NK) cells by upregulating the expression of the disialylated ganglioside GD3 at their surface. The increased level of GD3 expression on senescent cells that naturally occurs upon aging in liver, lung, kidney or bones leads to a strong suppression of NK-cell-mediated immunosurveillance. In mice, we found that targeting GD3^+^ senescent cells with anti-GD3 immunotherapy attenuated the development of experimentally induced or age-related lung and liver fibrosis and age-related bone remodeling. These results demonstrate that GD3 upregulation confers immune privilege to senescent cells. We propose that GD3 acts as a senescence immune checkpoint (SIC) that allows senescent cells to escape immunosurveillance and to trigger immune anergy during aging.

## Abstract (web summary)

This study identifies a novel immune checkpoint in senescent cells that is linked to the ganglioside GD3 and that contributes to the evasion of immune clearance by these cells and to aging and age-related diseases.

## Main

Advancing age goes hand in hand with the increased susceptibility to develop diseases that lead to functional decline, loss of autonomy and healthcare system saturation. These include a wide range of cancers, chronic diseases and immunosenescence, resulting in increased vulnerability to infection, as evidenced by the severe acute respiratory syndrome coronavirus 2 (SARS-CoV-2) pandemic, which is severely and disproportionately affecting the older adult populations. Mechanistically, the accumulation of senescent cells (SnCs) in tissues emerges as a key driver of aging and age-associated diseases^1^. Thus, according to the geroscience hypothesis, considerable efforts are being made to find senotherapeutic strategies that allow the elimination or modification of SnCs to prevent and simultaneously treat many age-related diseases^2^. Different senolytic compounds target the SnC intrinsic property to resist apoptosis due to Bcl-2 family protein overexpression (for example, ABT-737, an inhibitor of Bcl-2 and Bcl-XL^3^, and ABT-263, an inhibitor of Bcl-W and Bcl-XL^4^). More recently, the use of senescent-specific chimeric antigen receptor (CAR)-T cells in mice strongly reduced age-associated liver fibrosis and extended overall survival of lung adenocarcinoma-bearing mice treated with senescence-inducing drugs^5^. Finally, the use of senolytics in old mice reduced SARS-CoV-2 infection mortality^6^.

Despite the existence of immune pathways to eliminate them^7–11^, some SnCs can be tolerated in tissues for decades^12,13^, and how they can be tolerated by the immune system remains an open question^7–11^. The mechanism(s) by which these SnCs evade T cell surveillance can depend on immune checkpoints such as PD-L1 (refs. ^14,15^). However, how SnC cells can evade from innate immunity, such as NK cell killing, is still elusive. In the present study, we discovered that SnCs can gain an immune privilege when they express at their cell surface a high level of the ganglioside GD3, leading to the escape from natural killer (NK) cell killing. This is the case for a large panel of SnC types, which upregulate the *ST8SIA1* gene encoding the enzyme synthetizing GD3. In contrast, oncogene-induced SnCs do not trigger *ST8SIA1* expression, enabling their elimination by NK cells. Moreover, we demonstrate that anti-GD3 immunotherapy in mice prevents the development of bleomycin-induced lung fibrosis and attenuates different types of age-related disorders: lung and liver fibrosis and osteoporosis. These findings reveal GD3 as a senescence immune checkpoint (SIC) and as a promising target for anti-senescence therapy.

## Results

### Replicative SnCs recruit NK cells but locally inhibit their degranulation capacities

**Fig. 1:**
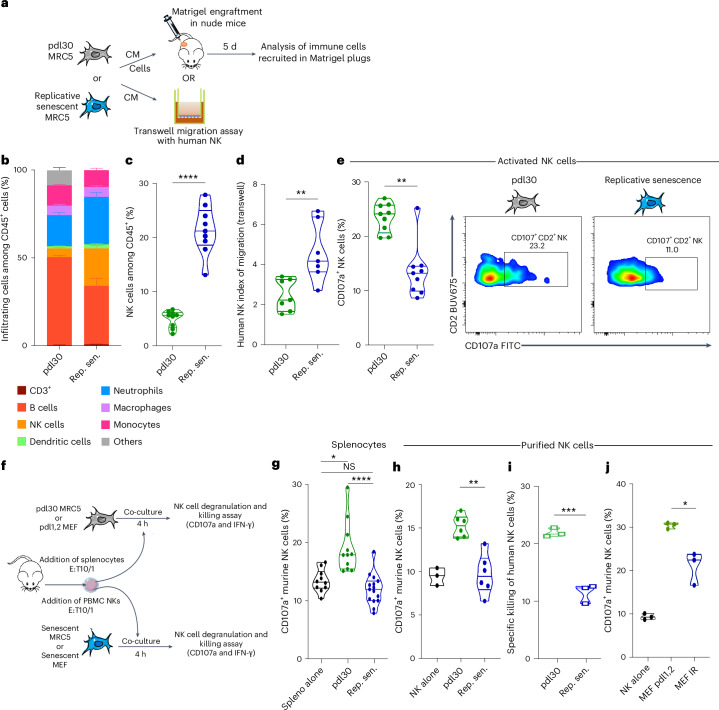
Human replicative SnCs recruit NK cells in vivo but inhibit their degranulation in an SASP-independent mechanism. **a**, Scheme of the Matrigel plug assay and the transwell migration assay. **b**, Quantification of the immune cell infiltration induced by human replicative senescent MRC5 cells or proliferative MRC5 cells after pdl30 in Matrigel plug assay. **c**, Quantification of NK cells among infiltrating CD45^+^ cells. **d**, Quantification of in vitro migration assay of primary human NK cells in presence of conditioned media from pdl30 or replicative senescent MRC5 cells during transwell assays. **e**, Quantification of the NK cell degranulation within the Matrigel plug assay. **f**, Representative scheme and the effector:target (E:T) ratio of the in vitro co-culture assay of human replicative MRC5 senescent cells or irradiated senescent MEFs with mouse splenocytes or purified human NK cells or purified mouse NK cells. **g**,**h**, Quantification of NK cell degranulation in bulk among splenocytes (**g**) or purified (**h**) during in vitro co-culture experiments. **i**, In vitro killing assay of pdl30 MRC5 or replicative senescent MRC5 by primary human purified NK cells. **j**, Quantification of primary purified mouse NK cell degranulation in co-culture with pdl1,2 or irradiated senescent MEF cells. Data are presented as mean ± s.e.m. Experiments were performed with *n* = 9 mice per group (**b**–**e**)—*P < 0.05, **P < 0.01 and ***P < 0.001; two-tailed Mann–Whitney *U*-test (**b**–**j**)—or represent the mean of *n* = 3 independent experiments—*P < 0.05, **P < 0.01 and ***P < 0.001; Student’s t-test (**g**). Rep. sen., replicative senescent. Source data

**Extended Data Fig. 1:**
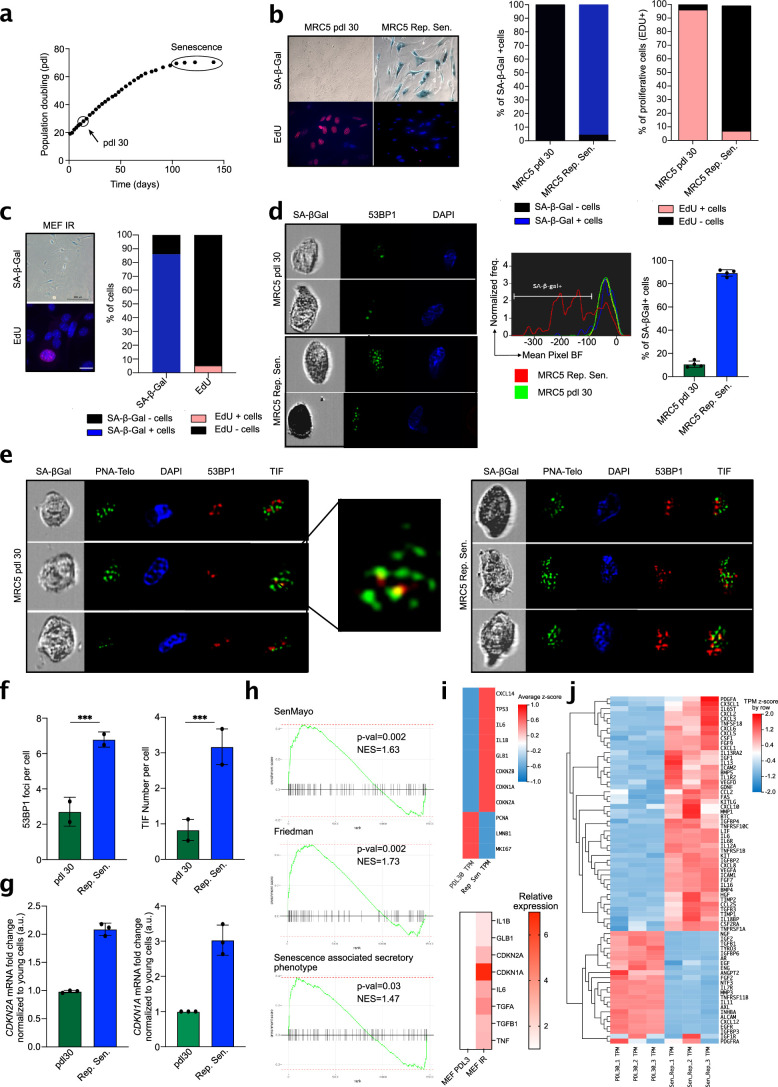
Characterization of MRC5 replicative senescent cells. **a**, Growth curve of the MRC5 cells until replicative senescence. pdl. Population doubling level. **b**, **c**, SA-b-Galactosidase and EdU incorporation assay on pdl30 or replicative senescent MRC5 (**b**) and on irradiated MEF (**c**) (Upper scale = 200μm, lower scale 20μm) (left panel) and quantification of the percentage of SA-b-Galactosidase + and EdU – cells (right panels). **d-f**, Analysis of the SA-b-Galactosidase assay, DNA damage level (53BP1 staining) (**d, e**) or DNA damage level (53BP1 staining) specifically at telomeres (telomere staining (PNA-Telo) and colocalization (TIF for Telomere Induced Foci)) (**e, f**) on pdl30 or replicative senescent MRC5 using ImagestreamX and quantification of the number of total 53BP1 foci or 53BP1 foci at telomere (TIF). Due to the SA-b-Galactosidase staining, cells are darker, and the Mean pixel intensity is decreasing (**d**). **g**, qPCR analysis of *CDKN2A* (left panel) and *CDKN1A* (right panel) expression in replicative senescence in MRC5 cells. **h**, Senescence-related gene set enrichement analysis (GSEA) on SenMayo, Friedman and Senescence Associated Secretory Phenotype gene sets of replicative senescent MRC5. **i**, Heatmap of senescent markers of replicative senescent MRC5 compared to pdl30 cells sequenced in bulk RNAseq (upper panel) of irradiated MEF compared to pdl3 cells quantified by qPCR (lower panel). **j**, Heatmap of soluble factors expressed by replicative senescent MRC5 compared to pdl30 cells sequenced in bulk RNAseq. Data are representative of 8 different batches of replicative senescent cells corresponding to the batches used in Figs. 1 and 2. Data are represented as mean ± s.e.m. Experiment done on n = 4 independent experiments (**d**); Experiment done on n = 2 independent experiments (**e**, **f**); Experiment done on n = 3 senescent batches (**g-j**);*p < 0.05, **p < 0.01, and ***p < 0.001; two-tailed Mann–Whitney U test (**d,f**). Source data

**Extended Data Fig. 2:**
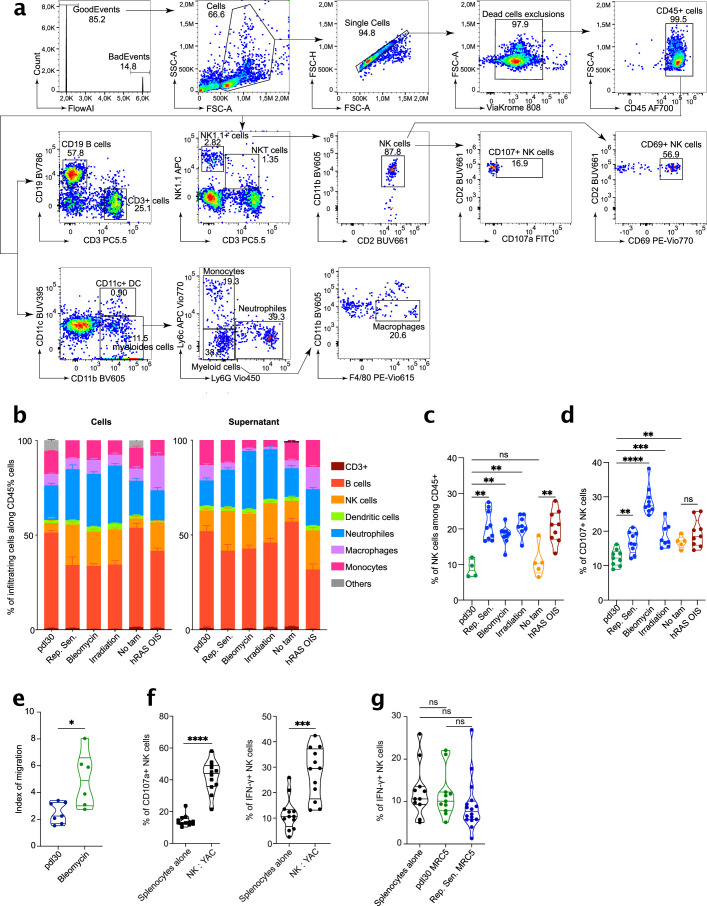
Senescent cells but not their SASP affect immune infiltration and NK cell activation. **a**, Gating strategy for the immune phenotyping of infiltrating cells from the Matrigel plug assay (Fig. 1a-b). **b**, Quantification of the immune cell infiltration induced by proliferative, oncogene or different stress-induced senescent MRC5 cells (left panel) or supernatant (right panel) in matrigel plugs. **c-d**, Quantification of the NK cell infiltration (**c**) and degranulation (**d**) induced by the supernatant from proliferative, replicative senescent, oncogene or stress induced MRC5 cells in matrigel plugs. **e**, Quantification of in vitro migration of primary human NK cells in presence of supernatant from pdl30 or bleomycin induced senescent MRC5 during transwell assays. **f**, Positive (YAC-1 cells, a strong NK cell target) and negative controls of degranulation (CD107a+ NK cells) or IFN-g production in in vitro co-culture experiments. **g**, Analysis of IFN- g production in in vitro co-culture experiments with senescent cells. All experiments are performed with n = 9 mice per group (**a-d**); *p < 0.05, **p < 0.01, and ***p < 0.001; Data are represented as mean ± s.e.m.; two-tailed Mann–Whitney U test or data represent the mean of n = 3 independent experiments; *p < 0.05, **p < 0.01, and ***p < 0.001; two-tailed Mann–Whitney U test (**e, f**). Source data

To understand how SnCs can evade the immune system, we studied the immune response to human replicative SnCs using an in vivo Matrigel plug assay in nude mice (Fig. 1a–c,e). Specifically, human lung primary fibroblasts (MRC5) were cultured until replicative senescence, defined as at least 90% senescence-associated β-galactosidase-positive (SA-β-Gal^+^) and 10% 5-ethynyl-2′-deoxyuridine-negative (EdU−) cell population (Extended Data Fig. 1a,b); increased DNA damage response, including at telomeres (Extended Data Fig. 1d–f); increased expression of *CDKN2A* and *CDKN1A* (Extended Data Fig. 1g); an enrichment of multiple senescence signatures (Extended Data Fig. 1h,i and Supplementary Table 1; GSE262856); and an increased expression at the transcriptomic level (quantitative polymerase chain reaction (qPCR) and RNA sequencing (RNA-seq) analysis) of soluble and pro-inflammatory molecules (Extended Data Fig. 1i–k). Consistent with previous reports^10,16–18^, the innate immune recruitment induced by human replicative SnCs was extensive, with an increase of NK cells (CD3^−^NK1.1^+^CD2^+^) and neutrophils (CD11b^+^Ly6G^+^Ly6C^−^) infiltration (Fig. 1b,c and Extended Data Fig. 2a,b). This recruitment is dependent on the senescence-associated secretory profile (SASP) because an in vivo Matrigel assay or an in vitro transwell migration assay performed with the conditioned media of the same SnCs increased both mouse (Extended Data Fig. 2c) and human (Fig. 1d) NK cell recruitment, respectively. It is noteworthy that, although the SASP in the Matrigel plug slightly increased NK cell degranulation (Extended Data Fig. 2d), the in vivo degranulation of NK cells recruited by human SnCs was reduced two-fold compared to NK cells recruited by proliferative cells (Fig. 1e). Consistently, in an in vitro co-culture experiment (Fig. 1f–j), human SnCs strongly inhibited mouse NK cell degranulation as compared to proliferative young counterparts (Fig. 1f,g) but not the interferon-gamma (IFN-γ) production (Extended Data Fig. 2f,g). Replicative human SnCs also inhibited human NK cell degranulation (Fig. 1h) and human NK-cell-specific killing (Fig. 1i). Similarly, mouse NK cell degranulation inhibition was observed with purified mouse NK cells co-cultivated with mouse SnCs (irradiated mouse embryonic fibroblasts (MEFs); Fig. 1j). Thus, although the SASP of human SnCs enhanced mouse and human NK cell recruitment in vitro and in vivo, human replicative and mouse ionizing radiation (IR)-induced SnCs inhibited mouse and human NK cell functions (degranulation and killing) through an SASP-independent mechanism.

### Immunosuppressive capacities of SnCs rely on GD3 expression at their surface

**Fig. 2:**
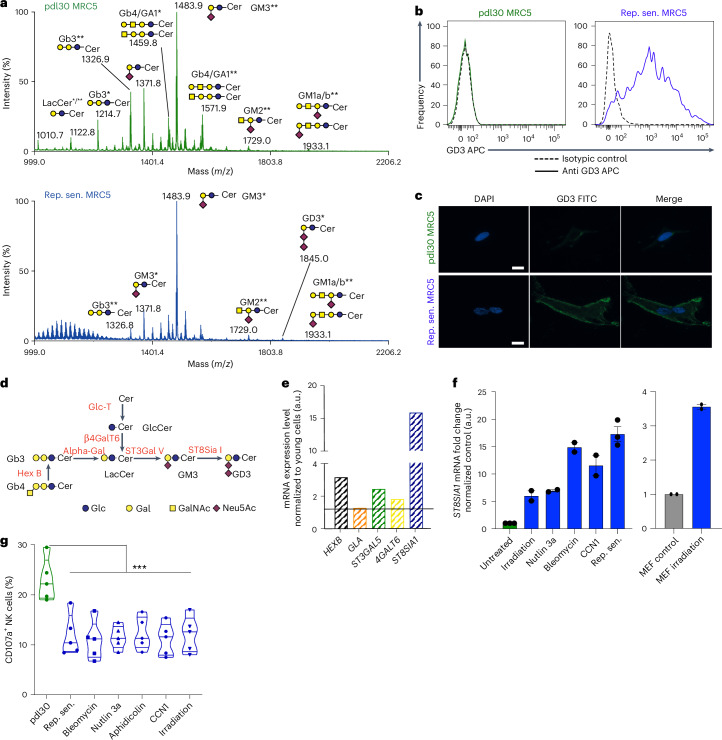
The ganglioside GD3 is strongly expressed by SnCs and inversely correlates with their immunogenic properties toward NK cells. **a**, Mass spectrometry analysis of permethylated GSLs from human young (pdl30) or replicative senescent MRC5 cells; GSLs are present as d18:1/C16:0 (*) and d18:1/C24:0 (**) isomers. **b**,**c**, Analysis of GD3 expression in human young or replicative senescent MRC5 cells, either by FACS (**b**) or by IF (**c**) (scale, 20 μm). **d**, Representative scheme of ganglioside biosynthesis pathway. **e**, qPCR analysis of ganglioside biosynthesis enzymes in replicative SnCs. **f**, qPCR analysis of *ST8SIA1* expression in replicative and stress-induced senescence in MRC5 cells and MEFs. **g**, Quantification of mouse NK cell degranulation in in vitro co-culture experiment with proliferative, replicative senescent or stress-induced MRC5 cells. Data represent the mean ± s.e.m. of *n* = 2 independent experiments (**f**), *n* = 3 independent experiments (**a**–**e**) and *n* = 5 independent experiments (**g**). *P < 0.05, **P < 0.01 and ***P < 0.001; two-tailed Mann–Whitney *U*-test. Source data

**Extended Data Fig. 3:**
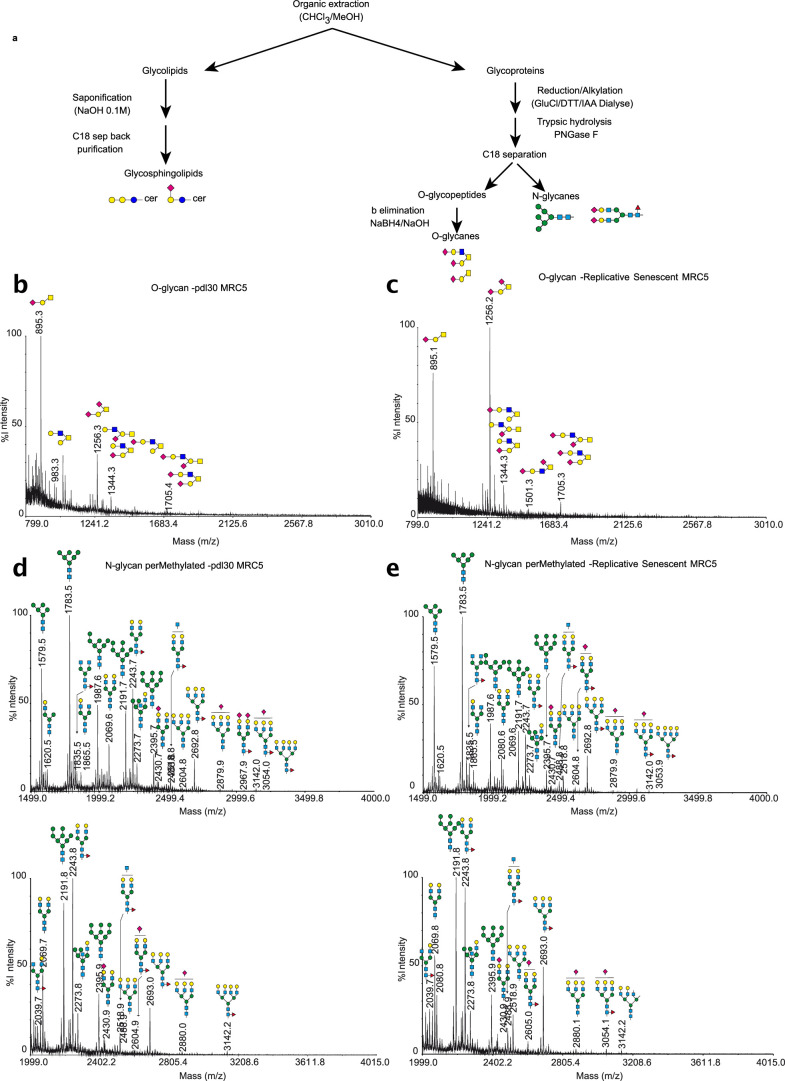
Mass spectrometry analysis of human replicative senescent cells for O-glycans and N-Glycans. **a**, scheme representing the analysis strategy. **b, c**, Representative data of the O-glycan composition of pdl30 (young) MRC5 (**b**) or replicative senescent MRC5 (**c**). **c, d**, **d**, **e**, Representative data of the N-glycan composition of pdl30 (young) MRC5 (**c**)(**d**) or replicative senescent MRC5 (**d**)(**e**).

**Extended Data Fig. 4:**
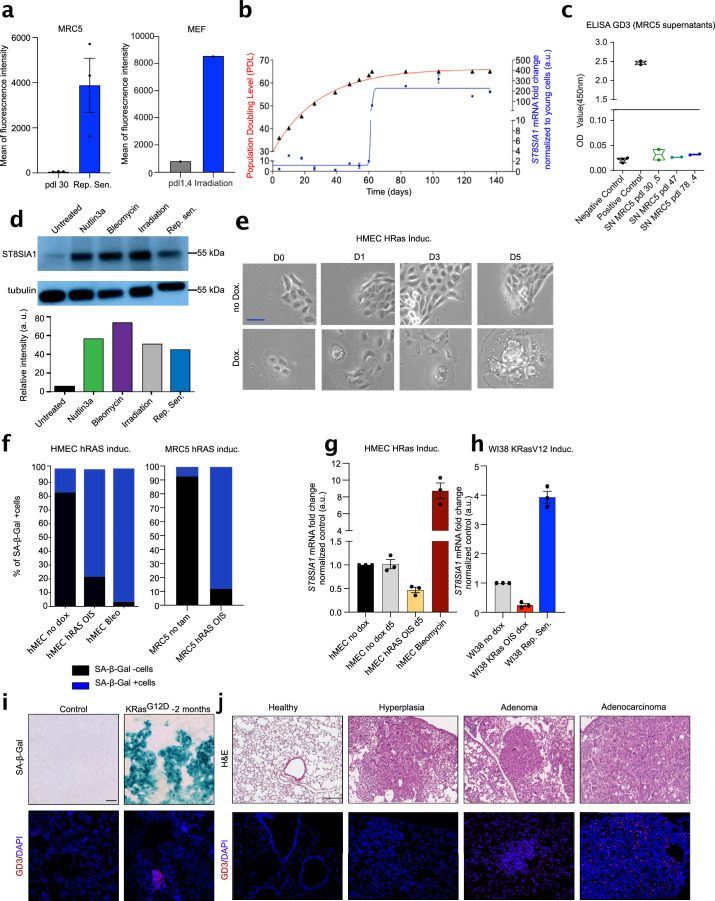
GD3 producing ST8SIA1 enzyme in expressed by Replicative and Stress induced senescent cells but not by Oncogene-induced senescent cells. **a**, Quantification of GD3 expression analysis by flow cytometry of MRC5 cells (corresponding to Fig. 2b) or MEF cells. **b**, Growth curve of MRC5 and kinetic of *ST8SIA1* expression by qPCR during replicative senescence. **c**, GD3 dosage by ELISA in supernatants of proliferating or replicative senescent cells (2 independent experiments). **d**, Western blot quantification of ST8SIA1 in different types of senescence. **e**, Morphology analysis of HMEC cells overexpressing Hras in an inducible manner (representative of n > 5 experiments) (Scale =100μm). **f**, SA-b-Galactosidase assay, and quantification for HMEC and MRC5 cells overexpressing Hras in an inducible manner. **g**, **h**, qPCR assessment of *ST8SIA1* expression in HMEC overexpressing Hras senescent cells or Bleomycin induced HMEC senescent cells (**g**) or WI38 cells in replicative senescent or RasV12 induced senescence (**h**). **i**, SA-b-Galactosidase assay and GD3 immunofluorescence staining on paraffin embedded lung section from control or 2 months-induced KrasG12D overexpression mice. **j**, Analysis of GD3 expression in lungs of KrasG12D overexpression mice overtime. **i, j** are representative of n = 2 mice per condition (Scale =100μm). Data are represented as mean ± s.e.m. The experiments were performed on n = 3 (**a** left, **f, g, h**). The experiment was performed on n = 1 (**a** right, **d**). Source data

Because SASP was insufficient to mimic the immunosuppressive effects of SnCs on NK cell degranulation (Extended Data Fig. 2d), we hypothesized that cell surface molecules, essentially represented by glycoproteins and glycolipids, are involved. We analyzed the glycocalyx composition of proliferative and senescent MRC5 cells using mass spectrometry, focusing on glycosphingolipids (Fig. 2a), *O*-glycans (Extended Data Fig. 3a–c) and *N*-glycans (Extended Data Fig. 3d,e). Mass spectrometry analysis showed that the proliferative MRC5 *N*-glycome was made of oligomannosylated (Man_5_GlcNAc_2_ to Man_9_GlcNAc_2_), neutral and complex sialylated LacNAc containing *N*-glycans, whereas the *O*-glycome was exclusively constituted of monosialylated and disialylated short *O*-glycans (Extended Data Fig. 3b,d). The *N*-glycome and *O*-glycome were not significantly modified in replicative MRC5 SnCs compared to proliferative young MRC5 cells (Extended Data Fig. 3c,e). In contrast, the glycosphingolipids pattern of replicative SnCs was modified compared to proliferative control cells. Although young cells contained both neutral globosides (Gb3 and Gb4/GA1) and gangliosides (GM3, GM2 and GM1 sialylated), replicative SnCs contained primarily gangliosides with a marked upregulation of disialylated ganglioside (GD3) (Fig. 2a). Flow cytometry and immunofluorescence (IF) analysis (Fig. 2b,c and Extended Data Fig. 4a) confirmed the induction of GD3 expression in replicative SnCs. Based on the expression of the ganglioside biosynthesis pathway genes (Fig. 2d), we found that only the gene encoding ST8SIA1, the enzyme that synthetizes GD3, was sharply increased in replicative SnCs (Fig. 2e). The expression of *ST8SIA1* did not increase progressively during the cell proliferative history but, instead, was induced and massively upregulated at the onset of senescence (Extended Data Fig. 4b). The fact that GD3 was not detected in the supernatant of proliferative MRC5 cells or replicative SnCs rules out the hypothesis that GD3 is a SASP component (Extended Data Fig. 4c). Such an upregulation of *ST8SIA1* expression, together with an elevated level of GD3, was observed in prematurely induced human SnCs triggered by a wide range of stressors (Fig. 2f and Extended Data Fig. 4d) as well as in irradiated MEFs (Fig. 2f and Extended Data Fig. 4a).

### Oncogene-induced SnC capacity to activate NK cells depends on their repression of GD3 expression through the RIP140/PGC-1α/ERRα pathway

**Fig. 3:**
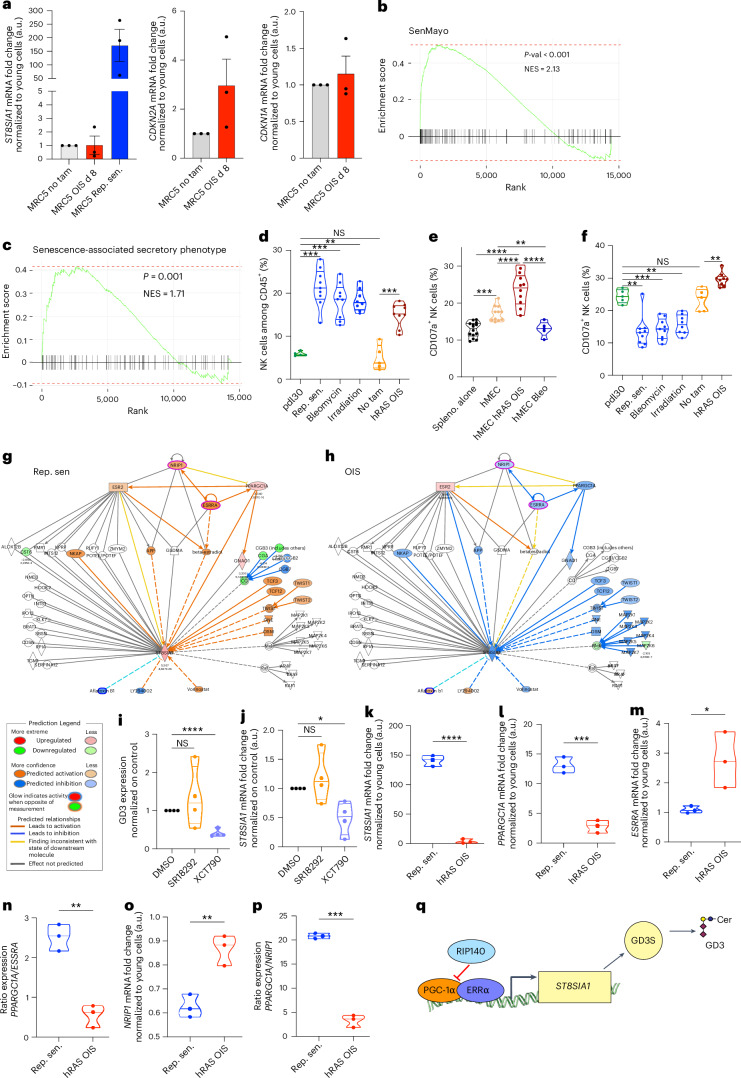
ERRα/PGC-1α-dependent GD3 expression is absent in OIS allowing their activation of NK cells. **a**, qPCR assessment of *ST8SIA1*, *CDKN2A* and *CDKN1A* expression in MRC5 cells in tamoxifen (tam)-inducible hRAS senescence. **b**,**c**, Senescence-related GSEA on SenMayo (**b**) and SASP (**c**) gene sets of hRAS-induced senescent MRC5 cells. **d**, Quantification of the NK cell infiltration induced by proliferative, replicative senescent, oncogene-induced or stress-induced MRC5 cells in Matrigel plug assay. **e**, Quantification of mouse NK cell degranulation in in vitro co-culture experiment with oncogene-induced or bleomycin-induced senescence in hMECs. **f**,**e**, Quantification of the in vivo NK cell degranulation induced by young, replicative, oncogene-induced or stress-induced senescent MRC5 cells in Matrigel plug assay. **g**,**h**, IPA of ST8SIA1 upstream pathways. Gene networks revealed by DEG in replicative senescence (**g**) and hRAS oncogene-induced senescence (**h**) in MRC5 cells compared to their control by RNA-seq (Extended Data Fig. 1) are overlayed in colors on each network. **i**,**j**, Flow cytometry assessment of GD3 expression (**i**) and qPCR assessment (**j**) of ST8SIA1 expression by replicative senescent MRC5 cells after 72 h of treatment with PGC1-α inhibitor (SR18292) and ERRα inhibitor (XCT790) normalized on DMSO-treated cells. **k**–**n**, qPCR analysis of *ST8SIA1* (**k**), *PPARGC1A* (**l**) and *ESRRA* (**m**) normalized on proliferative MRC5 cells and ratio between the expressions of *PPARGC1A* and *ESRRA* (**n**). **o**,**p**, qPCR analysis of *NRIP1* normalized on proliferative MRC5 cells (**o**) and ratio between the expressions of *PPARGC1A* and *NRIP1* (**p**). **q**, Representative scheme of the regulation of *ST8SIA1* expression by the nuclear receptor ERRα, its co-activator PGC1-α and its co-repressor RIP140. Data are represented as mean ± s.e.m. (**a**). Experiments in **a** and **k**–**p** were performed on *n* = 3 batches of SnCs. Experiments in **i** and **j** were performed on *n* = 4 independent experiments. Experiments in **d** and **f** are representative of *n* = 9 mice. Experiment in **e** was performed on *n* = 10 independent experiments. *P < 0.05, **P < 0.01 and ***P < 0.001; two-tailed Mann–Whitney *U*-test. Source data

In contrast, when senescence in MRC5 cells was induced by oncogene activation (oncogene-induced senescence (OIS); Extended Data 4e,f), *ST8SIA1* expression was downregulated compared to replicative senescent cells (Fig. 3a–c). Similar results were obtained with OIS WI38 fibroblasts or human mammary epithelial cells (hMECs) (Extended Data Fig. 4e,g,h). Accordingly, in the lungs of 2-month-old KRasG12D-expressing mice, OIS cells, defined as SA-β-Gal^+^ cells, did not express GD3 (Extended Data Fig. 4i,j). The NK cell recruitment capacity was not modified by the expression of GD3 (Fig. 3d and Extended Data Fig. 2b–d), demonstrating an uncoupling between SASP chemoattractant functions and GD3 immunosuppressive roles. Although consistently, with their lack of GD3 expression, OIS cells increased NK cell degranulation in vitro (Fig. 3e) or in vivo Matrigel assay (Fig. 3f), all other types of SnCs inducing GD3 abolished NK cell degranulation in vivo (Fig. 3f) or in vitro (Fig. 2g).

To investigate the mechanisms governing the differential expression of ST8SIA1/GD3 between replicative SnCs and OIS cells, we analyzed potential regulatory networks of *ST8SIA1* by Ingenuity Pathway Analysis (IPA). This analysis suggested a regulation of *ST8SIA1* expression by signaling pathways, including NFκB, MAPK or PGC-1α pathways (Fig. 3g,h). To improve the accuracy of candidate pathways regulating *ST8SIA1* at senescence, we then enriched the pathway analysis (Enriched Network Analysis) with RNA-seq data of proliferative or replicative senescent MRC5 cells (Fig. 3b, Extended Data Fig. 1h and Supplementary Table 1). The enriched network pinpointed the PGC1α/ERRα pathway as a key regulator of *ST8SIA1* gene expression (Fig. 3g,h), a pathway known for its role in senescence-associated metabolic changes and mitochondrial dysfunction^1,19^. Treatments with inhibitors targeting PGC1-α (SR18292) and ERRα (XCT790) in replicative SnCs showed the crucial role of ERRα in regulating *ST8SIA1* and GD3 expression (Fig. 3i,j). Notably, as compared to replicative SnCs, OIS cells expressed a lower level of the *PPARGC1A* (PGC1-α) and *ST8SIA1* genes as well as a higher level of the *ESRRA* (ERRα) and *NRIP1* (RIP140) genes (Fig. 3k–m,o). Overall, there is an inverse *PPARGC1A/ESRRA* and *PPARGC1A/NRIP1* ratio between replicative SnCs and OIS cells (Fig. 3n,p). This suggests that the OIS cells repress *ST8SIA1* gene expression via RIP140, whereas replicative SnCs activate the expression of the *ST8SIA1* gene through an increase in PPARGC1A (Fig. 3q).

### NK cell immunosuppression by SnCs depends on GD3 expression

**Fig. 4:**
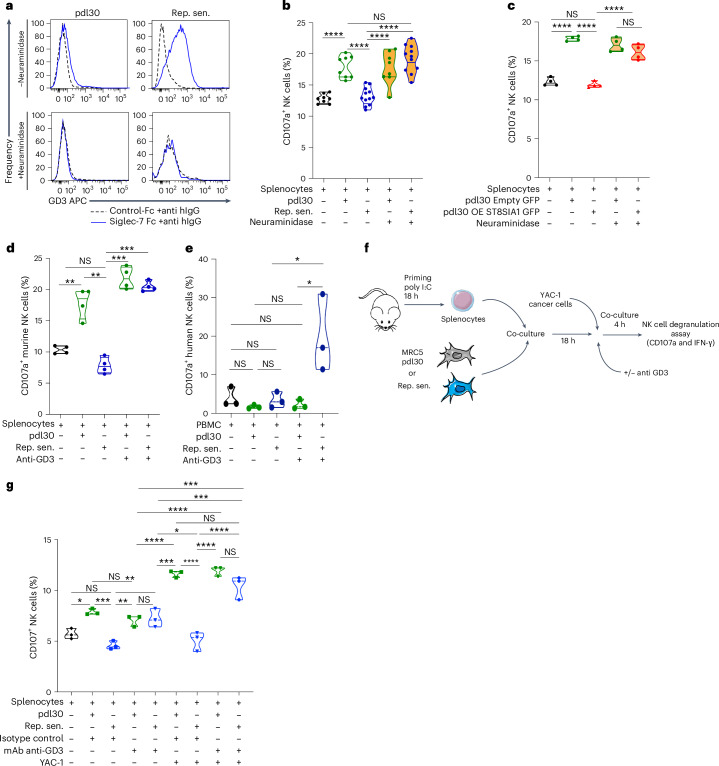
GD3 expression by SnCs directly determines their NK-cell-mediated immune surveillance. **a**, Flow cytometry analysis of the binding of soluble recombinant Siglec-7-Fc proteins by pdl30 or replicative senescent MRC5 cells with or without neuraminidase treatment. **b**, Degranulation (CD107a^+^) of NK cells in in vitro co-culture experiments with pdl30 or replicative senescent MRC5 cells treated or not with neuraminidase. **c**, Degranulation of NK cells in in vitro co-culture with young cells overexpressing ST8SIA1 and treated or not with neuraminidase. **d**,**e**, Quantification of mouse (**d**) or human (**e**) NK cell degranulation in co-culture experiment with young or senescent MRC5 cells with an anti-GD3 antibody. **f**, Representative scheme of the in vitro cancer cell challenge assay. **g**, Determination of NK cell functionality in vitro after 18 h of co-culture with senescent cells and 4-h YAC-1 cell rechallenge. Data represent the mean of *n* = 4 independent experiments (**b–d**). Experiment in **e** was performed on *n* = 3 independent experiments. Experiment in **g** was performed with *n* = 8 mice per group. *P < 0.05, **P < 0.01 and ***P < 0.001; two-tailed Mann–Whitney *U*-test. Source data

**Extended Data Fig. 5:**
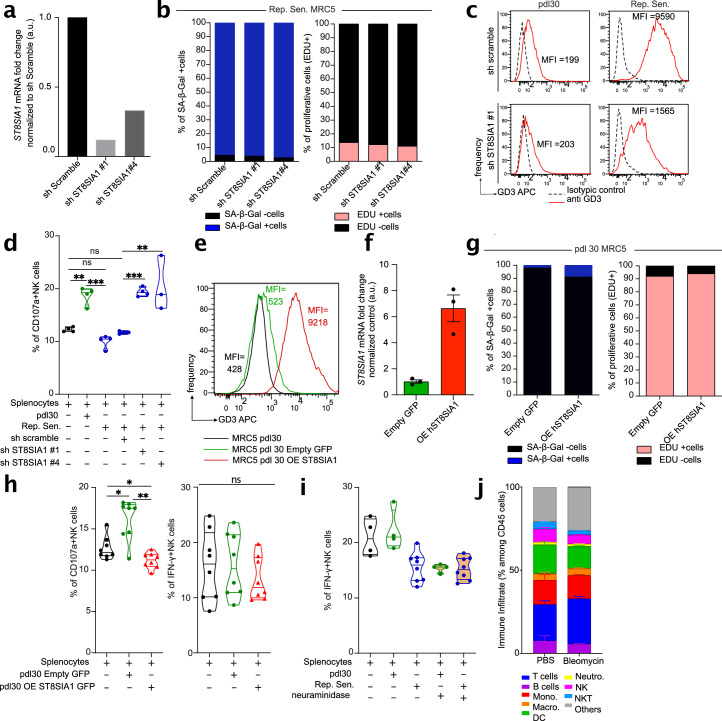
The expression of a sialylated GD3 is strictly required for inhibition of NK cell degranulation by senescent cells. **a**, analysis by qPCR of *ST8SIA1* expression in replicative senescent after lentiviral infection for shRNA targeting *ST8SIA1*. **b**, Quantification of the percentage of SA-b-Galactosidase positive and EdU negative cells after *ST8SIA1* knock-down. **c**, Flow cytometry analysis of GD3 expression in replicative senescent after lentiviral infection for shRNA targeting *ST8SIA1*. **d**, in vitro degranulation of NK cells after a co-culture experiment using replicative senescent cells knocked down for *ST8SIA1*. **f, g**, Cytometry analysis of GD3 expression (**e**) and qPCR expression of *ST8SIA1* (**f**) by pdl30 MRC5 overexpressing or not *ST8SIA1*. **g**, Quantification of the percentage of SA-b-Galactosidase positive and EdU negative cells after ST8SIA1 overexpression. **h**, degranulation (CD107a + ; left panel) or IFN-g production (right panel) by NK cells in in vitro co-culture experiments using pdl30 MRC5 overexpressing ST8SIA1. **i**, IFN-g production by NK cells in in vitro co-culture experiments with pdl30 or replicative senescent MRC5 cells treated or not with neuraminidase. **j**, Analysis of the immune infiltration by flow cytometry of control or fibrotic lungs used in Fig. 7a-d. Experiment done on n = 1 (**a**). Data represent the mean of n = 4 independent experiments (**b-d**); Data represent the mean ± s.e.m of n = 3 independent experiments (**f,g**); Experiment done on n > 4 (**i**). Data represented as mean ± s.e.m of n = 5 mice (**j**) *p < 0.05, **p < 0.01, and ***p < 0.001; two-tailed Mann–Whitney *U* test. Source data

**Extended Data Fig. 6:**
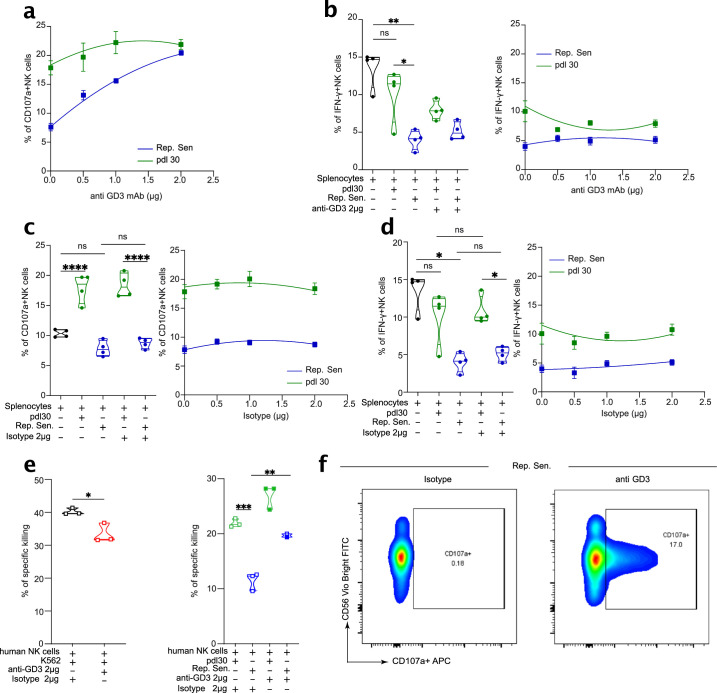
mAb against GD3 restore efficient NK cell degranulation and killing of senescent cells. **a**, Dose response effect of GD3 mAb on NK cell degranulation against replicative senescent or pdl30 MRC5 (data corresponding to Fig. 3e). **b**, IFN-g production in *in vitro* co-culture experiments using anti-GD3 monoclonal antibody (corresponding to Fig. 3e). **c, d**, degranulation (**c**, CD107a+ NK cells) or (**d**) IFN- g production in *in vitro* co-culture experiments using isotypic mouse IgG3 antibody as control as same concentration than anti-GD3 mAb. **e**, *in vitro* killing assay of pdl30 MRC5 or replicative senescent MRC5 by human NK cells in presence or not of anti-GD3 mAb; performed in same time than experiment in Fig. 1f, same controls. **f**, Gating for the quantification of human NK cell degranulation in co-culture asssays performed in Fig. 2j. Data represent the mean of n > 3 independent experiments; *p < 0.05, **p < 0.01, and ***p < 0.001; two-tailed Mann–Whitney *U* test. Source data

As GD3 is known to trigger the inhibitory immunoreceptor Siglec-7 (or Siglec-E/H in mice)^20,21^, we tested the hypothesis that the elevated production of this ganglioside in SnCs mediates NK cell inhibition. Binding of soluble recombinant Siglec-7 receptor was strongly increased in senescent MRC5 cells in contrast to proliferative MRC5 cells where it was almost absent (Fig. 4a). This binding was totally abolished by enzymatic treatment (neuraminidase, which hydrolyzes terminal N-acyl or O-acyl neuraminic acids; Fig. 4a), revealing that the sialic acids can engage the inhibitory receptor Siglec-7. Consistently, OIS cells that do not express GD3 increased NK cell degranulation in vitro (Fig. 2d) or in vivo Matrigel assay (Fig. 2e), all other types of SnCs inducing GD3 abolished NK cell degranulation in vivo (Fig. 2e) or in vitro (Fig. 2f). The NK cell recruitment capacity was not modified by the expression of GD3 (Fig. 2g and Extended Data Fig. 2b–d), demonstrating an uncoupling between SASP chemoattractant functions and GD3 immunosuppressive roles. Accordingly, sialic acid abolition using neuraminidase (Fig. 4a) or GD3 removal via knockdown (KD) of *ST8SIA1* (Extended Data Fig. 5a,b) totally rescued the capacity of SnCs to activate NK cell degranulation (Fig. 4a and Extended Data Fig. 5d) but did not increase IFN-γ production (Extended Data Fig. 5i). Conversely, *ST8SIA1* overexpression leading to GD3 expression in young dividing cells (Extended Data Fig. 5e–g) inhibited NK cell degranulation, a process impaired by neuraminidase treatment (Fig. 4c and Extended Data Fig. 5h). Knocking down *ST8SIA1* expression in SnCs did not alter the senescence state, as evidenced by a similar proportion of SA-β-Gal^+^ cells and the absence of proliferation (Extended Data Fig. 5a–d). Conversely, proliferative cells forced to overexpress *ST8SIA1* did not enter senescence and remained proliferative (Extended Data Fig. 5g). These observations revealed that, if GD3 upregulation can be linked to the senescence program, GD3 expression does not induce senescence. GD3 targeting by an anti-GD3 monoclonal antibody (mAb) was sufficient to restore murine NK cell degranulation (Fig. 4d) in a dose-dependent manner (Extended Data Fig. 6a–d) as well as human primary NK cell degranulation (Fig. 4e and Extended Data Fig. 6f) and killing (Extended Data Fig. 6e). The immunosuppressive capacities of GD3^+^ SnCs affected NK degranulation but did not appear to significantly affect IFN-γ production capacities (Extended Data Figs. 2f,g, 5i and 6b,d). This is consistent with the fact that IFN-γ production can be induced by signals from other immune cells, such as dendritic cells or macrophages. This confirms the hypothesis that GD3-dependent NK cell inhibition does not rely on SASP or on the action of another immune compartment. The magnitude of the inhibition induced by GD3 in SnCs on NK cells was assessed by rechallenging the NK cells with the strong immunogenic cancer cell line YAC-1 (Fig. 4f). The presence of GD3 in SnCs was sufficient to deeply inhibit the function of NK cells, rendering them completely hypofunctional against YAC-1 cells (Fig. 4g). This effect was entirely alleviated by adding anti-GD3 mAb in the co-culture (Fig. 4f,g), showing that GD3 can favor cancer immune escape in SnCs. This suggests that GD3^+^ SnC accumulation upon aging favors the emergence of age-associated cancers by an early blockade of the NK-cell-dependent immune surveillance. Altogether, these results demonstrate that the level of GD3 in senescent cells determines their ability to escape elimination by NK cells.

### GD3 SnCs are present in senescence-associated diseases

**Fig. 5:**
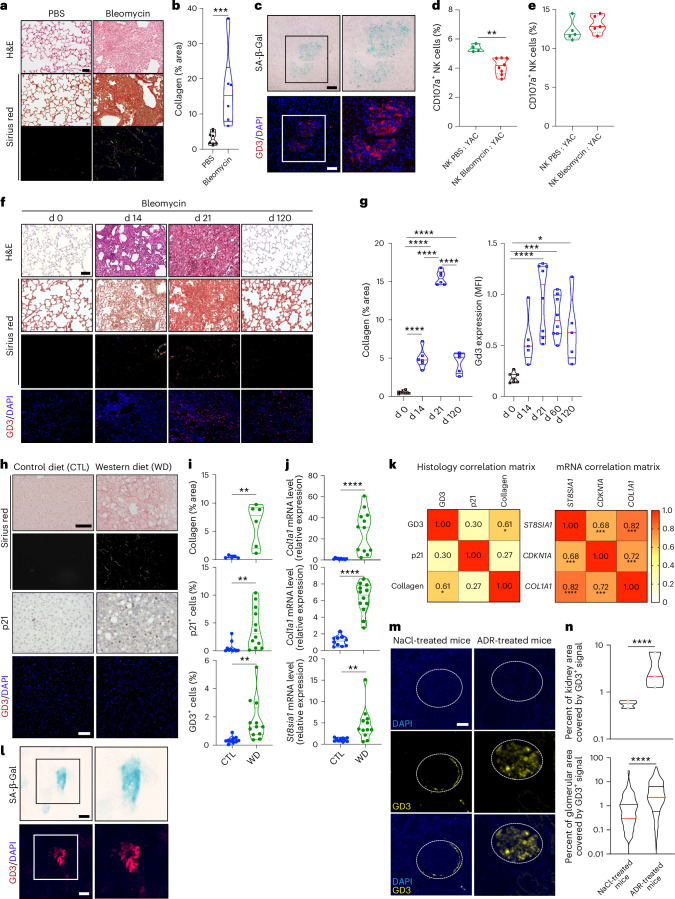
GD3^+^ SnCs present, accumulate and persist in experimental models of senescence-associated diseases and inhibit NK cell functionality. **a**,**b**, H&E or picosirius red staining (in white or polarized light) in bleomycin-induced lung fibrosis sections (**a**) and quantification of collagen deposit (sirius red staining; **b**) (scale, 100 μm). **c**, SA-β-Gal assay and GD3 expression analysis (**b**) corresponding to fibrotic lungs used in Fig. 7a–d (scale, 100 μm). **d**,**e**, Determination of the intrapulmonary (**d**) and splenic (**e**) NK cell functionality ex vivo from control or bleomycin-instilled mice against YAC-1 cells after 4 h of rechallenge. **f**,**g**, Histology analysis, quantification of collagen deposit (sirius red staining in white or polarized light) and GD3 expression in mean intensity fluorescence (MFI) in fibrotic lungs over time (**f**) and their quantification (**g**) (scale, 100 μm). **h**–**j**, Livers of mice fed with Western or control diets stained with picrosirius red (in white or polarized light) and labeled for p21 in IHC or GD3 in IF (**h**), their histological quantification (**i**) and mRNA quantification by qPCR (**j**). **k**, Pearson coefficient correlation analysis for GD3, p21 and collagen fibers (left panel) and their corresponding gene expressions (right panel). **l**, SA-β-Gal assay and GD3 expression analysis in ADR-induced kidney fibrosis. **m**, GD3 expression in kidney glomeruli from ADR-treated or control (NaCl) mice. **n**, Quantification of the percentage of kidney area (left panel) or the glomerular area (right panel) covered by GD3^+^ signal in ADR-treated or control (NaCl) kidney (scale, 10 μm). Experiments in **a**–**g** were performed with *n* = 8 mice per group. Experiments in **h** and **k** were performed on *n* = 5 mice in the control group and on *n* = 6 mice in the Western diet group. Experiements in **i** and **j** were performed on *n* = 10 mice in the control group and on *n* = 12 mice in the Western diet group. Experiments in **l**–**n** were performed with *n* = 4 mice per group or at least 461 glomeruli. *P < 0.05, **P < 0.01, ***P < 0.001 and ****P < 0.0001. **b**–**g**,**n**, Two-tailed Mann–Whitney *U*-test. **i**,**j**, Two-tailed unpaired *t*-test. **k**, Pearson correlation. d, day. Source data

**Fig. 6:**
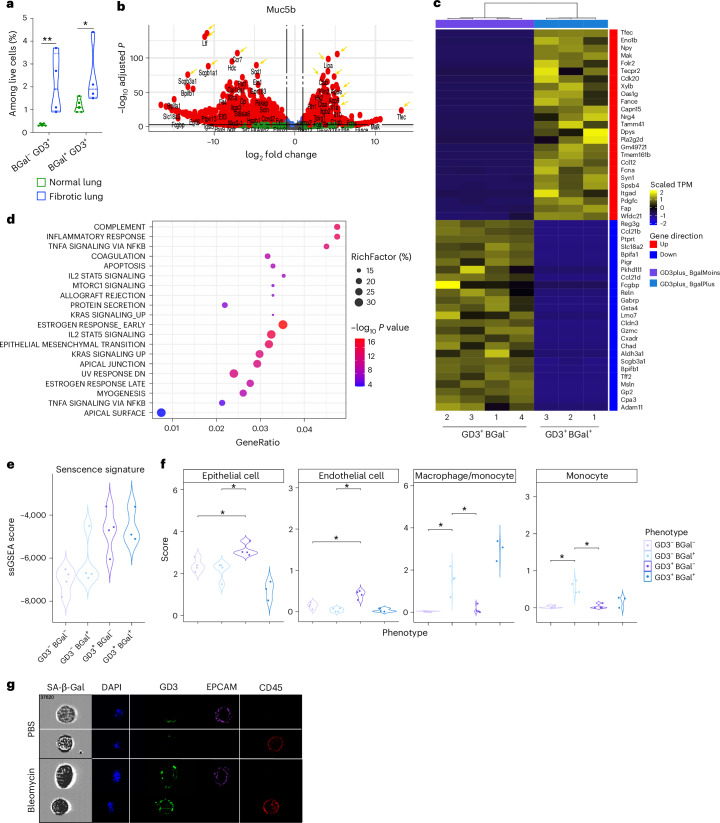
GD3 is a senescence-associated surface marker in bleomycin-induced fibrotic lungs. **a**, Quantification by flow cytometry of the frequency of SA-β-Gal^+/−^ cells and GD3^+/−^ cells in fibrosis-bearing lungs. **b**–**d**, Volcano plot (**b**), heatmap of DEGs (**c**) and KEGG pathway analysis (**d**) between sorted GD3^+^SA-β-Gal^−^ and GD3^+^SA-β-Gal^+^ cells in bulk RNA-seq. Important senescence pathways or genes are highlighted or pinpointed in yellow. **e**,**f**, Senescence ssGSEA (**e**) and deconvolution analysis (**f**) of the GD3^+/−^ and SA-β-Gal^+/−^ sorted fractions. **g**, Characterization by ImageStream^X^ of senescent cells using SA-β-Gal assay, GD3, EPCAM and CD45 staining. Experiments were performed with *n* = 4 mice per group. *P < 0.05, two-tailed Mann–Whitney *U*-test. Source data

To assess the physio-pathological impact of NK cell surveillance alleviation by GD3-expressing SnCs, we analyzed the GD3 expression by SnCs in injured lungs. Indeed, recent studies have established a clear link among SnC accumulation, fibrosis and lung disease ^17,22,23^, a process that can be studied using intratracheal instillation of bleomycin in mice^24^. As expected, bleomycin treatment increased collagen deposition (Fig. 5a,b). The fibrotic areas contain SA-β-Gal^+^ cells expressing GD3 (Figs. 5b,c and 6a). Fluorescent SA-β-Gal assay and GD3 staining analyzed by flow cytometry revealed a two-fold increase in GD3^+^SA-β-Gal^+^ cells and a more than three-fold overall increase in GD3^+^ cells compared to normal lungs (Fig. 6a). In contrast to human lung fibrosis, bleomycin-induced fibrosis regressed spontaneously^25^. Indeed, we observed that the fibrotic lesion partially regressed at day 120 (Fig. 5f,g), with a significant decrease in collagen deposition (Fig. 5g, left panel) but a similar intensity of GD3 (Fig. 5g, right panel), suggesting the persistence of GD3^+^ SnCs within fibrotic sequelae. In fibrotic lungs at day 27, we observed a slight alteration in global immune infiltration with more NK cells (Extended Data Fig. 5j). However, these NK cells degranulated less against YAC-1 cells in ex vivo rechallenge assay than their control counterparts (Fig. 5d). Because the function of NK cells localized away from the lungs (spleen) was not impacted by the bleomycin treatment (Fig. 5e), the GD3^+^ SnCs of the lung are unlikely to have systemic effects on NK cell inhibition. Such an association of GD3^+^ SnCs to fibrosis was not restricted to lung injuries.

We found GD3^+^ SnCs in experimental murine liver fibrosis and steatohepatitis induced by a Western diet (Fig. 5h–k) and in kidney fibrosis induced by adriamycin (ADR) treatment (Fig. 5l–n). In the fibrotic livers where p21, GD3 and sirius red stainings were increased, collagen deposition correlated with GD3 expression (Fig. 5i,k). Similarly, *Col1a1*, *St8sia1* and *Cdkn1a* mRNA expression levels were increased and correlated altogether (Fig. 5j,k). A marked induction of GD3 was observed in ADR-injured kidneys (Fig. 5l,m), which was significant for both total kidney area (Fig. 5n, upper panel) and glomeruli area (Fig. 5n, lower panel).

### GD3 as a cell surface senescence marker in vivo

**Extended Data Fig. 7:**
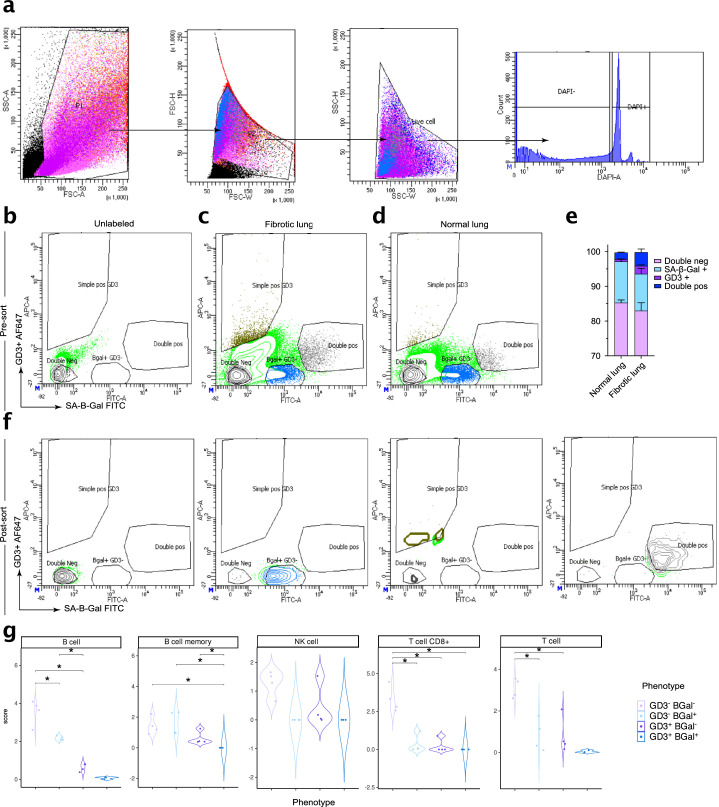
The proportion of GD3 positive senescent cells increases in murine fibrotic lungs and can be sorted by FACS. **a**, Gating strategy for the sorting of the sequenced fractions analyzed in Fig. 6. **b-d**, Position of the sorting gates for the unlabeled fraction (**b**), a fibrotic lung (**c**) and a normal lung (**d**). **e**, Proportion of the different fractions analyzed by flow cytometry for normal and fibrotic murine lungs for n = 4 animals, data represent the mean ± s.e.m. **f**, Post sort quality control of the sorting of the different fractions. **g**, Deconvolution analysis of the GD3 + /- and SA-β-Gal + /- sorted fractions. Experiments are performed with n = 4 mice per group; *p < 0.05, two-tailed Mann–Whitney *U* test. Source data

To comprehensively characterize the senescent state of the GD3^+^ cells within fibrotic lungs, we sorted 40,000 of them by flow cytometry based on GD3 expression and SA-β-Gal activity (Fig. 6a,b and Extended Data Fig. 7) and analyzed them by RNA-seq (GSE262926; Supplementary Table 2). We compared specifically GD3^+^SA-β-Gal^+^ and GD3^+^SA-β-Gal^−^ cells by differential gene expression analysis (Supplementary Table 3). Differentially expressed genes (DEGs) are represented by volcano plot (Fig. 6b) and heatmap (Fig. 6c). Even though both populations modulate different senescence-associated genes, we observed that they have different profiles depending on SA-β-Gal activity. Molecular Signatures Database (MSigDB) hallmark pathway^26^ analysis showed that both populations were enriched in senescence-associated pathways (Fig. 6d) including SASP factors and revealing two populations of GD3^+^ senescent cells. The comparison of the four subpopulations by single-sample gene set enrichment analysis (ssGSEA)^27^ analysis revealed that only GD3^+^ cells, independently of the SA-β-Gal activity, showed a senescence signature (Fig. 6e). Finally, using the marker gene-based method (MCP-counter), we showed that SA-β-Gal^+^GD3^+^ cells predominantly correspond to senescent macrophages, whereas SA-β-Gal^−^GD3^+^ cells correspond to senescent endothelial and epithelial cells (Fig. 6f). Using imaging flow cytometry, GD3 expression can be observed at the cell surface of both senescent epithelial cells (EpCAM^+^CD45^−^ cells) or senescent immune cells (CD45^+^ cells) (Fig. 6g). Consistently, the transcriptome of SA-β-Gal^+^GD3^−^ cells was not enriched in senescence-associated genes (Fig. 6e) and corresponds essentially to a non-senescent macrophage/myeloid population. These findings show that GD3 is overexpressed in SnCs, regardless of their cell lineage, and highlight the high potential of GD3 to be a novel marker for a better SnC identification, in conjunction with SA-β-Gal.

### Targeting GD3 by immunotherapy improves the development of senescence-related and age-related diseases

**Fig. 7:**
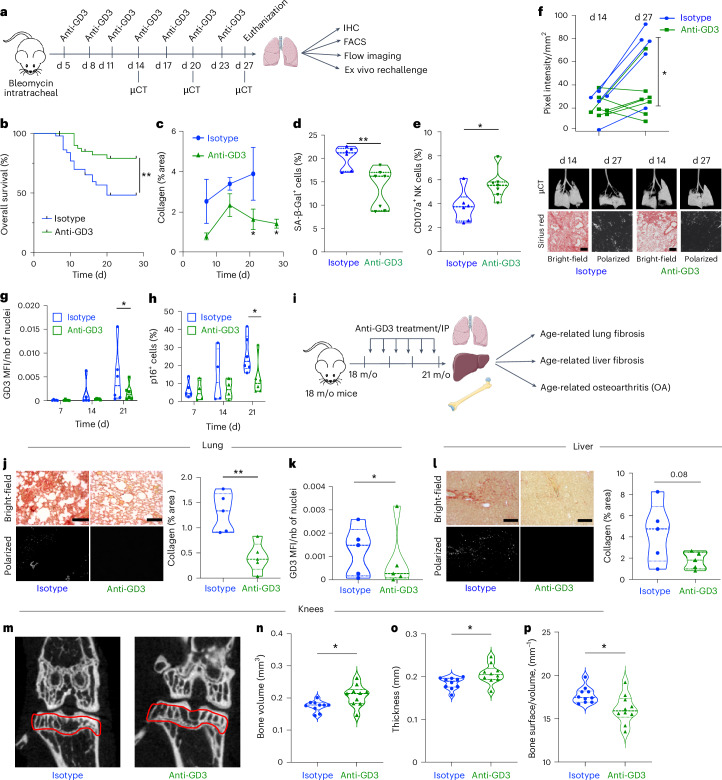
GD3 targeting in vivo increases overall mouse survival and reduces senescence-associated diseases by locally restoring NK-cell-mediated immunosurveillance. **a**, Representative scheme of in vivo anti-GD3 mAb treatment in lung fibrosis model. **b**, Overall survival analysis of fibrotic mice (*n* = 48 in control group and *n* = 45 in anti-GD3 group). **c**, Evaluation of the fibrosis over time by quantification of collagen fibers using picosiruis red staining (*n* = 5 mice per group and per timepoint; data represent the mean ± s.e.m.). **d**, Quantification by flow cytometry of SA-β-Gal^+^ cells infiltrating lungs at d27. **e**, Evaluation by flow cytometry of intrapulmonary NK cell functionality at d27. **f**, Evaluation of lung fibrosis by μCT imaging (d14 and d27) and picosiruis red staining (d27) (d14 and d27 µCT and picrosirius red images representing the same mouse over time for each group) (scale, 100 μm). **g**,**h**, Quantification of the mean fluorescence intensity in function of number of nuclei (MFI/nb) of GD3 by IF (**g**) and the percentage of p16^+^ cells by IHC over time (**h**). **i**, Representative scheme of the in vivo aging experiment. **j**, Evaluation of age-related lung fibrosis by collagen quantification using picosirius red staining (*n* = 5 mice per group) (scale, 100 μm). **k**, Quantification of the MFI/nb of GD3 by IF in the lungs (*n* = 5 mice per group). **l**, Evaluation of age-related liver fibrosis by collagen quantification using picosirius red staining (*n* = 5 mice per group) (scale, 100 μm). **m**–**p**, μCT high-resolution imaging (**m**) and quantification of the bone volume (**n**), thickness (**o**) and ratio between bone surface and volume (**p**) of the bone structure of the knees of the mice treated with isotype of anti-GD3 (*n* = 5 mice, 10 legs per group). All bleomycin-induced fibrosis experiments were performed with *n* = 8 mice per group. *P < 0.05, **P < 0.01 and ***P < 0.001; two-tailed Mann–Whitney *U*-test (**a**–**p**). d, day; m/o, months old. Source data

**Extended Data Fig. 8:**
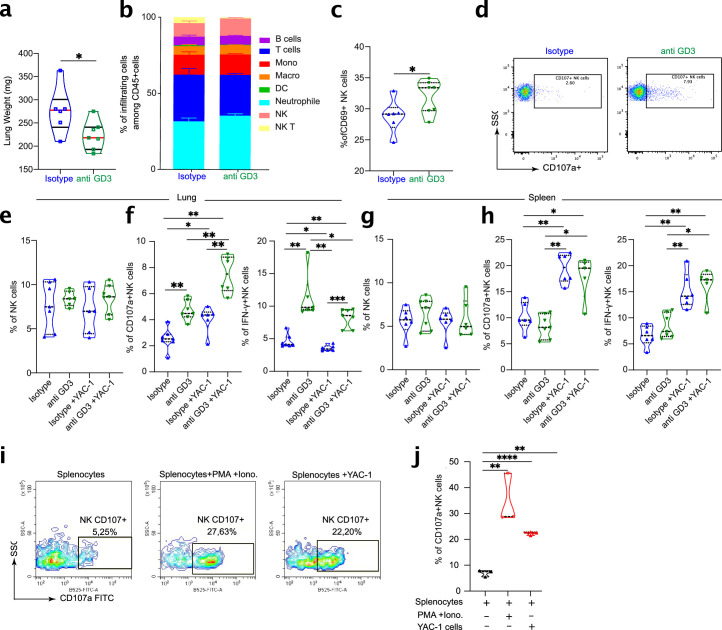
Anti GD3 targeting in vivo restore NK cell functionality locally in vivo and ex vivo. **a**, Lung weight at d27 after instillation with Bleomycin in function of the treatment with the anti-GD3 antibody. **b**, Analysis of the immune infiltration in the lungs by flow cytometry of isotypic control or anti GD3 mAb treated fibrotic mice used in Fig. 7a-h. **c**, Evaluation by flow cytometry of the percentage of CD69+ activated intrapulmonary NK cells at d27; data corresponding to Fig. 7e. **d**, Density plot for the quantification of NK degranulation corresponding to the Fig. 4e. **e**, Determination of the quantity of NK cells within the lung of fibrotic mice depending on the treatment. **f**, Determination of the intrapulmonary NK cell functionality ex vivo from treated or control mice against YAC-1 cells after 4 hours of rechallenge. **g**, Determination of the quantity of NK cells within the spleen of fibrotic mice depending on the treatment. **h**, Determination of the NK cell functionality ex vivo from the spleen of treated or control mice against YAC-1 cells after 4 hours of rechallenge. **i, j**, NK cell functionality after 24 hours of culture without MRC5 (controls data corresponding to Fig. 2). Experiment done on n > 6 mice (**a-h**); Experiment done on n = 3 experiment (**a-h**). *p < 0.05, **p < 0.01, and ***p < 0.001; two-tailed Mann–Whitney *U* test. Source data

Next, we investigated whether anti-GD3 mAb treatment could inhibit the tolerogenic effects of SnCs in vivo (Fig. 7a–h). Mouse anti-GD3 treatment significantly improved overall survival (Fig. 7b), decreased collagen accumulation (Fig. 7c) and significantly reduced bleomycine-induced lung fibrosis, as determined by lung weight (Extended Data Fig. 8a) and lung density measurement via in vivo micro-computed tomography (µCT) imaging (Fig. 7f). The anti-GD3 treatment was sufficient to block disease progression, and even to reverse it for some mice (Fig. 7f, upper panel), as confirmed through histological analyses at the end of treatment (Fig. 7f, lower panel). The anti-fibrotic effect of anti-GD3 treatment is associated with a decreased SA-β-Gal^+^ cell population (Fig. 7d), a decreased GD3 expression (Fig. 7g) and a decrease of p16^+^ cells (Fig. 7h). The anti-GD3 mAb was sufficient to restore intrapulmonary NK cell degranulation (Fig. 7e) and activation (Extended Data Fig. 8e,f), whereas the overall proportion of immune cells was not affected (Extended Data Fig. 8b). Although the NK cell proportion in the lung (Extended Data Fig. 8e) or the spleen (Extended Data Fig. 8g) was not changed by the treatment, lung NK cell function (degranulation and IFN-γ production) against YAC-1 was strongly enhanced in ex vivo rechallenge experiment (Extended Data Fig. 8f). In contrast, anti-GD3 mAb treatment did not alter spleen NK cell function (Extended Data Fig. 8h), showing that NK cell inhibition is restricted to the lung and is GD3 dependent. Collectively, these results show that the bypass of NK cell immune surveillance by GD3 leads to SnC accumulation and sustains the development of lung fibrosis. Such an association of GD3^+^ SnCs with fibrosis was not restricted to lung injuries. We found GD3^+^ SnCs in experimental murine liver fibrosis and steatohepatitis induced by a Western diet (Fig. 5h–k) and in kidney fibrosis induced by ADR treatment (Fig. 5l–n). In the fibrotic livers where p21, GD3 and sirius red stainings were increased, collagen deposition correlated with GD3 expression (Fig. 5i,k). Similarly, *Col1a1*, *St8sia1* and *Cdkn1a* mRNA expression levels were increased and correlated altogether (Fig. 5j,k). A marked induction of GD3 was observed in ADR-injured kidneys (Fig. 5l,m), which was significant for both total kidney area (Fig. 5n, upper panel) and glomeruli area (Fig. 5n, lower panel).

**Extended Data Fig. 9:**
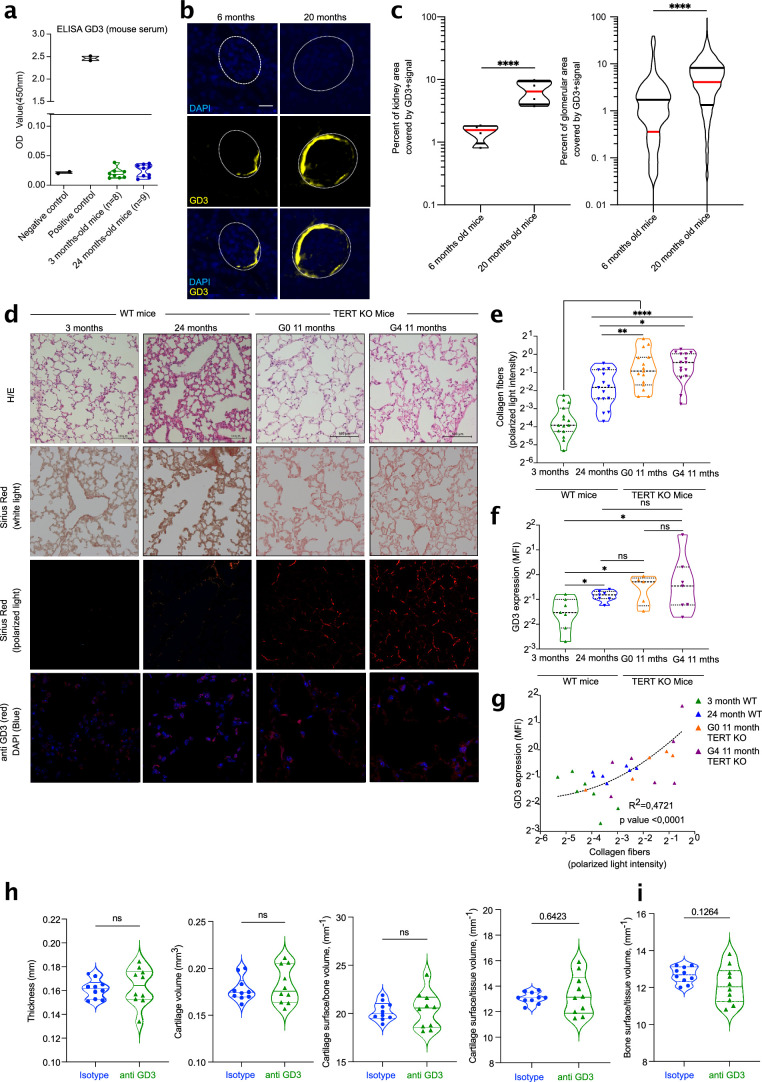
GD3 is not secreted in the serum of old mice but is strongly increased by senescent cells found in natural Age-associated kidney and lung fibrosis. **a**, GD3 dosage by ELISA in serum of 3 months or 24 months-old mice (right panel, 8 to 9 mice per group). **b**, GD3 expression in kidney glomeruli from 6-months or 20-months old mice. (Scale =10μm) **c**, Quantification of the percentage of kidney area (left panel) or the glomerular area (right panel) covered by GD3 + signal in 6-months or 20-months old kidney. **d**, H&E, Sirius Red (in white and polarized light) and GD3 immunofluorescence staining in young (3 months), old (24 months) and telomerase KO mice in G0 and G4 (11 months) (Scale =100μm)**. e,f**, Quantification of collagen deposition (**e**) and GD3 expression in lungs (**f**). **g**, Correlation between collagen fibers quantity and GD3 expression in aged mice. Data represent the Pearson uncentred correlation. **h, i**, Analysis of the knee’s cartilage (**h**) and bone (**i**) thickness, volume, and ratio surface/volume (S/V) between isotype and anti-GD3 (n = 5 mice, 10 legs per group), p-values calculated by two-tailed Mann-Whitney test. **a-c** experiments are performed with n = 4 mice per group or at least 461 glomeruli; *p < 0.05, **p < 0.01, and ***p < 0.001; Mann–Whitney *U* test. **d-f** experiments are performed with n = 8 mice per group. *p < 0.05, **p < 0.01, and ***p < 0.001; two-tailed Mann–Whitney *U* test. Source data

**Extended Data Fig. 10:**
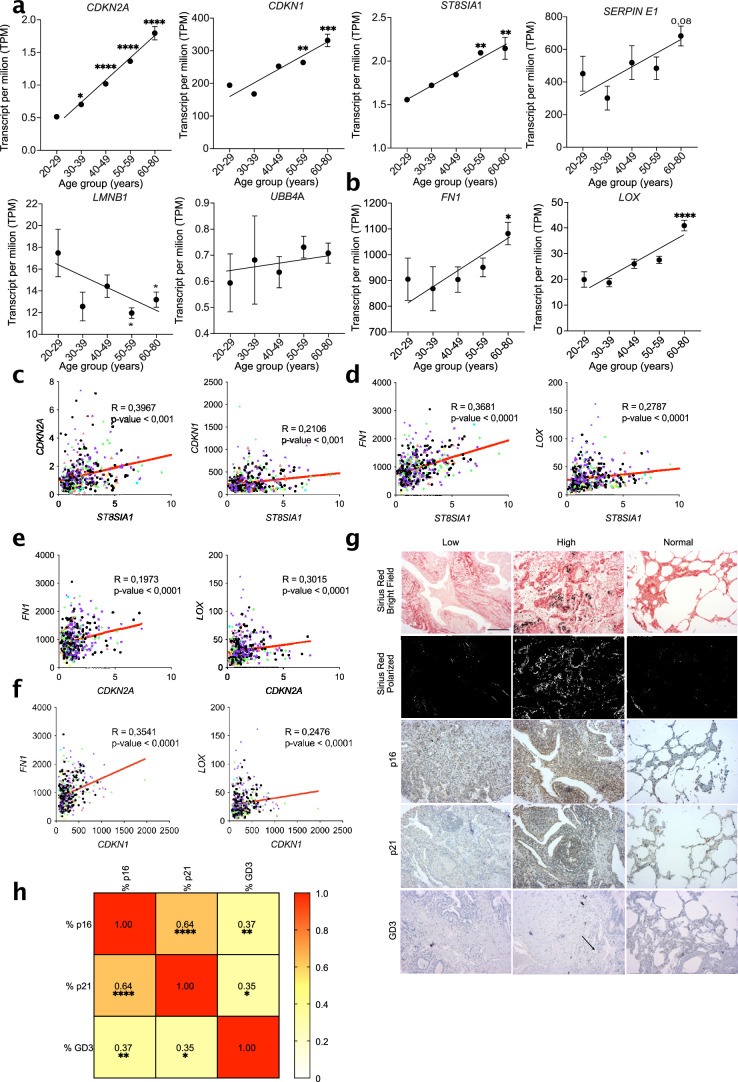
ST8SIA1 and GD3 expression increases with age and fibrosis in human lung and correlates with the expression of senescence markers upon aging. **a, b**, Analysis of gene expression by RNAseq of normal human lung samples from different ages. Data are extracted from GTEX consortium and the relative gene expression (transcript per million or TPM) is represented in function of the group of age for senescence associated genes (**a**) or fibrosis associated genes (**b**). Data represent the mean ± s.e.m of n = 427 patients. *p < 0.05, **p < 0.01, and ***p < 0.001; two-way Anova test. **c-f**, Gene expression correlation (in TPM) between ST8SIA1 and CDKN2A or CDKN1 gene expression (**c**); ST8SIA1 and FN1 or LOX (**d)**; CDKN2A and FN1 or LOX (**e**); or CDKN1 and FN1 or LOX (**f**). Data represent the Pearson uncentred correlation *p < 0.05, **p < 0.01, ***p < 0.001 and ****p < 0.0001. **g**, Picrosirius red staining (in white or polarized light), p16, p21 and GD3 labeling in IHC in pulmonary interstitial fibrosis and normal samples (commercial tissue microarray TMA from USBiomax). Data representative of n = 52 patients. (Scale =200μm). **h**, Pearson correlation matrix between histological quantification of p16, p21and GD3 (n = 52). Pearson correlation *p < 0.05, **p < 0.01, ***p < 0.001 and ****p < 0.0001. Source data

We then investigated the expression of GD3 upon aging. GD3 was not found in a secreted or cleaved form in the serum of old mice (Extended Data Fig. 9a), but its expression was significantly increased in SnCs found in old kidneys (Extended Data Fig. 9b,c) or old lungs (Extended Data Fig. 9d–f). The age-related low-grade lung fibrosis (Extended Data Fig. 9d,e) strongly correlated with the expression of GD3 within the lung (Extended Data Fig. 9f,g). Moreover, using Genotype-Tissue Expression (GTEx) consortium RNA-seq data^28^, we observed a significant increase of *ST8SIA1* expression in human aged lungs (Extended Data Fig. 10a,b) that was significantly correlated with senescence-related genes (*CDKN2A* and *CDKN1A* expression) and fibrosis-related genes (*FN1* and *LOX* expression) (Extended Data Fig. 10c–f). Such correlation between collagen deposition and the frequencies of p16, p21 and GD3^+^ cells (Extended Data Fig. 10g,h) was also observed in tissue microarray (TMA) analysis, comprising 24 human lung fibrosis samples, mirroring the associations found in the GTEx study. These analyses indicate that, upon human aging, GD3^+^ SnCs accumulate within tissues where they are associated with fibrosis.

Finally, we assessed the therapeutic potential of anti-GD3 immunotherapy in old mice (18 months). A six-dose intraperitoneal regimen over 3 months significantly reduced collagen deposition in the lungs (Fig. 7i,j) and GD3 expression (Fig. 7k), suggesting an anti-fibrotic effect in the lungs. Notably, the systemic treatment also impacted age-related liver fibrosis, with decreased collagen deposition (Fig. 7l). Furthermore, the anti-GD3 treatment had a protective effect against age-related bone remodeling in joint. µCT and confocal imaging revealed increased bone volume (Fig. 7m,n) and bone thickness (Fig. 7o) and reduced subchondral bone erosion (Fig. 7p), despite the absence of significant changes in cartilage structure (Extended Data Fig. 9l,m). These findings demonstrate the therapeutic potential of targeting GD3 to attenuate multiple age-related pathologies, including lung and liver fibrosis, as well as age-related bone remodeling in joint.

## Discussion

In summary, we revealed that human and mouse SnCs can exhibit strong immunosuppressive functions in vivo, with the ability to inhibit NK cell function toward senescent and cancer cells through a GD3-dependent pathway. Recent data showed that pre-oncogenic SnCs can be immunosuppressive and limit their elimination via the SASP by recruiting myeloid cells^16^ or by regulating the HLA-E pathway^18^ or by overexpressing PD-L1 (ref. ^14^). Here we show that non-oncogenic SnCs control their immune surveillance in an SASP-independent mechanism by upregulating GD3 expression at their surface, thereby determining their immune escape. This finding provides a mechanism by which some SnCs evade from immune surveillance within tissues by the immune system and, thus, can accumulate with age. Accordingly, in a model of severe lung fibrosis, GD3 targeting by mAb is sufficient to improve overall survival, reduce lung fibrosis and restore cancer immunosurveillance. Notably, anti-GD3 treatment in old mice was sufficient to decrease age-related lung and liver fibrosis as well as age-related bone remodeling associated with osteoarthritis (OA) disease, opening new therapeutic approaches to treat age-related disabilities. It is noteworthy that the OIS cells behave in an opposite way, downregulating GD3 level, a mechanism that could have evolved to favor the elimination of pre-cancerous cells. We propose that GD3 is an SIC whose level determines SnC immune surveillance. Our results show that SICs are promising targets for therapies of age-related diseases.

## Methods

The research conducted in this study complies with local and institutional guidelines. The study protocol, including all procedures involving animals, was reviewed and approved by the Comité Institutionnel d’Éthique Pour l’Animal de Laboratoire, registered at the French Ministry of Higher Education and Research under number 28. The research was conducted under authorization numbers 2017020215478898, 16319-2018071917443610v2, 2015102215087555, 2020042723583497, 2023051709131709v5 and 2022012015452005. Peripheral blood mononuclear cells (PBMCs) from healthy donors were collected with informed consent from the donors and were obtained through the Etablissement Français du Sang (agreement no. 22-093).

### Cells

MRC5 cells (American Type Culture Collection (ATCC), CCL-171), WI-38 cells (ATCC, CCL-75), normal hMECs (ATCC, PCS-600-010) and MEFs (ATCC, SCRC-100) were grown in DMEM (Thermo Fisher Scientific, 21885108) supplemented with 10% of FBS and 1% of penicillin–streptomycin at 37 °C with 5% CO_2_. hMECs and MRC5 cells were modified to overexpress in inducible manner h-Ras by M.-C.M. and D.B., respectively.

YAC-1 cells (ATCC, TIB-160) were cultured in RPMI medium (Thermo Fisher Scientific, 61870044) supplemented with 10% of FBS and 1% of penicillin–streptomycin (Invitrogen) at 37 °C with 5% CO_2_. Mycoplasma tests were performed every 3 months, and experiments were performed only on mycoplasma-negative cells.

### Induction of senescence

Replicative senescence was induced by continuous passaging of MRC5 primary human fibroblasts at 5% O_2_ until they reached a plateau in their growth curve. Cumulative population doubling level (pdl) was calculated using the following equation:

$${\rm{pdl}}=\frac{\log {n}_{c}-\log {n}_{s}}{\log 2}$$

where *n*_c_ represents the number of cells counted after expansion and *n*_s_ represents the number of cells seeded. For all experiments, young cells were used at pdl30, and SnCs were defined as at least 90% SA-β-Gal^+^ and no more than 10% of EdU^+^ cells. MRC5 primary human fibroblasts were induced to senescence by exposure to X-ray radiation at a total dose of 10 Gy at a rate of 5 Gy min^−1^, treated with Nutlin 3a at 10 µM for 24 h, CCN1 2.5 µg ml^−1^ for 6 d, bleomycin during 24 h at 50 µg ml^−1^ and aphidicolin during 24 h at 2.5 µg ml^−1^. MEFs were induced in senescence by exposure to X-ray radiation at a total dose of 10 Gy at a rate of 5 Gy min^−1^. OIS was induced in hMECs, and MRC5 fibrobalsts were transduced with a vector expressing H-RASG12V. hMECs were treated with doxycycline hyclate (Sigma-Aldrich, D9891) at 1 µg ml^−1^ to activate H-RASG12V during 5 d or 12 d, and MRC5 cells were treated with 4-hydroxytamoxifen (Sigma-Aldrich, H7904) at 50 nm every 48 h for 8 d. Mycoplasma test was performed every month, and experiments were performed only on mycoplasma-negative cells.

### Animals—Matrigel plug assays

Experiments were performed on 8–10-week-old female NMRI nude mice from Charles River Laboratories. In total, 500,000 cells in 100 µl of PBS or the equivalent of the supernatant of the same cells concentrated by Amicon 10 kDa (Milipore) in 100 µl of PBS and complemented with 400 µl of Matrigel growth factor reduced (Corning, 354230) were inoculated subcutaneously on the back under isoflurane anesthesia. Five days later, mice were euthanized, and Matrigel plugs were harvested. Infiltrating cells were recovered after enzymatic dissociation by Dispase (Corning, 354235), Collagenase A and DNAse I (Roche, 05952077103) digestion for 30 min at 37 °C. Infiltrating cells were stained with directly coupled antibodies for 30 min at 4 °C after saturation with Fc-Block anti-CD16/CD32 antibodies (clone 2.4G2) for 15 min on ice. After washes in 0.5 mM EDTA 2% FBS PBS, cells were analyzed using a FACSAria III cytometer (BD Biosciences) with DIVA6 and FlowJo 10 software.

### Mouse model of ADR-induced kidney injury

A single dose of 12 mg kg^−1^ ADR (doxorubicin hydrochloride; Sigma-Aldrich, D1515) was injected in the tail vein of 3-month-old BALB/c female mice (Charles River Laboratories). Littermates were injected similarly with saline (NaCl 0.9%). All mice were weighed twice a week in the timecourse of the experiments. Kidneys of the mice were collected 14 d after injection, inflated with 4% (w/v) formaldehyde in PBS, embedded in paraffin and sectioned for further histological analysis. BALB/c mice were obtained from Janvier Labs (strain BALB/cJRj).

### Bleomycin-induced lung fibrosis

Next, 8–10-week-old pathogen-free male C57BL/6 mice (Charles River Laboratories) were treated with intratracheal instillation of 50 µl of PBS or bleomycin (2.5 U kg^−1^; Sigma-Aldrich, B8416-15UN). Animals were maintained on a 12-h light/dark cycle with food and water ad libitum. After the experiments, mouse lung tissues were excised and either included in OCT frozen or inflated with 4% (w/v) paraformaldehyde in PBS, embedded in paraffin, sectioned and stained with hematoxylin and eosin (H&E) and picrosirius red. The percentage of collagen was quantified with ImageJ. Alternatively, lungs were freshly dissociated with a Miltenyi Lung Dissociation Kit (ref. no. 130-905-927) and gentleMACS with Heaters. Infiltrating cells were stained with directly coupled antibodies for 30 min at 4 °C after saturation with Fc-Block anti-CD16/CD32 antibodies (clone 2.4G2) for 15 min on ice. After washes in 0.5 mM EDTA 2% FBS PBS, cells were analyzed using a FACSAria III cytometer with DIVA6 and FlowJo 10 software.

### Mice and Western diet

Three-month-old C57BL/6J/Rj wild-type mice were acclimated under a 12-h light/dark cycle at a temperature of 21 ± 2 °C. They were fed ad libitum either a Western diet 21% fat (MP Biomedicals, Western diet, 7293508) or a chow diet, and the drinking water was supplemented with 15% fructose for 22 weeks. Livers were collected and fixed in 10% buffered formalin, embedded in paraffin, sectioned (5 μm thick) and stained with either H&E or picrosirus red.

### Anti-GD3 treatment in old mice

Eighteen-month-old pathogen-free male C57BL/6J mice (Charles River Laboratories) were treated six times every 15 d with 150 µg of anti-GD3 in 150 µl of PBS intraperitoneally. Animals were maintained on a 12-h light/dark cycle with food and water ad libitum. After the experiments, lungs, livers and legs were collected. Mouse lung and liver tissues were excised and either included in OCT frozen or fixed with 4% (w/v) formaldehyde in PBS, embedded in paraffin, sectioned and stained with H&E and picrosirius red. The percentage of collagen was quantified with ImageJ. For all mouse experiments, the number of animals needed was determined using a Monte Carlo power test before experiments. All mouse experiments were conducted according to local and international institutional guidelines and were approved by either the Animal Care Committee of the Institute of Research on Cancer and Aging in Nice (IRCAN) or the regional (CIEPAL Côte d’Azur Agreements NCE/2015-266 no. 2015102215087555 and NCE/2020-675 no. 2020042723583497) and national (French Ministry of Research) authorities.

### SA-β-Gal staining on cultured cells

Cells were fixed and stained using an SA-β-Galactosidase Staining Kit (Abcam, ab65351), following the manufacturer’s instructions. After staining, cells were incubated for 12–14 h at 37 °C and then visualized by phase-contrast microscopy. The percentage of SA-β-Gal^+^ cells was calculated by determining the ratio of SA-β-Gal^+^ cells (blue staining) among all cells counted. A minimum of 150 cells were counted for each condition, and proliferative MRC5 cells (pdl30) were used as negative control for each experiment.

### EdU proliferation assay on cultured cells

To measure cell proliferation, an EdU assay was performed following instructions from Thermo Fisher Scientific. MRC5 cells and hMECs were plated in 24-well plates at 5 × 10^4^ cells per well for 12 h and then incubated in 50% conditioned medium and 50% serum-free DMEM with 10 μmol L^−1^ EdU (Invitrogen/Click-IT EdU Alexa Fluor 647 Kit) for 12 h. Cells were fixed and underwent DNA staining to detect cycling cells. Confocal fluorescence microscopy (Zeiss, LSM880) was used to image the cells, and the percentage of proliferative cells was calculated as the ratio of EdU^+^ cells (red nucleus) to total cells counted (DAPI^+^), with at least 100 cells assessed per condition. Proliferative MRC5 cells (pdl30) served as a positive control.

### Extraction and purification of glycolipids

Cells (2 × 10^7^) were lyophilized and extracted three times with CHCl_3_/CH_3_OH (2:1, v/v) and once by CHCl_3_/CH_3_OH (1:2, v/v) using intermediary centrifugations at 2,500*g* for 20 min. Combined supernatants were dried under a nitrogen stream, subjected to mild saponification in 0.1 M NaOH in CHCl_3_/CH_3_OH (1:1, v/v) at 37 °C for 2 h and evaporated to dryness. Samples were reconstituted in CH_3_OH/0.1% TFA in water (1:1, v/v) and applied to a reverse-phase C_18_ cartridge (Waters) equilibrated in the same solvent. After washing with CH_3_OH/0.1% TFA in water (1:1, v/v), glycosphingolipds (GSLs) were eluted by CH_3_OH, CHCl_3_/CH_3_OH (1:1, v/v) and CHCl_3_/CH_3_OH (2:1, v/v). The elution fraction was dried under nitrogen stream before structural analysis.

### Sequential release and purification of *N*-glycans and *O-*glycans

Cells were resuspended in 6 M guanidinium chloride and 5 mM EDTA in 0.1 M Tris/HCl, pH 8.4, and agitated for 4 h at 4 °C. Dithiothreitol was added to 20 mM and incubated for 5 h at 37 °C, followed by 50 mM iodoacetamide overnight in the dark at room temperature. The reduced/alkylated glycoproteins were dialyzed against water at 4 °C for 72 h and lyophilized. Samples were digested with trypsin TPCK (Sigma-Aldrich) at a 20:1 ratio (w/w) in 50 mM NH_4_HCO_3_, pH 8.5, for 24 h at 37 °C. Digestion was stopped by heating at 100 °C for 5 min, followed by C18 Sep-Pak chromatography. C18 Sep-Pak was equilibrated in 5% acetic acid, and bound peptides were eluted with 20%, 40% and 60% propanol in 5% acetic acid, pooled and lyophilized. *N*-glycans were released by digestion with 10 U *N*-glycosidase F (Roche) in 50 mM NH_4_HCO_3_, pH 8.4, overnight at 37 °C. *N*-glycans and *O*-glycopeptides were separated by C18 Sep-Pak using the same protocol. Propanol fractions containing *O*-glycopeptides were pooled and freeze dried. *O*-glycans were liberated by reductive elimination in 1 M NaBH_4_ and 0.1 M NaOH at 37 °C for 72 h. The reaction was stopped by adding Dowex 50 × 8 resin until pH 6.5. After drying, boric acid was distilled with methanol, and the material was purified by cation-exchange chromatography on Dowex 50 × 2 resin.

### Mass spectrometry

Glycans and glycolipids were permethylated according to the method of Ciucanu and Kerek^29^ before mass spectrometry analysis. In brief, samples were incubated with DMSO/NaOH/ICH_3_ during 2 h under sonication. The reaction was stopped with water, and the permethylated glycans were extracted in CHCl_3_ and washed at least seven times with water. Permethylated glycans were solubilized in CHCl_3_ and mixed with 2,5-dihydroxybenzoic acid matrix solution (10 mg ml^−1^ dissolved in CHCl_3_/CH_3_OH (1:1, v/v)) and spotted on a MALDI plate. MALDI-TOF mass spectra were acquired on a Voyager Elite DE-STR mass spectrometer (Perspective Biosystems), and MALDI-TOF/TOF was analyzed on a 4800 Proteomics Analyzer mass spectrometer (Applied Biosystems) in reflectron positive mode by delayed extraction using an acceleration mode of 20 kV, a pulse delay of 200 ns and grid voltage of 66%. For each spectrum, 5,000 laser shots were performed and accumulated.

### IF

To analyze the presence of the ganglioside GD3 at the cell surface of MRC5, we seed 5 × 10^4^ cells in the 24-well plate. Cells were fixed during 10 min at room temperature with the 1× PBS solution containing 4% formaldehyde. For the GD3 staining, we used a primary antibody anti-GD3 R24 (Abcam, 11779) at 1:1,000, overnight at 4 °C. A secondary antibody against mouse whole IgG in FITC was used at 1:3,000 during 1 h at room temperature (Jackson ImmunoResearch, 115-095-003). The autofluorescence of SnCs was reduced by the use of Autofluorescence Eliminator Staining (Merck Millipore, 2160) following the manufacturer’s instructions. The analysis was performed using fluorescence microscopy and used the same time of laser exposure in all conditions.

### Western blotting

Cells were trypsinized, washed in PBS and lysed with RIPA buffer (Sigma-Aldrich) with protease and phosphatase inhibitors (Sigma-Aldrich) for 30 min on ice. Protein concentrations were measured using a BCA Protein Assay Kit (Interchim). Cell lysates (40 μg protein) were diluted in SDS sample buffer with reducing agent (NuPage, Life Technologies) and boiled for 5 min at 95 °C. Proteins were separated by electrophoresis at 150 V for 1 h on 4–20% Mini-Protean TGX gels (Bio-Rad) and transferred onto Amersham Hybond-P PVDF membranes (GE Healthcare). Membranes were blocked, probed with primary antibodies overnight at 4 °C, washed and incubated with HRP-conjugated secondary antibodies (Vector Laboratories, 1:20,000) for 1 h at room temperature. Detection was done using an ECL kit (GE Healthcare). Membranes were stripped at 4 °C with Antibody Stripping Buffer 1× for 10 min before re-probing. Protein bands were quantified using ImageJ software, normalized to α-tubulin. Primary antibodies were mouse monoclonal anti-ST8SIA1 (R&D Systems) and anti-α-tubulin (Sigma-Aldrich).

### qRT–PCR

Total RNA isolation from cells was performed using TRIzol (Sigma-Aldrich, T9424). Reverse transcription was performed with a High-Capacity RNA-to-cDNA Kit (Applied Biosystems, 4388950). qRT–PCR was performed on a Step-One Plus real-time system (Applied Biosystems) according to the manufacturer’s protocol. qPCRs were made on cDNAs obtained using Fast Start Universal SYBR Green (Roche, 491391401). Data were analyzed according to the Pfaffl method after calculation of primer efficiency. RPL0 or 36B4 was used as an endogenous control. All reactions were performed in triplicate, and at least three independent experiments were performed to generate each dataset.

### Bulk RNA-seq on cell lines

RNA from young and senescent cells was extracted using the TRI reagent (Sigma-Aldrich) protocol, with quality and concentration assessed via NanoDrop and Bioanalyzer (Agilent). Library construction, sequencing and initial data filtering, including adaptor removal, were conducted by BGI Genomics. RNA-seq was performed on the DNBSEQ platform in paired-end mode with a 150-bp read length. Low-quality reads, adaptor contamination and excessive unknown base (N) reads were filtered out using SOAPnuke. The reads were aligned to the GCF_000001405.39_GRCh38.p13 genome using HISAT2, followed by fusion gene and differential splicing detection using EricScript (0.5.5-5) and rMATS (v.4.1.1). Bowtie 2 was used for further alignment to the gene set, and quantification was done with RSEM (v.1.2.28). Differential gene expression analysis was performed using DESeq2. Downstream analysis, including DEGs and heatmap generation, was completed with BGI’s Dr. Tom software. Heatmaps were created using log-normalized transcripts per million (TPM) values. GSEA was performed using the fgsea package for three senescence-associated pathways (SAUL_SEN_MAYO, FRIDMAN_SENESCENCE_UP and REACTOME_SENESCENCE_ASSOCIATED_SECRETORY_PHENOTYPE_SASP) from the MSigDB database, considering pathways significantly enriched at an adjusted *P* value (*P*_adj_) lower than 0.05. All data are available in the Gene Expression Omnibus (GEO) under accession number GSE262856.

### GD3 dosage by ELISA

Serum from young (3 months old) or old (24 months old) mice or supernatant from young MRC5 cells until replicative senescent MRC5 cells were collected. The amount of free GD3 in sera or supernatants was assessed by ELISA against GD3 following the recommendations of the manufacturer (CUSABIO, CSB-EQ027866HU).

### Mouse co-culture experiment

Splenocytes were extracted from C57Bl/6j naive mice previously stimulated in vivo for 14 h by an intraperitoneal injection of 150 µg of poly I:C (Invivogen, tlrl-pic-5). The MRC5 cells or MEFs were plated at 5 × 10^4^ cells per well in a 96-well plate the day before the experiment for the good adherence of the cells. All co-cultures were performed with four different mice at each time. Then, the degranulation capacity of NK cells was tested when co-cultured with senescent/young or modulated for ST8SIA1 expression MRC5. NK cells were added to the culture for 4 h in presence of monensin and brefeldin (BD Biosciences, 554724 and 555029) at the effector:target ratio of 1:1. Degranulation activity of the NK cells was then measured by fluorescence-activated cell sorting (FACS) by anti-CD107a and IFN-γ staining.

### Human co-culture experiment

Human PBMCs were isolated from healthy donors with the standard Ficoll (Eurobio, CMSMSL01-0U) method, resuspended in RPMIc (RPMI 1640 (Gibco) + 1% penicilin–streptomycin + 10% FBS) and incubated overnight at 4 °C. Senescent and young MRC5 cells were plated at 5 × 10^4^ cells per well in a 96-well plate the day before the experiment for the good adherence of the cells. The next day, NK cells were isolated from pre-incubated PBMCs using a human NK cell untouched isolation (Miltenyi Biotec, 130-092-657) following the manufacturer’s instructions. Isolated NK cells were primed in vitro for 1 h with IL-15 at 100 ng ml^−1^ (PeproTech, 200-15). Then, stimulated NK cells were added to the co-culture for 4 h in presence of GolgiPlug (BD Biosciences, 555029) at the effector:target ratio of 1:5. Activities of the NK cells were measured by FACS by anti-CD107a and IFN-γ staining.

### Transwell migration assay

Human PBMCs were isolated from healthy donors with the standard Ficoll (Eurobio, CMSMSL01-0U) method, resuspended in RPMIc (RPMI 1640 (Gibco) + 1% penicilin–streptomycinn + 10% FBS) and incubated overnight at 4 °C. The next day, NK cells were isolated from pre-incubated PBMCs using a human NK cell untouched isolation (Miltenyi Biotec, 130-092-657) following the manufacturer’s instructions. NK cells were resuspended in MECGm at 5 × 10^6^ cells per milliliter. In transwell plates (insert membrane Transwell in transparent polycarbonate, Ø inserts 6.5 mm, porosity 3 µm; Corning, 003415), 600 μl of conditioned media from senescent or young cells or DMEM (Gibco) supplemented or not with 4-OHT to the bottom of the wells and 100 μl of cell suspension were added to the inserts in duplicate. Cells were migrated 3 h at 37 °C under 5% CO_2_. NK cells were stained and identified as CD56^+^CD3^−^CD14^−^CD19^−^.

### Ex vivo rechallenge experiment

Primary cells from the spleen (crushing on a 70-µm cell strainer) or from the lungs (dissociation with Miltenyi Lung Dissociation Kit, 130-905-927 and gentleMACS with Heaters) were extracted from PBS or bleomycin-instillated mice. Bulk of primary cells (either from the spleen or the lungs) was then rechallenged in vitro with YAC-1 cells for 4 h in presence of monensin and brefeldin (BD Biosciences, 554724 and 555029) at the effector:target ratio of 5:1. Degranulation activity of the NK cells was then measured by FACS by anti-CD107a and IFN-γ staining. Alternatively, after seeding, MRC5 cells were co-cultured for 18 h with poly I:C primed splenocytes before adding YAC-1 cancer cells to the culture for 4 h in presence of monensin and brefeldin (BD Biosciences, 554724 and 555029) at the effector:target ratio of 1:1. Degranulation activity of the NK cells was then measured by FACS by anti-CD107a and IFN-γ staining.

### Real-time NK cell cytotoxic assay

A real-time cytotoxic assay was performed as previously described^30,31^. In brief, target cells were labeled with 0.5 µM Calcein-AM (Molecular Probes, 11524277) for 15 min at room temperature. Proliferative or replicative senescent MRC5 cells were additionally treated with 100 µM indomethacin (Sigma-Aldrich, 405268) to block multidrug-resistant transporters that expulse calcein. The inhibitor was maintained in the medium during the assay. Human primary NK cells were purified from PBMCs from healthy donors after FACS. Calcein-labeled targets were incubated with human NK cells for 4 h at 37 °C, 5% CO_2_, and real-time monitoring of NK cell killing was performed on a BioTek Cytation 5 (Agilent). Cell images were processed using BioTek Gen5 software (Agilent). The percentage of lysis from triplicates was calculated as follows: % lysis = (1 − ((experimental well at *t*/experimental well at *t*_0_) / (control well at *t*/control well at *t*_0_))) × 100.

### Micro bulk RNA-seq on bleomycin-induced fibrotic lungs

Four 9-week-old male C57BL/6 mice (Charles River Laboratories) received intratracheal instillation of 50 µl of PBS or bleomycin (2.5 U kg^−1^; Sigma-Aldrich). After 28 d, the lungs were recovered and dissociated using Dispase (Corning), Collagenase A and DNAse I (Roche) digestion for 30 min at 37 °C. Cells were filtered, resuspended in DMEM with 10% FBS and incubated with 100 nM Bafilomycin A1 (Sigma-Aldrich) for 1 h at 37 °C, 5% CO_2_. Cells were then stained for SA-β-Gal using a Senescence Assay Kit (Abcam) and subsequently with AF647-coupled GD3 antibody for 30 min at 4 °C after Fc-Block anti-CD16/CD32 antibody saturation for 15 min on ice. DAPI was used as a viability marker for 10 min. Four cell populations (50,000 cells each: GD3^−^β-Gal^−^, GD3^+^β-Gal^−^, GD3^−^β-Gal^+^ and GD3^+^β-Gal^+^) were FACS-sorted into 350 µl of TCL lysis buffer (Qiagen) with 1% β-mercaptoethanol (Sigma-Aldrich) using a FACSAria III (BD Biosciences). Tubes were vortexed, snap frozen in liquid nitrogen and stored at −80 °C for RNA extraction. Total RNA was extracted with a Single Cell RNA Purification Kit (Norgen) and treated with RNAse-free DNAse (Qiagen). Quantitative and qualitative RNA analysis was done using an Ultra Sensitivity RNA Kit and Femto Pulse system (Agilent). Another DNAse treatment with a Heat&Run gDNA removal kit was done to avoid genomic contamination. DNA libraries were prepared from 3,000 pg of total RNA with the SMART-Seq Stranded Mammalian Single Cell Kit (Takara/Clontech). cDNA library quality was assessed using a 4200 TapeStation (Agilent) and a Qubit 4 Fluorometer (Thermo Fisher Scientific). Libraries were pooled, normalized to 1.5 nM, denatured and diluted to 300 pM. The pooled libraries were hybridized on a NovaSeq SP Flow cell and sequenced on the NovaSeq platform (Illumina), with a paired-end mode targeting 32 million reads per sample. Primary base call files were converted to FASTQ files with bcl2fastq. Alignment and normalization were done on the mouse genome (GRCm38) using STAR (v.2.7.3a), and gene expression was quantified with Salmon (v.1.4) using GENCODE vM25 annotations. Expression was normalized by TPM. RNA-seq quality control included total read count, alignment and duplication rate. One sample was excluded for not meeting quality standards. All analyses were done in R (v.4.3.0). DESeq2 (v.1.40.2) identified DEGs between β-Gal^+^GD3^+^ and β-Gal^−^GD3^+^ samples, considering genes with fold change (FC) ≥ 2 and Benjamini–Hochberg-adjusted *P* ≤ 0.001. Over-representation analysis (MsigDB hallmark collection) was done with clusterProfiler (v. 4.8.3), considering pathways with *P*_adj_ ≤ 0.05. Graphical representations used ggplot2 (v.3.4.2), and ComplexHeatmap (v.2.16.0) showed the top 25 significant DEGs. ssGSEA scores were calculated using a custom gene signature (Glb1, Cdkn2a, Cdkn1a, Trp53, Mki67 and Pcna). Tumor immune infiltration was inferred using the MCP-counter tool within the immunedeconv R package (v.2.1.0). A Wilcoxon rank-sum test was used to compare groups. *P* values are unadjusted due to the low sample number, with significance levels indicated: * *P* ≤ 0.05; ** *P* ≤ 0.01; *** *P* ≤ 0.001; **** *P* ≤ 0.0001. If no stars appear, no significant difference was found.

### Culture with metabolic inhibitors

MRC5 cells in replicative senescence were seeded in six-well plates at 300,000 cells per well. They were cultured with DMSO, a PGC-1α inhibitor (SR18292 at 10 μM; Sigma-Aldrich, SML2146) or an ERRα inhibitor (XCT790 at 5 μM; Sigma-Aldrich, X4753) for 72 h at 37 °C, 5% CO_2_. The cells were recovered and stained with directly AF647 coupled antibody directed against GD3 (BIOTEM) for 30 min at 4 °C after saturation with Fc-Block anti-CD16/CD32 antibodies (clone 2.4G2) for 15 min on ice and then analyzed using flow cytometry. The RNA was extracted using the TRI reagent protocol (Sigma-Aldrich, T9424).

### In vivo lung imaging by µCT

High-resolution CT scans were performed using a dedicated system (eXplore speCZT CT120, GE Healthcare). Mice were gas anesthetized (air and 1–2% isoflurane) in an air-warmed imaging chamber (Minerve) to maintain body temperature during the scanning time. µCT image acquisition consisted of 400 projections collected in one full rotation of the gantry in approximately 5 min in a single bed focused on the lungs, with a 450 mA/80 kV X-ray tube. Two-dimensional (2D) and three-dimensional (3D) images were obtained and analyzed using MicroView software (GE Healthcare).

### Bone parameter analyses

Hind leg knee was dissected to remove smooth tissues and scanned in a SkyScan 1176 µCT scanner (Bruker; 0.5 mm aluminum filter, 45 kV, 500 µA, 18-µm resolution, 0.5° rotation angle). Scans were reconstructed using CTAn v.1.9, Nrecon v.1.6 (Bruker) and a 3D model visualization software program (CTVol v.2.0). Misalignment compensation, ring artifacts and beam hardening were adjusted to obtain the correct re-construction of each paw. Bone degradation was quantified in subchondral bone and the epiphysis region of the medial and/or lateral plateau for each tibia (CTAn software; Bruker). Reconstructed 3D images of joints were obtained using Avizo software (Avizo Lite 9.3.0; FEI).

### Cartilage structure quantification by confocal laser scanning microscopy

Articular cartilage of the tibia medial plateau was scanned through its depth in *x*–*y*–*z* mode with a confocal laser scanning microscope (Leica, TCS SP5-II) with a voxel size of 6 μm, a 5× dry objective and a UV laser light source (l¼ 405 nm). Stacks of images were then done and analyzed to quantitatively evaluate several parameters of articular cartilage. Assessment of cartilage morphometric parameters was performed in the medial plateau of each tibia using Avizo software (FEI).

### SA-β-Gal staining and DNA damage analysis at telomeres (telomere-induced foci) by ImageStream^X^ analysis

Cells were resuspended (either cells in culture or primary cells after tissues digestion) in PBS and then fixed with 4% paraformaldehyde for 15 min at room temperature and stained using the Senescence-Associated β-Galactosidase Staining Kit (Abcam, ab65351) following the manufacturer’s instructions. After staining, cells were incubated for 12–14 h at 37 °C, sealed and protected from light. After washes, cells were analyzed by ImageStream^X^ or permeabilized with PBS/Triton X-100 (0.5%) and incubated 10 min at room temperature and then at 87 °C for 10 min while vortexing and overnight at room temperature protected from light with 70% formamide, 1% blocking reagent, 10 mM Tris pH 7.2, 4 nM PNA probe-FITC. Cells were stained with specific antibody for 53BP1 (1/300) (Novus Biological, NB100-305) and then incubated with goat anti-rabbit Alexa Fluor 647 (1/900) (Jackson ImmunoResearch, 111-607-003). Nuclei were stained with Hoechst (1/2,000), and then cells were washed twice and analyzed by ImageStream^X^.

### Histology and immunohistochemistry

For murine tissues, antigen retrieval was conducted on 5-μm paraffin sections using Vector unmasking reagent (Vector Laboratories, H3300). Sections were blocked (Avidin/Biotin block, MOM kit; Vector Laboratories, BMK-2202 and SP-2001) and incubated overnight at 4 °C with mouse monoclonal anti-GD3 (Abcam, ab11779; 1:40), anti-p16 (Abcam, ab54210; 1:100) or rabbit anti-p21 (Abcam, ab188224; 1:100). Primary antibody detection used a biotinylated anti-mouse IgG (MOM kit) or anti-rabbit IgG (Jackson ImmunoResearch, 111-065-144). For IF, tissues were incubated with streptavidin-Cy3 (Jackson ImmunoResearch, 016-160-084) for 1 h at room temperature. For immunohistochemistry (IHC), an ABC-HRP kit was used, and the DAB substrate (Vector Laboratories, PK-6100 and SK-4100) revealed staining.

For human tissues, microarrays were from Biomax (ref. LC561). Antigen retrieval was performed on 5-μm sections using Vector unmasking reagent. Sections were blocked with goat serum and incubated overnight at 4 °C with mouse monoclonal anti-GD3 (Abcam, ab11779; 1:40), anti-p16 (Abcam, ab54210; 1:100) or rabbit anti-p21 (Abcam, ab227443; 1:100). Detection used a biotinylated anti-mouse IgG (MOM kit) or anti-rabbit IgG (Jackson ImmunoResearch). An ABC-HRP kit and the DAB substrate (Vector Laboratories, PK-6100 and SK-4100) were used for staining.

For fibrosis analysis, slides were stained with picrosirius red solution for 1 h and washed with acetic acid solution and absolute alcohol before imaging in white light or polarized light.

Stained tissue sections were sequentially scanned using an HD Zeiss microscope allowing imaging of the entire section. For signal analysis, all glomeruli (about 120) were manually demarcated within each kidney section. Quantification of the signal within glomeruli or within the remaining area of the kidney was performed using ImageJ software. For signal analysis of lung fibrosis, 10 fields per slide were quantified for *n* = 8 mice per group using ImageJ software both for GD3 staining and for siruis red staining in polarized light. IHC quantifications were done using QuPath v.0.5.0.

### GTEx human gene expression analysis

The GTEx project is an ongoing effort to build a comprehensive public resource to study tissue-specific gene expression and regulation. Samples were collected from 54 non-diseased tissue sites across nearly 1,000 individuals. We used the raw open-access files from GTEx datasets to extract gene expression and various metainformations, such as age, sex and organ of origin, from the human donor cohort. We generated a home-made Python script (freely available upon reasonable request) that crossed GTEx RNA-seq data with related GTEx annotation files. The script is usable in Windows, Mac or Linux. The output is saved in a single Excel file containing the information for the selected gene. Data were then plotted and statistically analyzed using GraphPad Prism v. 8 and v.9.

### IPA

IPA (Qiagen, https://www.qiagen.com/ingenuity)^32^ was used to investigate how expression of ST8SIA1 regulators was altered in replicative-induced and oncogene-induced senescence. From the Ingenuity Pathway Knowledge Base (IPKB), links between RIP140 and ESRRA known ST8SIA1 upstream regulators, such as PPARGC1a and ESR2, were plotted and overlayed with observed (red/green) and IPA-predicted (orange/blue) modulation for those genes from RNA-seq data analysis for both senescence types. Only DEGs with false discovery rate (FDR) < 5% and │log_2_ FC│ > 2 were considered for this analysis.

### Statistics and reproducibility

No statistical method was used to predetermine sample size, but our sample sizes are similar to those reported in previous studies^3,17^. Mouse experiments were performed on *n* = 4–12 mice as indicated in the figure legends. No exclusion criteria were defined, and no data were excluded. The investigators were not blinded to allocation during experiments and outcome assessment. Statistical tests were all performed with GraphPad Prism 8 and 9 software, including the normality and lognormality tests, Student’s *t*-test, the Mann–Whitney test, the log-rank test, Pearson correlation and two-way ANOVA tests. Animal groups were normalized based on weight and clinical score in the cages before each experiment. No randomization method was used to allocate animals or samples to experimental groups.

### Reporting summary

Further information on research design is available in the Nature Portfolio Reporting Summary linked to this article.

## Supplementary information

Supplementary information Supplementary methods: antibodies and primers

Reporting Summary

Supplementary Tables 1–3 Table 1. DEGs between proliferating pdl30 MRC5 cells and replicative senescent MRC5 cells obtained by bulk RNA-seq corresponding to Extended Data Fig. 1. Table 2. DEGs between GD3^+^SA-β-Gal^−^ and GD3^+^SA-β-Gal^+^ obtained by microbulk RNA-seq corresponding to Fig. 6. Table 3. DEGs between proliferating pdl30 MRC5 cells and replicative senescent MRC5 cells obtained by bulk RNA-seq corresponding to the heatmap in Fig. 6.

## Source data

Source Data Fig. 1 Statistical Source Data

Source Data Fig. 2 Statistical Source Data

Source Data Fig. 2 Unprocessed images

Source Data Fig. 3 Statistical Source Data

Source Data Fig. 3 Unprocessed images

Source Data Fig. 3 Unprocessed images

Source Data Fig. 3 Unprocessed images

Source Data Fig. 3 Unprocessed images

Source Data Fig. 3 Unprocessed images

Source Data Fig. 4 Statistical Source Data

Source Data Fig. 5 Statistical Source Data

Source Data Fig. 5 Unprocessed images

Source Data Fig. 6 Statistical Source Data

Source Data Fig. 7 Statistical Source Data

Source Data Fig. 7 Unprocessed images

Source Data Extended Data Fig. 1 Statistical Source Data

Source Data Extended Data Fig. 1 Unprocessed images

Source Data Extended Data Fig. 2 Statistical Source Data

Source Data Extended Data Fig. 4 Statistical Source Data

Source Data Extended Data Fig. 4 Unprocessed images and western blots

Source Data Extended Data Fig. 5 Statistical Source Data

Source Data Extended Data Fig. 6 Statistical Source Data

Source Data Extended Data Fig. 7 Statistical Source Data

Source Data Extended Data Fig. 8 Statistical Source Data

Source Data Extended Data Fig. 9 Statistical Source Data

Source Data Extended Data Fig. 9 Unprocessed images

Source Data Extended Data Fig. 10 Statistical Source Data

Source Data Extended Data Fig. 10 Unprocessed images

## Data Availability

All data are available in the main text or the supplementary materials. For data coming from the GTEx project, a home-made Python script was created and is freely available upon reasonable request. All RNA-seq data are deposited in the Gene Expression Omnibus under accession numbers GSE262856 and GSE262926.
