# Supplementary material for: A ganglioside-based immune checkpoint enables senescent cells to evade immunosurveillance during aging
Source: Nat Aging. 2024 Dec 27;5(2):219–36. doi: 10.1038/s43587-024-00776-z (PMC11839482; doi:10.1038/s43587-024-00776-z)
Supplement: Supplementary file 1 — Supplementary methods: antibodies and primers [file 43587_2024_776_MOESM1_ESM.pdf]

nature aging

Supplementary information https://doi.org/10.1038/s43587-024-00776-z

A ganglioside-based immune checkpoint enables senescent cells to evade immunosurveillance during aging

In the format provided by the authors and unedited

Antibodies:

Specificity	Company	Clone	Species	Isotype	Fluorochrome	Reference
**anti Ly6G (Gr1)**	eBioscience	RB6-8C5	Rat	IgG2b, k	PE	12-5931-82
**anti CD107a**	BD Biosciences	1D4B	Rat	IgG2a/k	FITC	553793
**anti CD11b**	BD Biosciences	M1/70	Rat	IgG2b	APC-H7	550993
**anti CD11c**	BD Biosciences	HL3	Hamster	IgG1	FITC	557400
**anti CD19**	BD Biosciences	1D3	Rat	IgG2a, κ	FITC	553785
**anti NKp46**	BD Biosciences	29A1.4	Rat	IgG2a	PE	560757
**anti CD45**	BD Biosciences	30-F11	Rat	IgG2b	PerCP	557235
**anti CD45**	BD Biosciences	30-F11	Rat	IgG2b	A700	560510
**anti CD69**	BD Biosciences	H1.2F3	Hamster	IgG1/K	PE-Cy7	552879
**anti CD8a**	BD Biosciences	53-6.7	Rat	IgG2a,K	BV650	563152
**anti IFN-g**	BD Biosciences	4S.B3	Mouse	IgG1/K	PE	554552
**anti IFN-g**	BD Biosciences	XMG1.2	Rat	IgG1/K	PE	554412
**anti NK-1.1**	BD Biosciences	PK136	Mouse	IgG2a/k	APC	550627
**anti NK-1.1**	Biolegend	PK136	Mouse	IgG2a, κ	APC	108710
**anti NKp46**	BD Biosciences	29A1.4	Rat	IgG2a	Alexa 647	560755
**anti-CD107a**	BD Biosciences	1D4B	Rat SD	IgG2a, κ	V450	560648
**anti-CD11b**	Biolegend	M1/70	Rat	IgG2b, κ	BV605	101237
**anti-CD11c**	Biolegend	N418	Hamster	IgG	PE/dazzle	117347
**anti-CD19**	Biolegend	6D5	Rat	IgG2a, κ	BV510	115545
**anti-CD19**	Biolegend	6D5	Rat	IgG2a, κ	BV785	115543
**anti-CD25**	eBioscience	PC 61.5	Hamster	IgG1 l	PE-Cy7	25-0251-82
**anti-NKp46**	BD HORIZON	29A1.4	Rat	IgG2a, κ	BV510	563455
**anti-CD3e**	BD Biosciences	145-2C11	Hamster	IgG1, κ	FITC	553062
**anti-CD3e**	Biolegend	145-C11	Hamster	IgG	PerCP	100302
**anti-CD4**	BD Biosciences	GK1.5	Rat	IgG2b, κ	PE	553730
**anti-CD4**	LifeTech	monoclonal	Rat	IgG2a	PE-AF700	MCD0424
**anti-CD8a**	BD Biosciences	53-6.7	Rat LOU	IgG2a, κ	PerCP-Cy5.5	551162
**anti-F4/80**	Biolegend	BM8	Rat	IgG2a, κ	PerCP-Cy5.5	123128
**anti-F4/80**	Biolegend	BM8	Rat	IgG2a, κ	BV510	123135

**anti-Ly6C**	eBiosciences	HK1.4	Rat	IgG2c, κ	APC-eFluor780	47-5932-82
**anti-Gr-1**	Biolegend	RB6-8C5	Rat	IgG2b, κ	PE	108408
**anti-Gr-1**	BD Biosciences	RB6-8C5	Rat	IgG2b, κ	PE	553128
**anti-Gr-1**	Biolegend	RB6-8C5	Rat	IgG2b, κ	BV421	108434
**anti-Ly6G**	BD Biosciences	1A8	Rat LEW	IgG2a, κ	PE-Cy7	560601
**anti-Ly6G**	Biolegend	1A8	Rat	IgG2a, κ	BV421	127627
**anti-Ly6G**	BD Biosciences	1A8	Rat LW	IgG2a, κ	BV711	563979
**anti-GD3**	Abcam	R24	Mouse	IgG3	uncoupled	ab11779
**anti-GD3**	Biotem	R24 Hybridoma ATCC HB-8445	Mouse	IgG3	Uncoupled endotoxin free for *in vivo* experiment	HB-8445
**anti-53BP1**	Novus Biological	Rabbit polyclonal	Rabbit	IgG	uncoupled	NB100-305
**Recombinant Human Siglec-7 Fc Chimera Protein**	R&D Systems	Human recombinant			uncoupled	1138-SL-050
**anti-p16**	Abcam	2D9A12	Mouse	IgG2b	uncoupled	**ab54210**
**anti-p21**	Abcam	polyclonal	Rabbit	IgG	uncoupled	**ab227443**
**anti-p21**	Abcam	EPR18021	Rabbit	IgG	uncoupled	**ab188224**
**anti-IFNγ**	Biolegend	45B3	Mouse	IgG1	BV421	**502532**
**anti-CD3**	BD Biosciences	UCHT1	Mouse	IgG2a	BV711	**563725**
**anti-CD14**	Biolegend	M5E2	Mouse	IgG1	BV711	**301838**
**anti-CD19**	Biolegend	HIB19	Mouse	IgG1	BV711	**302246**
**anti-CD56**	Miltenyi Biotec	AF12-7H3	Mouse	IgG1	Vio Bright FITC	**130-113-309**
**anti-CD11c**	BD Biosciences	HL3	Hamster	IgG1	BUV395	564080
**anti-CD2**	BD Biosciences	RM2-5	Rat	IgG2b	BUV661	741467
**anti-Ly-6G**	Miltenyi Biotec	REA526	Recombinant human	IgG1	vio blue	130-119-902
**anti-CD11b**	Biolegend	M1/70	Rat	IgG2b	BV605	101257
**anti-CD19**	Biolegend	6D5	Rat	IgG2a	BV786	115543
**anti-CD107a**	Miltenyi Biotec	REA777	Recombinant human	IgG1	Vio B515	130-111-320
**anti-CD3**	Miltenyi Biotec	REA641	Recombinant human	IgG1	PerCP vio700	130-120-826
**anti-F4/80**	Miltenyi Biotec	REA126	Recombinant human	IgG1	PE-Vio615	130-123-913
**anti-CD69**	Miltenyi Biotec	REA937	Recombinant human	IgG1	PE-Vio770	130-115-577
**anti-NK1.1**	Biolegend	PK136	Mouse	IgG2a	APC	108710

**anti-CD45**	Biolegend	30-F11	Rat	IgG2b	AF700	103128
**anti-Ly-6C**	Miltenyi Biotec	REA796	Recombinant human	IgG1	APC Vio770	130-111-919

Primers:

Gene	Species	Forward	Reverse
**ST8SIA1**	*Homo sapiens*	GGGTGAGGCAAGTTGA AAGG	AGGTCCTCAGCGAAT TTCCA
**36B4**	*Homo sapiens*	ACTCGTTTGTACCCGTT GATG	AACTCTGCATTCTCG CTTCCT
**GLB1**	*Homo sapiens*	AGCTACGACTATGATGC CCC	ACAGAATGTCCAGAG CTGCT
**NRIP1**	*Homo sapiens*	TCTACGCAAGGAGGAG GAGA	TTGGAGACAGACGAA CACTGA
**PPAARGC1A**	*Homo sapiens*	AGCCTCTTTGCCCAGAT CTT	GGCAATCCGTCTTCA TCCAC
**ESRRA**	*Homo sapiens*	GGCATTGAGCCTCTCT ACATCA	TCTCCGAGGAACCCT TTGG
**CDKN1A**	*Homo sapiens*	AGGTGGACCTGGAGAC TCTCAG	TCCTCTTGGAGAAGA TCAGCCG
**CDKN2A**	*Homo sapiens*	GCCCAACGCACCGAAT AGTT	GCAGGTACCGTGCGA CATC
**36b4**	*Mus musculus*	AGATTCGGGATATGCT GTTGGC	TCGGGTCCTAGACCA GTGTTC
**St8sia1**	*Mus musculus*	TGGGAAACGGTGGGAT TCTG	TGGCGAATTATGCTG GGGTT
**Cdkn1a**	*Mus musculus*	AATTGGAGTCAGGCGC AGAT	CATGAGCGCATCGCA AT
**Cdkn2a**	*Mus musculus*	GCCCAACGCCCCGAAC TCTTTC	GCGACGTTCCCAGCG GTACACA
**Glb1**	*Mus musculus*	GGATGGACAGCCATTC CGAT	CACGGTCCCCAGAAA ACTCA
**Tnf**	*Mus musculus*	ACGTCGTAGCAAACCA CC	CCCTTGAAGAGAACC TGGGAG
**Il6**	*Mus musculus*	CTGGAGTACCATAGCTA CCTGG	GAGAGCATTGGAAAT TGGGGT
**Tgfa**	*Mus musculus*	GCTCTGCTAGCGCTGG GTAT	CTGCATGCTCACAGC GAAC
**Tgfb1**	*Mus musculus*	CAGACATTCGGGAAGC AGTG	AAAGCCCTGTATTCC GTCTCC
**Col1a1**	*Mus musculus*	Mm00801666_g1 (Thermofisher)	Mm00801666_g1 (Thermofisher)
