# Supplementary material for: A ganglioside-based immune checkpoint enables senescent cells to evade immunosurveillance during aging
Source: Nat Aging. 2024 Dec 27;5(2):219–36. doi: 10.1038/s43587-024-00776-z (PMC11839482; doi:10.1038/s43587-024-00776-z)
Supplement: Supplementary file 2 — Reporting Summary [file 43587_2024_776_MOESM2_ESM.pdf]

nature portfolio

Corresponding author(s): Julien Cherfils-Vicini

Last updated by author(s): Oct 22, 2024

Reporting Summary

Nature Portfolio wishes to improve the reproducibility of the work that we publish. This form provides structure for consistency and transparency in reporting. For further information on Nature Portfolio policies, see our Editorial Policies and the Editorial Policy Checklist.

Statistics

For all statistical analyses, confirm that the following items are present in the figure legend, table legend, main text, or Methods section.

n/a	Confirmed	
☐	☒	The exact sample size (*n*) for each experimental group/condition, given as a discrete number and unit of measurement
☐	☒	A statement on whether measurements were taken from distinct samples or whether the same sample was measured repeatedly
☐	☒	The statistical test(s) used AND whether they are one- or two-sided<br>*Only common tests should be described solely by name; describe more complex techniques in the Methods section.*
☐	☒	A description of all covariates tested
☐	☒	A description of any assumptions or corrections, such as tests of normality and adjustment for multiple comparisons
☐	☒	A full description of the statistical parameters including central tendency (e.g. means) or other basic estimates (e.g. regression coefficient) AND variation (e.g. standard deviation) or associated estimates of uncertainty (e.g. confidence intervals)
☐	☒	For null hypothesis testing, the test statistic (e.g. *F*, *t*, *r*) with confidence intervals, effect sizes, degrees of freedom and *P* value noted<br>*Give P values as exact values whenever suitable.*
☒	☐	For Bayesian analysis, information on the choice of priors and Markov chain Monte Carlo settings
☒	☐	For hierarchical and complex designs, identification of the appropriate level for tests and full reporting of outcomes
☐	☒	Estimates of effect sizes (e.g. Cohen's *d*, Pearson's *r*), indicating how they were calculated

Our web collection on statistics for biologists contains articles on many of the points above.

Software and code

Policy information about availability of computer code

Data collection	FACS DIVA version 6 (Cytometry)<br>CyteExpert version 2.3 (Cytometry)<br>StepOne™ (RT-qPCR)<br>MicroView (GE Healthcare) (Micro-CT image acquisition)<br>Inspire (ImagestreamX acquisition)<br>ZEN (Immunofluorescence acquisition)
Data analysis	FlowJO 10 (Cytometry)<br>CyteExpert (Cytometry)<br>GEN5 software (Biotek) (real time NK cell killing assay<br>MicroView (GE Healthcare) (Micro-CT image analysis)<br>Ideas 6.2 (ImagestreamX analysis)<br>ZEN (Immunofluorescence image analysis)<br>FIJI 2.16.0 (Image analysis)<br>QuPath v0.5.1<br>Ingenuity Pathway Analysis<br>Prism GraphPad version 8 and 9

For manuscripts utilizing custom algorithms or software that are central to the research but not yet described in published literature, software must be made available to editors and reviewers. We strongly encourage code deposition in a community repository (e.g. GitHub). See the Nature Portfolio guidelines for submitting code & software for further information.

Data

Policy information about availability of data

All manuscripts must include a data availability statement. This statement should provide the following information, where applicable:

- Accession codes, unique identifiers, or web links for publicly available datasets
- A description of any restrictions on data availability
- For clinical datasets or third party data, please ensure that the statement adheres to our policy

Data and materials availability: All data are available in the main text or the supplementary materials. For data coming from the Genotype-Tissue Expression (GTEx) Project, home-made Python script has been created and is freely available on demand.

Field-specific reporting

Please select the one below that is the best fit for your research. If you are not sure, read the appropriate sections before making your selection.

☒ Life sciences ☐ Behavioural & social sciences ☐ Ecological, evolutionary & environmental sciences

For a reference copy of the document with all sections, see nature.com/documents/nr-reporting-summary-flat.pdf

Life sciences study design

All studies must disclose on these points even when the disclosure is negative.

Sample size	Reasonable sample size was chosen to ensure adequate reproducibility of results and was based on our previous studies. Mouse experiments are performed on n = 8 to 12 individuals as indicated in Fig legends after validation using a Monte Carlo power test before experiments. The distribution has been determined by ANOVA test each time.
Data exclusions	No exclusion criteria were defined, and no data were excluded
Replication	In vivo and In vitro experiments are repeated independently as indicated in each figure legends. All experimental findings were reproducible. No experiment was found to be irreproducible.
Randomization	Mice were equally divided for treatments and controls. a formal randomization tool was not employed. For survival analysis, mouse groups were constituted to be identical before treatment
Blinding	We were not blinded to group allocation during data collection and analysis. Many of our measurements were objective and quantitative, reducing the risk of bias. We ensured objectivity through quantitative measurements and predefined protocols, minimizing potential biases and maintaining the study's integrity.

Reporting for specific materials, systems and methods

We require information from authors about some types of materials, experimental systems and methods used in many studies. Here, indicate whether each material, system or method listed is relevant to your study. If you are not sure if a list item applies to your research, read the appropriate section before selecting a response.

Materials & experimental systems

n/a	Involved in the study	
☐	☒	Antibodies
☐	☒	Eukaryotic cell lines
☒	☐	Palaeontology and archaeology
☐	☒	Animals and other organisms
☒	☐	Human research participants
☒	☐	Clinical data
☒	☐	Dual use research of concern

Methods

n/a	Involved in the study	
☒	☐	ChIP-seq
☐	☒	Flow cytometry
☒	☐	MRI-based neuroimaging

Antibodies

Antibodies used

Specificity Company Clone Species Isotype Fluorochrome Reference
anti Ly6G (Gr1) eBioscience RB6-8C5 Rat IgG2b, k PE 12-5931-82
anti CD107a BD Biosciences 1D4B Rat IgG2a/k FITC 553793
anti CD11b BD Biosciences M1/70 Rat IgG2b APC-H7 550993
anti CD11c BD Biosciences HL3 Hamster IgG1 FITC 557400
anti CD19 BD Biosciences 1D3 Rat IgG2a, κ FITC 553785

anti NKp46 BD Biosciences 29A1.4 Rat IgG2a PE 560757
anti CD45 BD Biosciences 30-F11 Rat IgG2b PerCP 557235
anti CD45 BD Biosciences 30-F11 Rat IgG2b A700 560510
anti CD69 BD Biosciences H1.2F3 Hamster IgG1/K PE-Cy7 552879
anti CD8a BD Biosciences 53-6.7 Rat IgG2a,K BV650 563152
anti IFN-g BD Biosciences 4S.B3 Mouse IgG1/K PE 554552
anti IFN-g BD Biosciences XMG1.2 Rat IgG1/K PE 554412
anti NK-1.1 BD Biosciences PK136 Mouse IgG2a/k APC 550627
anti NK-1.1 Biolegend PK136 Mouse IgG2a, κ APC 108710
anti NKp46 BD Biosciences 29A1.4 Rat IgG2a Alexa 647 560755
anti-CD107a BD Biosciences 1D4B Rat SD IgG2a, κ V450 560648
anti-CD11b Biolegend M1/70 Rat IgG2b, κ BV605 101237
anti-CD11c Biolegend N418 Hamster IgG PE/dazzle 117347
anti-CD19 Biolegend 6D5 Rat IgG2a, κ BV510 115545
anti-CD19 Biolegend 6D5 Rat IgG2a, κ BV785 115543
anti-CD25 eBioscience PC 61.5 Hamster IgG1 l PE-Cy7 25-0251-82
anti-NKp46 BD HORIZON 29A1.4 Rat IgG2a, κ BV510 563455
anti-CD3e BD Biosciences 145-2C11 Hamster IgG1, κ FITC 553062
anti-CD3e Biolegend 145-C11 Hamster IgG PerCP 100302
anti-CD4 BD Biosciences GK1.5 Rat IgG2b, κ PE 553730
anti-CD4 LifeTech monoclonal Rat IgG2a PE-AF700 MCD0424
anti-CD8a BD Biosciences 53-6.7 Rat LOU IgG2a, κ PerCP-Cy5.5 551162
anti-F4/80 Biolegend BM8 Rat IgG2a, κ PerCP-Cy5.5 123128
anti-F4/80 Biolegend BM8 Rat IgG2a, κ BV510 123135
anti-Ly6C eBiosciences HK1.4 Rat IgG2c, κ APC-eFluor780 47-5932-82
anti-Gr-1 Biolegend RB6-8C5 Rat IgG2b, κ PE 108408
anti-Gr-1 BD Biosciences RB6-8C5 Rat IgG2b, κ PE 553128
anti-Gr-1 Biolegend RB6-8C5 Rat IgG2b, κ BV421 108434
anti-Ly6G BD Biosciences 1A8 Rat LEW IgG2a, κ PE-Cy7 560601
anti-Ly6G Biolegend 1A8 Rat IgG2a, κ BV421 127627
anti-Ly6G BD Biosciences 1A8 Rat LW IgG2a, κ BV711 563979
anti-GD3 Abcam R24 Mouse IgG3 uncoupled ab11779
anti-GD3 Biotem R24 Hybridoma ATCC HB-8445 Mouse IgG3 Uncoupled endotoxin free for in vivo experiment Hybridoma ATCC HB-8445
anti-53BP1 Novus Biological Rabbit polyclonal Rabbit IgG uncoupled NB100-305
Recombinant Human Siglec-7 Fc Chimera Protein R&D Systems Human recombinant uncoupled 1138-SL-050
anti-p16 Abcam 2D9A12 Mouse IgG2b uncoupled ab54210
anti-p21 Abcam polyclonal Rabbit IgG uncoupled ab227443
anti-p21 Abcam EPR18021 Rabbit IgG uncoupled ab188224
anti-IFNg Biolegend 45B3 Mouse IgG1 BV421 502532
anti-CD3 BD Biosciences UCHT1 Mouse IgG2a BV711 563725
anti-CD14 Biolegend M5E2 Mouse IgG1 BV711 301838
anti-CD19 Biolegend HIB19 Mouse IgG1 BV711 302246
anti-CD56 Miltenyi Biotec AF12-7H3 Mouse IgG1 Vio Bright FITC 130-113-309
anti-CD11c BD Biosciences HL3 Hamster IgG1 BUV395 564080
anti-CD2 BD Biosciences RM2-5 Rat IgG2b BUV661 741467
anti-Ly-6G Miltenyi Biotec REA526 Recombinant human IgG1 vio blue 130-119-902
anti-CD11b Biolegend M1/70 Rat IgG2b BV605 101257
anti-CD19 Biolegend 6D5 Rat IgG2a BV786 115543
anti-CD107a Miltenyi Biotec REA777 Recombinant human IgG1 Vio B515 130-111-320
anti-CD3 Miltenyi Biotec REA641 Recombinant human IgG1 PerCP vio700 130-120-826
anti-F4/80 Miltenyi Biotec Recombinant human IgG1 PE-Vio615 130-123-913 REA126
anti-CD69 Miltenyi Biotec Recombinant human IgG1 PE-Vio770 130-115-577 REA937
anti-NK1.1 Biolegend PK136 Mouse IgG2a APC 108710
anti-CD45 Biolegend 30-F11 Rat IgG2b AF700 103128
anti-Ly-6C Miltenyi Biotec Recombinant human IgG1 APC Vio770 130-111-919 REA796

Validation

All the antibodies used in this study are commercial and are validated by the manufacturer

Eukaryotic cell lines

Policy information about cell lines

Cell line source(s)

MRC5 (obtained from the ATCC, Manassas, VA, ref #CCL-171)
WI-38 (obtained from the ATCC, Manassas, VA, ref #CCL-75)
normal human primary Mammary Epithelial Cells (hMEC; obtained from the ATCC, Manassas, VA, ref #PCS-600-010)
YAC-1(obtained from the ATCC, Manassas, VA, ref #TIB-160

Authentication	None of the cell line used were authenticated
Mycoplasma contamination	Mycoplasma test were performed every 3 months by PCR and experiments are performed only on mycoplasma negative cells.
Commonly misidentified lines (See ICLAC register)	none

Animals and other organisms

Policy information about studies involving animals; ARRIVE guidelines recommended for reporting animal research

Laboratory animals	Experiments are performed on 8- to 10-week-old female NMRI Nude mice from Charles River (France); of 3 months old BALB/c mice (Charles River); 3 motnh-old or 18 months old C57Bl6jRj (Jax lab) or internal cross (IRCAN mouse house facility) for TERT KO mice. Animals were maintained in a 12 ∶ 12-h light-dark cycle with food and water ad libitum with a controlled temperature (20-24°C) and hygrometry (50-70%).
Wild animals	none
Field-collected samples	none
Ethics oversight	All mouse experiments were conducted according to local and international institutional guidelines and were approved by either the Animal Care Committee of the IRCAN and the regional (CIEPAL Côte d'Azur Agreements NCE/2015-266#2015102215087555 and NCE/2020-675# 2020042723583497) and national (French Ministry of Research) authorities.

Note that full information on the approval of the study protocol must also be provided in the manuscript.

Flow Cytometry

Plots

Confirm that:

☒ The axis labels state the marker and fluorochrome used (e.g. CD4-FITC).

☒ The axis scales are clearly visible. Include numbers along axes only for bottom left plot of group (a 'group' is an analysis of identical markers).

☒ All plots are contour plots with outliers or pseudocolor plots.

☒ A numerical value for number of cells or percentage (with statistics) is provided.

Methodology

Sample preparation	For matrigel plug assay, infiltrating cells were collected after enzymatic dissociation by Dispase (Corning), collagenase A, and DNAse I (Roche) digestion for 30 min at 37°C. Infiltrating cells stained with directly coupled antibodies for 30 min at 4°C after saturation with Fc-Block anti-CD16/CD32 antibodies (clone 2.4G2) for 15 min on ice. After washes in 0.5 mM EDTA 2% FCS PBS, cells were analyzed using an ARIA III cytometer BD Biosciences with DIVA6 software and FlowJo 10 software.<br>For mouse model of lung fibrosis, lungs were freshly dissociated with Miltenyi Lung dissociation Kit (ref 130-905-927) and GentleMacs with Heaters. Infiltrating cells stained with directly coupled antibodies for 30 min at 4°C after saturation with Fc-Block anti-CD16/CD32 antibodies (clone 2.4G2) for 15 min on ice. After washes in 0.5 mM EDTA 2% FCS PBS, cells were analyzed using an ARIA III cytometer BD Biosciences with DIVA6 software and FlowJo 10 software.<br>For FACS analysis on cell in vitro, cell were washed and trypsinizated. Then, For the GD3 staining, we use a primary antibody anti-GD3 R24 (Abcam) at 1:1000, overnight at 4°C. A secondary antibody against mouse whole IgG in FITC is used at 1:3000 during 1 hour at room temperature (Jackson ImmunoResearch).<br>For NK cell degranulation assay, NK cells are added to the culture for 4 h in presence of monensin and brefeldin (BD Biosciences) at the effector/target ratio of 1:1. Degranulation activity of the NK cells is then measured by FACS by the anti-CD107a (FITC BD Biosciences) and IFN-g staining (PE BD Biosciences).
Instrument	All the experiment are acquired using ARIA III cytometer BD Biosciences with DIVA6 software and FlowJo 10 software or Cytoflex LX 6 lasers. For Flow imaging technology, an ImageStreamX mark II from Luminex was used.
Software	BD DIVA 6<br>FlowJO 10<br>CyteExpert 2.3<br>IDEAS 6.2
Cell population abundance	No sorting experiment were used

Gating strategy

All cytometry analysis are performed after FSC-A/SSC-A plot, then doublet cell exclusions (FSC-H/FSC-W and SSC-H/SSC-W plots) and CD45/SSC plot to select CD45+ cells. Then, the specific immune cells are alamyzed base on CD3+ CD4+ or CD3+ CD8+ cells for T cell analysys; CD11b+ GR1+ for MDSC; NKp46+ CD3- for NK cells. Then, the specific analysis to each sub population are described in main figures.

☒ Tick this box to confirm that a figure exemplifying the gating strategy is provided in the Supplementary Information.
