# Supplementary figures and images for: A ganglioside-based immune checkpoint enables senescent cells to evade immunosurveillance during aging (part 1 of 2)
Source: Nat Aging. 2024 Dec 27;5(2):219–36. doi: 10.1038/s43587-024-00776-z (PMC11839482; doi:10.1038/s43587-024-00776-z)

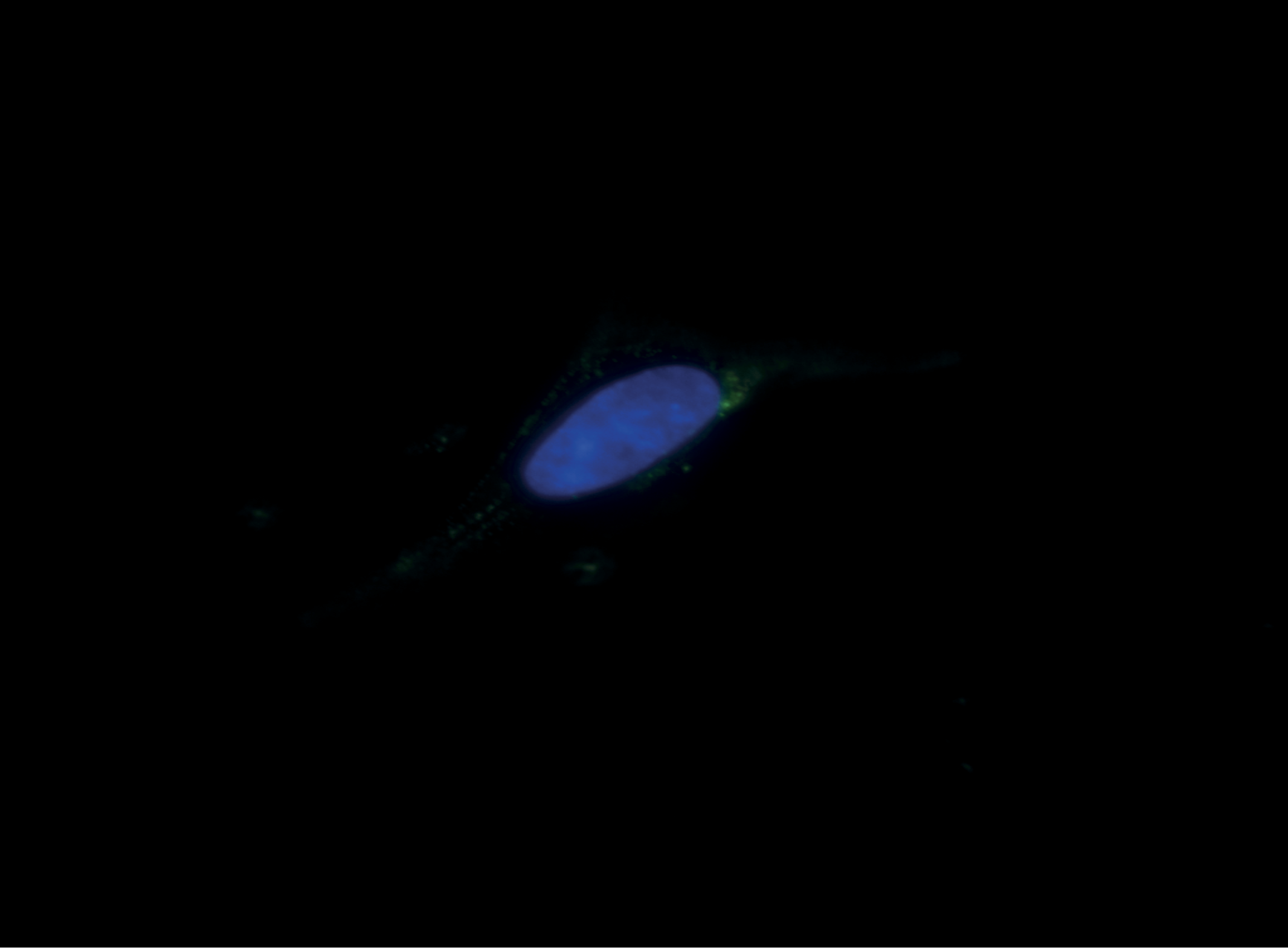

Supplement: Supplementary file 6 — Unprocessed images [file 43587_2024_776_MOESM6_ESM.zip › SD_Figure_2_images/Fig_2_C_pdl30.png]

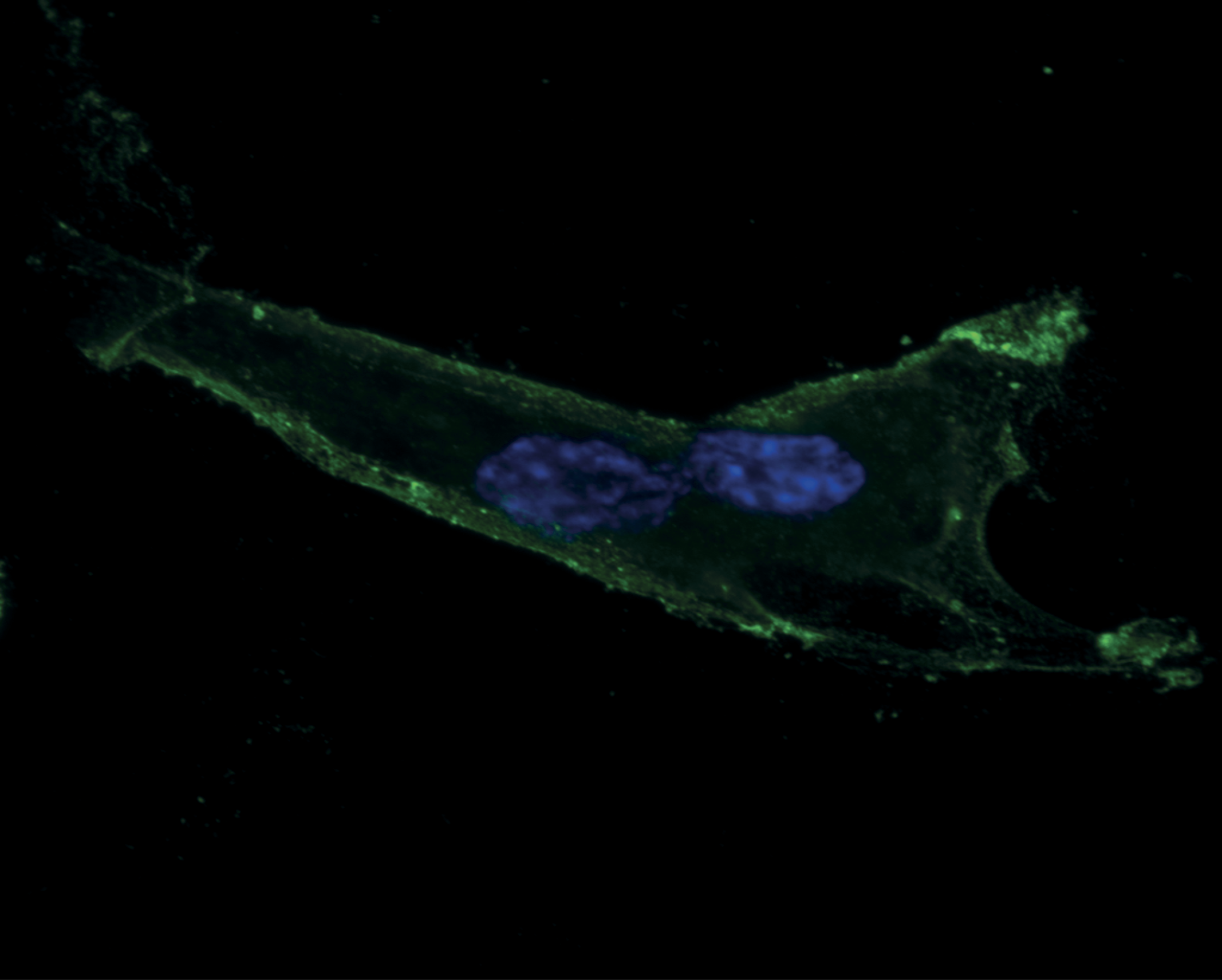

Supplement: Supplementary file 6 — Unprocessed images [file 43587_2024_776_MOESM6_ESM.zip › SD_Figure_2_images/Fig_2_C_Rep_sen.png]

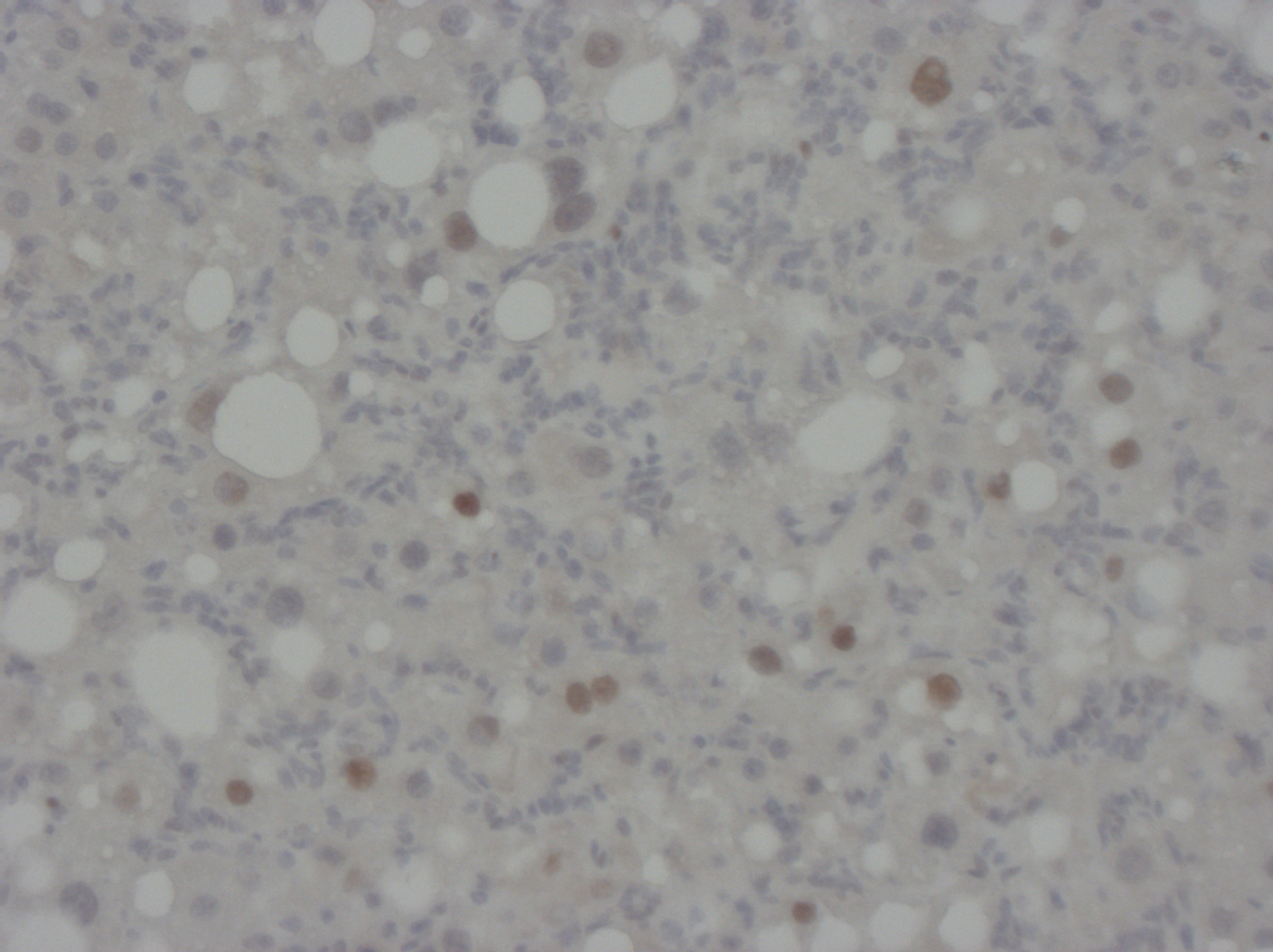

Supplement: Supplementary file 15 — Unprocessed images [file 43587_2024_776_MOESM15_ESM.zip › SD_Figure_5_images/ Fig_5_H_FOIE 1 WD 1 p21-03-Image Export-113.tif]

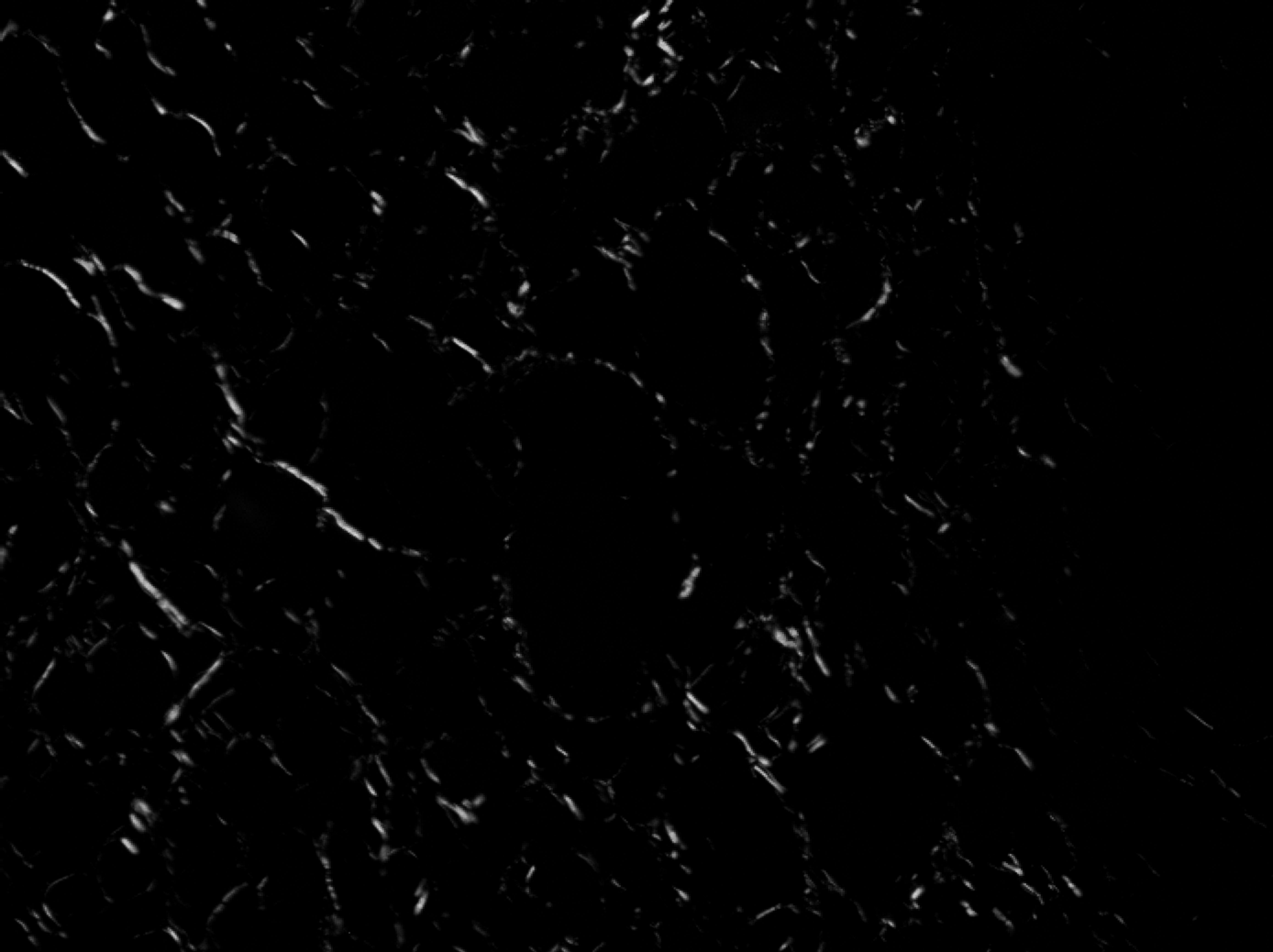

Supplement: Supplementary file 15 — Unprocessed images [file 43587_2024_776_MOESM15_ESM.zip › SD_Figure_5_images/ Fig_5_H_FOIE 5 WD1-06 PL.tif]

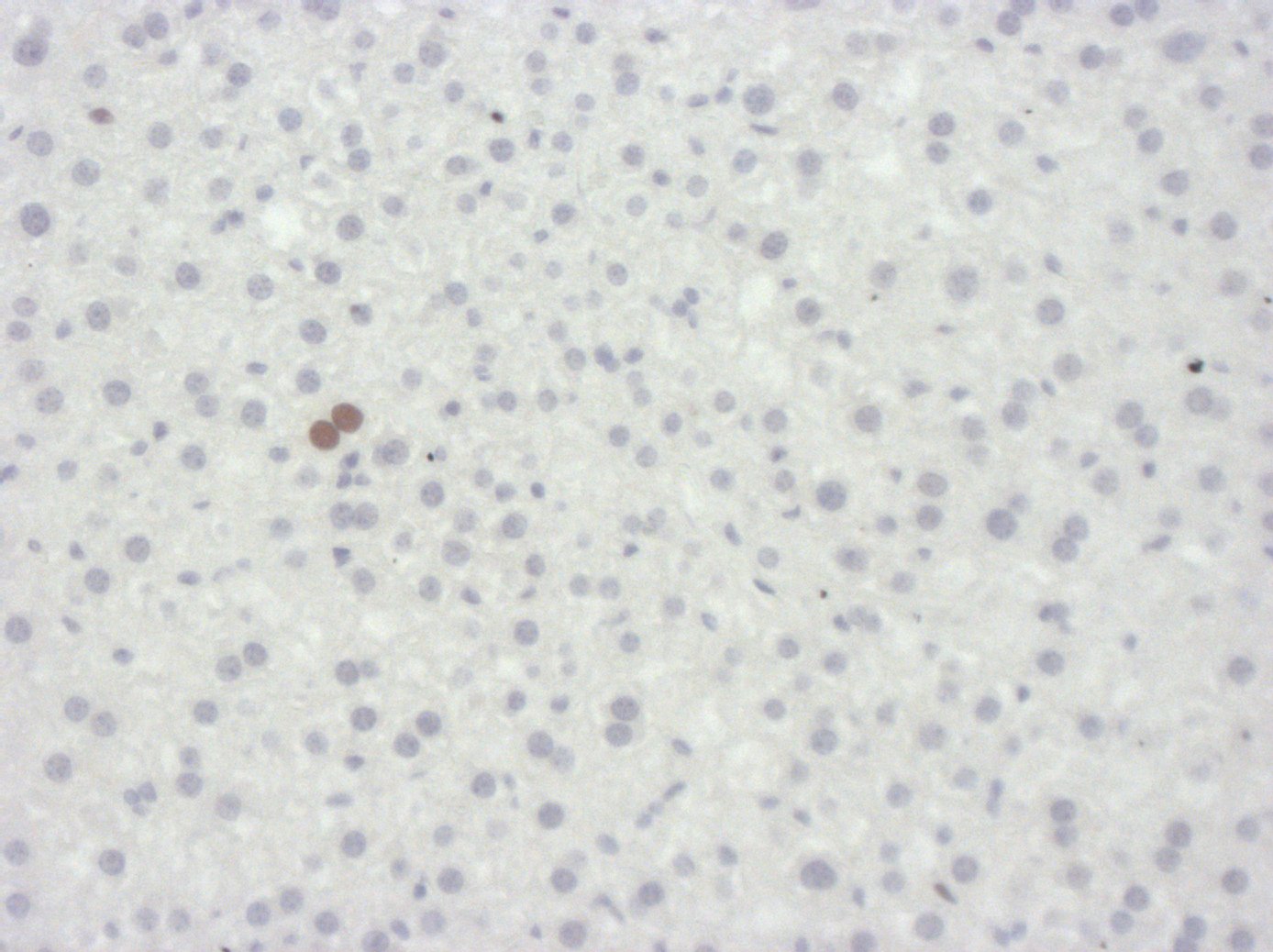

Supplement: Supplementary file 15 — Unprocessed images [file 43587_2024_776_MOESM15_ESM.zip › SD_Figure_5_images/ Fig_5_H_FOIE 12 CD2 p21-02-Image Export-362.jpg]

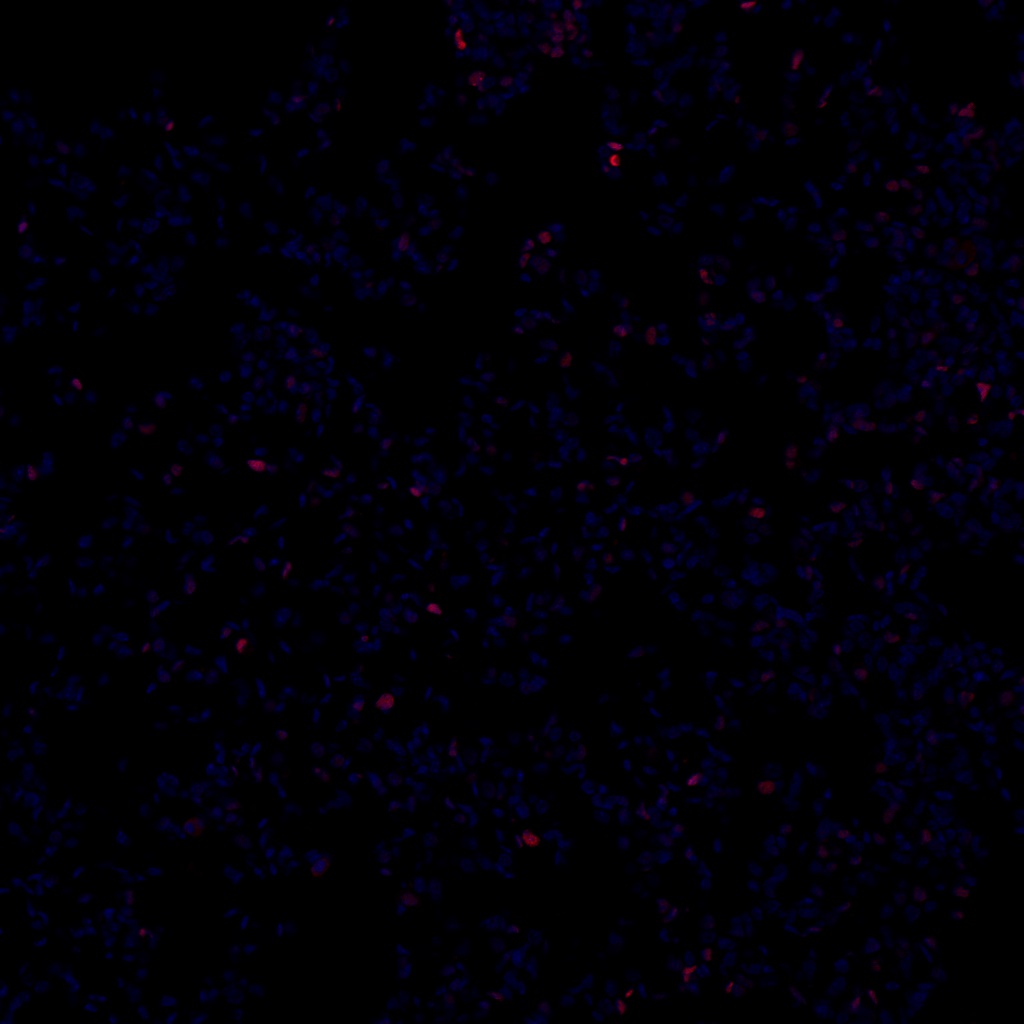

Supplement: Supplementary file 15 — Unprocessed images [file 43587_2024_776_MOESM15_ESM.zip › SD_Figure_5_images/Fig_5_F_bleo day 120 gd3 3-Image Export-04_c1+2.jpg]

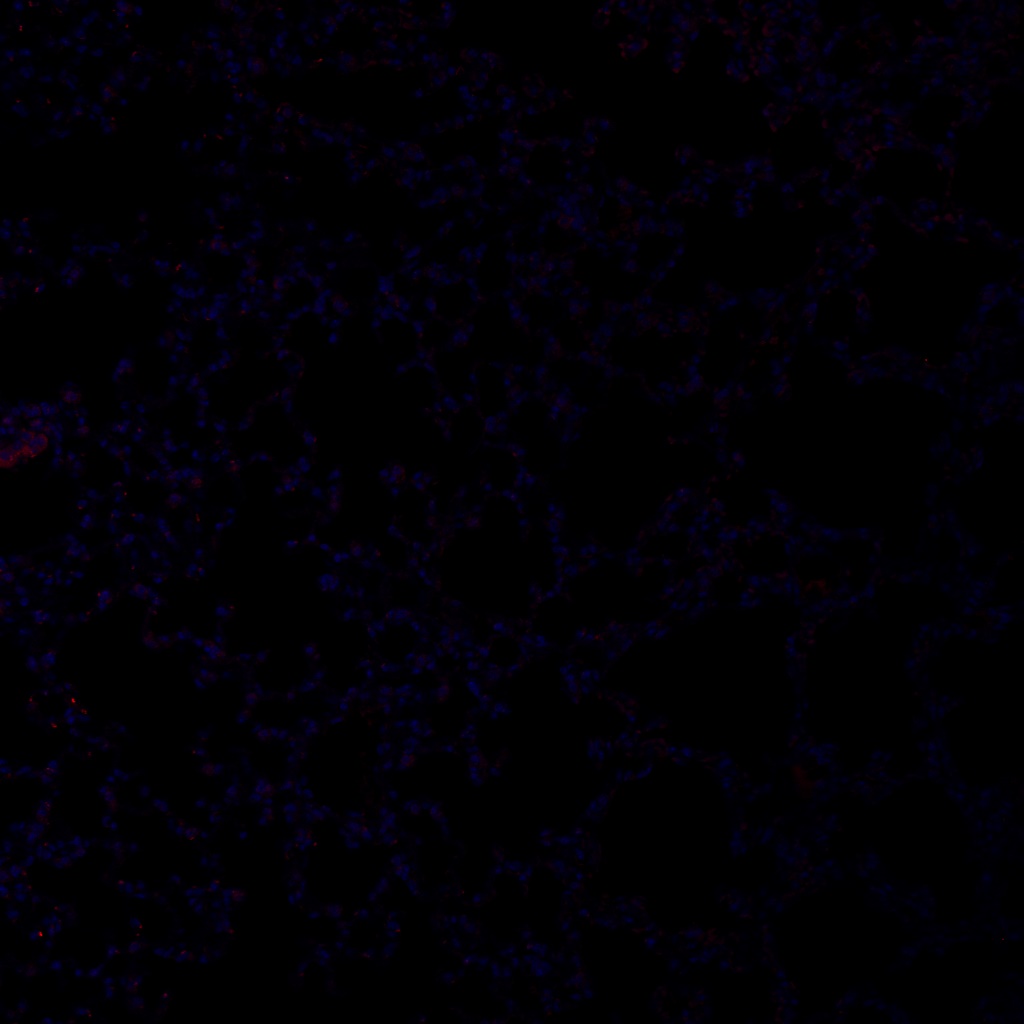

Supplement: Supplementary file 15 — Unprocessed images [file 43587_2024_776_MOESM15_ESM.zip › SD_Figure_5_images/Fig_5_F_wt 2 gd3 2-Image Export-29_c1+2.jpg]

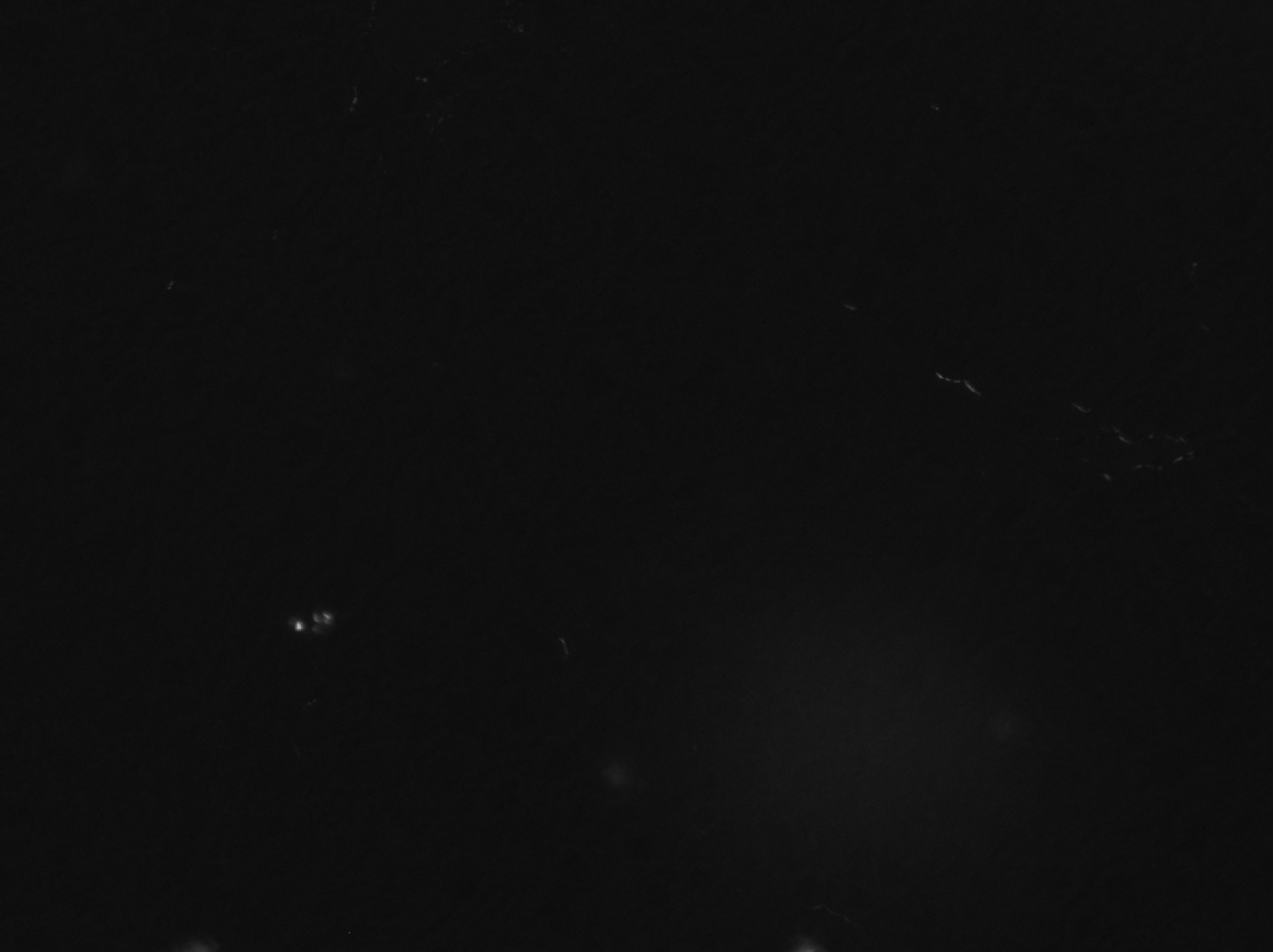

Supplement: Supplementary file 15 — Unprocessed images [file 43587_2024_776_MOESM15_ESM.zip › SD_Figure_5_images/ Fig_5_H_FOIE 18 CD1-09 PL.tif]

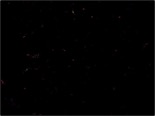

Supplement: Supplementary file 15 — Unprocessed images [file 43587_2024_776_MOESM15_ESM.zip › SD_Figure_5_images/Fig_5_F_POL 120 day.jpg]

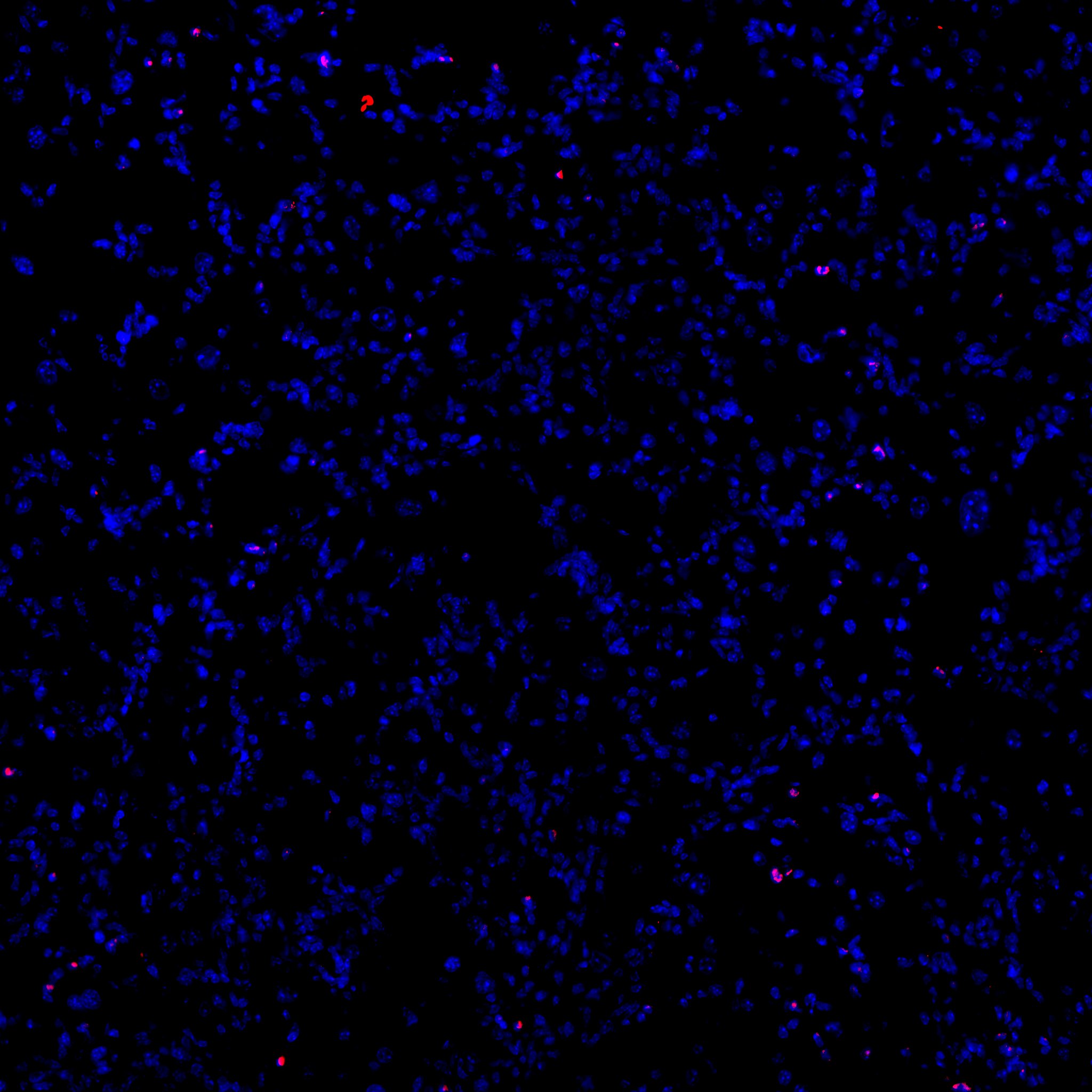

Supplement: Supplementary file 15 — Unprocessed images [file 43587_2024_776_MOESM15_ESM.zip › SD_Figure_5_images/ Fig_5_H_FOIE 7 WD 1 GD3-08.jpg]

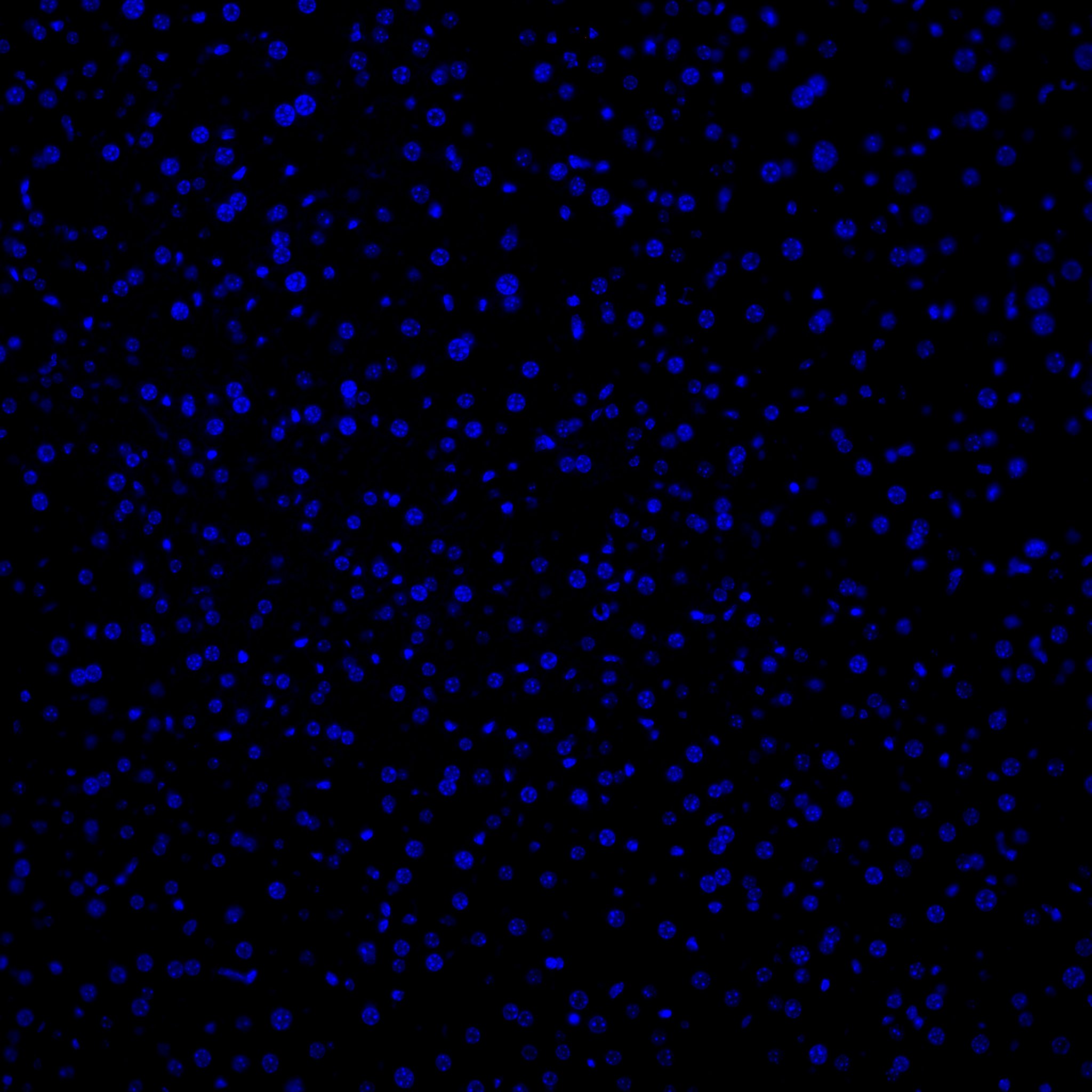

Supplement: Supplementary file 15 — Unprocessed images [file 43587_2024_776_MOESM15_ESM.zip › SD_Figure_5_images/ Fig_5_H_FOIE 12 CD 2 GD3-07.jpg]

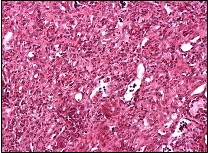

Supplement: Supplementary file 15 — Unprocessed images [file 43587_2024_776_MOESM15_ESM.zip › SD_Figure_5_images/Fig_5_F_HE 21 day.jpg]

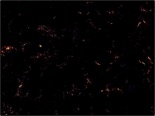

Supplement: Supplementary file 15 — Unprocessed images [file 43587_2024_776_MOESM15_ESM.zip › SD_Figure_5_images/Fig_5_F_POL 21 day.jpg]

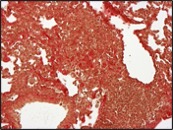

Supplement: Supplementary file 15 — Unprocessed images [file 43587_2024_776_MOESM15_ESM.zip › SD_Figure_5_images/Fig_5_A_SR Bloe.jpg]

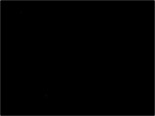

Supplement: Supplementary file 15 — Unprocessed images [file 43587_2024_776_MOESM15_ESM.zip › SD_Figure_5_images/Fig_5_F_POL 0 day.jpg]

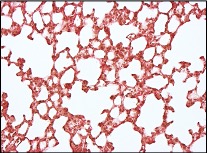

Supplement: Supplementary file 15 — Unprocessed images [file 43587_2024_776_MOESM15_ESM.zip › SD_Figure_5_images/Fig_5_F_SR 21 day.jpg]

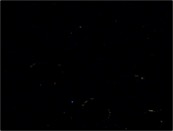

Supplement: Supplementary file 15 — Unprocessed images [file 43587_2024_776_MOESM15_ESM.zip › SD_Figure_5_images/Fig_5_A_POL PBS.jpg]

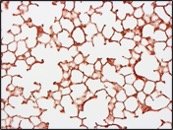

Supplement: Supplementary file 15 — Unprocessed images [file 43587_2024_776_MOESM15_ESM.zip › SD_Figure_5_images/Fig_5_A_SR PBS.jpg]

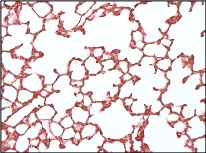

Supplement: Supplementary file 15 — Unprocessed images [file 43587_2024_776_MOESM15_ESM.zip › SD_Figure_5_images/Fig_5_F_SR 0 day.jpg]

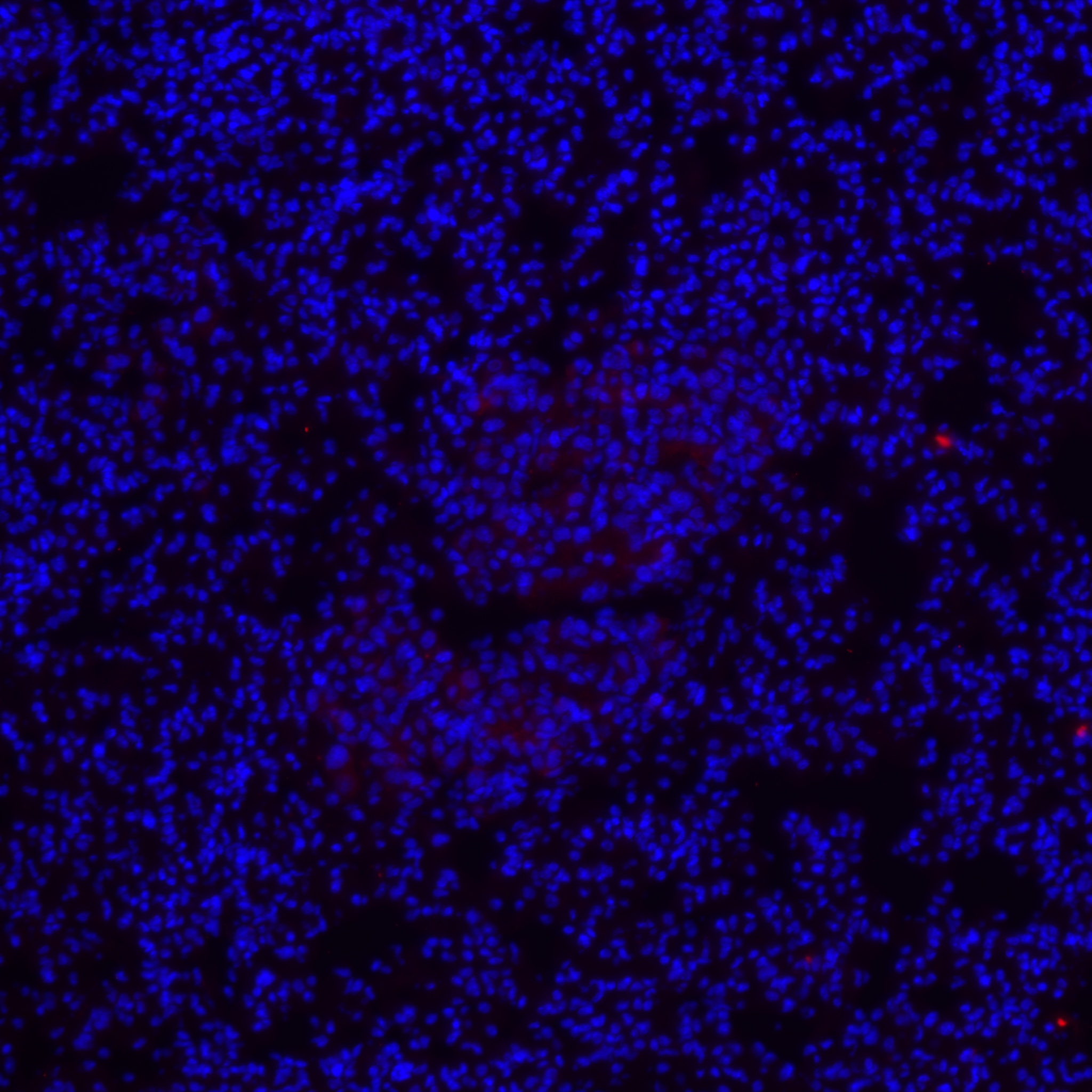

Supplement: Supplementary file 15 — Unprocessed images [file 43587_2024_776_MOESM15_ESM.zip › SD_Figure_5_images/Fig_5_C_2053 if 2-Image Export-02_c1+2.jpg]

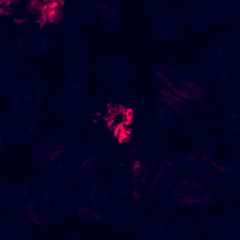

Supplement: Supplementary file 15 — Unprocessed images [file 43587_2024_776_MOESM15_ESM.zip › SD_Figure_5_images/Fig_5_L_MS1444-GD3-40X_figure version A.tiff]

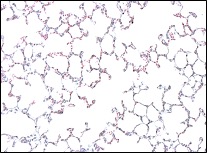

Supplement: Supplementary file 15 — Unprocessed images [file 43587_2024_776_MOESM15_ESM.zip › SD_Figure_5_images/Fig_5_F_HE 120 day.jpg]

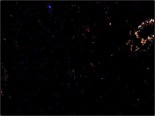

Supplement: Supplementary file 15 — Unprocessed images [file 43587_2024_776_MOESM15_ESM.zip › SD_Figure_5_images/Fig_5_F_POL 14 day.jpg]

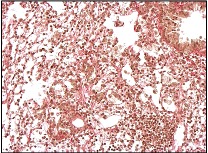

Supplement: Supplementary file 15 — Unprocessed images [file 43587_2024_776_MOESM15_ESM.zip › SD_Figure_5_images/Fig_5_F_SR 14 day.jpg]

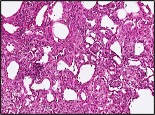

Supplement: Supplementary file 15 — Unprocessed images [file 43587_2024_776_MOESM15_ESM.zip › SD_Figure_5_images/Fig_5_F_HE 14 day.jpg]

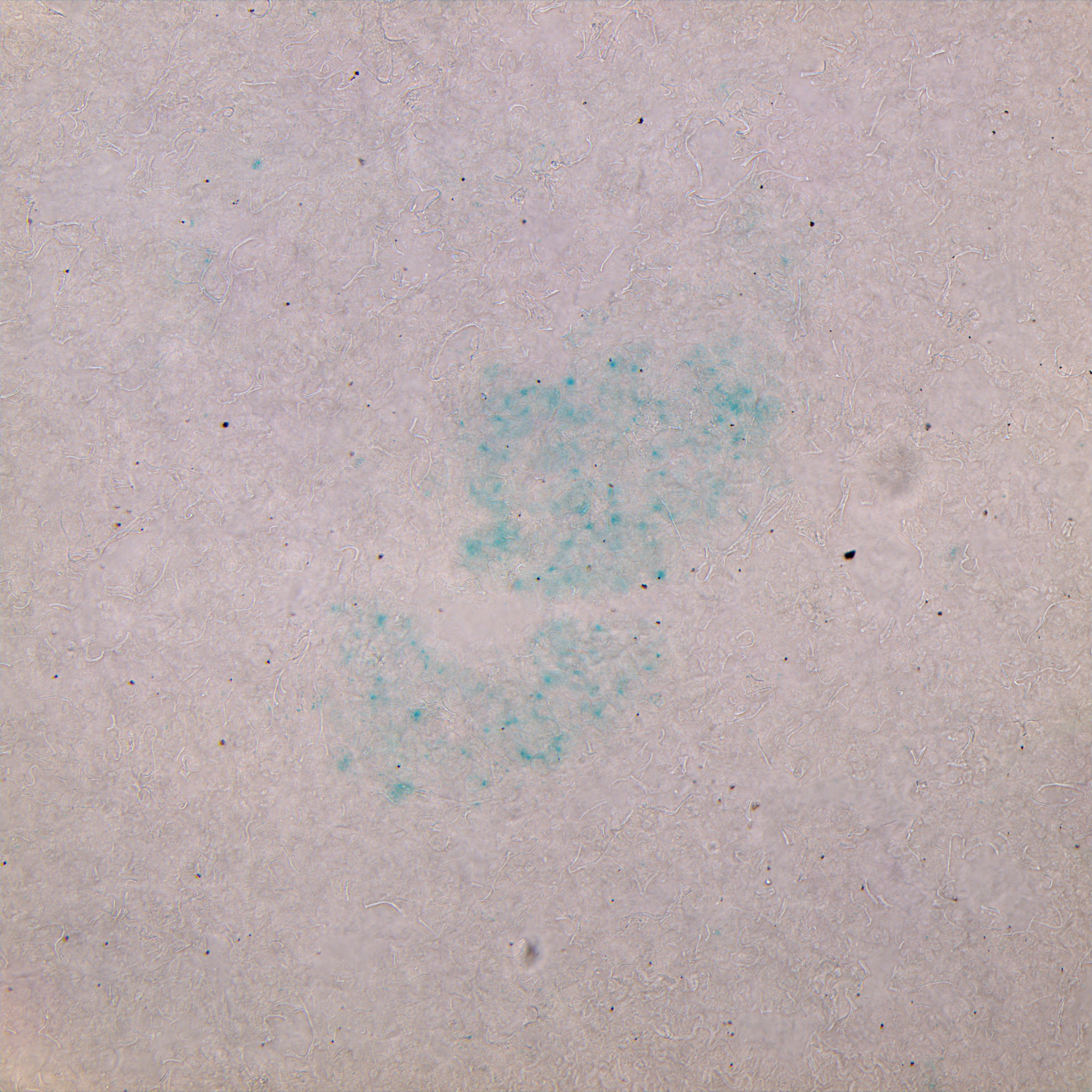

Supplement: Supplementary file 15 — Unprocessed images [file 43587_2024_776_MOESM15_ESM.zip › SD_Figure_5_images/Fig_5_C_2053 tl 2-Image Export-20_c1+2+3.jpg]

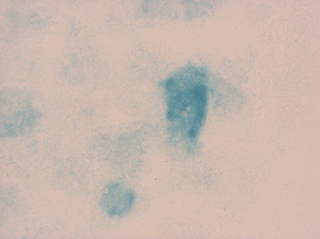

Supplement: Supplementary file 15 — Unprocessed images [file 43587_2024_776_MOESM15_ESM.zip › SD_Figure_5_images/Fig_5_L_MS1444-Xgal-40X_figure version A.tiff]

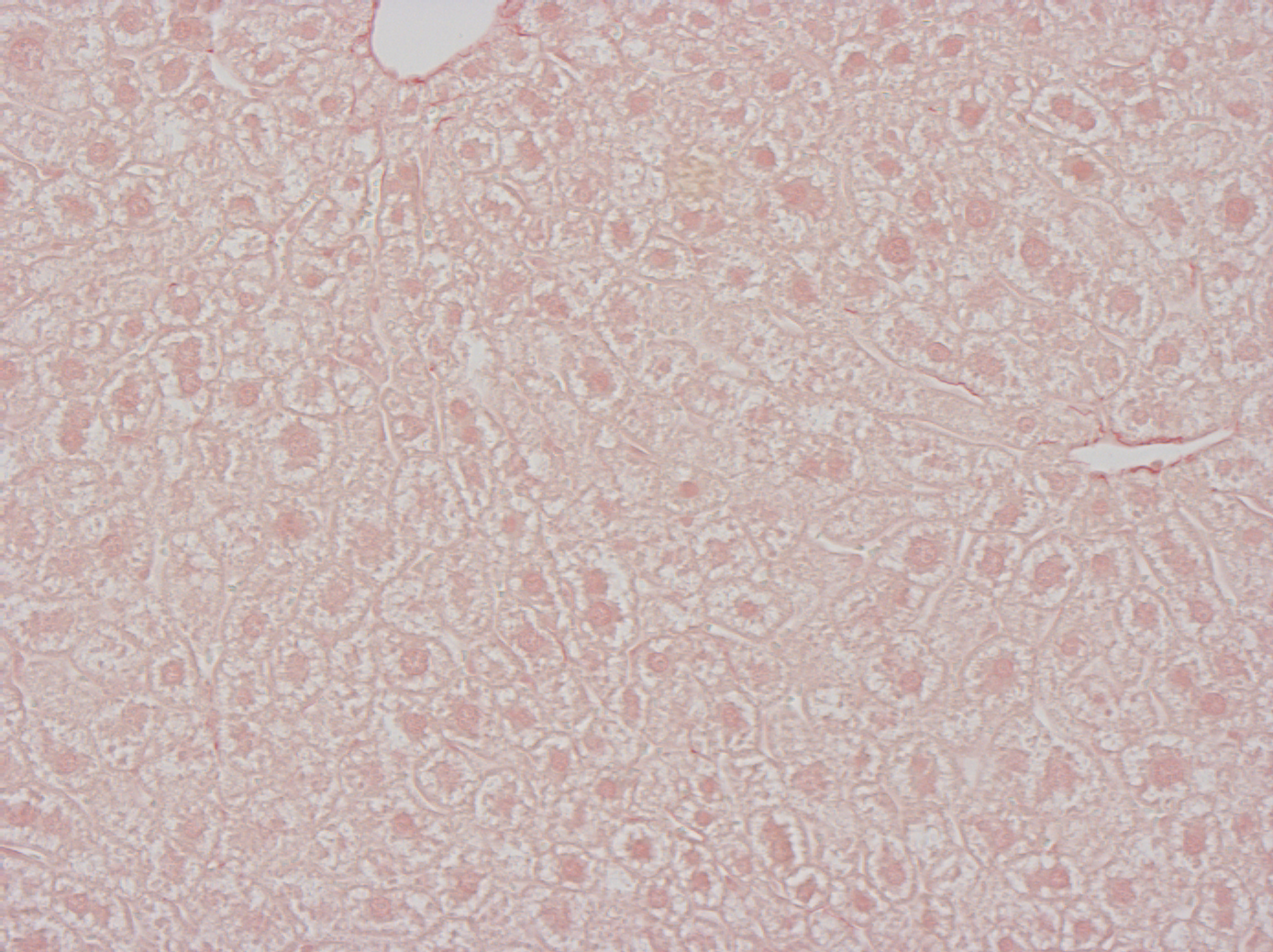

Supplement: Supplementary file 15 — Unprocessed images [file 43587_2024_776_MOESM15_ESM.zip › SD_Figure_5_images/ Fig_5_H_FOIE 18 CD1-09 BF.tif]

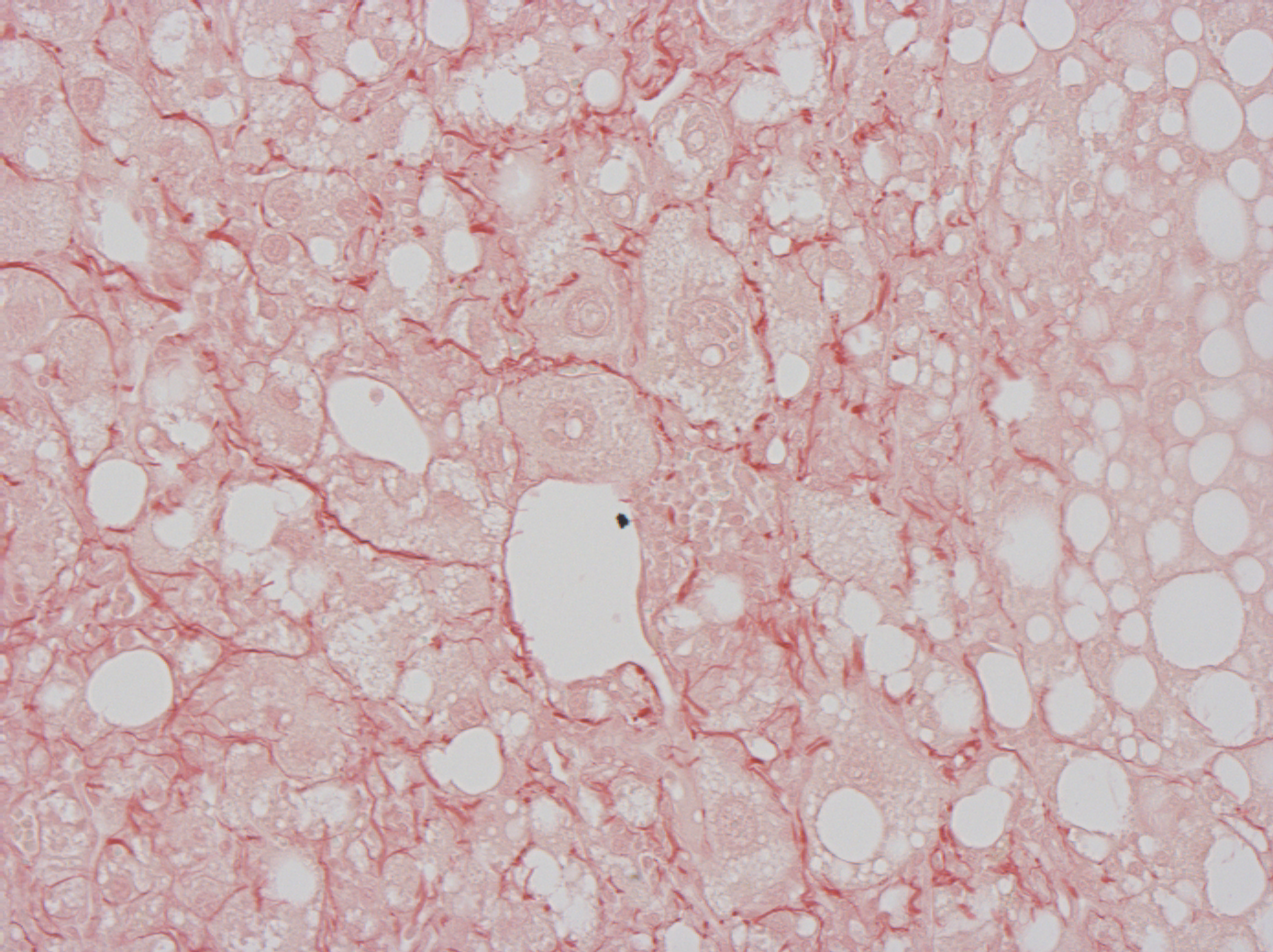

Supplement: Supplementary file 15 — Unprocessed images [file 43587_2024_776_MOESM15_ESM.zip › SD_Figure_5_images/ Fig_5_H_FOIE 5 WD1-06 BF.tif]

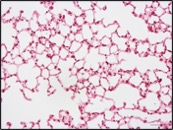

Supplement: Supplementary file 15 — Unprocessed images [file 43587_2024_776_MOESM15_ESM.zip › SD_Figure_5_images/Fig_5_A_HE PBS.jpg]

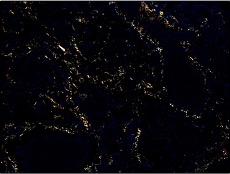

Supplement: Supplementary file 15 — Unprocessed images [file 43587_2024_776_MOESM15_ESM.zip › SD_Figure_5_images/Fig_5_A_POL bleo.jpg]

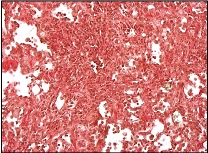

Supplement: Supplementary file 15 — Unprocessed images [file 43587_2024_776_MOESM15_ESM.zip › SD_Figure_5_images/Fig_5_F_SR 21day.jpg]

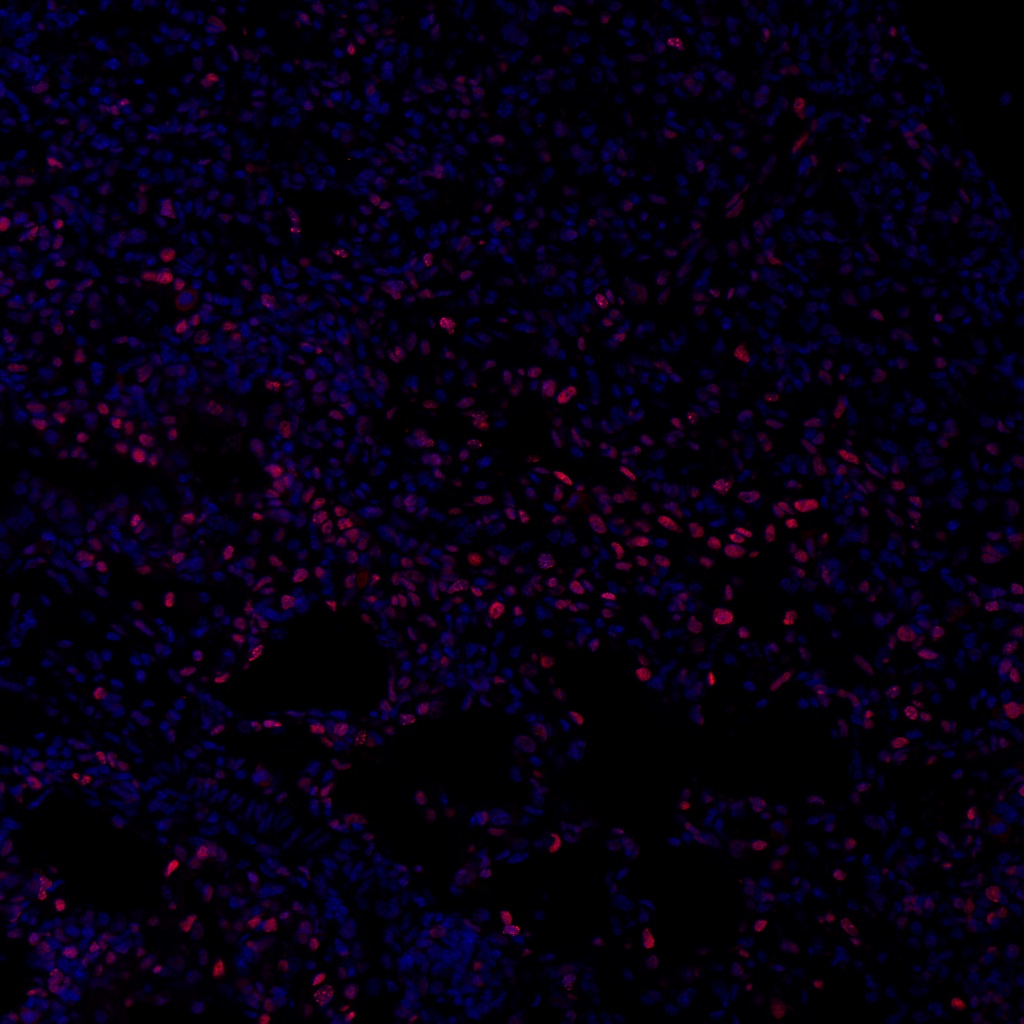

Supplement: Supplementary file 15 — Unprocessed images [file 43587_2024_776_MOESM15_ESM.zip › SD_Figure_5_images/Fig_5_F_bleo day 21 gd3 5-Image Export-20_c1+2.jpg]

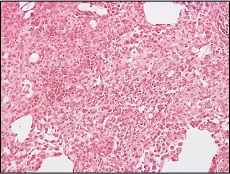

Supplement: Supplementary file 15 — Unprocessed images [file 43587_2024_776_MOESM15_ESM.zip › SD_Figure_5_images/Fig_5_A_HE bleo.jpg]

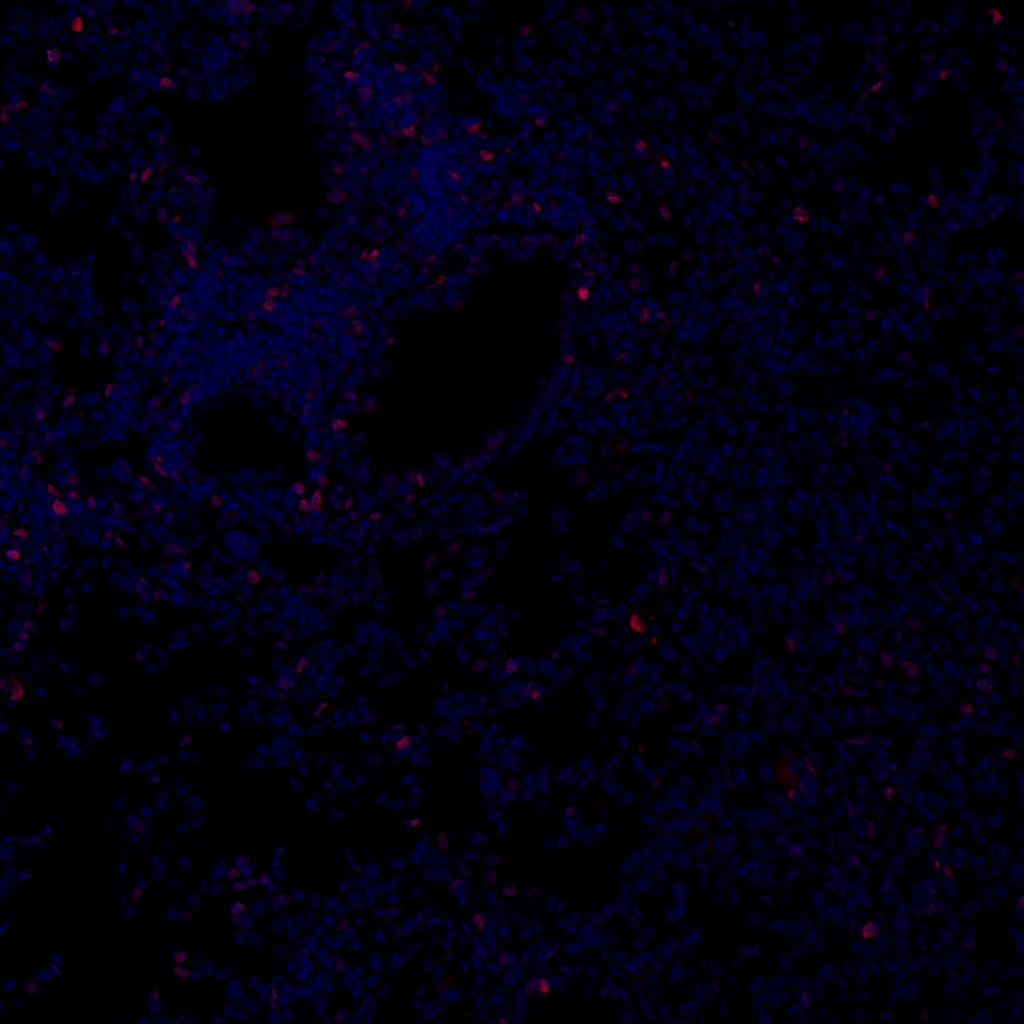

Supplement: Supplementary file 15 — Unprocessed images [file 43587_2024_776_MOESM15_ESM.zip › SD_Figure_5_images/Fig_5_F_bleo day 14 gd3 2-Image Export-03_c1+2.jpg]

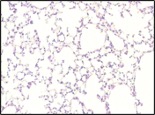

Supplement: Supplementary file 15 — Unprocessed images [file 43587_2024_776_MOESM15_ESM.zip › SD_Figure_5_images/Fig_5_F_HE 0 day.jpg]

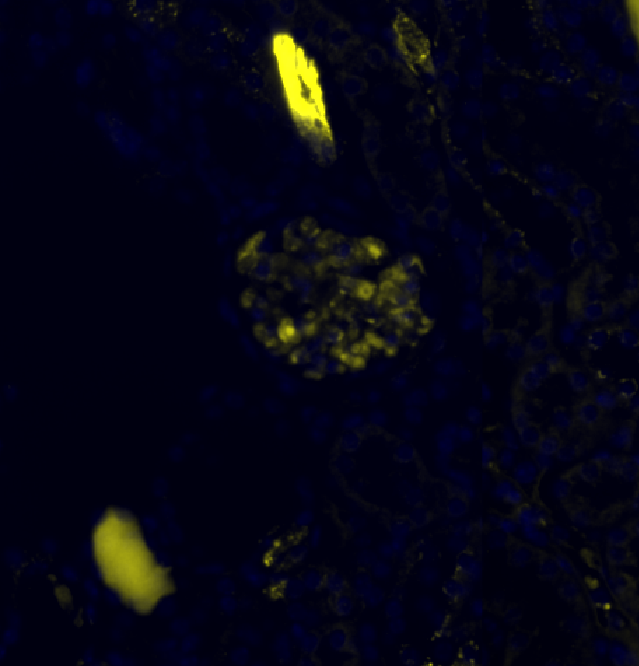

Supplement: Supplementary file 15 — Unprocessed images [file 43587_2024_776_MOESM15_ESM.zip › SD_Figure_5_images/Fig_5_M_image001.png]

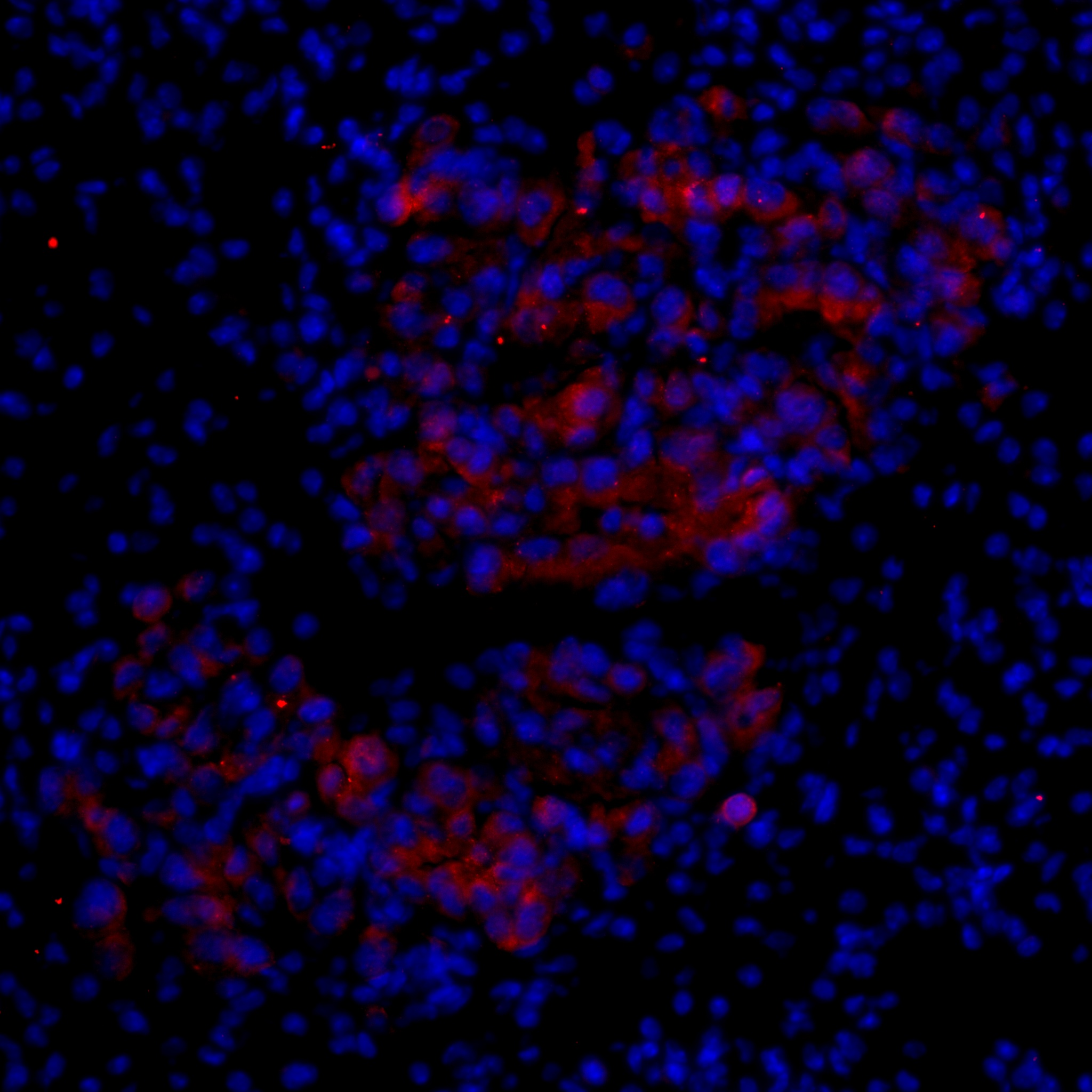

Supplement: Supplementary file 15 — Unprocessed images [file 43587_2024_776_MOESM15_ESM.zip › SD_Figure_5_images/Fig_5_C_2053 if 40x 8-Image Export-13_c1+2.jpg]

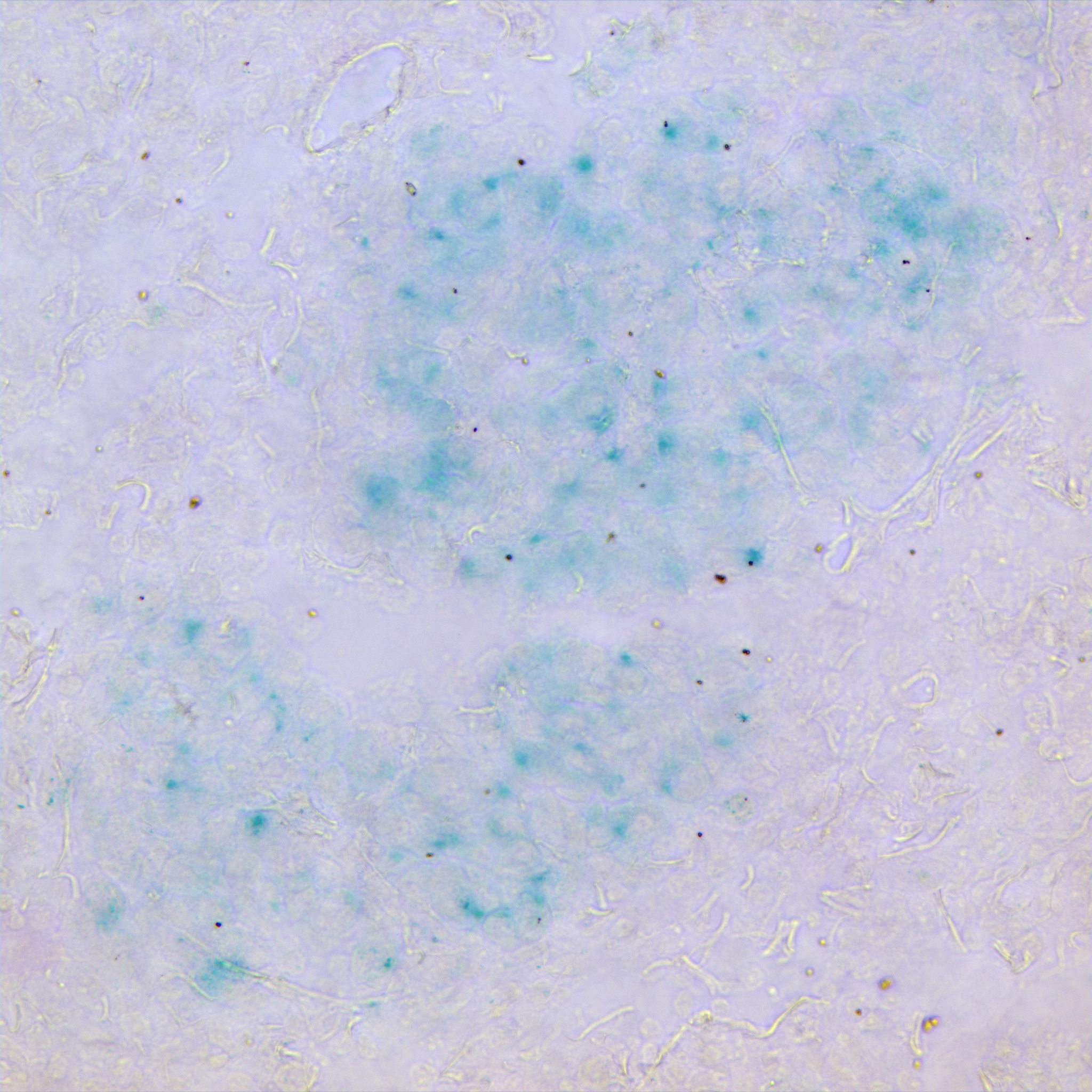

Supplement: Supplementary file 15 — Unprocessed images [file 43587_2024_776_MOESM15_ESM.zip › SD_Figure_5_images/Fig_5_C_2053tl 40x 8-Image Export-32_c1+2+3.jpg]

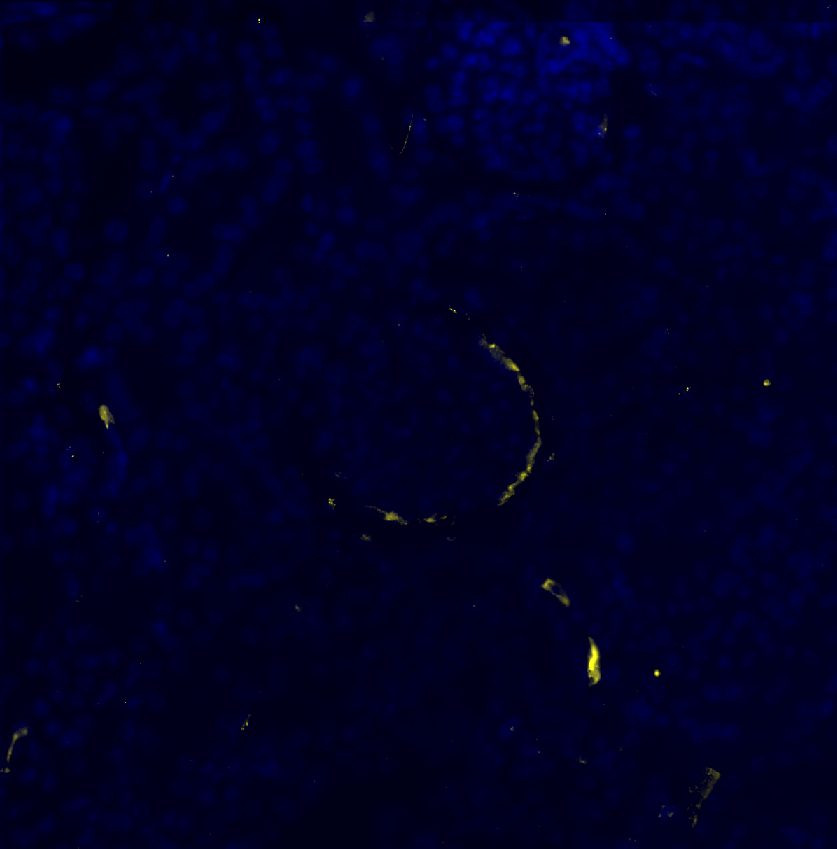

Supplement: Supplementary file 15 — Unprocessed images [file 43587_2024_776_MOESM15_ESM.zip › SD_Figure_5_images/Fig_5_M_image002.png]

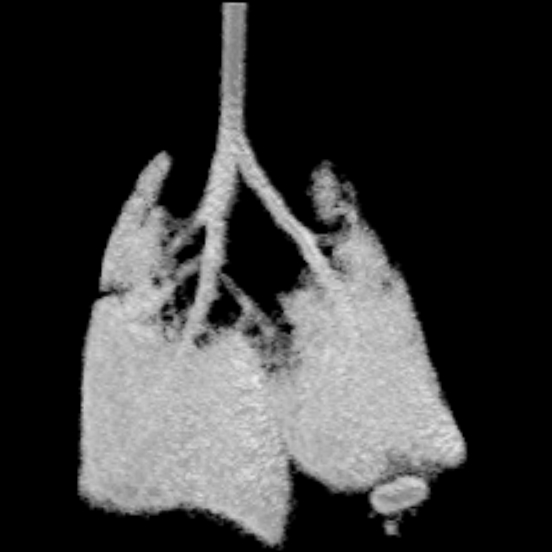

Supplement: Supplementary file 18 — Unprocessed images [file 43587_2024_776_MOESM18_ESM.zip › SD_Figure_7_images/Fig_7_F_IMAGE_S24_j15.jpeg]

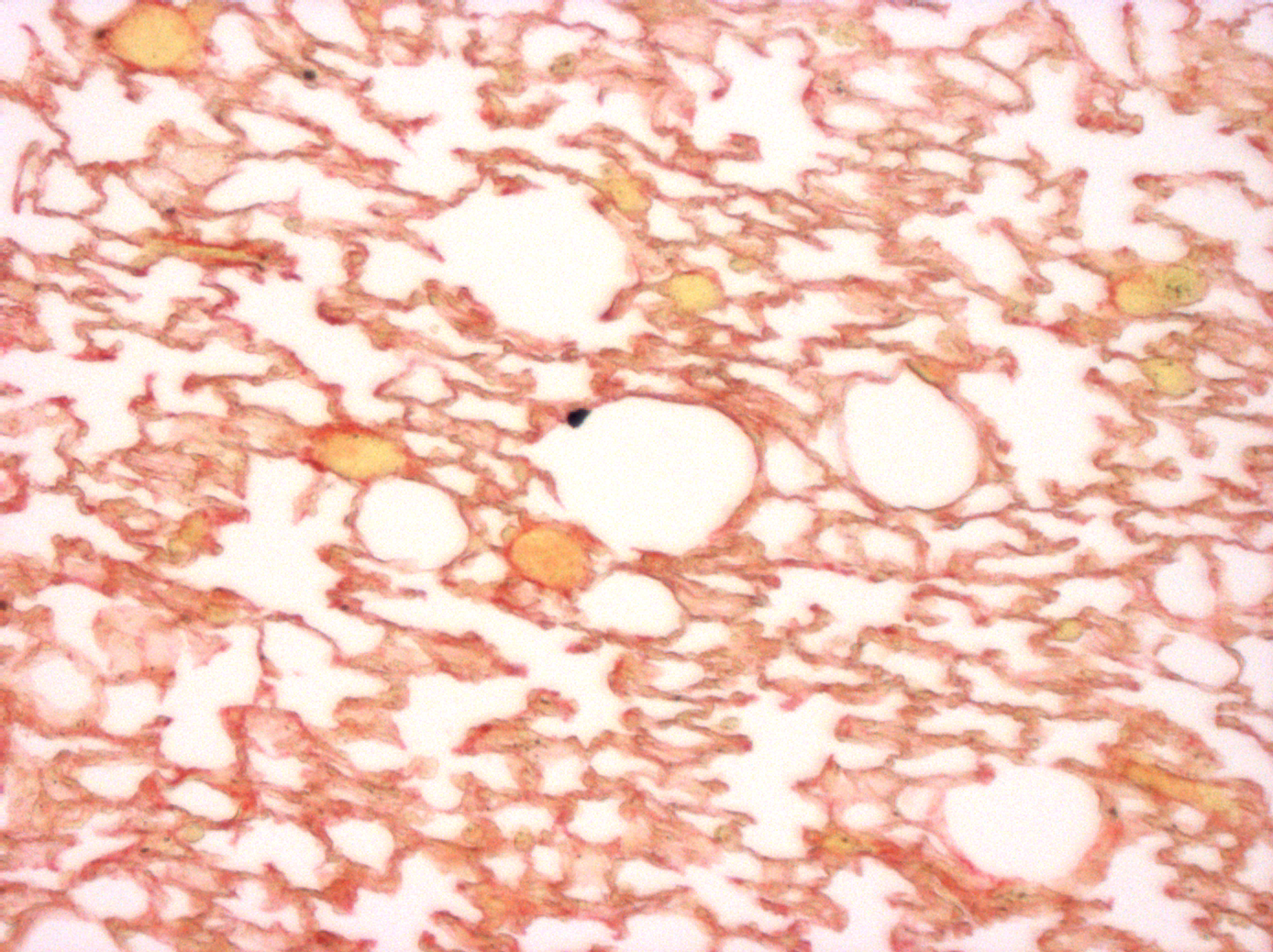

Supplement: Supplementary file 18 — Unprocessed images [file 43587_2024_776_MOESM18_ESM.zip › SD_Figure_7_images/Fig_7_J_Lung Male aGD3 65 04 BF.tif]

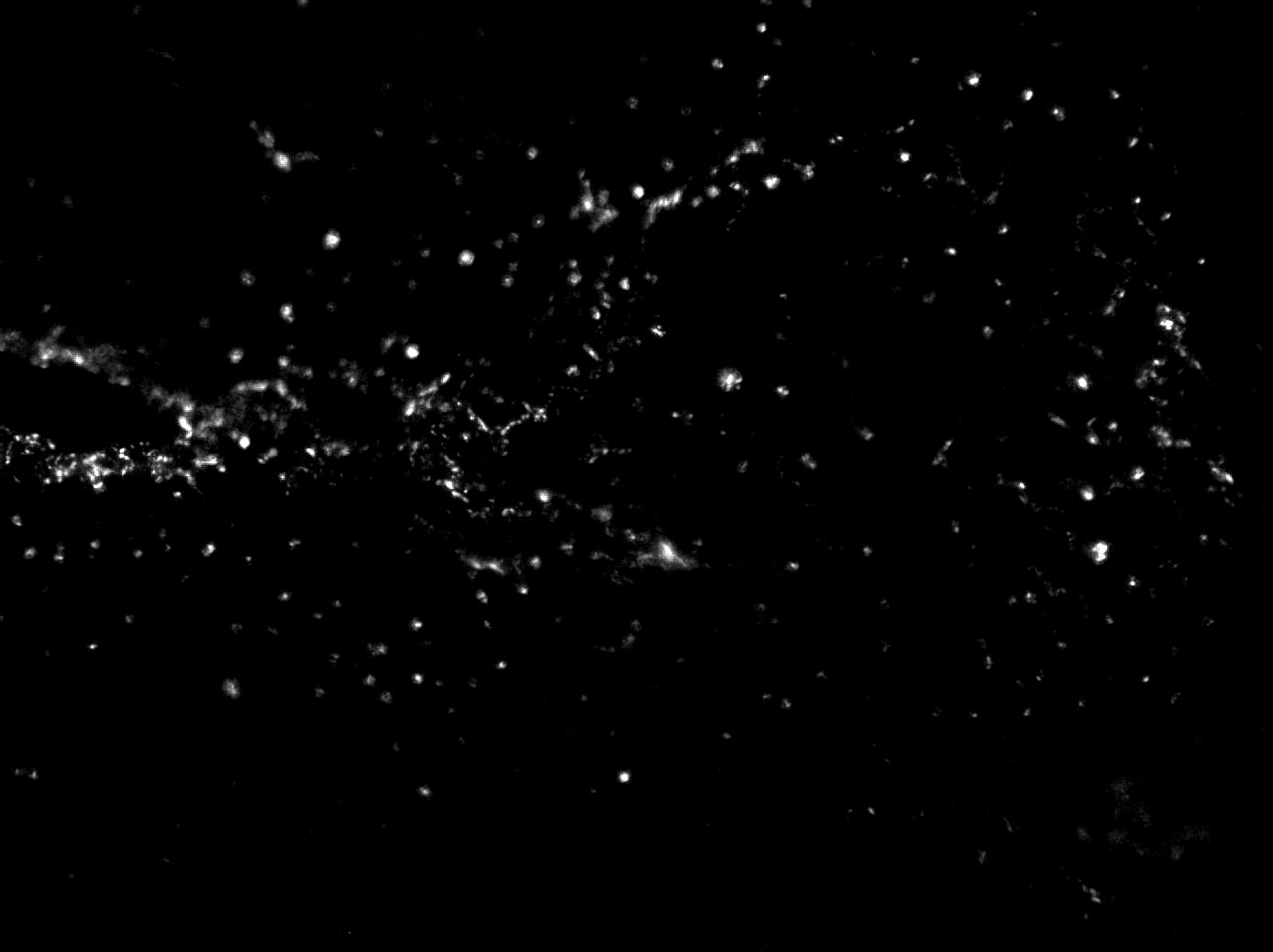

Supplement: Supplementary file 18 — Unprocessed images [file 43587_2024_776_MOESM18_ESM.zip › SD_Figure_7_images/Fig_7_L_Liver Male Ctl 4-07 8bit.jpg]

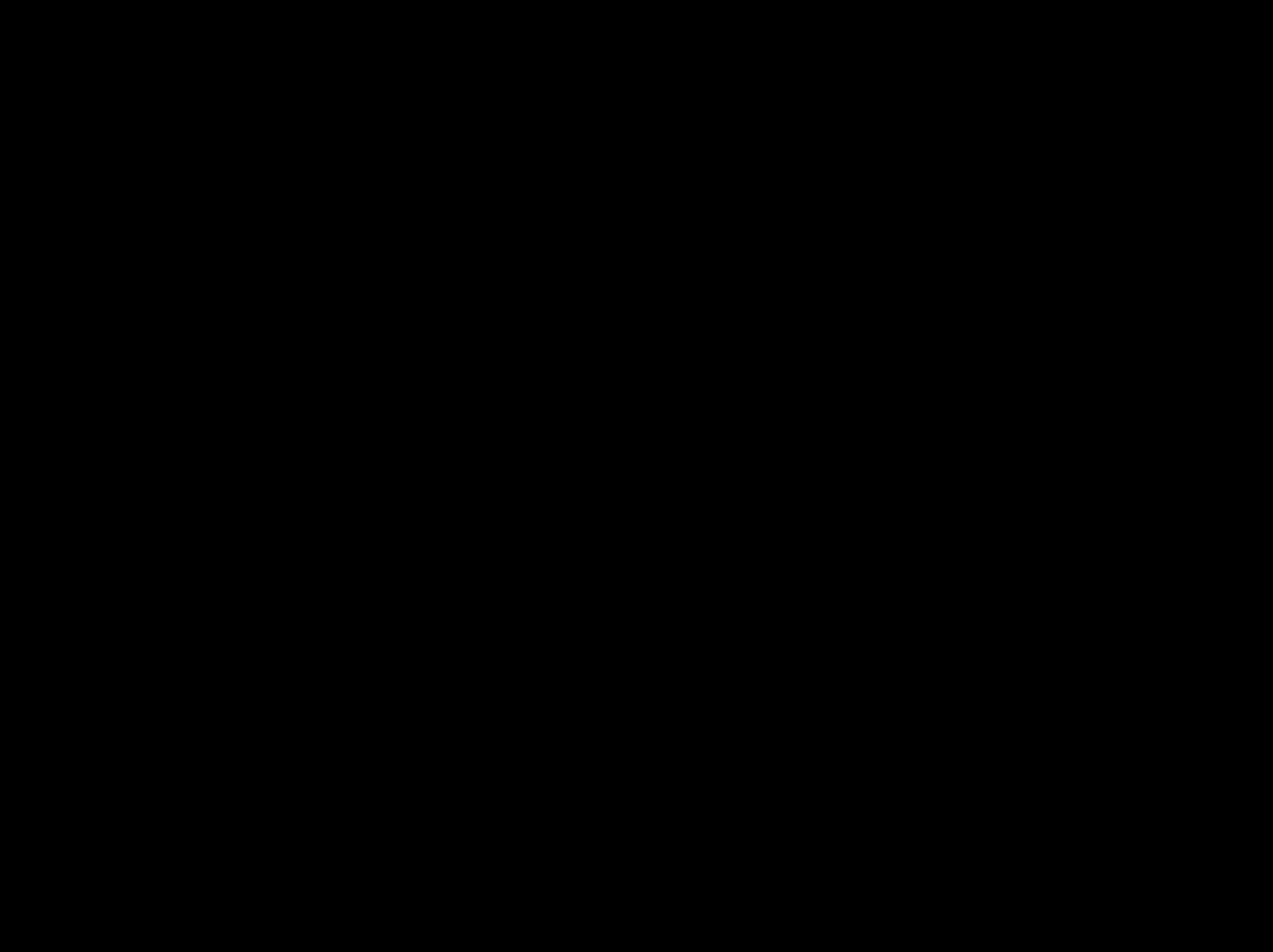

Supplement: Supplementary file 18 — Unprocessed images [file 43587_2024_776_MOESM18_ESM.zip › SD_Figure_7_images/Fig_7_L_Liver Male aGD3 66-05-1 8bit.tif]

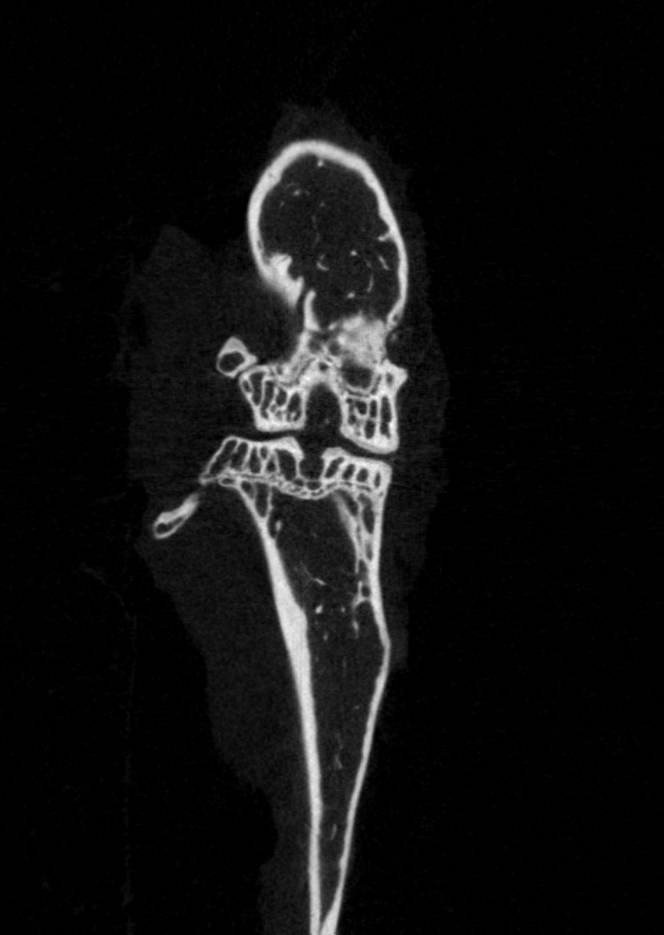

Supplement: Supplementary file 18 — Unprocessed images [file 43587_2024_776_MOESM18_ESM.zip › SD_Figure_7_images/Fig_7_M_uCT-groupe a-GD3.png]

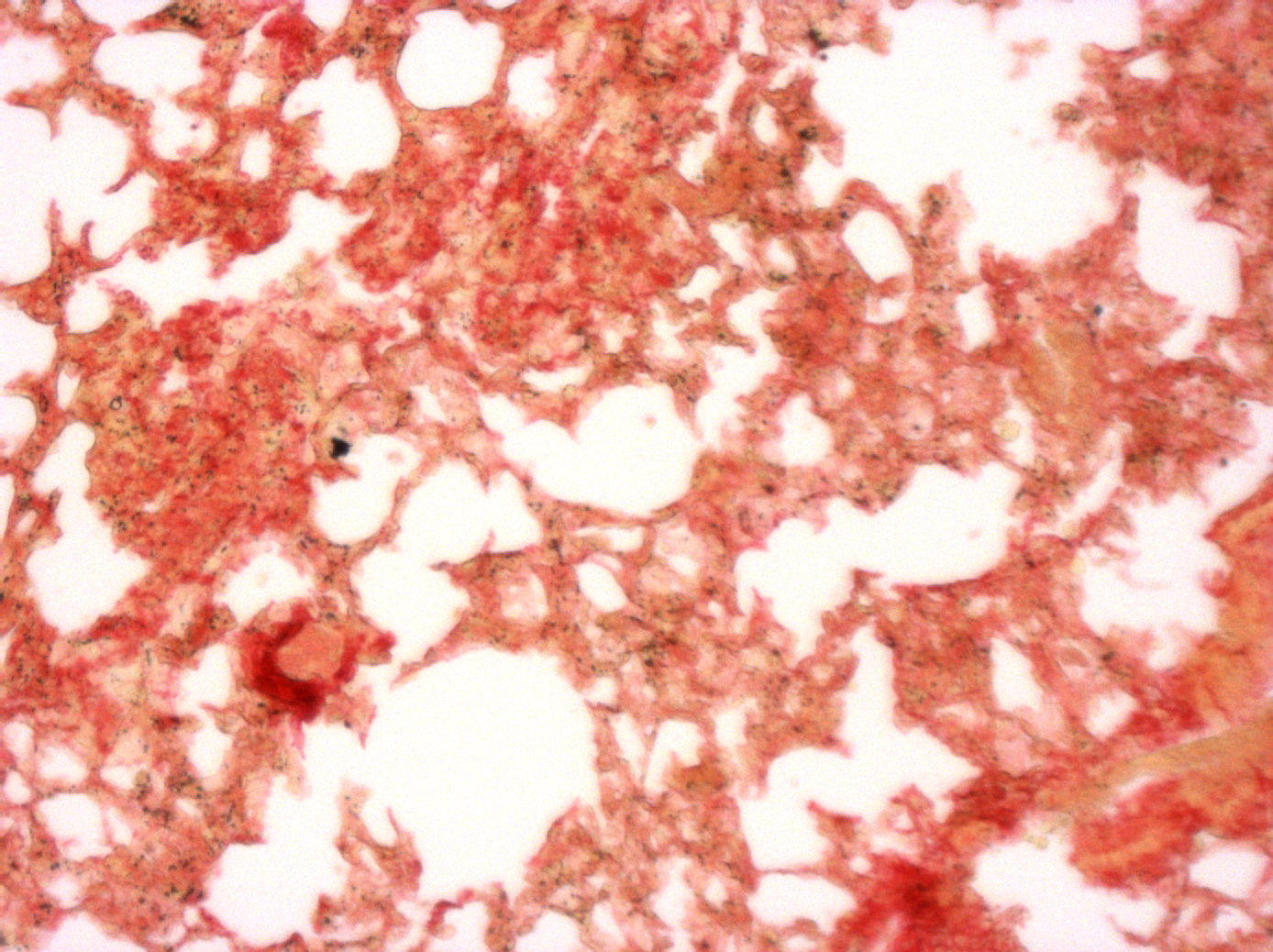

Supplement: Supplementary file 18 — Unprocessed images [file 43587_2024_776_MOESM18_ESM.zip › SD_Figure_7_images/Fig_7_J_Lung Male CTL 6 03 BF.tif]

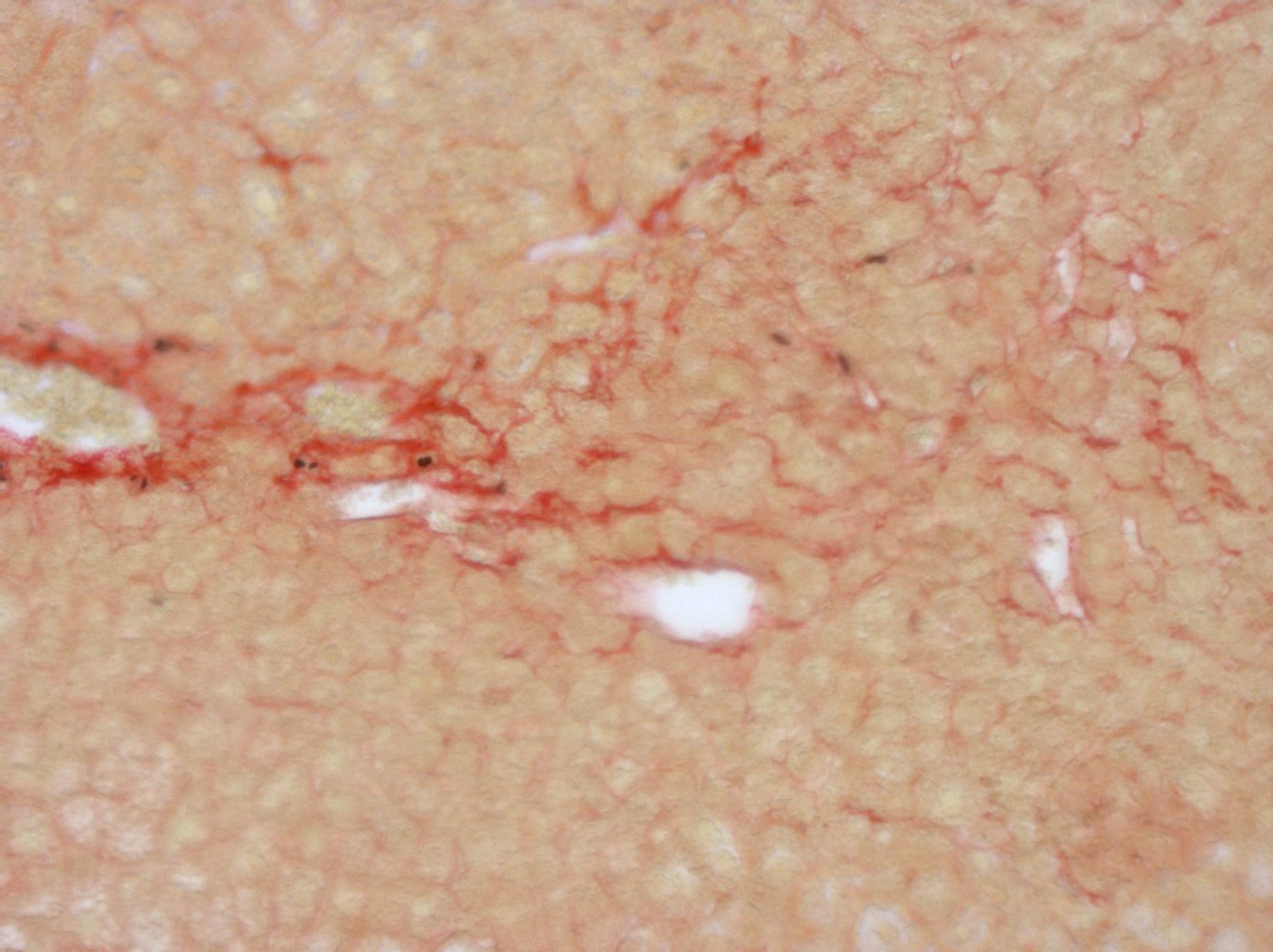

Supplement: Supplementary file 18 — Unprocessed images [file 43587_2024_776_MOESM18_ESM.zip › SD_Figure_7_images/Fig_7_L_Liver Male Ctl 4-07.jpg]

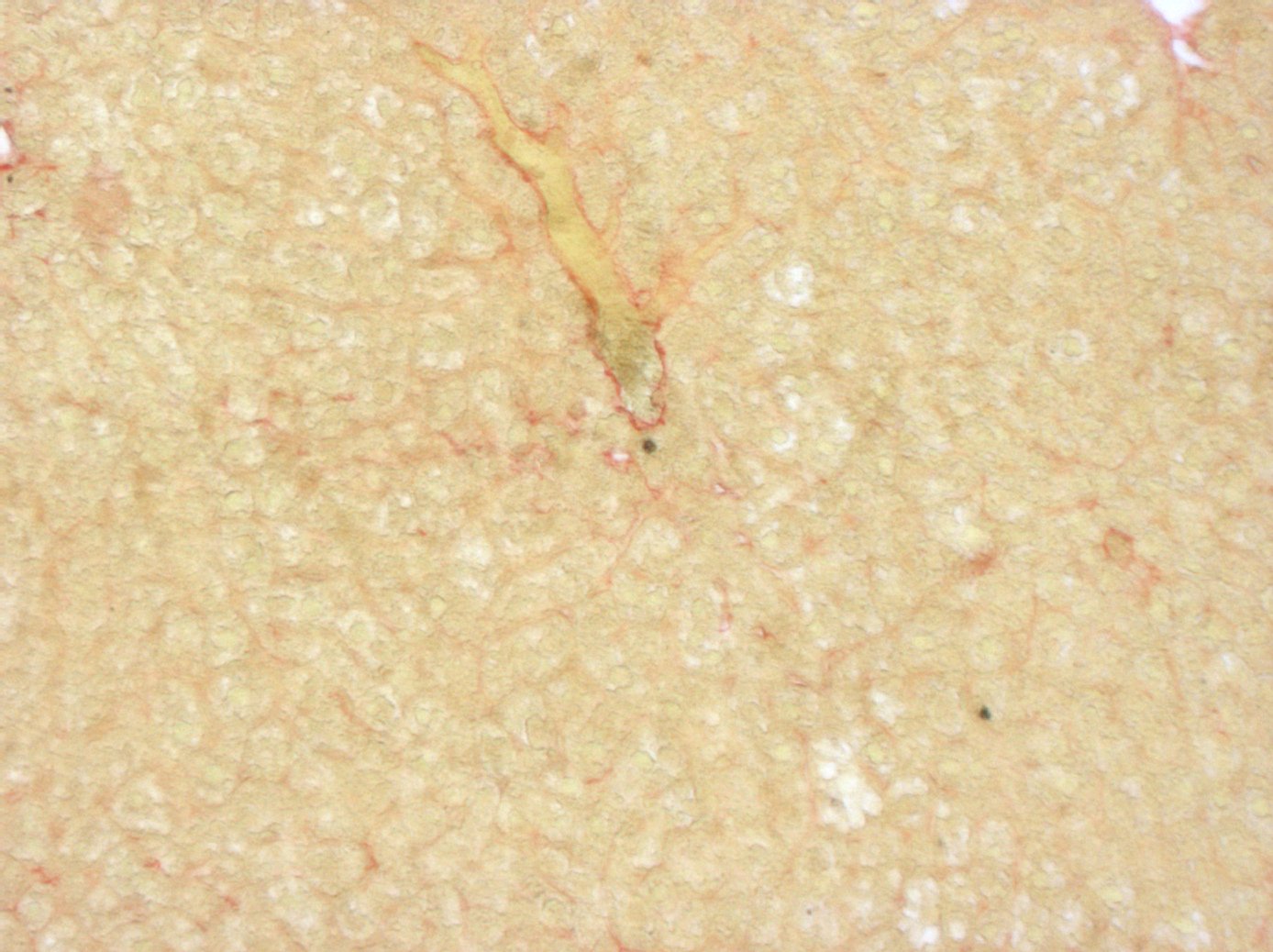

Supplement: Supplementary file 18 — Unprocessed images [file 43587_2024_776_MOESM18_ESM.zip › SD_Figure_7_images/Fig_7_L_Liver Male aGD3 66-05.jpg]

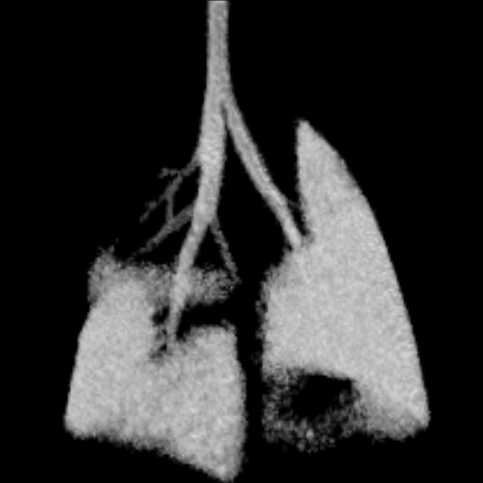

Supplement: Supplementary file 18 — Unprocessed images [file 43587_2024_776_MOESM18_ESM.zip › SD_Figure_7_images/Fig_7_F_IMAGE_S31_j15.jpeg]

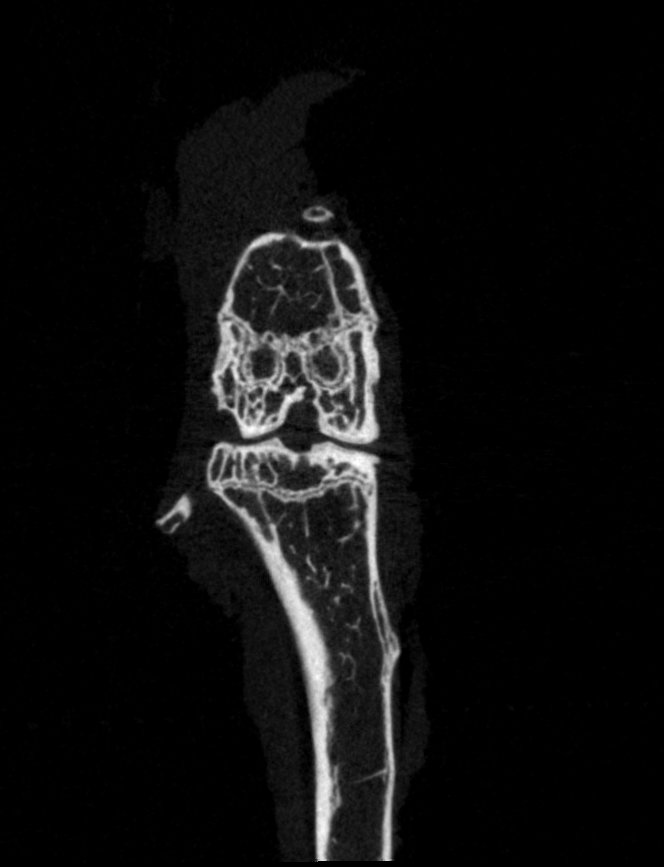

Supplement: Supplementary file 18 — Unprocessed images [file 43587_2024_776_MOESM18_ESM.zip › SD_Figure_7_images/Fig_7_M_uCT-groupe SHAM.png]

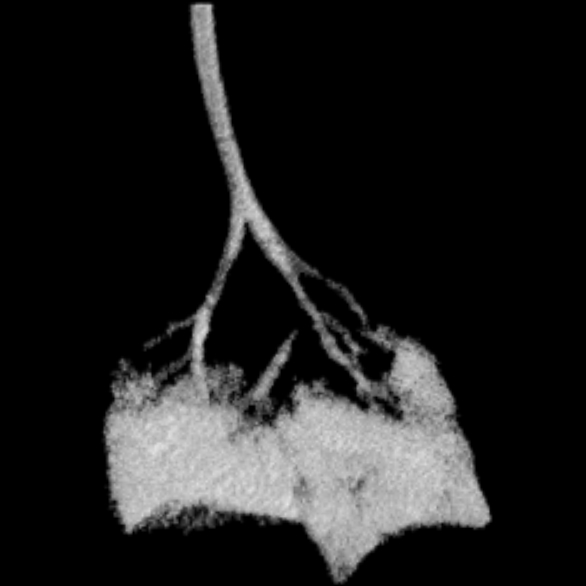

Supplement: Supplementary file 18 — Unprocessed images [file 43587_2024_776_MOESM18_ESM.zip › SD_Figure_7_images/Fig_7_F_IMAGE_S24_j27.jpeg]

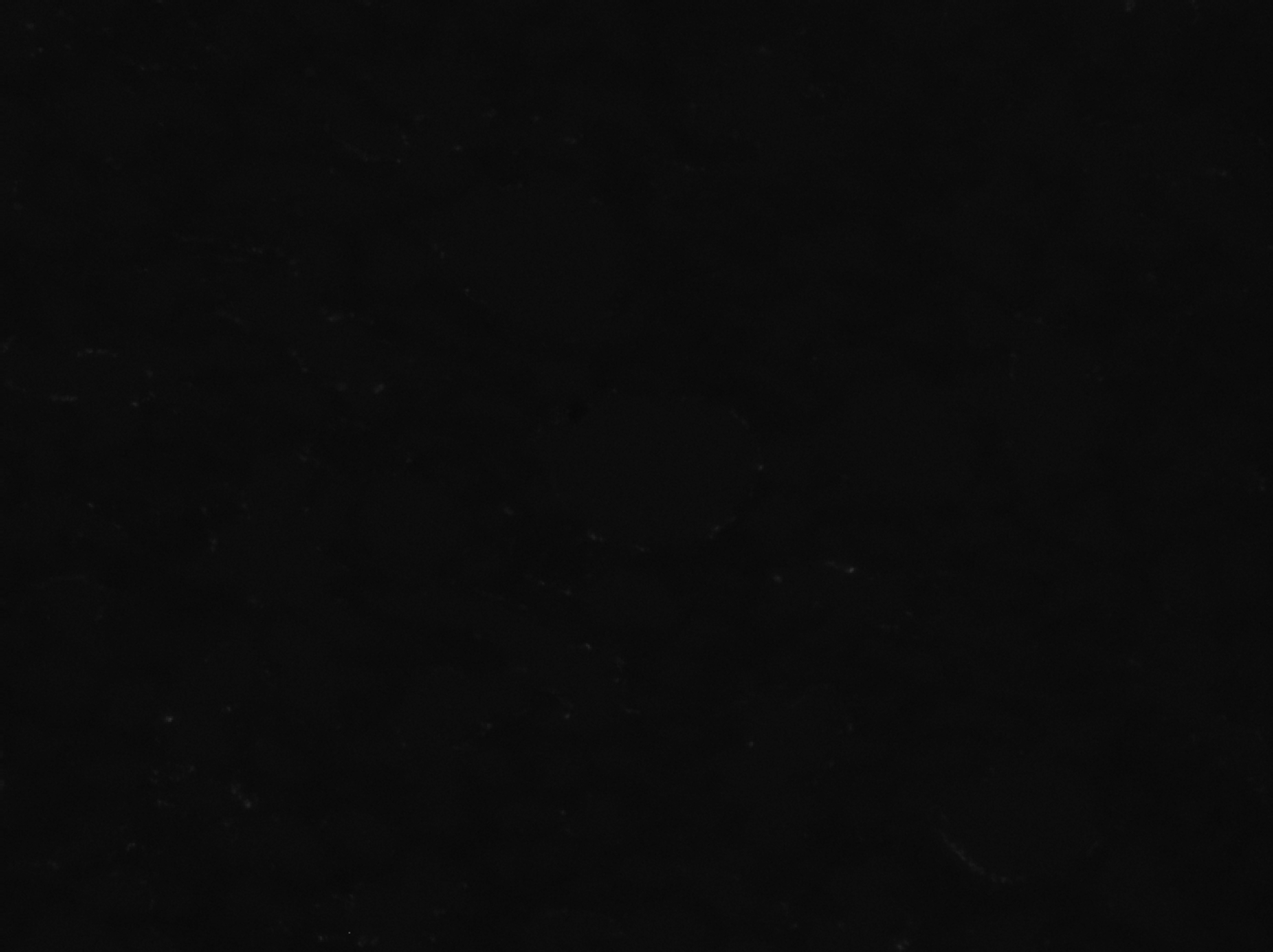

Supplement: Supplementary file 18 — Unprocessed images [file 43587_2024_776_MOESM18_ESM.zip › SD_Figure_7_images/Fig_7_J_Lung Male aGD3 65 04 PL.tif]

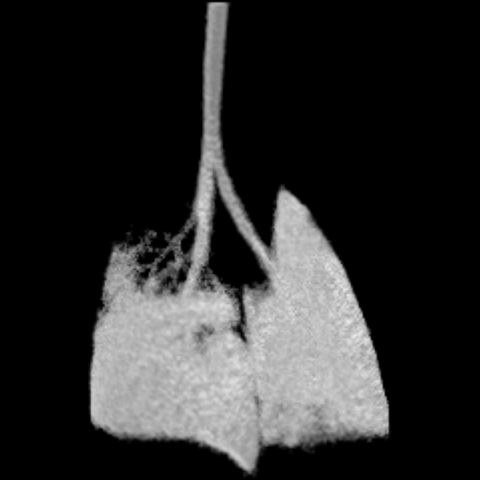

Supplement: Supplementary file 18 — Unprocessed images [file 43587_2024_776_MOESM18_ESM.zip › SD_Figure_7_images/Fig_7_F_IMAGE_S31_j27.jpg]

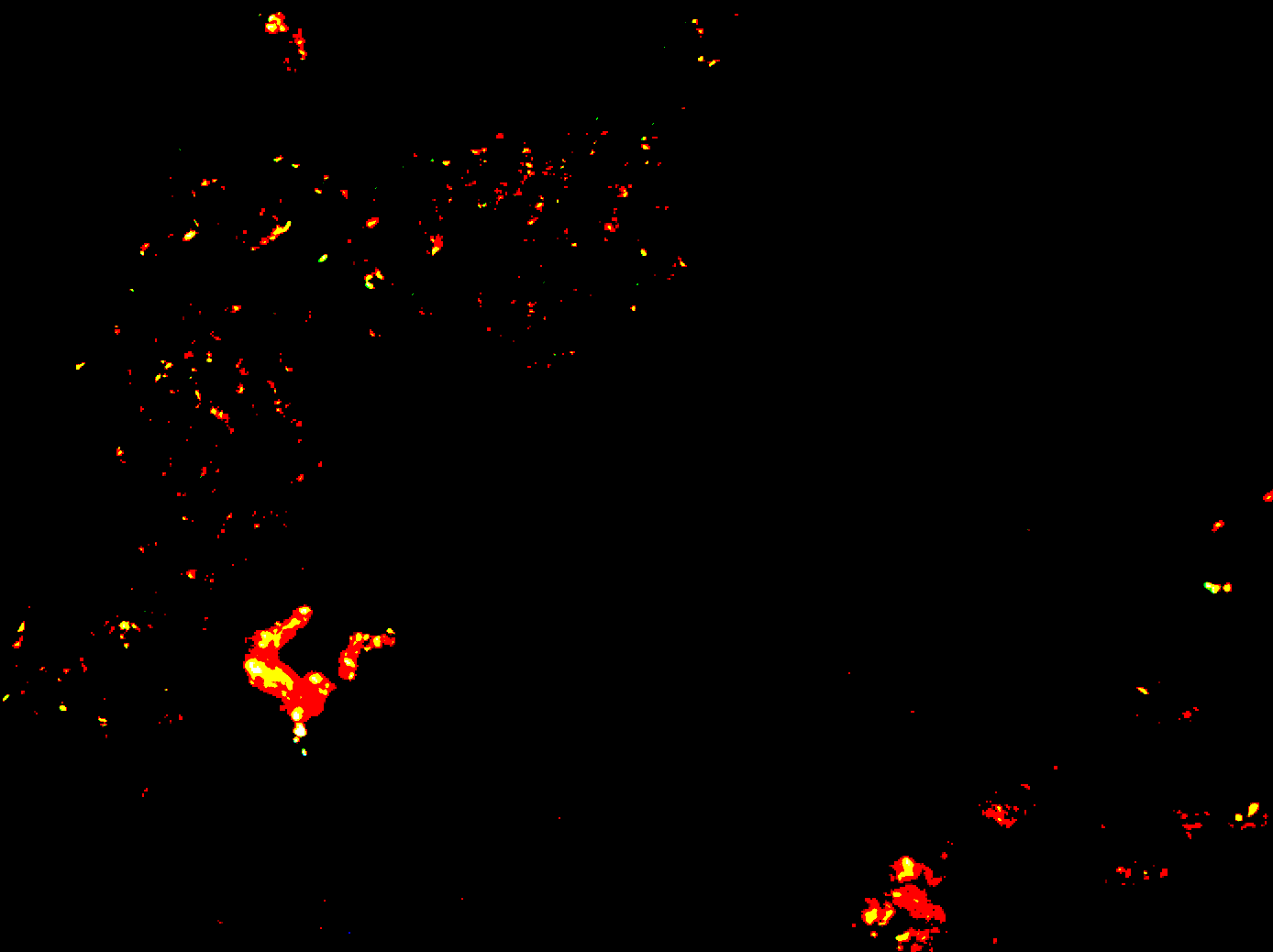

Supplement: Supplementary file 18 — Unprocessed images [file 43587_2024_776_MOESM18_ESM.zip › SD_Figure_7_images/Fig_7_J_Lung Male CTL 6 03 PL.tif]

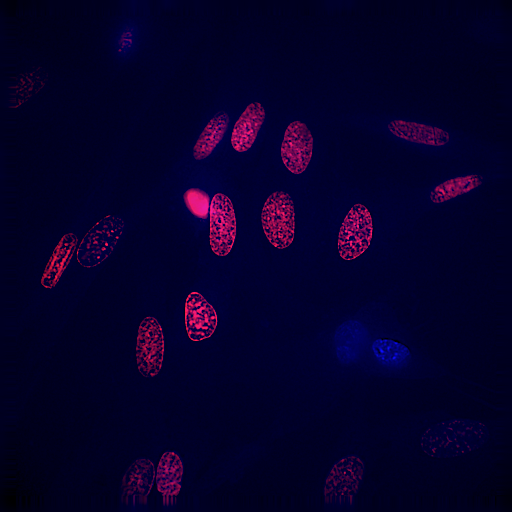

Supplement: Supplementary file 20 — Unprocessed images [file 43587_2024_776_MOESM20_ESM.zip › SD_ED_1_images/Ext_Data_1_B_EDU_PDL30.png]

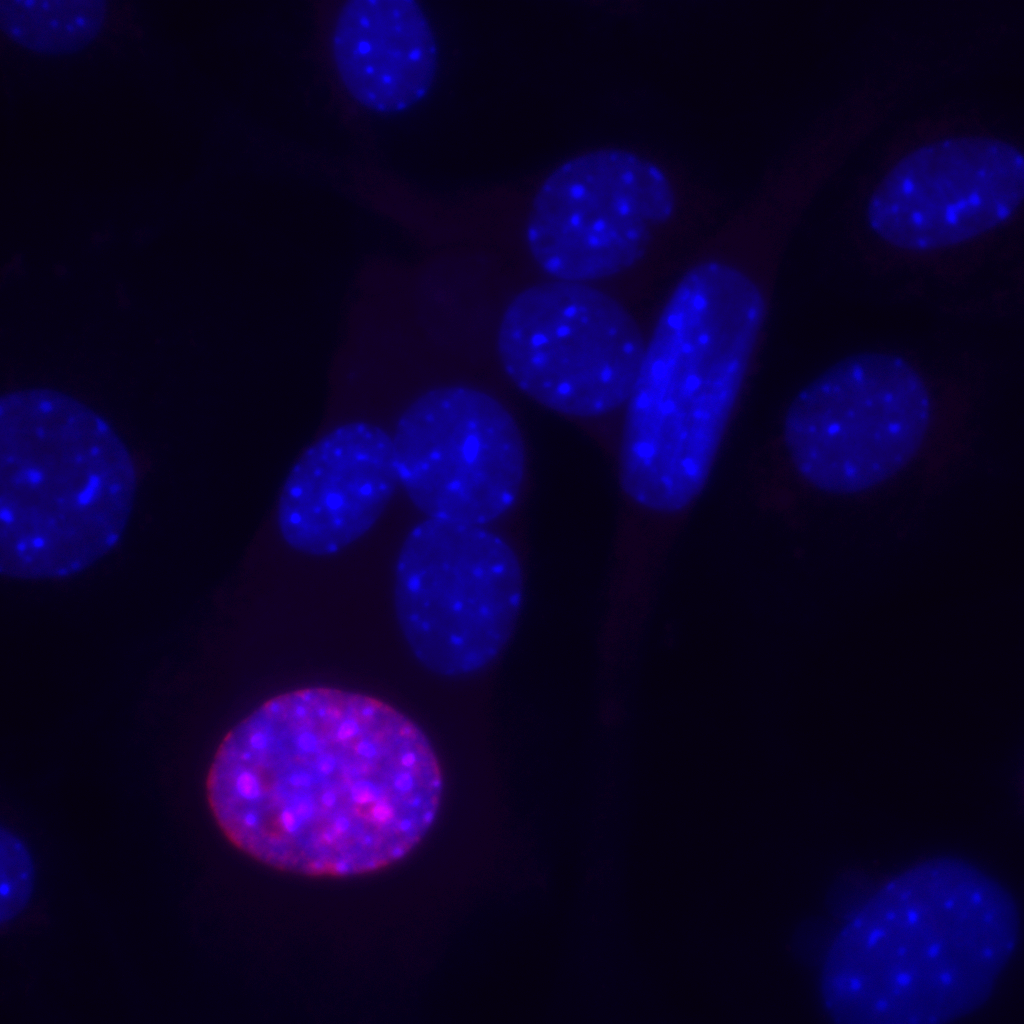

Supplement: Supplementary file 20 — Unprocessed images [file 43587_2024_776_MOESM20_ESM.zip › SD_ED_1_images/Ext_Fig_1_C_MEF_EDU.tif]

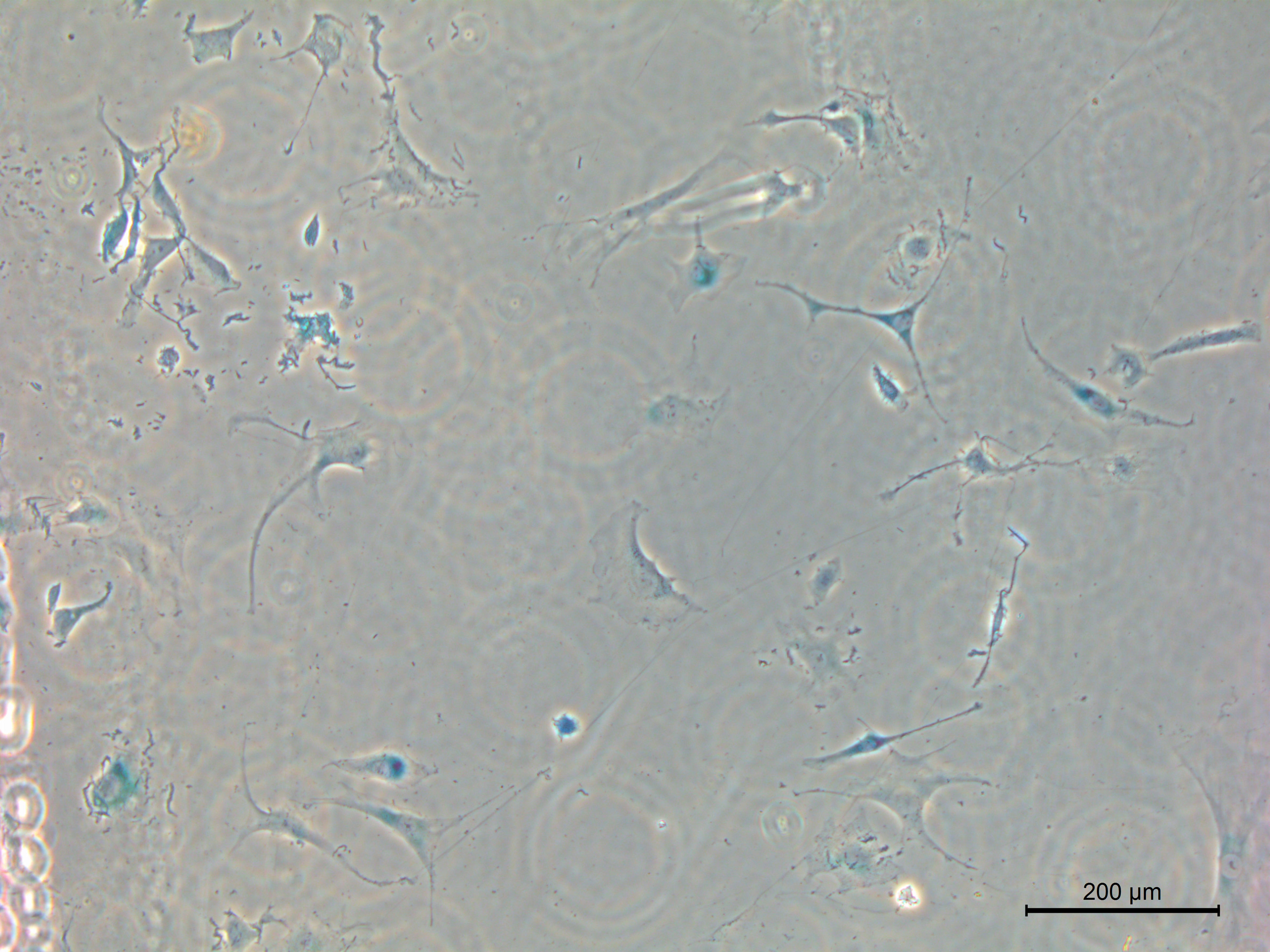

Supplement: Supplementary file 20 — Unprocessed images [file 43587_2024_776_MOESM20_ESM.zip › SD_ED_1_images/Ext_Fig_1_C_MEF_BGAL.tif]

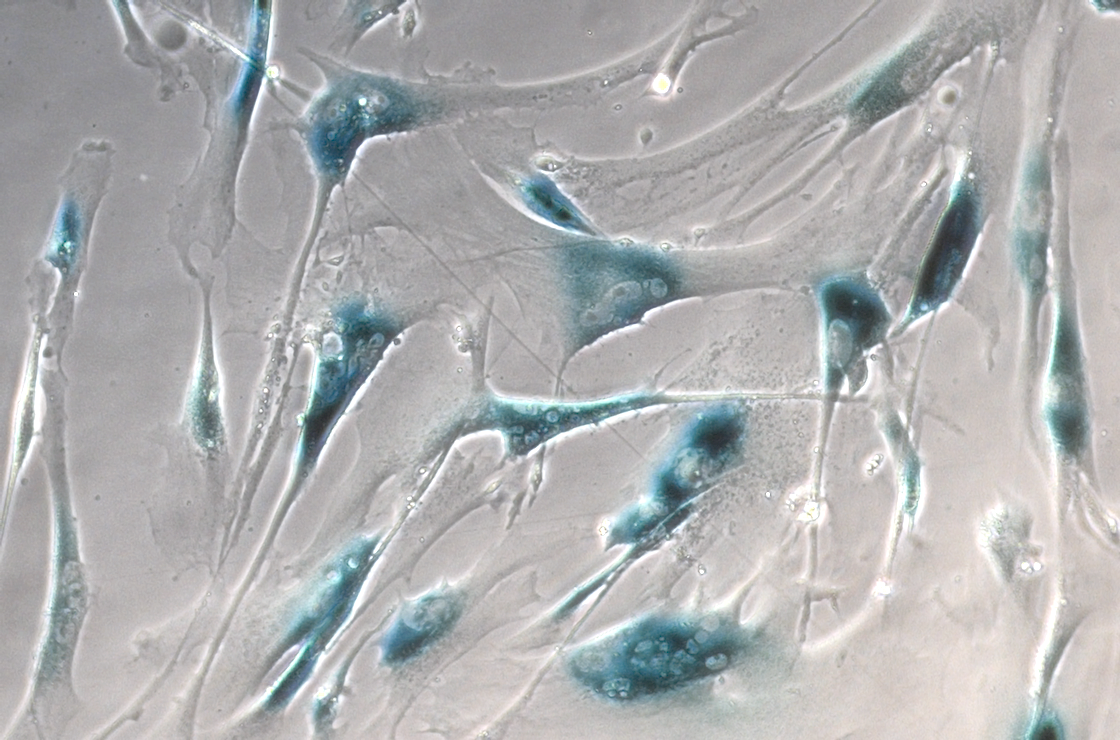

Supplement: Supplementary file 20 — Unprocessed images [file 43587_2024_776_MOESM20_ESM.zip › SD_ED_1_images/Ext_Data_1_B_BGAL_RepSen.png]

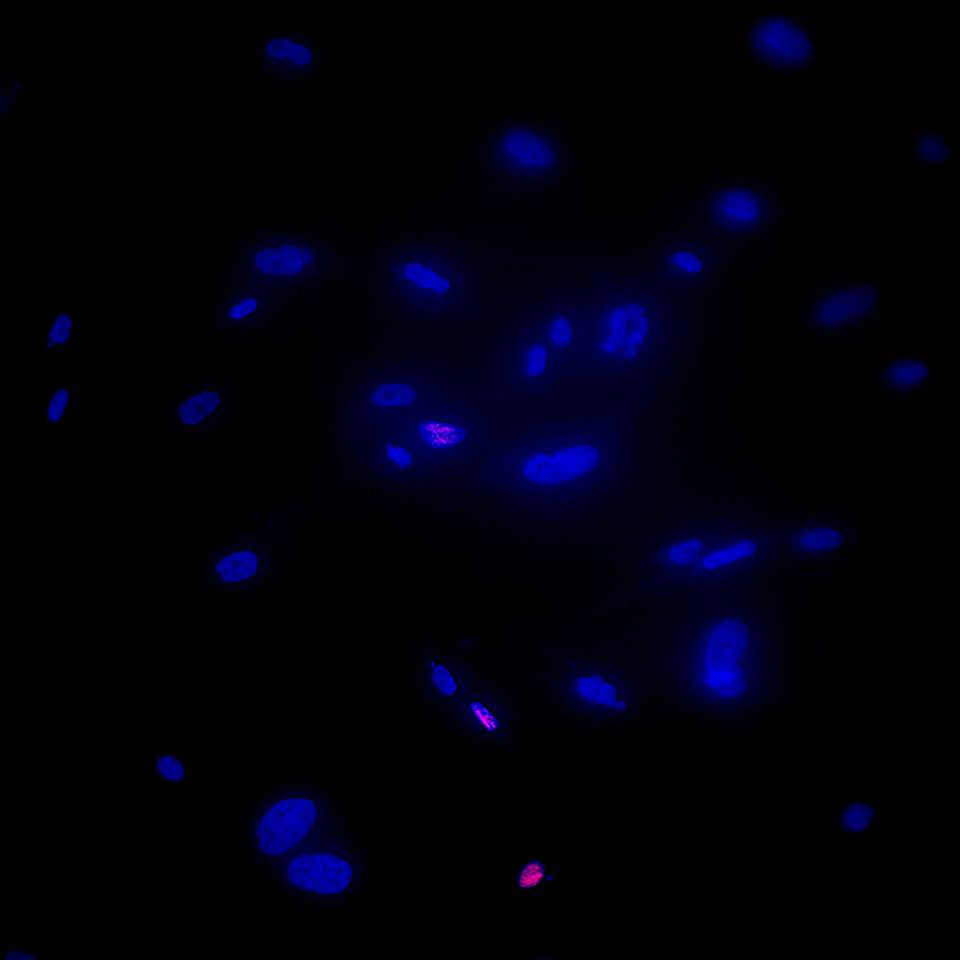

Supplement: Supplementary file 20 — Unprocessed images [file 43587_2024_776_MOESM20_ESM.zip › SD_ED_1_images/Ext_Data_1_B_EDU_RepSen.png]

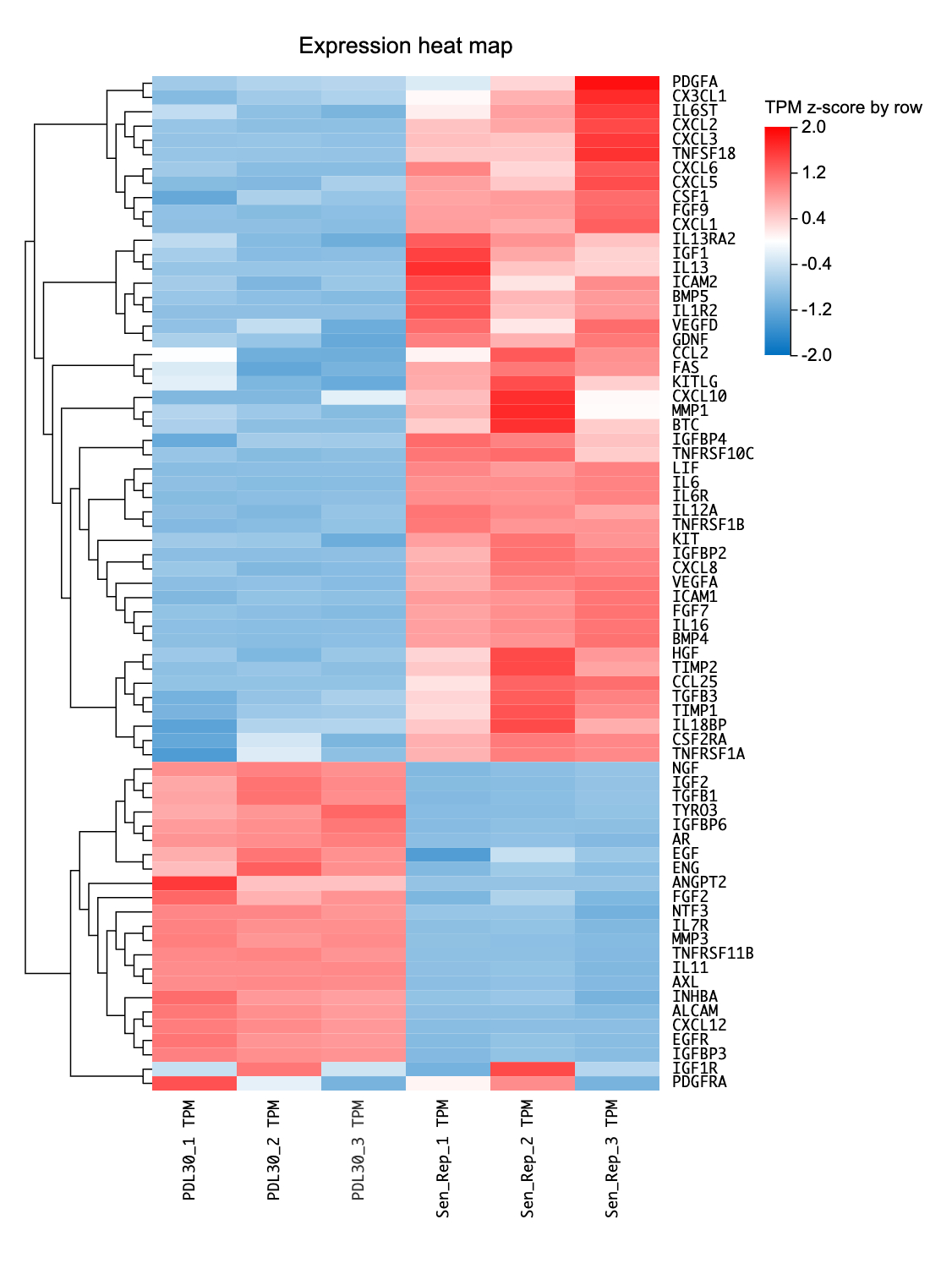

Supplement: Supplementary file 20 — Unprocessed images [file 43587_2024_776_MOESM20_ESM.zip › SD_ED_1_images/Ext_Fig_1_J_DEGs cluster heatmaps_2087.jpg]

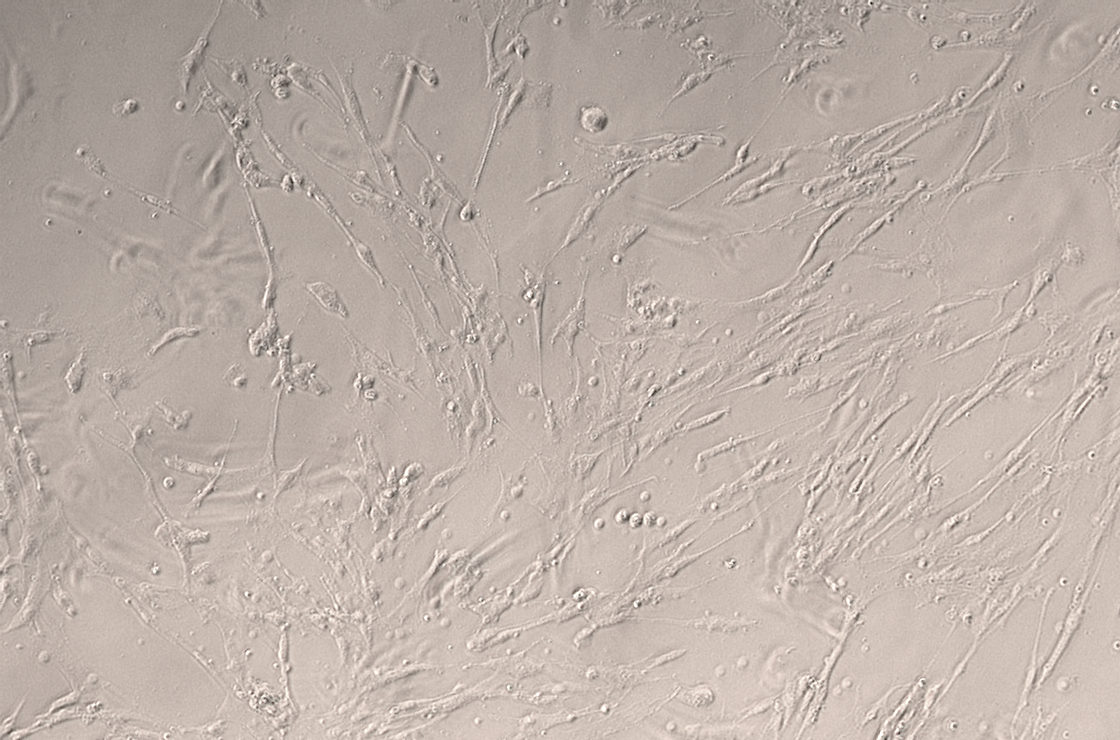

Supplement: Supplementary file 20 — Unprocessed images [file 43587_2024_776_MOESM20_ESM.zip › SD_ED_1_images/Ext_Data_1_B_BGAL_PDL30.png]

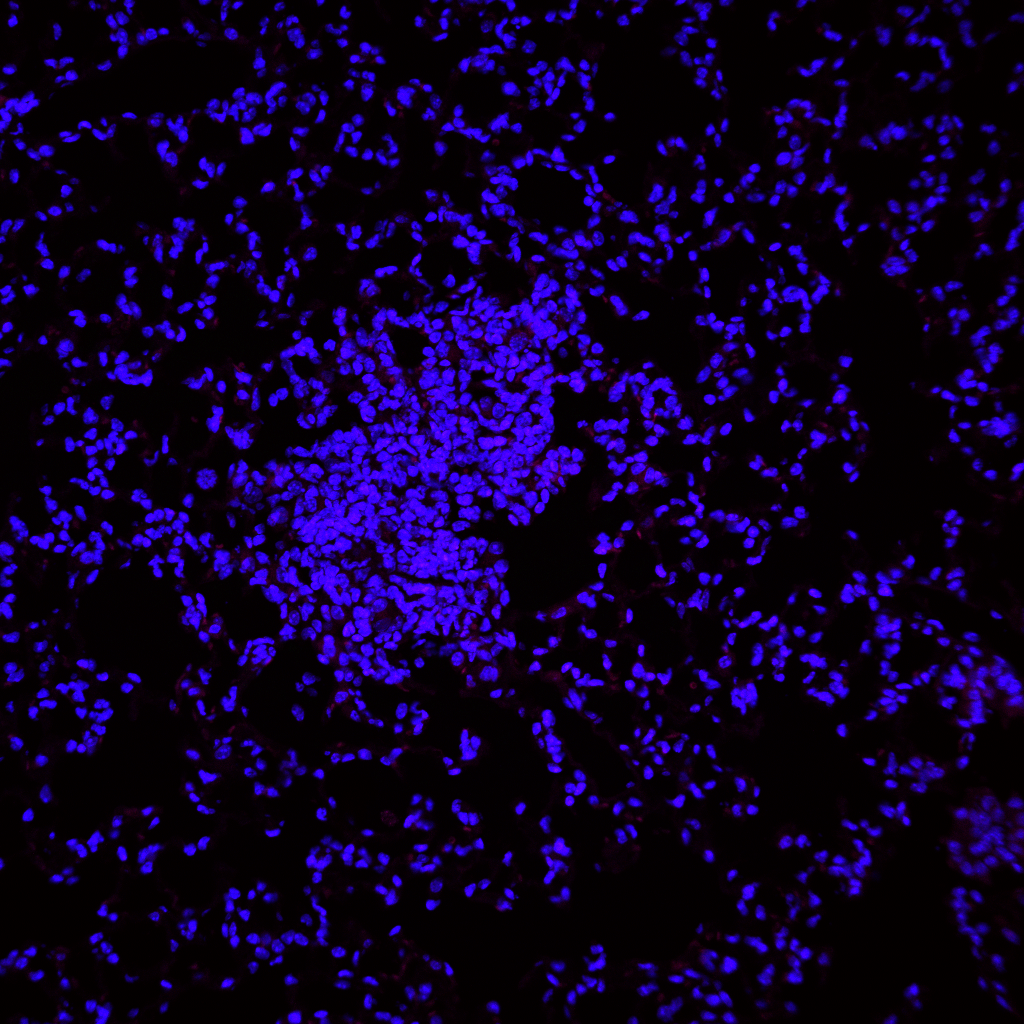

Supplement: Supplementary file 23 — Unprocessed images and western blots [file 43587_2024_776_MOESM23_ESM.zip › SD_ED_4_images/Ext_Fig_4_J_adenoma-Image Export-01_c1+2+3+4.tif]

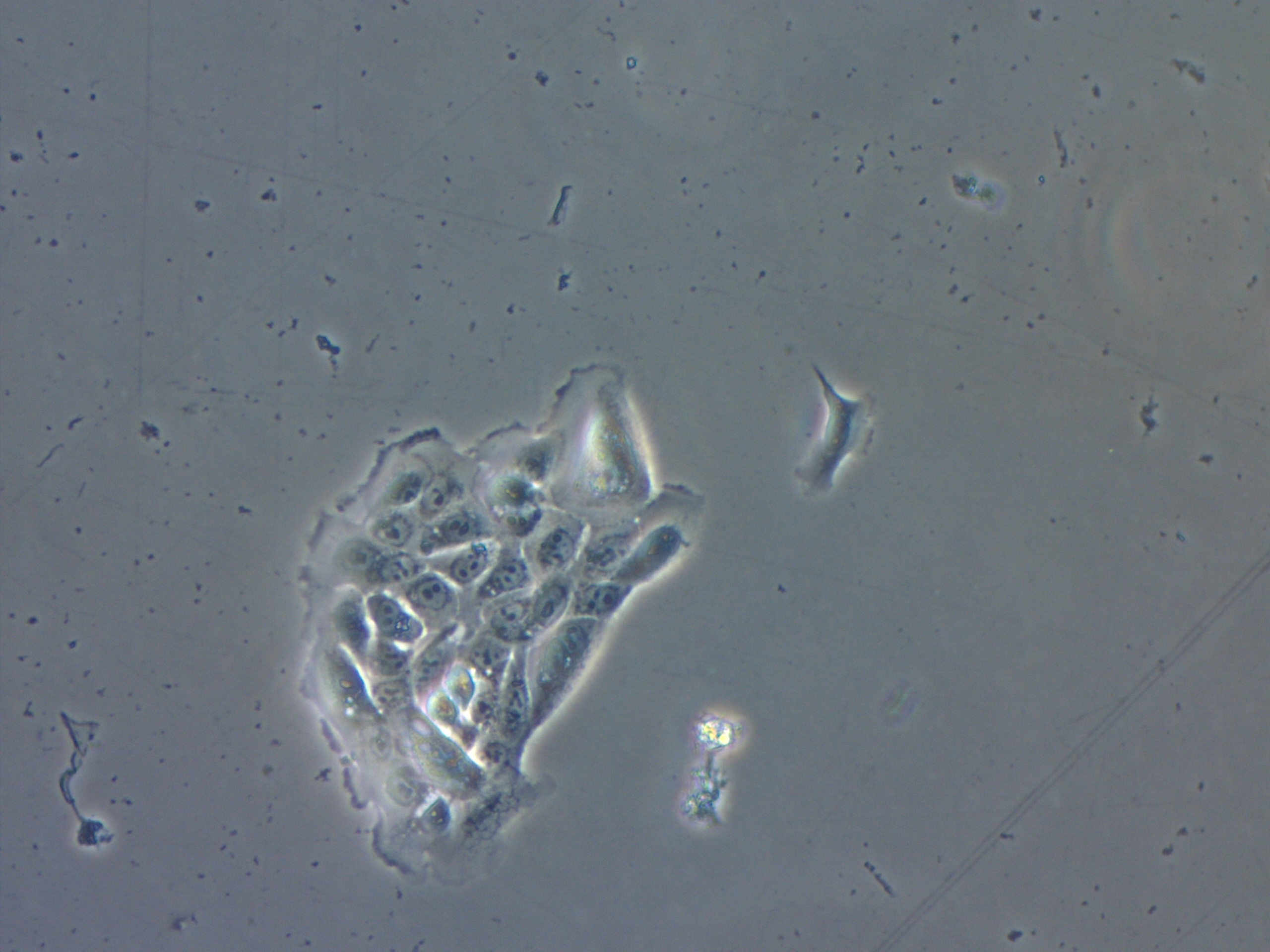

Supplement: Supplementary file 23 — Unprocessed images and western blots [file 43587_2024_776_MOESM23_ESM.zip › SD_ED_4_images/Ext_Fig_4_E_D5 No Dox.jpg]

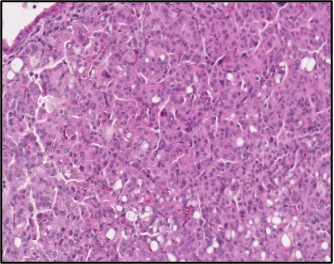

Supplement: Supplementary file 23 — Unprocessed images and western blots [file 43587_2024_776_MOESM23_ESM.zip › SD_ED_4_images/Ext_Fig_4_J_HE ADK.png]

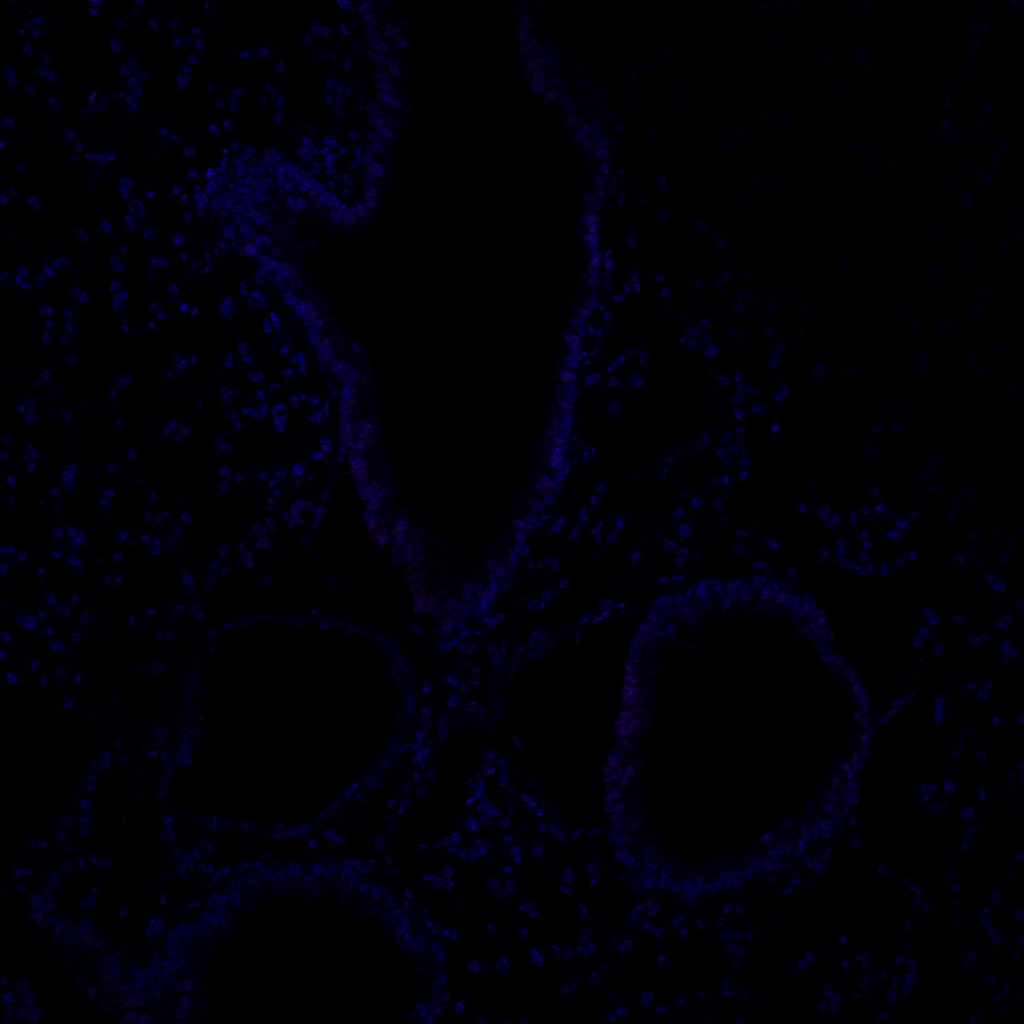

Supplement: Supplementary file 23 — Unprocessed images and western blots [file 43587_2024_776_MOESM23_ESM.zip › SD_ED_4_images/Ext_Fig_4_J_healthy gd3-Image Export-11_c1+2+3+4.tif]

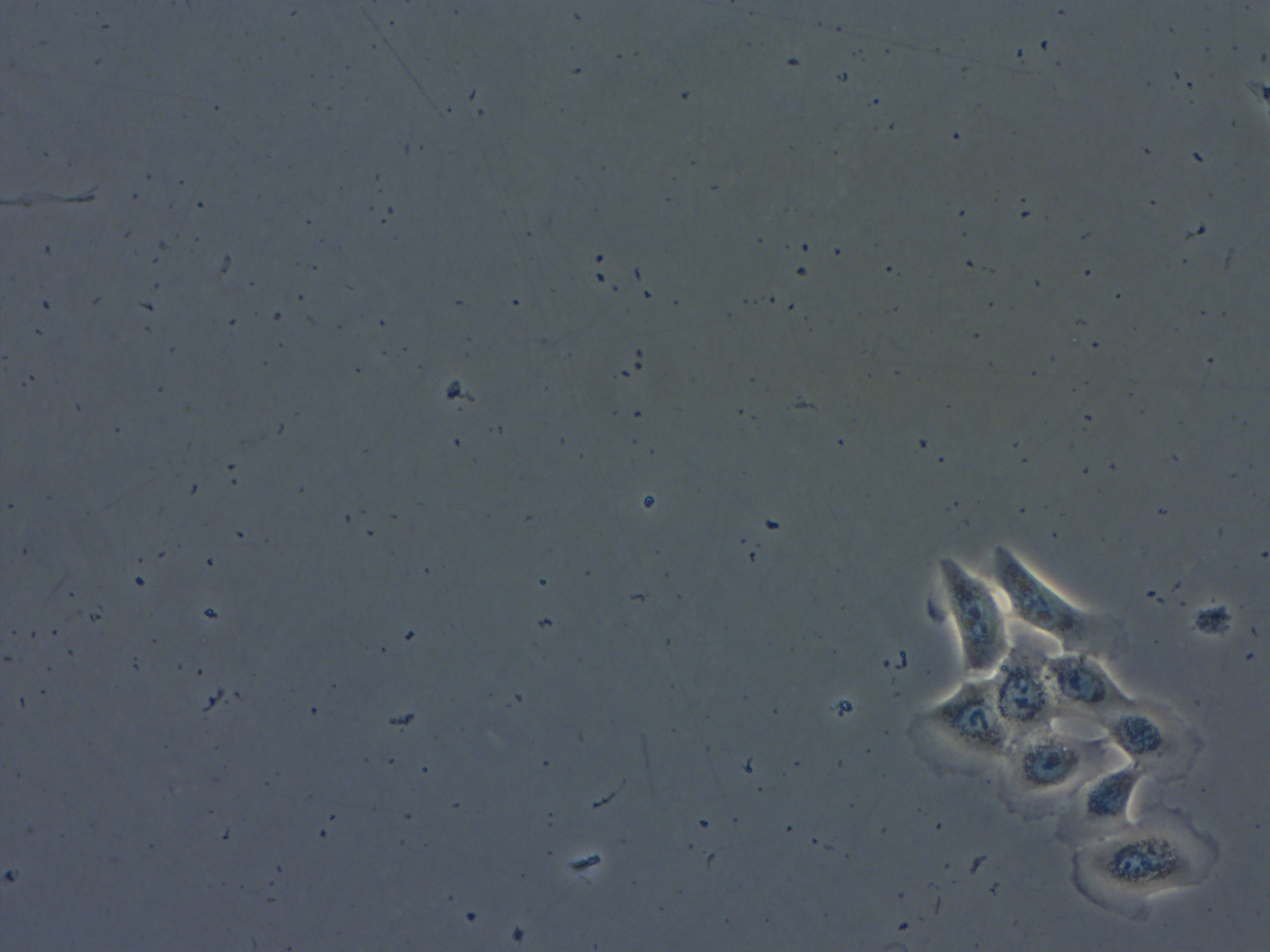

Supplement: Supplementary file 23 — Unprocessed images and western blots [file 43587_2024_776_MOESM23_ESM.zip › SD_ED_4_images/Ext_Fig_4_E_D2 No Dox.jpg]

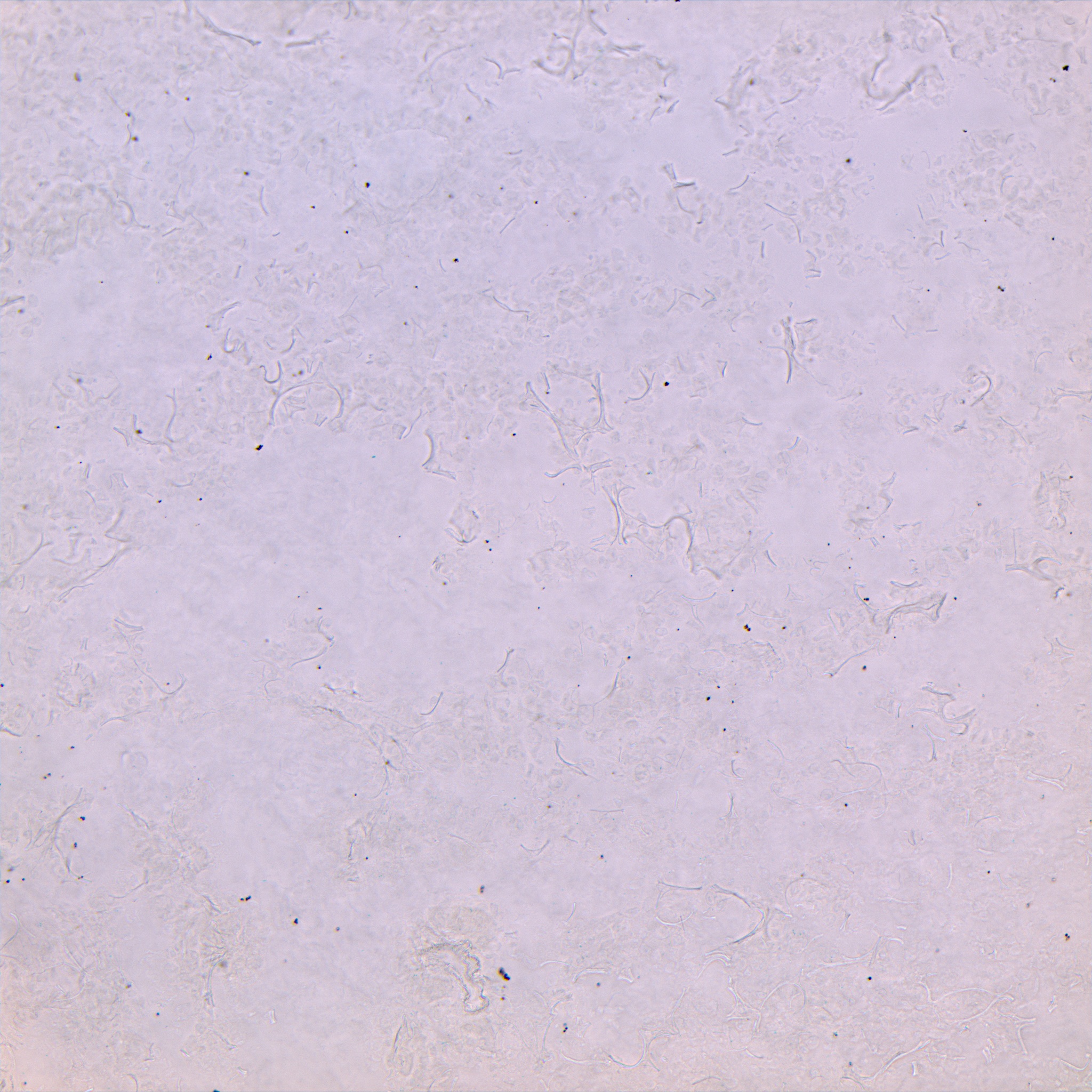

Supplement: Supplementary file 23 — Unprocessed images and western blots [file 43587_2024_776_MOESM23_ESM.zip › SD_ED_4_images/Ext_Fig_4_I_wt 20x tl 2-Image Export-61_c1+2+3.jpg]

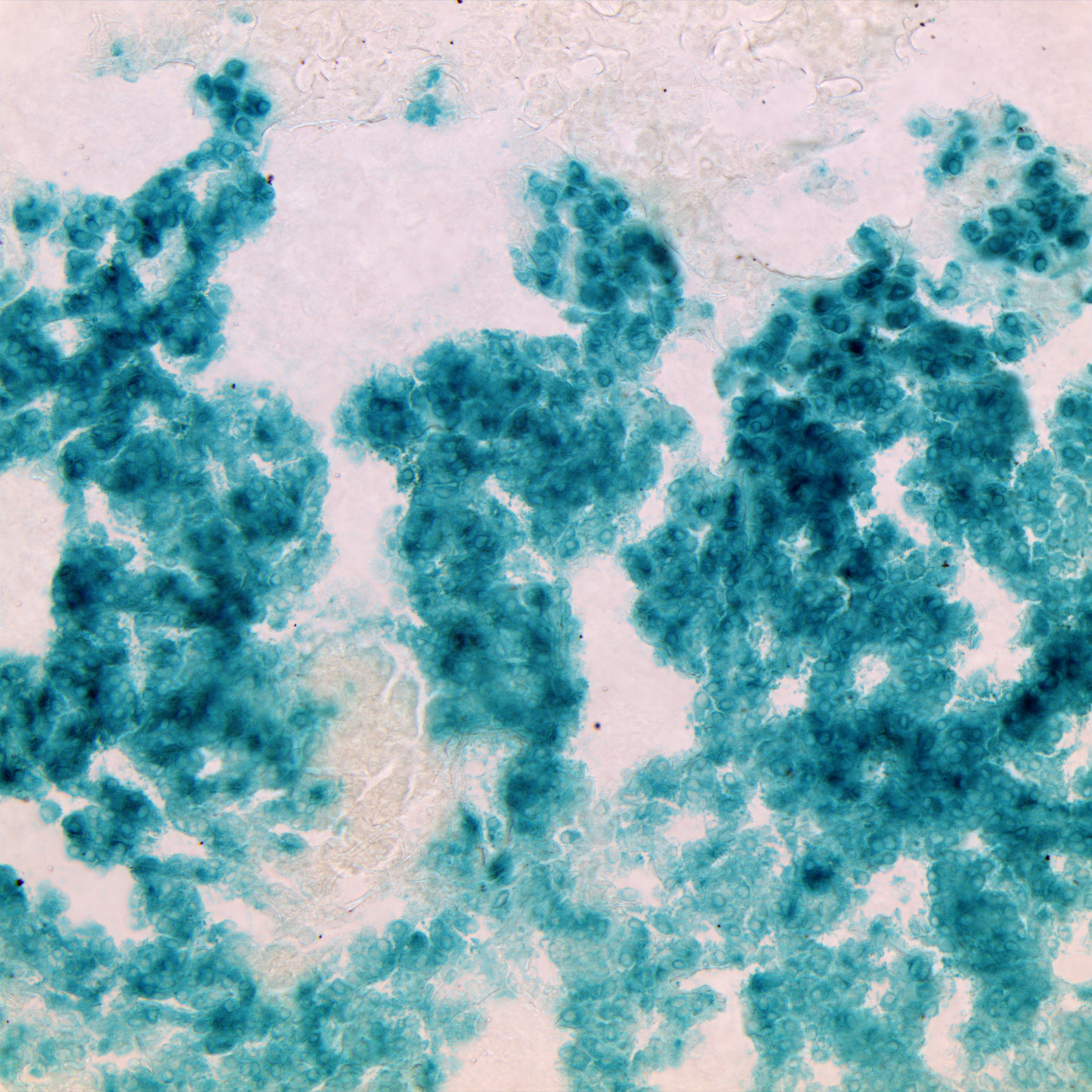

Supplement: Supplementary file 23 — Unprocessed images and western blots [file 43587_2024_776_MOESM23_ESM.zip › SD_ED_4_images/Ext_Fig_4_I_kras 20x tl 1-Image Export-53_c1+2+3.jpg]

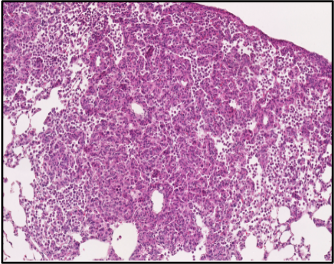

Supplement: Supplementary file 23 — Unprocessed images and western blots [file 43587_2024_776_MOESM23_ESM.zip › SD_ED_4_images/Ext_Fig_4_J_HE hyperplasia.png]

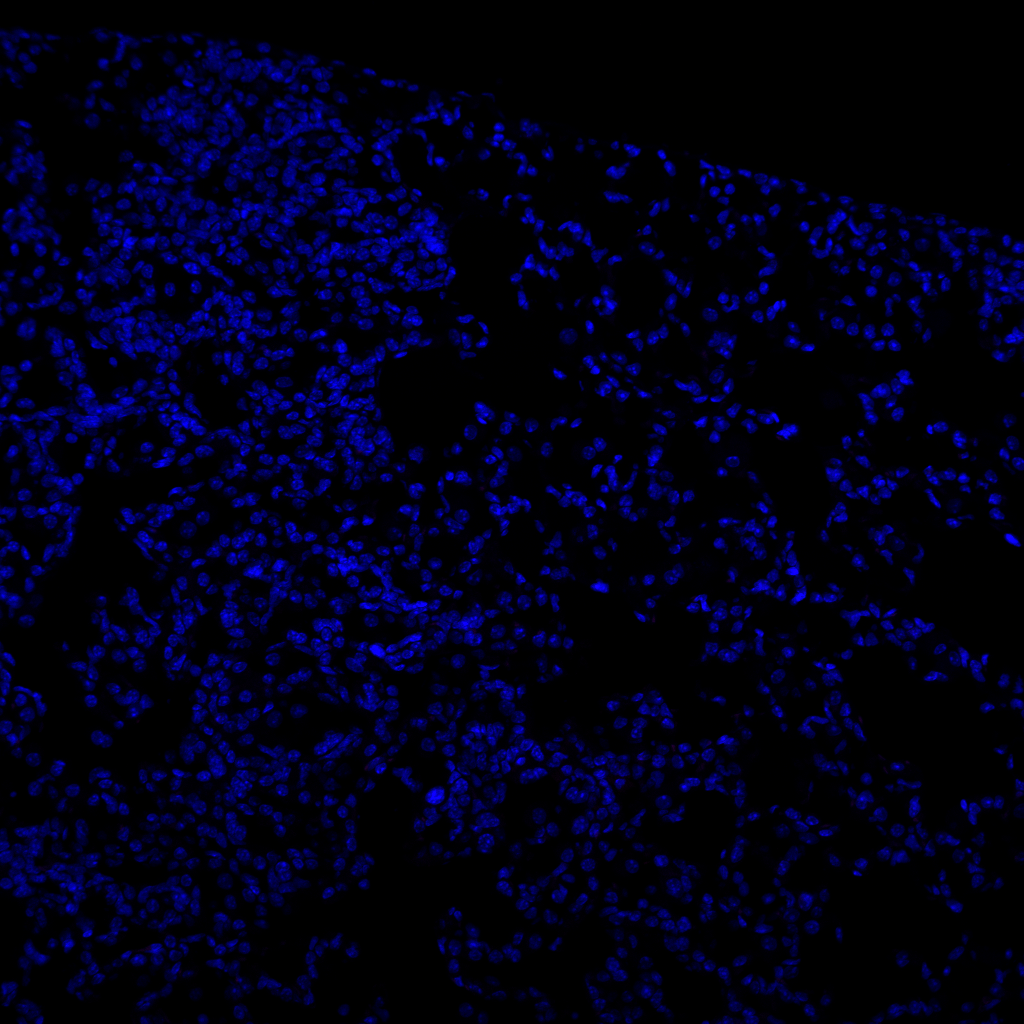

Supplement: Supplementary file 23 — Unprocessed images and western blots [file 43587_2024_776_MOESM23_ESM.zip › SD_ED_4_images/Ext_Fig_4_J_Hyperplasia 2-Image Export-09_c1+2+3+4.tif]

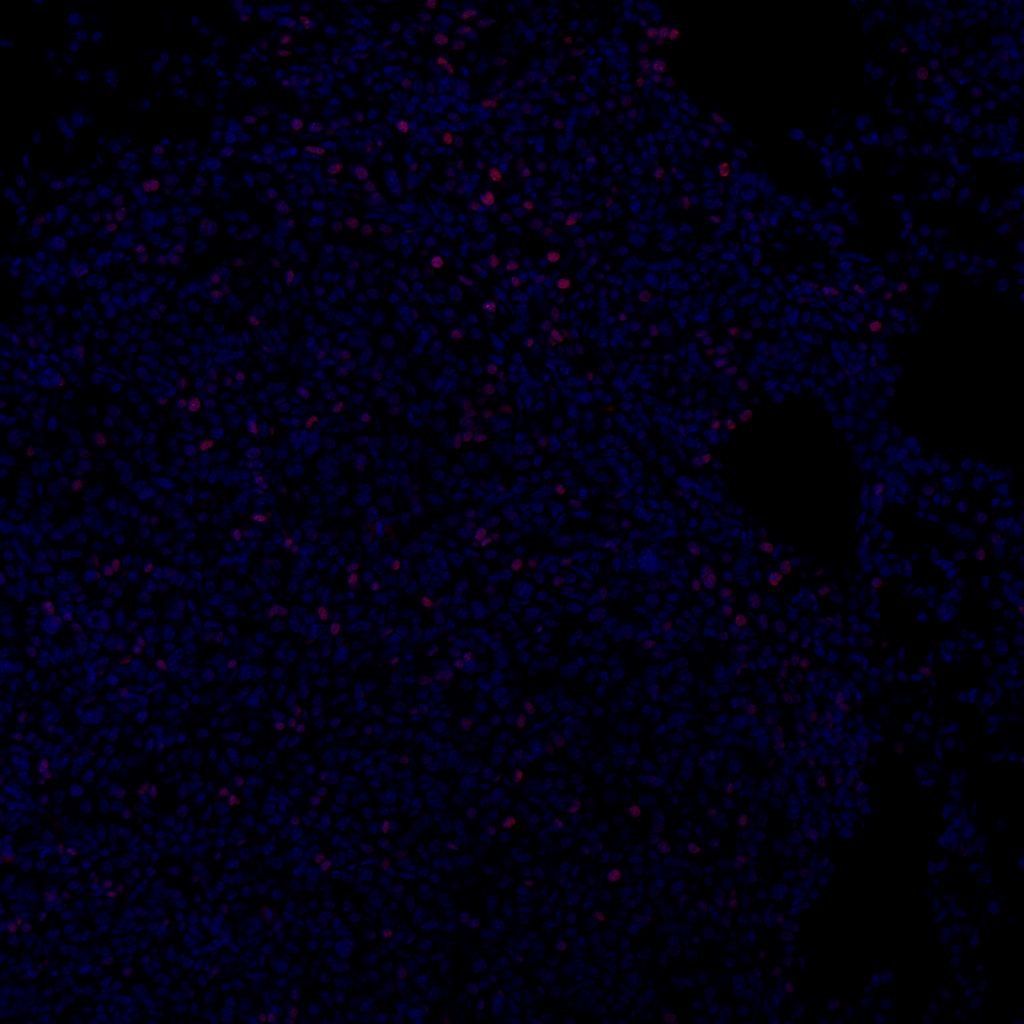

Supplement: Supplementary file 23 — Unprocessed images and western blots [file 43587_2024_776_MOESM23_ESM.zip › SD_ED_4_images/Ext_Fig_4_J_ADK 2-Image Export-49_c1+2+3+4.tif]

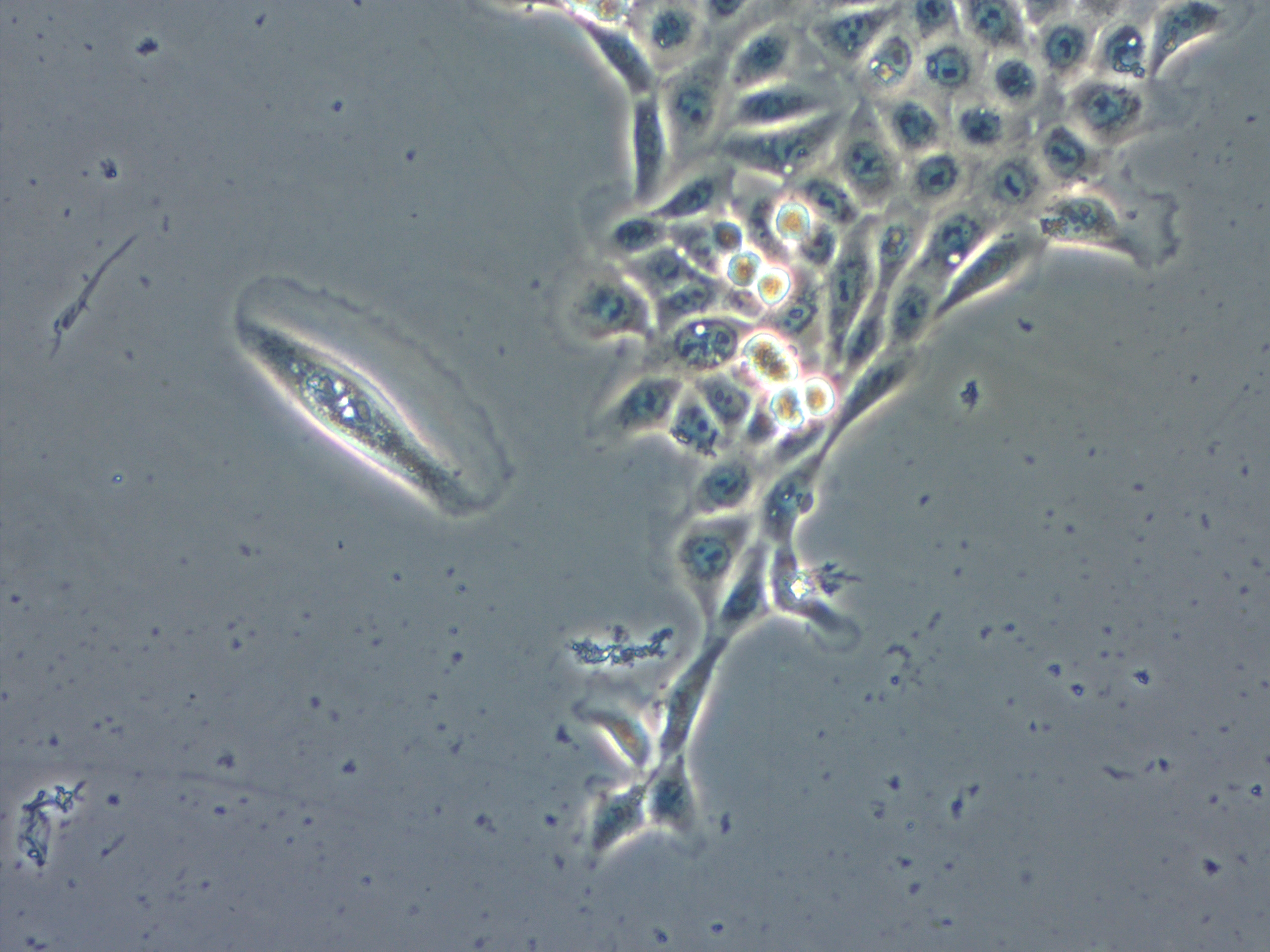

Supplement: Supplementary file 23 — Unprocessed images and western blots [file 43587_2024_776_MOESM23_ESM.zip › SD_ED_4_images/Ext_Fig_4_E_D4 No Dox.jpg]

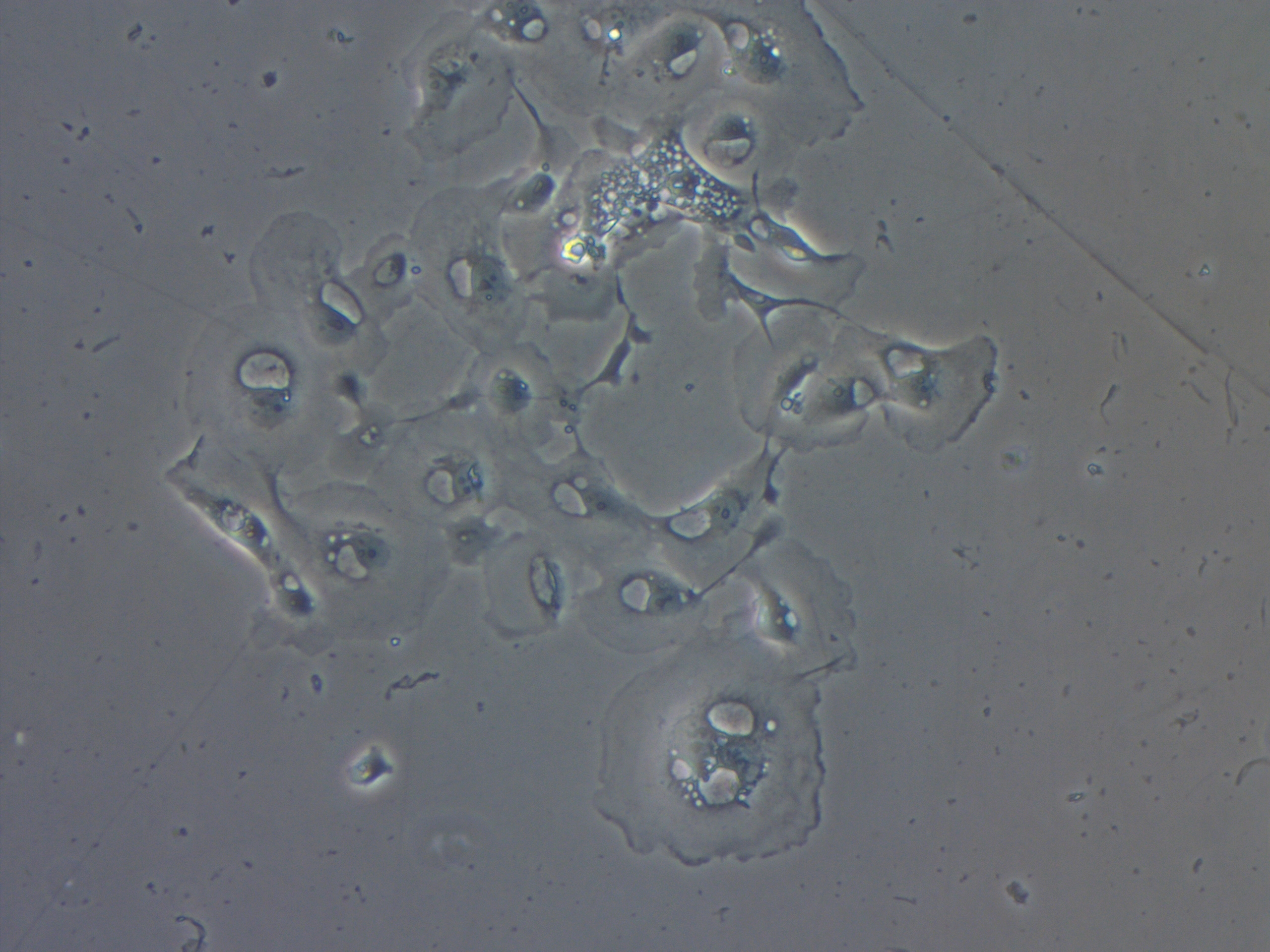

Supplement: Supplementary file 23 — Unprocessed images and western blots [file 43587_2024_776_MOESM23_ESM.zip › SD_ED_4_images/Ext_Fig_4_E_D4 +Dox.jpg]

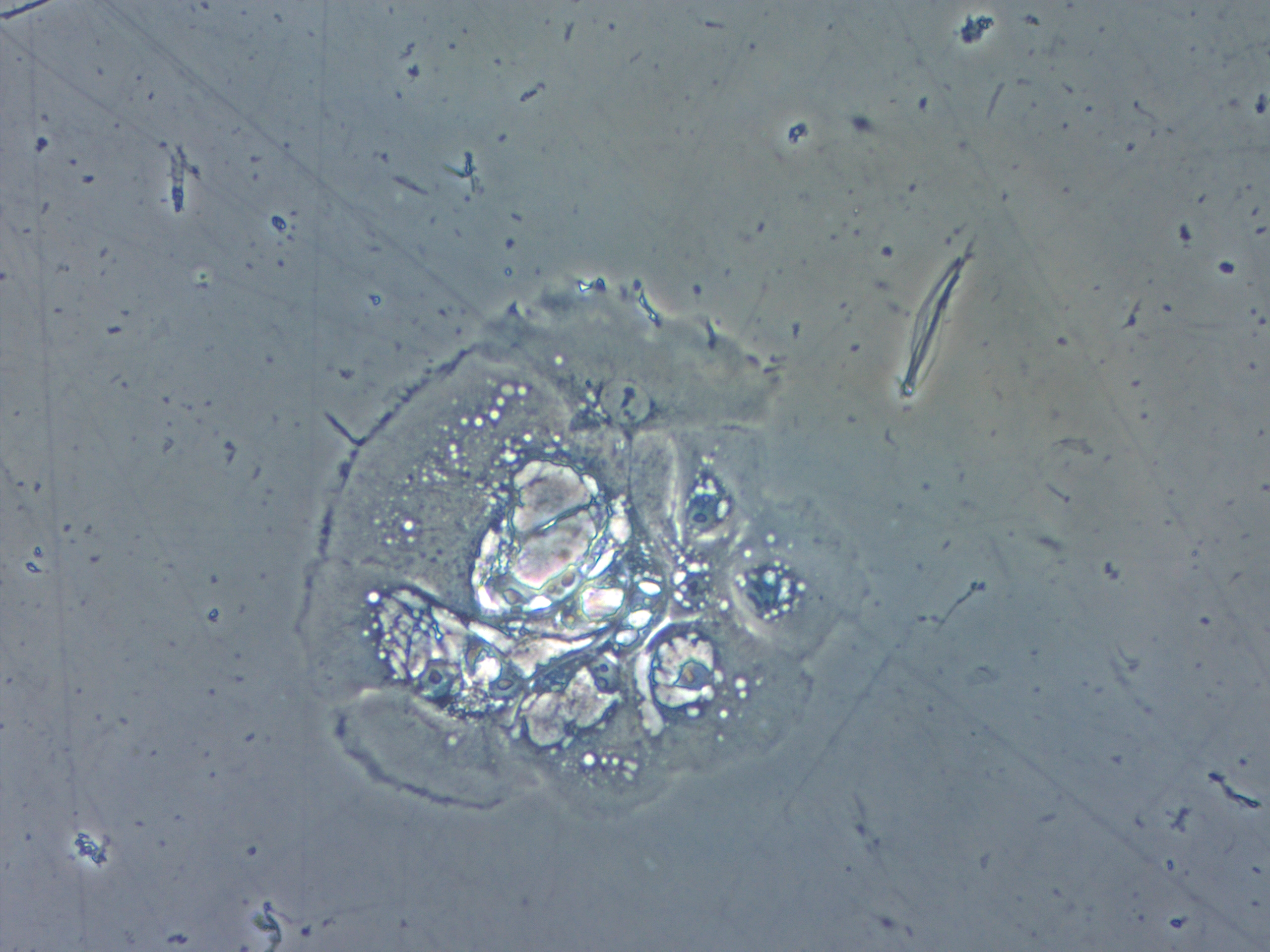

Supplement: Supplementary file 23 — Unprocessed images and western blots [file 43587_2024_776_MOESM23_ESM.zip › SD_ED_4_images/Ext_Fig_4_E_D5 +Dox.jpg]

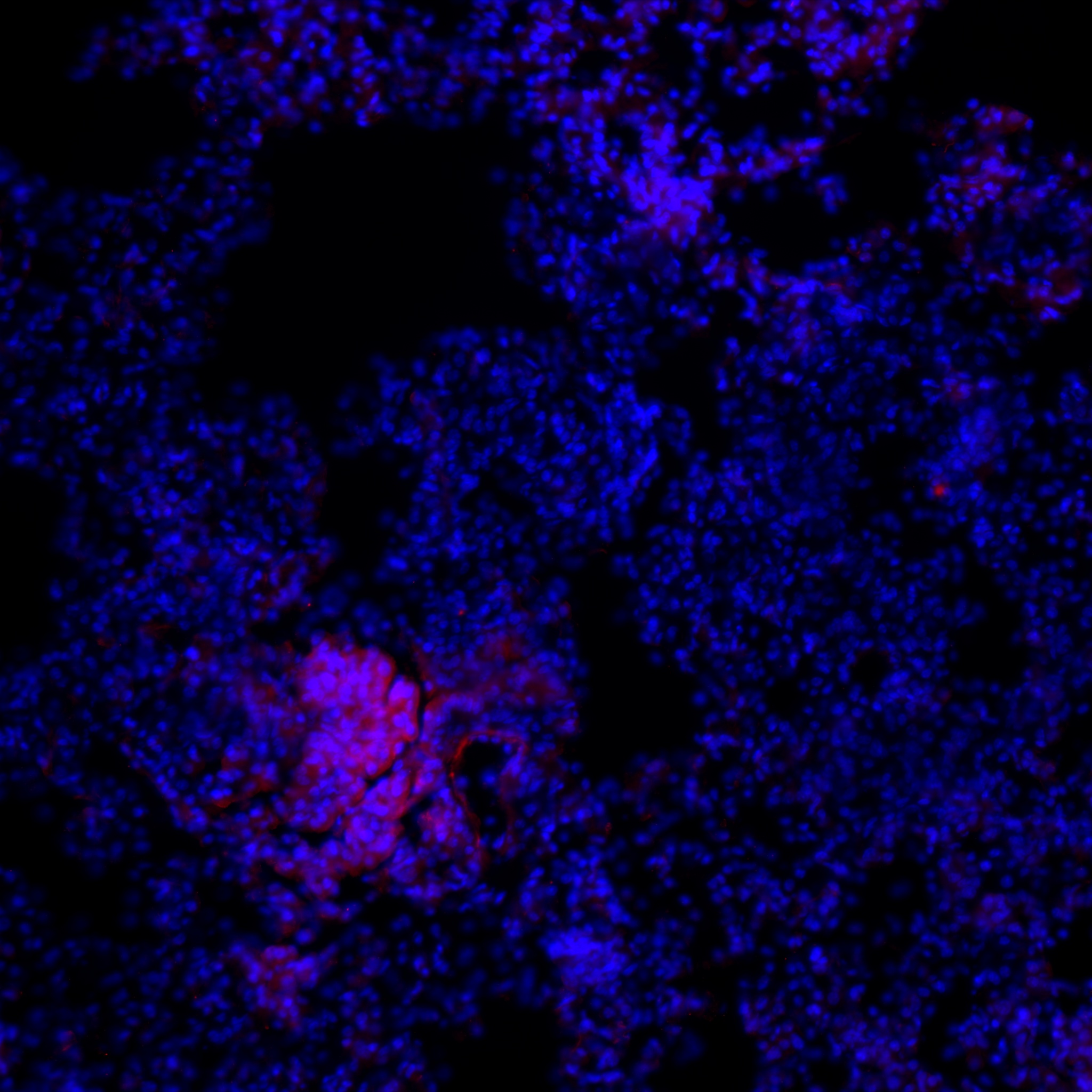

Supplement: Supplementary file 23 — Unprocessed images and western blots [file 43587_2024_776_MOESM23_ESM.zip › SD_ED_4_images/Ext_Fig_4_I_kras 20x if 1-Image Export-50_c1+2.jpg]

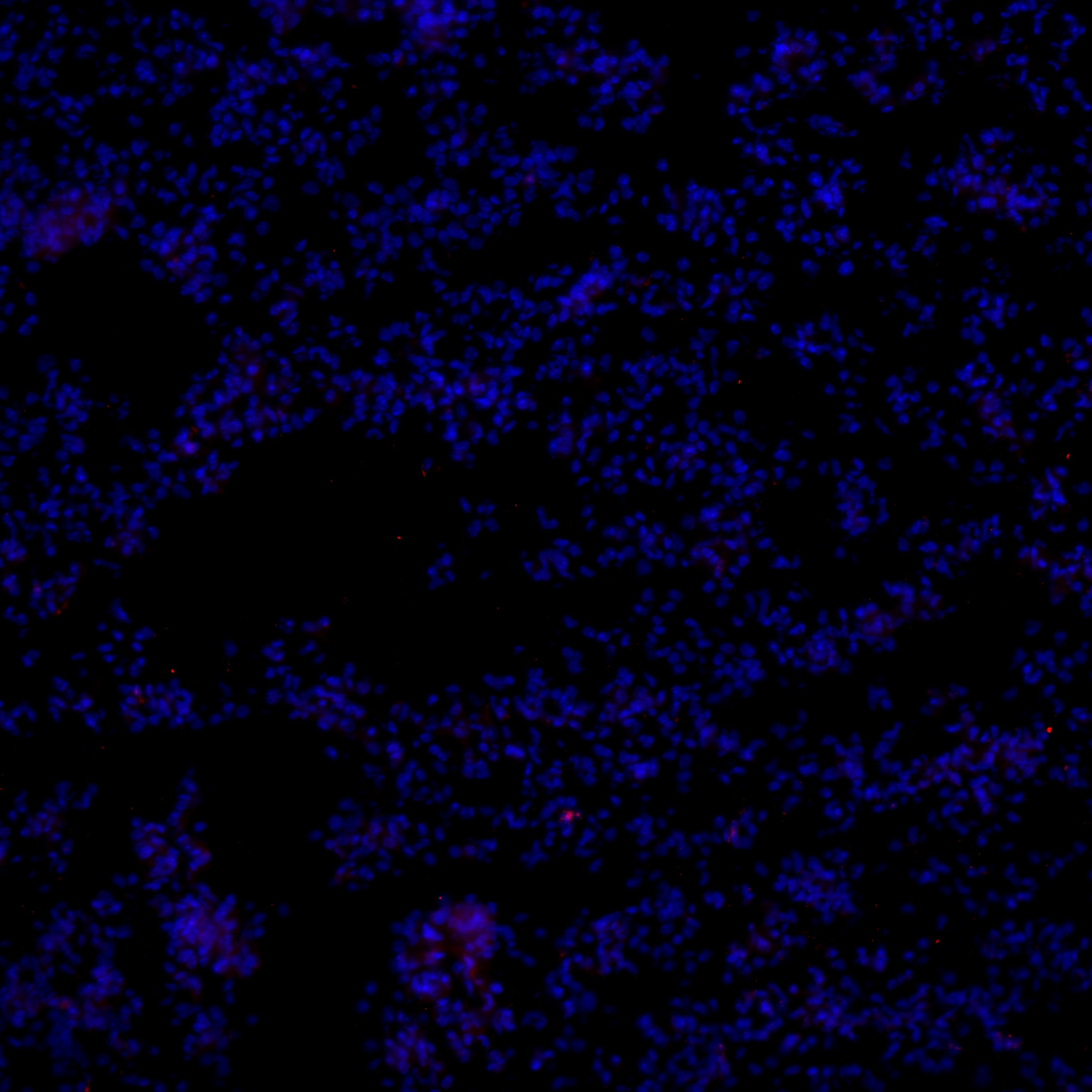

Supplement: Supplementary file 23 — Unprocessed images and western blots [file 43587_2024_776_MOESM23_ESM.zip › SD_ED_4_images/Ext_Fig_4_I_wt 20x if 2-Image Export-58_c1+2.jpg]

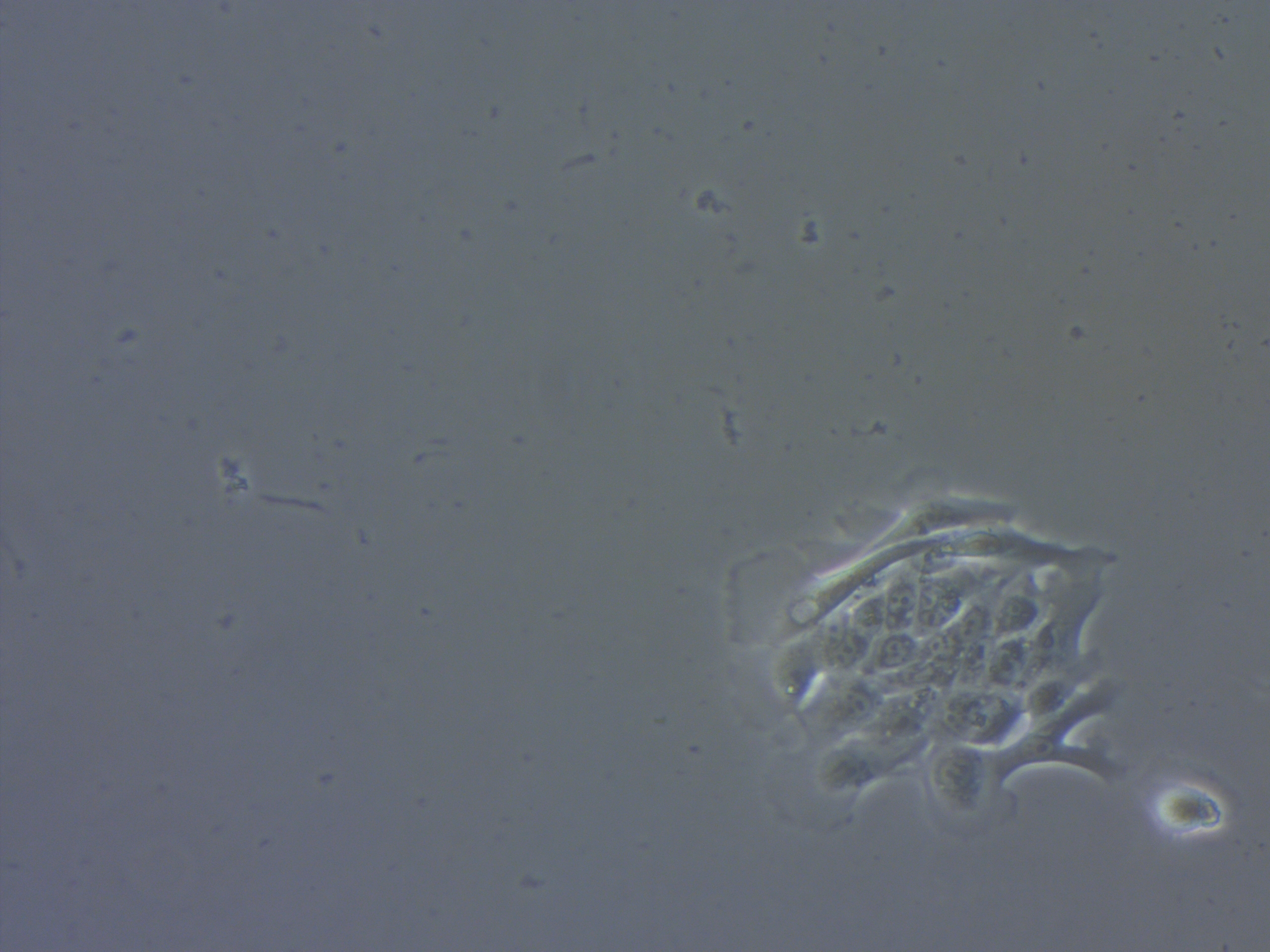

Supplement: Supplementary file 23 — Unprocessed images and western blots [file 43587_2024_776_MOESM23_ESM.zip › SD_ED_4_images/Ext_Fig_4_E_D3 No Dox.jpg]

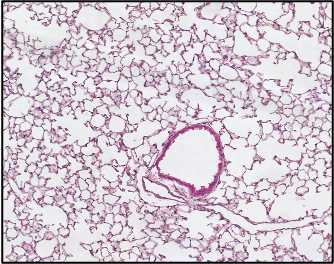

Supplement: Supplementary file 23 — Unprocessed images and western blots [file 43587_2024_776_MOESM23_ESM.zip › SD_ED_4_images/Ext_Fig_4_J_HE healthy.png]

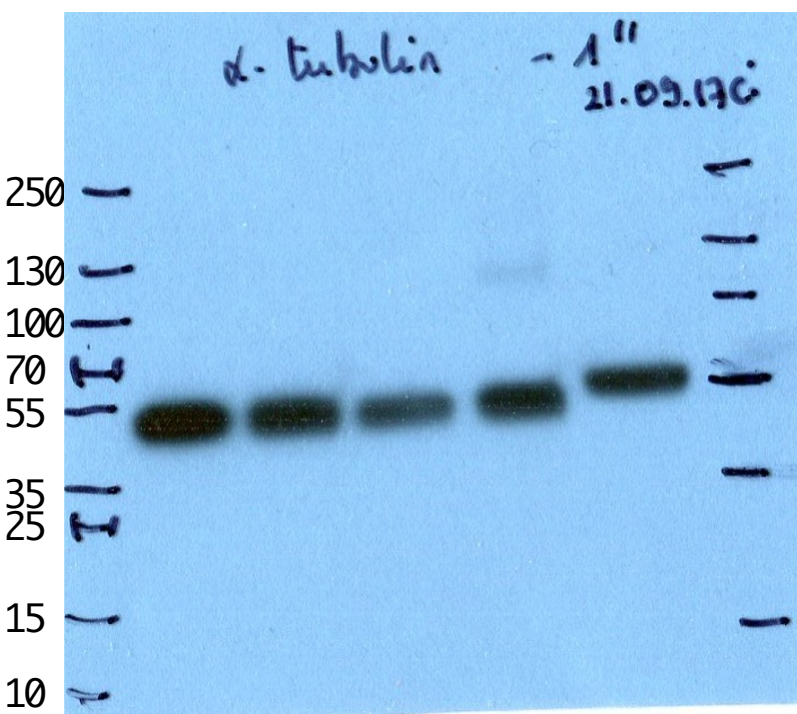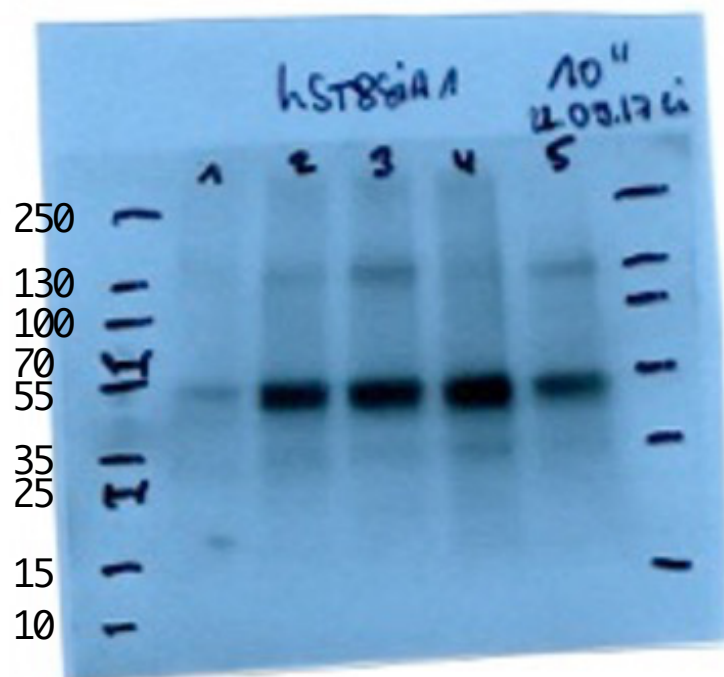

Supplement: Supplementary file 23 — Unprocessed images and western blots [file 43587_2024_776_MOESM23_ESM.zip › SD_ED_4_images/Ext_Data_4_D_Molecular_weight.pdf]

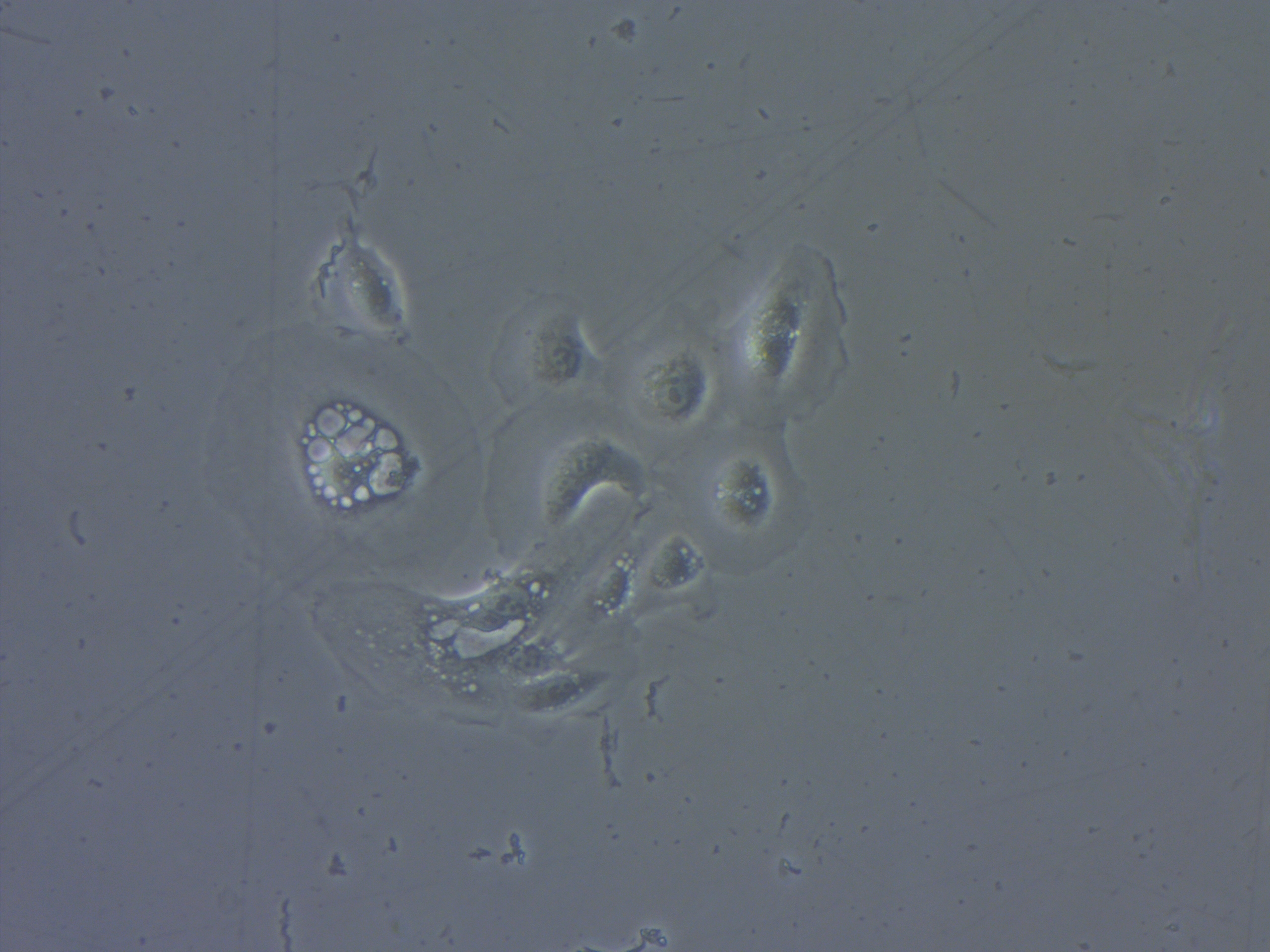

Supplement: Supplementary file 23 — Unprocessed images and western blots [file 43587_2024_776_MOESM23_ESM.zip › SD_ED_4_images/Ext_Fig_4_E_D3 +Dox.jpg]

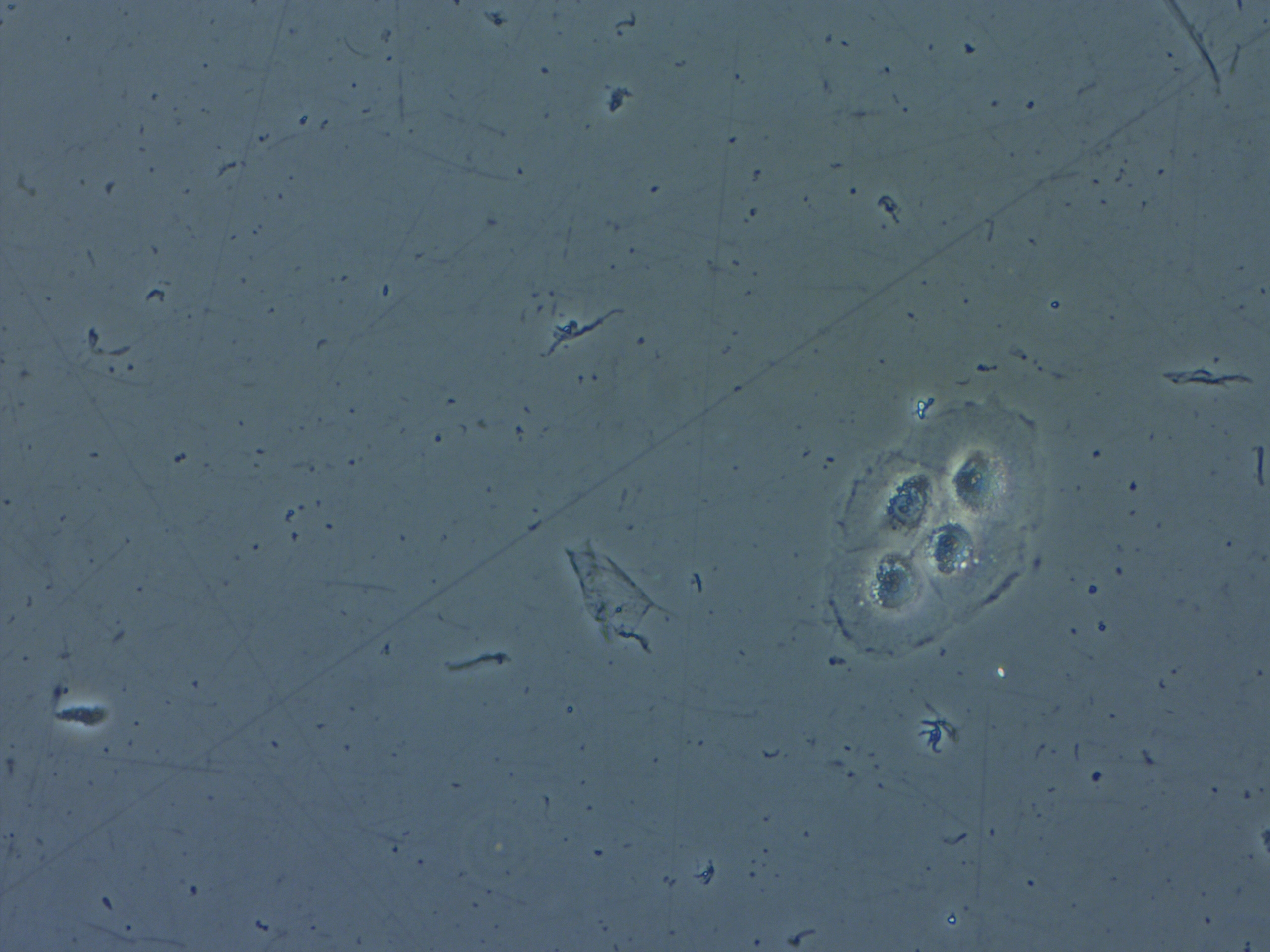

Supplement: Supplementary file 23 — Unprocessed images and western blots [file 43587_2024_776_MOESM23_ESM.zip › SD_ED_4_images/Ext_Fig_4_E_D2 +Dox.jpg]

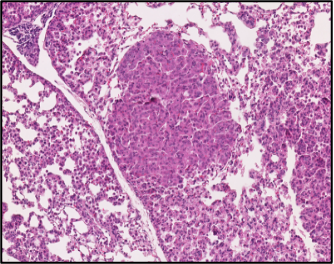

Supplement: Supplementary file 23 — Unprocessed images and western blots [file 43587_2024_776_MOESM23_ESM.zip › SD_ED_4_images/Ext_Fig_4_J_HE adenoma.png]

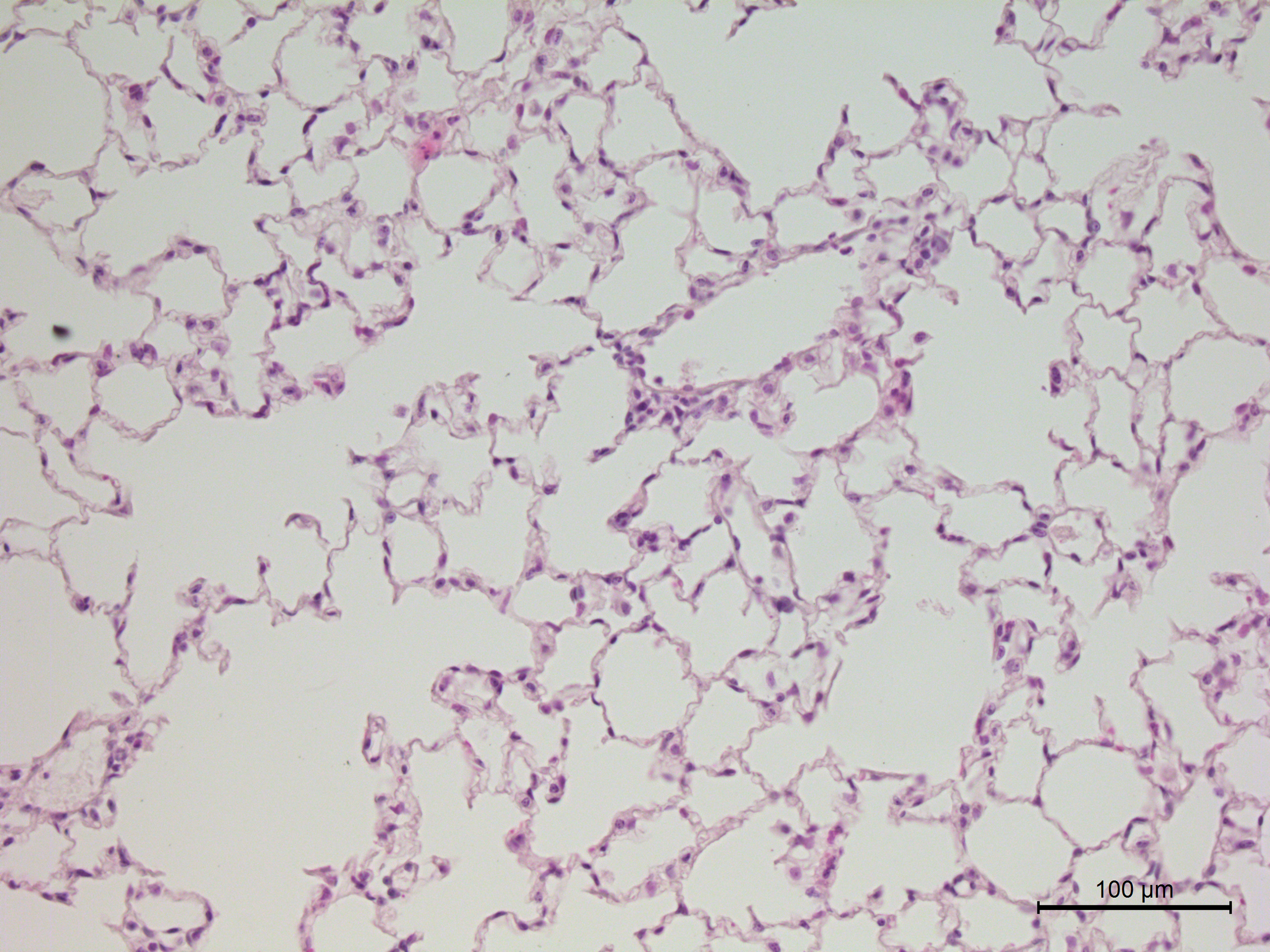

Supplement: Supplementary file 29 — Unprocessed images [file 43587_2024_776_MOESM29_ESM.zip › SD_ED_9_images/Ext_Data_9_D_G0_356_03_HE.tif]

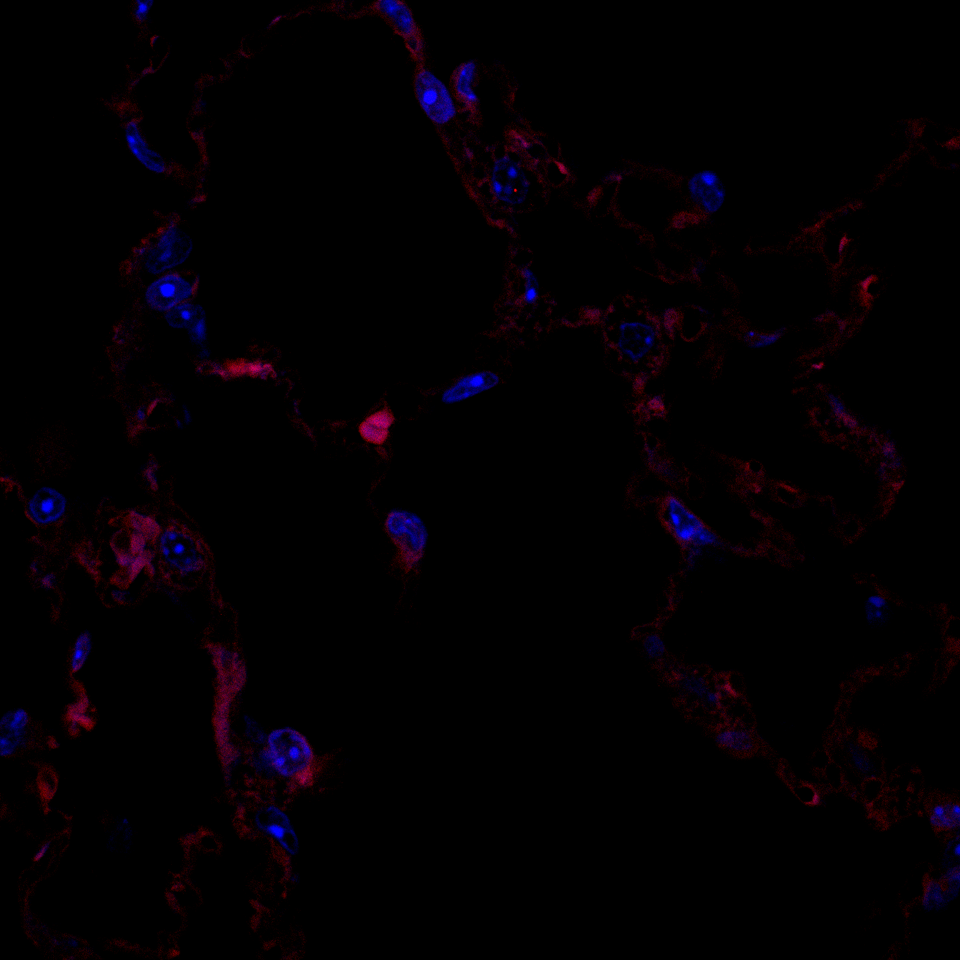

Supplement: Supplementary file 29 — Unprocessed images [file 43587_2024_776_MOESM29_ESM.zip › SD_ED_9_images/Ext_Data_Fig_9_D_KO_G0_GD3.tif]

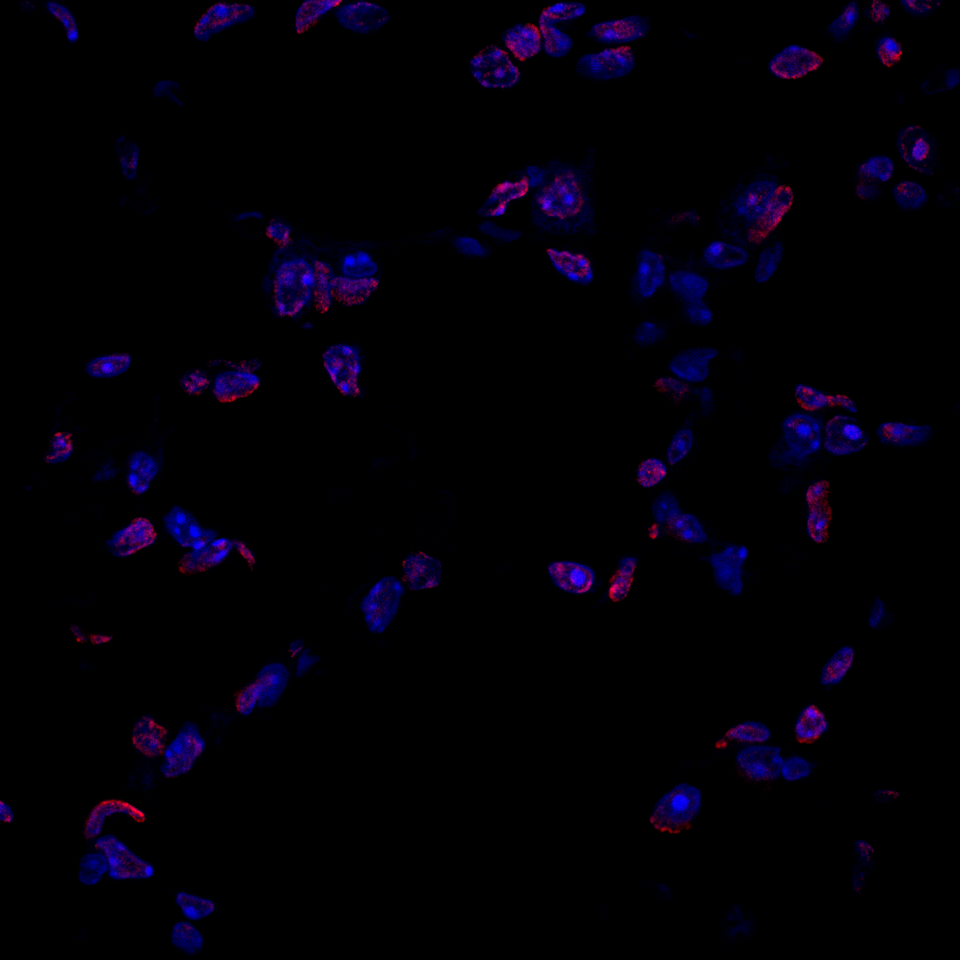

Supplement: Supplementary file 29 — Unprocessed images [file 43587_2024_776_MOESM29_ESM.zip › SD_ED_9_images/Ext_Data_Fig_9_D_WT_24mo_GD3.tif]

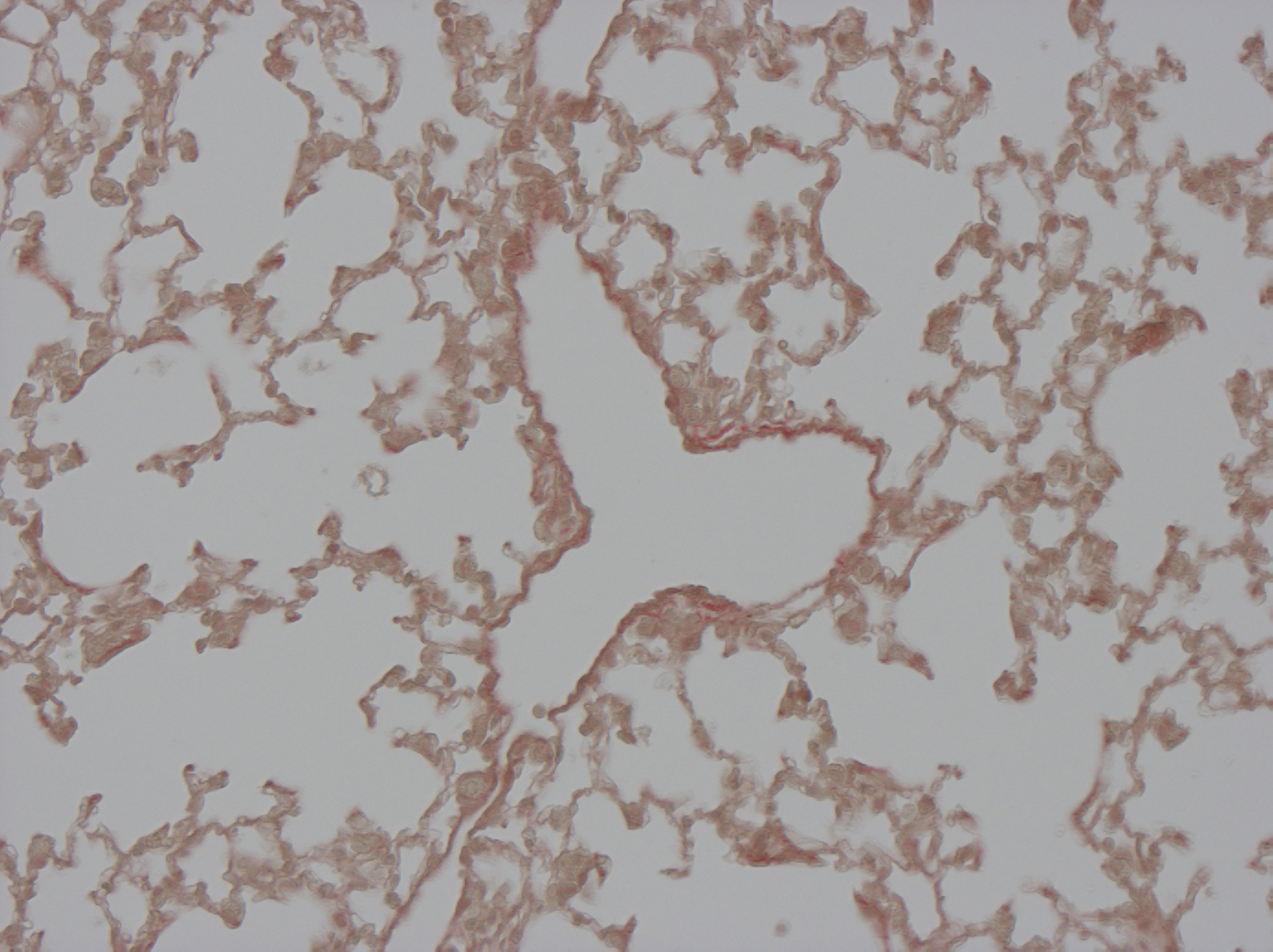

Supplement: Supplementary file 29 — Unprocessed images [file 43587_2024_776_MOESM29_ESM.zip › SD_ED_9_images/Ext_Data_9_SR_BF_3mo.tif]

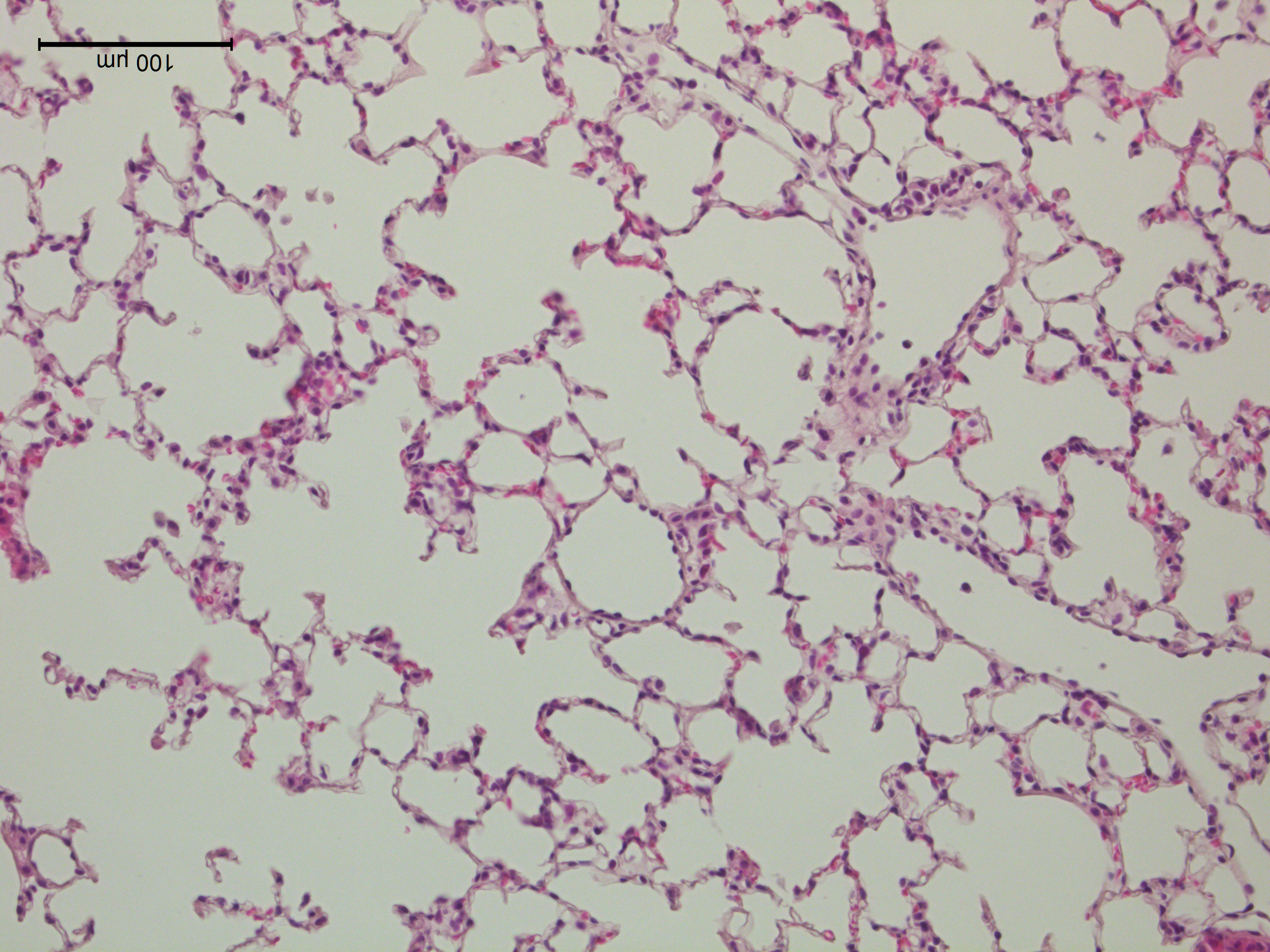

Supplement: Supplementary file 29 — Unprocessed images [file 43587_2024_776_MOESM29_ESM.zip › SD_ED_9_images/Ext_Data_9_HE_3mo.tif]

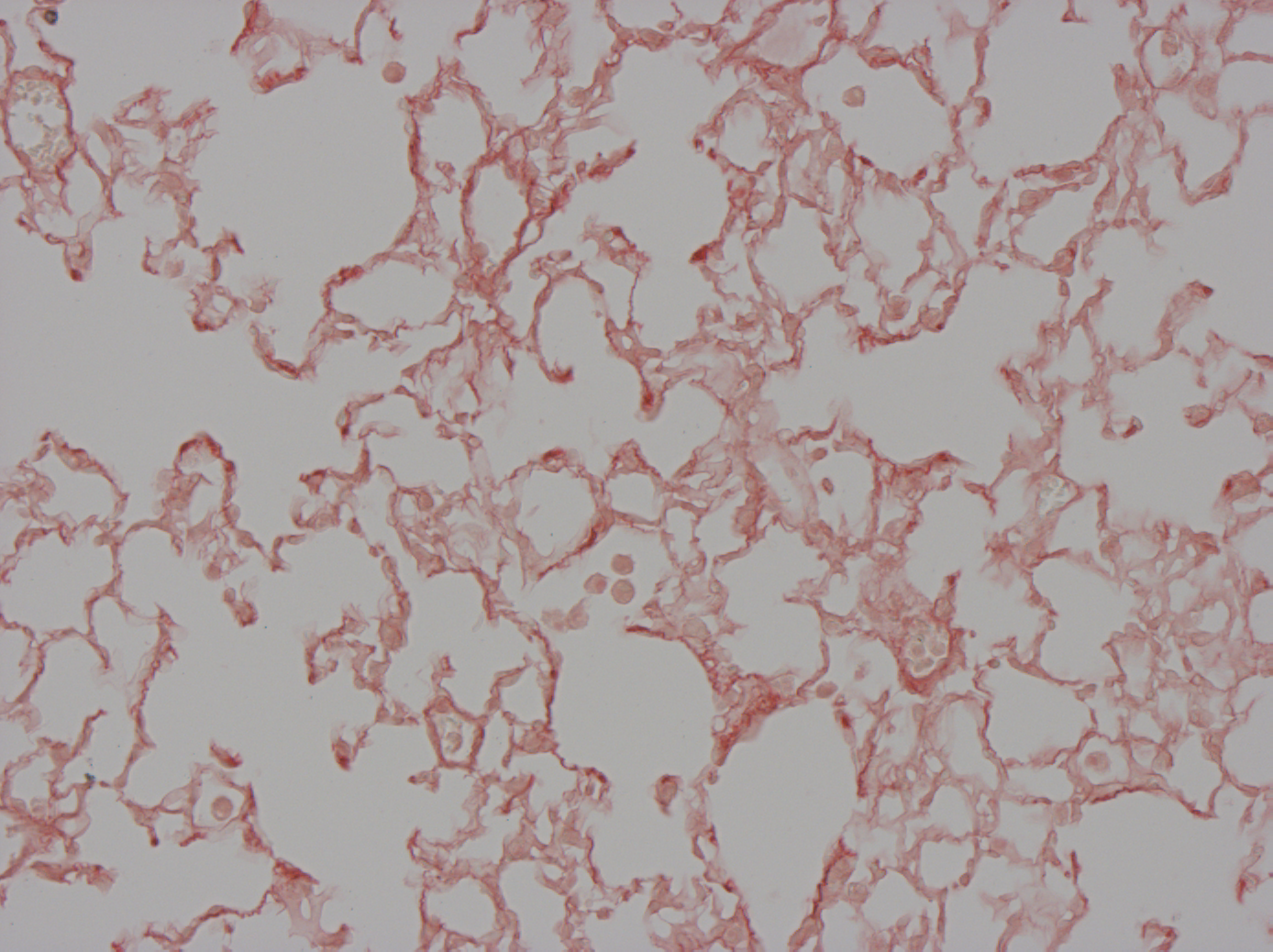

Supplement: Supplementary file 29 — Unprocessed images [file 43587_2024_776_MOESM29_ESM.zip › SD_ED_9_images/Ext_Data_9_D_G4_SR_BF.tif]

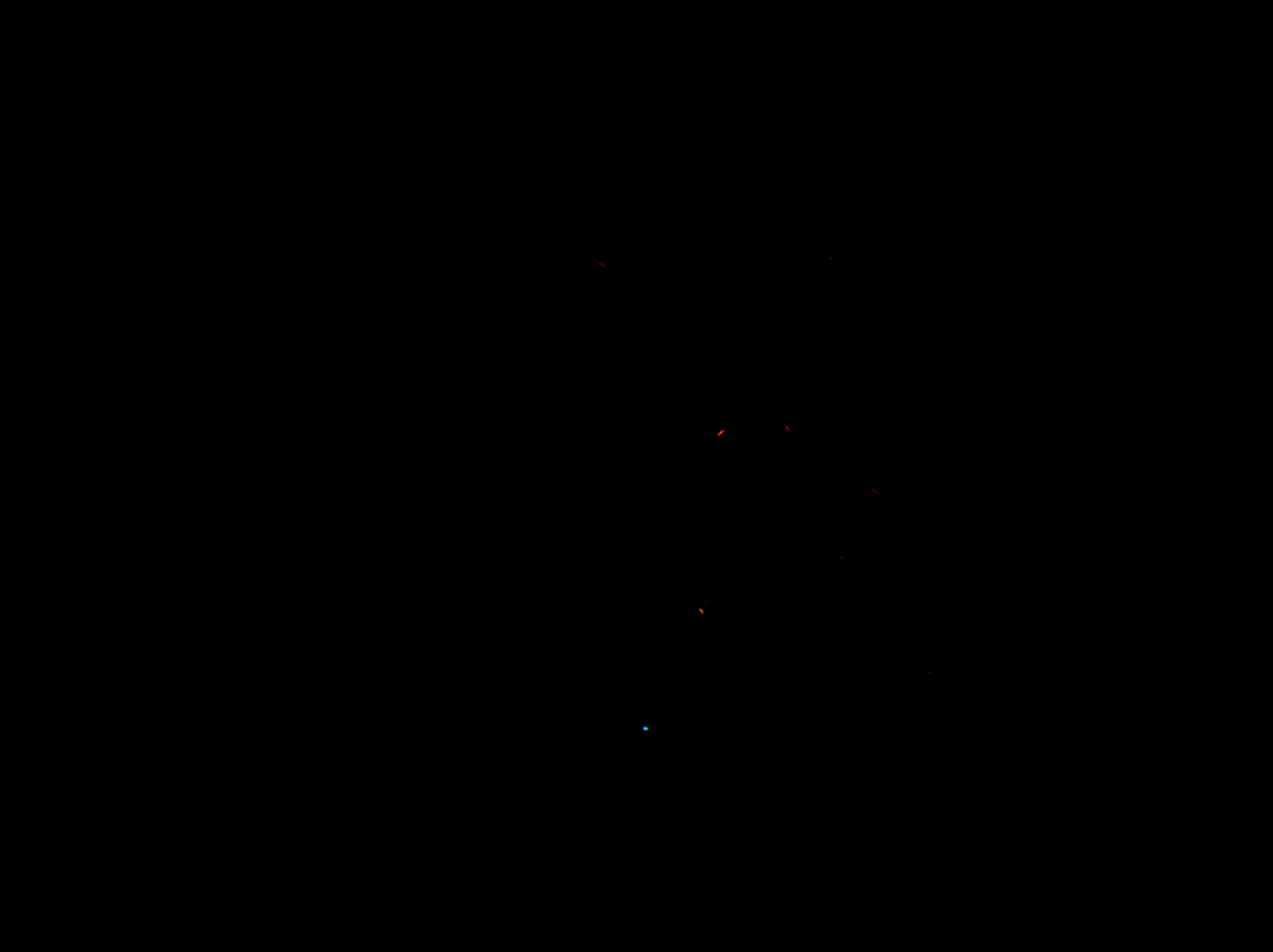

Supplement: Supplementary file 29 — Unprocessed images [file 43587_2024_776_MOESM29_ESM.zip › SD_ED_9_images/Ext_Data_9_SR_PL_3mo.tif]

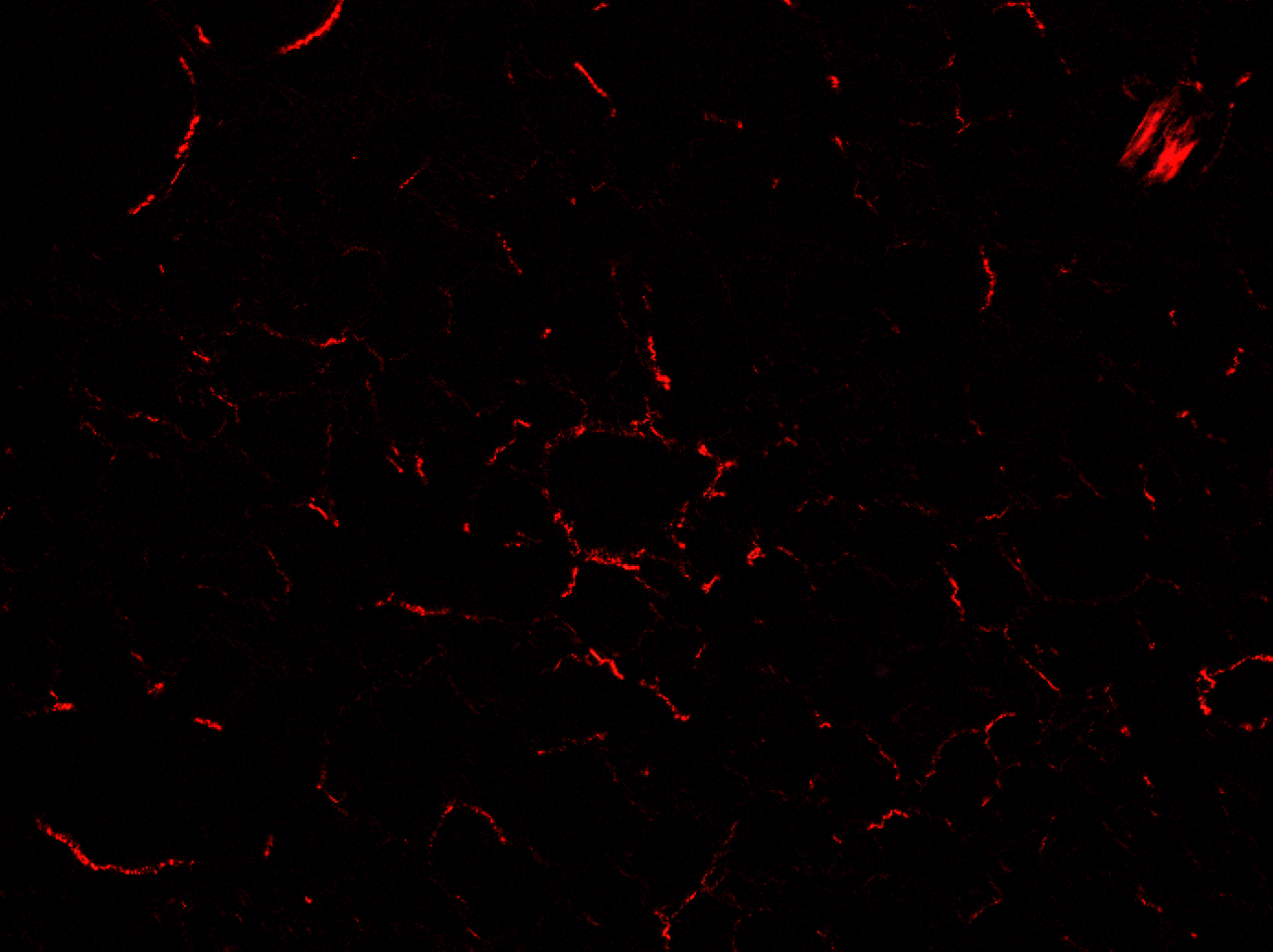

Supplement: Supplementary file 29 — Unprocessed images [file 43587_2024_776_MOESM29_ESM.zip › SD_ED_9_images/Ext_Data_9_D_G0_SR_PL.tif]

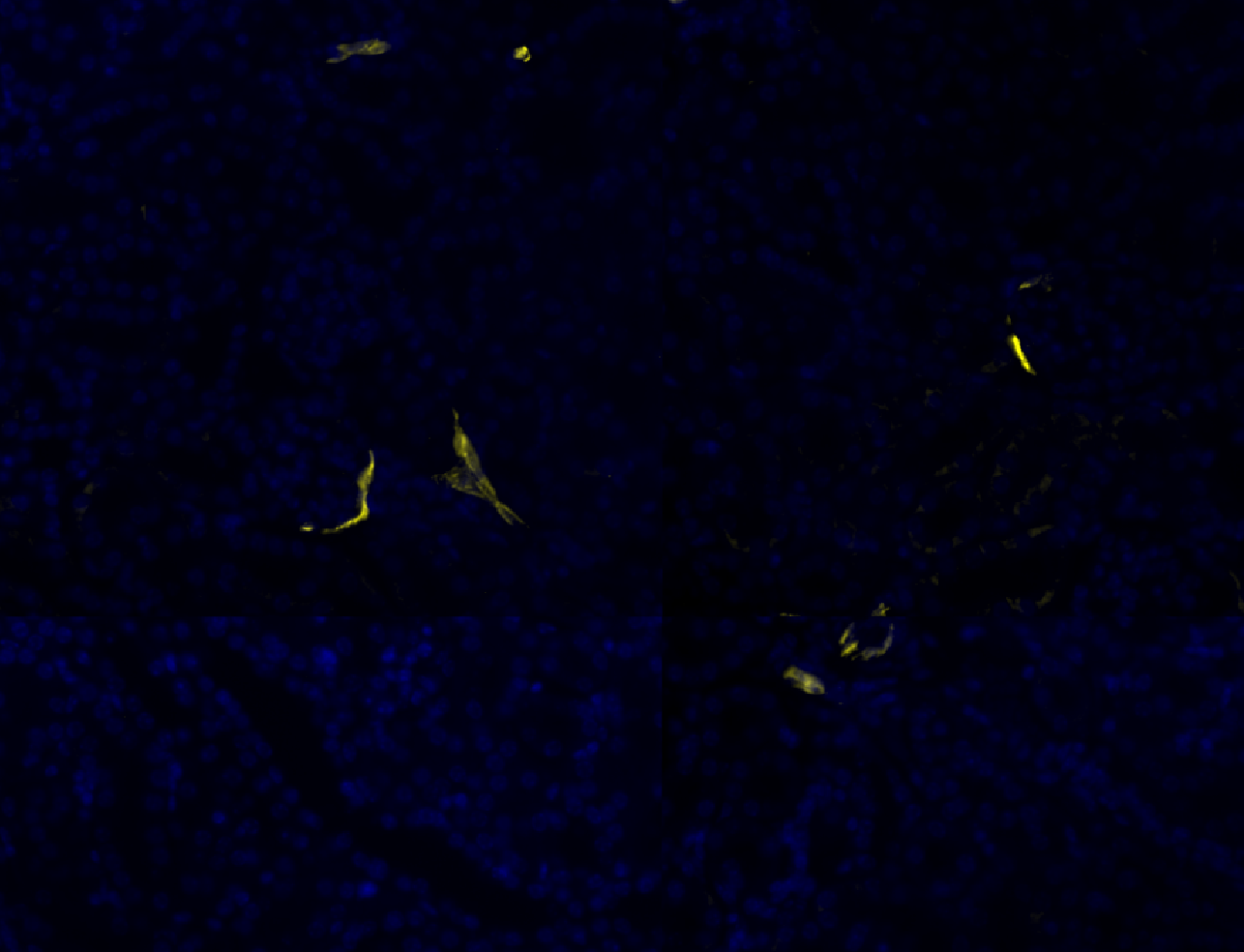

Supplement: Supplementary file 29 — Unprocessed images [file 43587_2024_776_MOESM29_ESM.zip › SD_ED_9_images/Ext_Fig_9_B_Young_DAPI + GD3.tif]

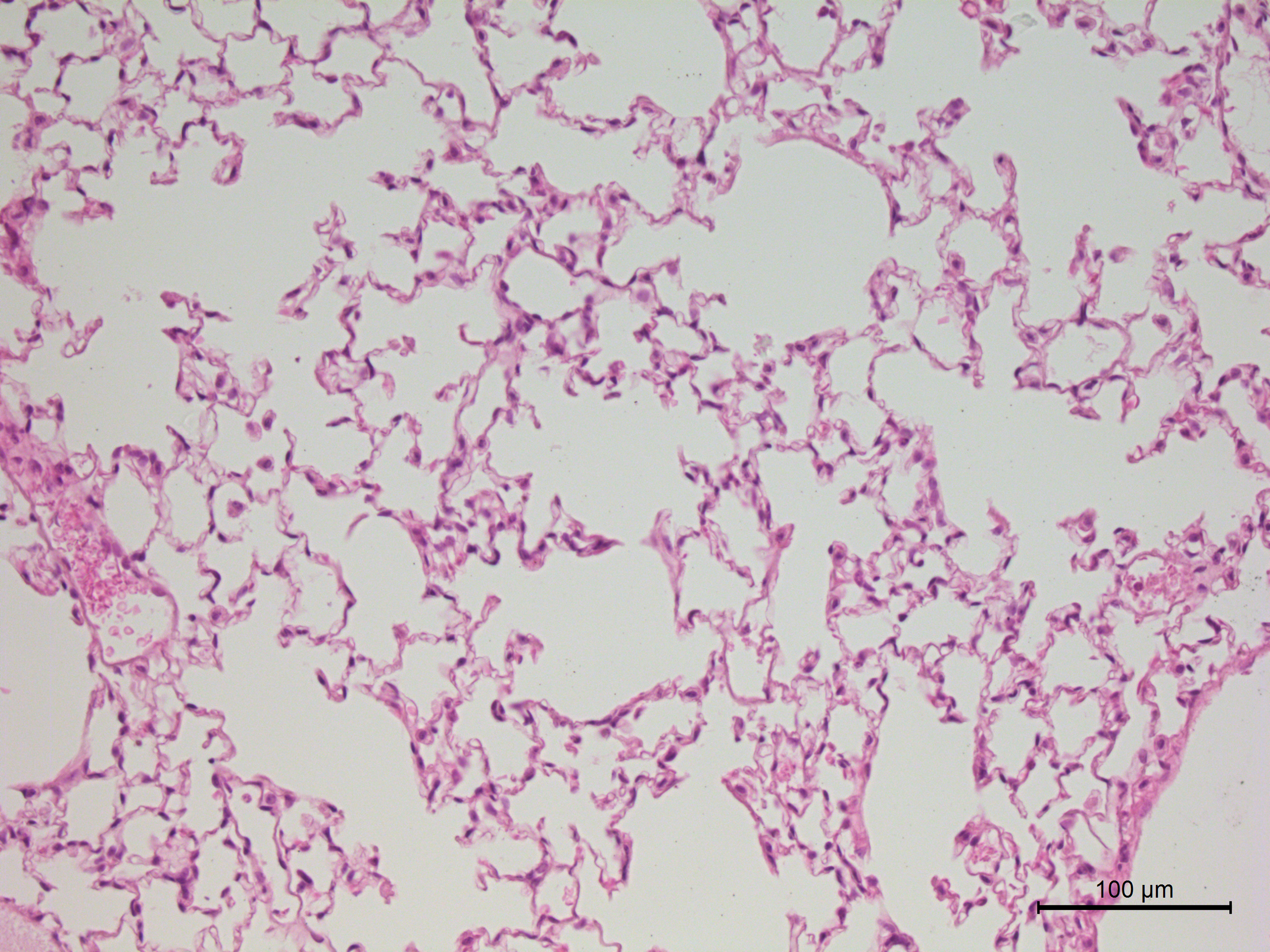

Supplement: Supplementary file 29 — Unprocessed images [file 43587_2024_776_MOESM29_ESM.zip › SD_ED_9_images/Ext_Data_9_D_G4_256_04_HE.tif]

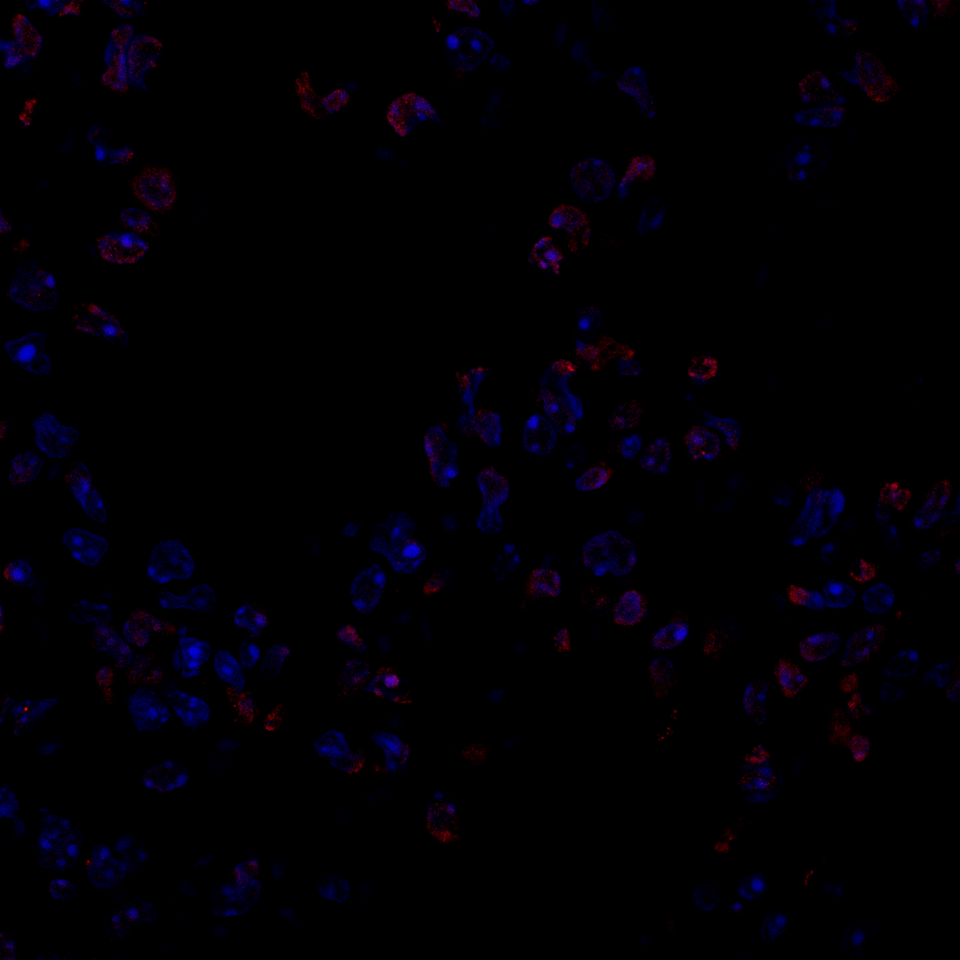

Supplement: Supplementary file 29 — Unprocessed images [file 43587_2024_776_MOESM29_ESM.zip › SD_ED_9_images/Ext_Data_Fig_9_D_WT_3mo_GD3.tif]

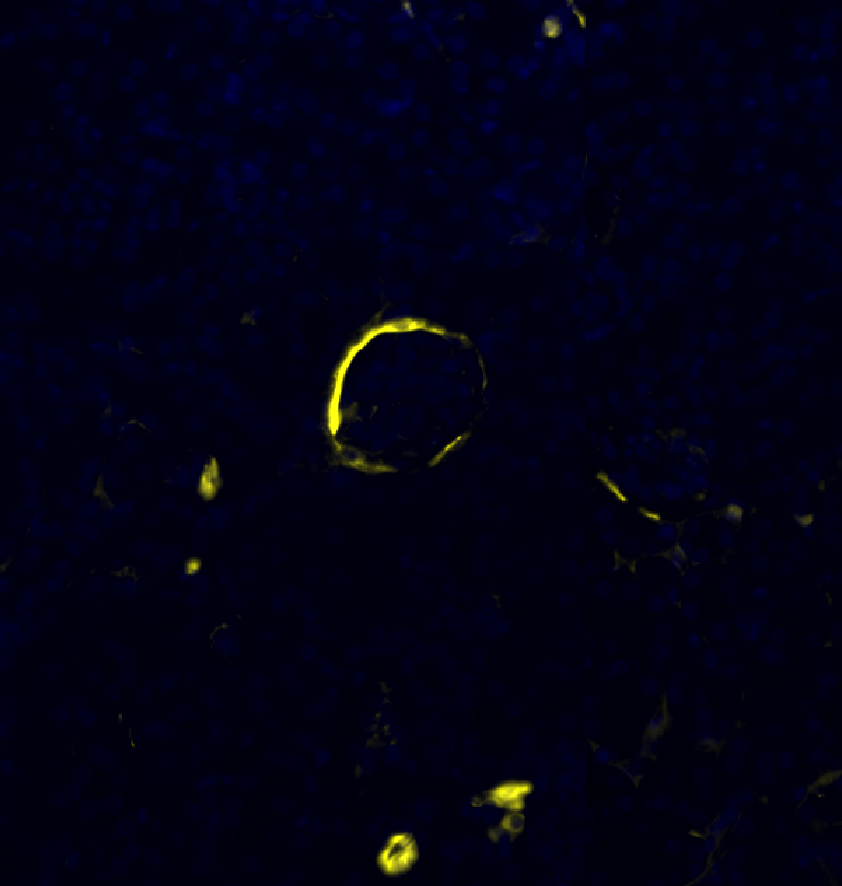

Supplement: Supplementary file 29 — Unprocessed images [file 43587_2024_776_MOESM29_ESM.zip › SD_ED_9_images/Ext_Fig_9_B_old_ DAPI + GD3.tif]

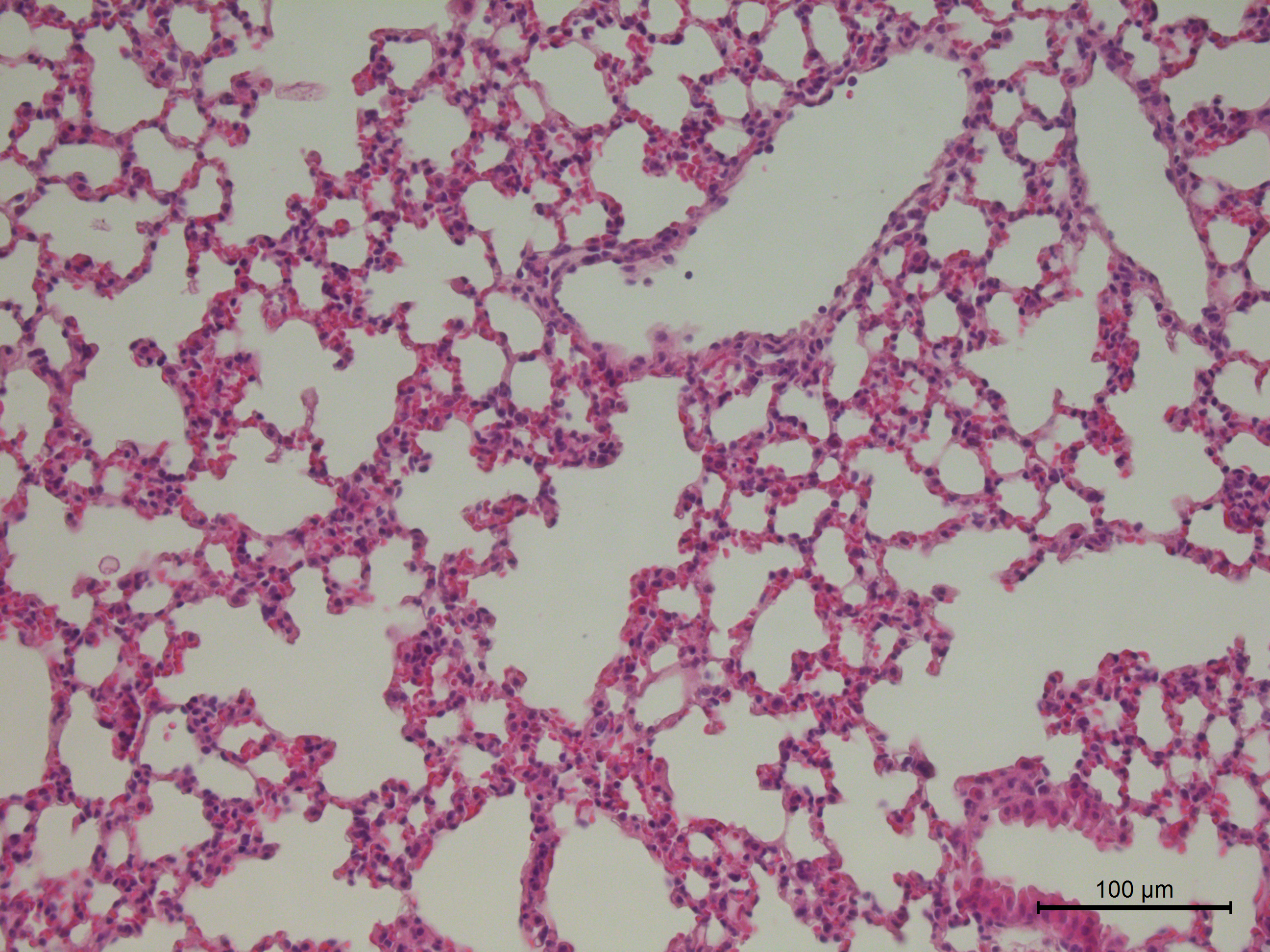

Supplement: Supplementary file 29 — Unprocessed images [file 43587_2024_776_MOESM29_ESM.zip › SD_ED_9_images/Ext_Data_9_HE_24mo.tif]

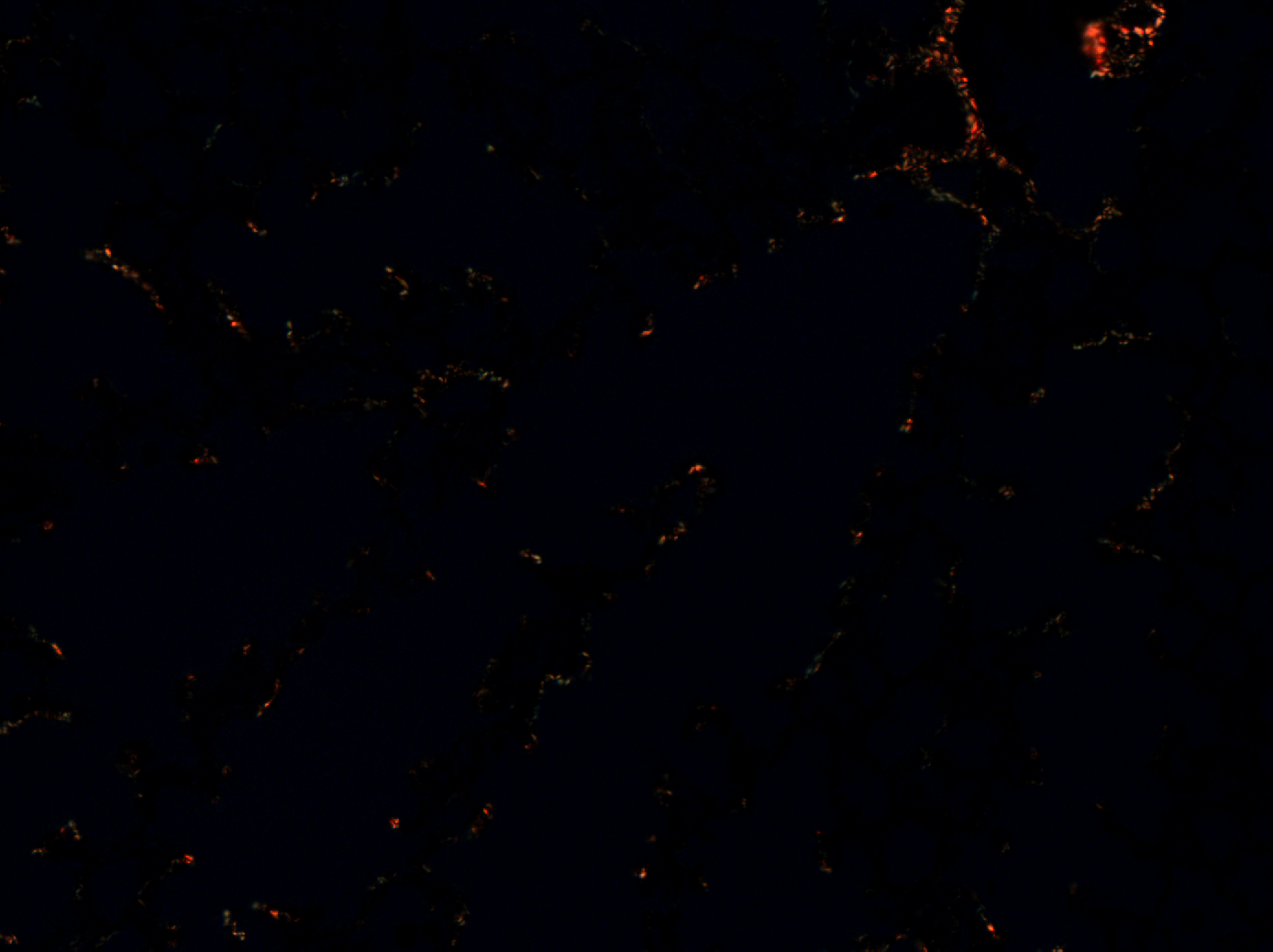

Supplement: Supplementary file 29 — Unprocessed images [file 43587_2024_776_MOESM29_ESM.zip › SD_ED_9_images/Ext_Data_9_SR_PL_24mo.tif]

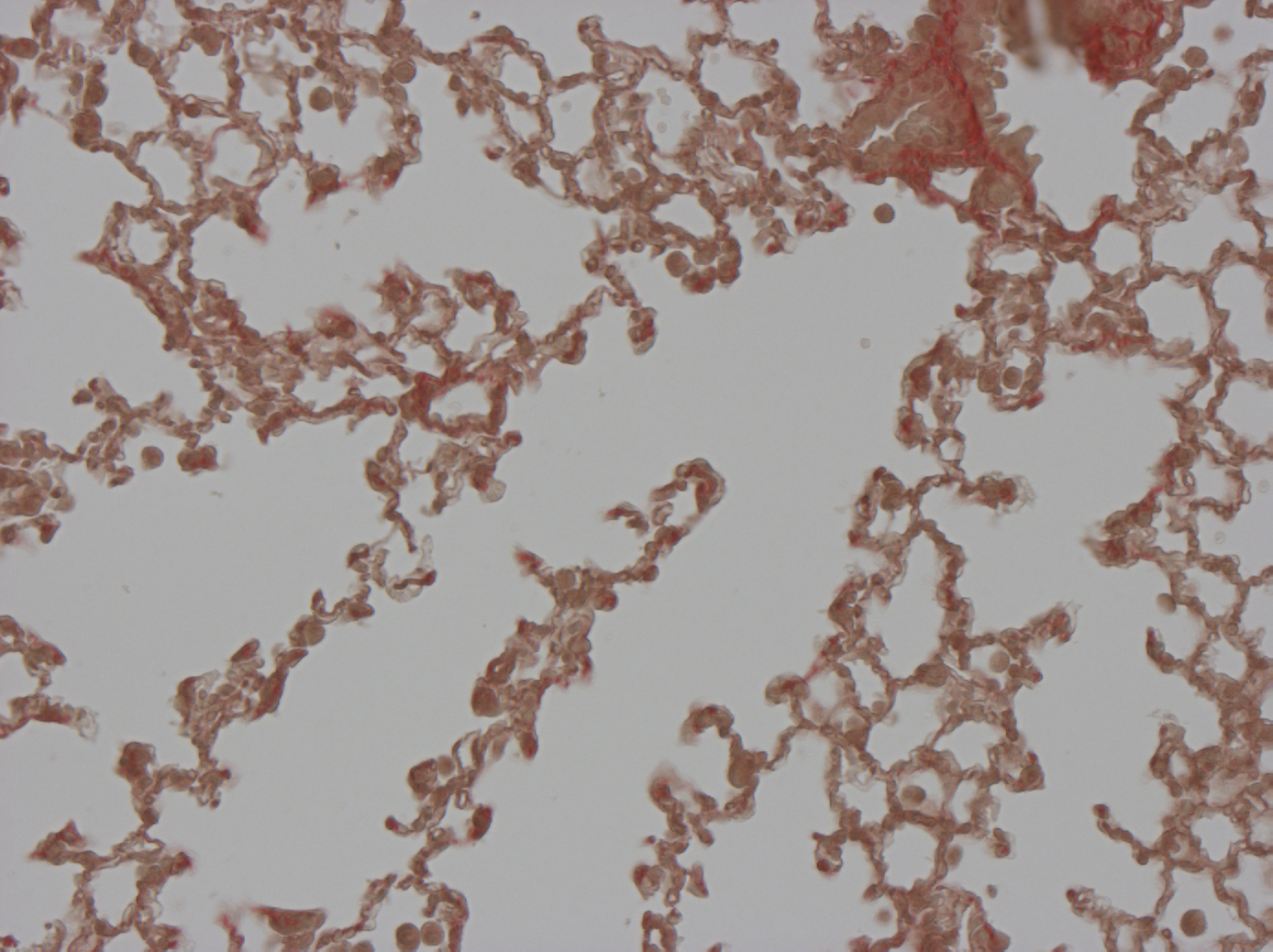

Supplement: Supplementary file 29 — Unprocessed images [file 43587_2024_776_MOESM29_ESM.zip › SD_ED_9_images/Ext_Data_9_SR_BF_24mo.tif]

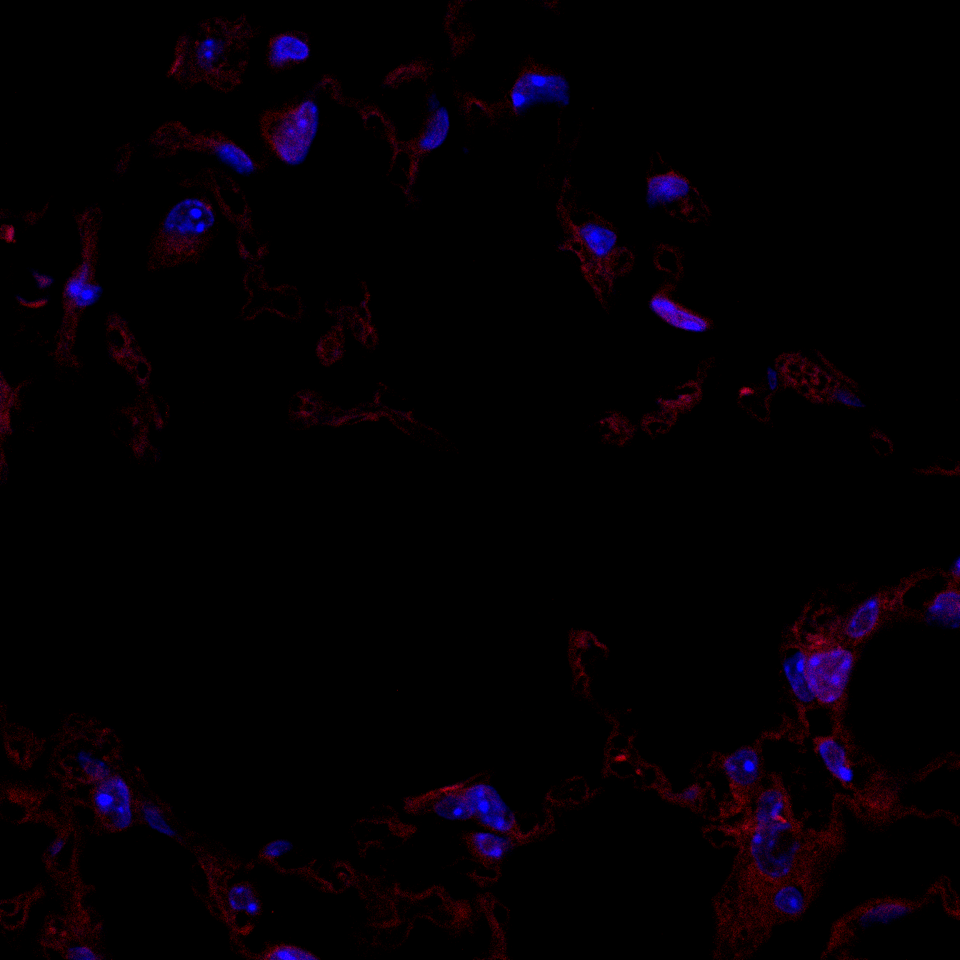

Supplement: Supplementary file 29 — Unprocessed images [file 43587_2024_776_MOESM29_ESM.zip › SD_ED_9_images/Ext_Data_Fig_9_D_KO_G4_GD3.tif]

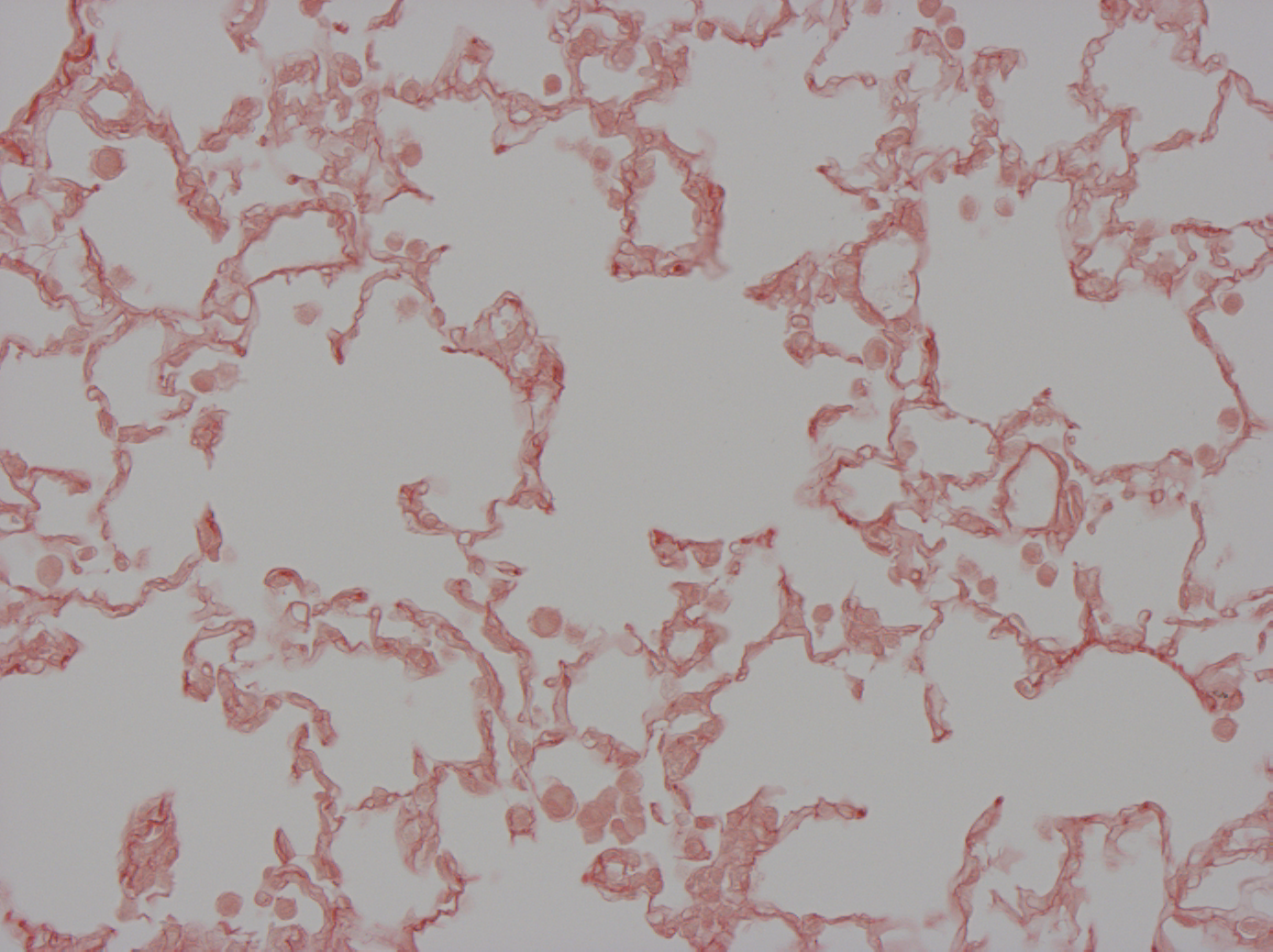

Supplement: Supplementary file 29 — Unprocessed images [file 43587_2024_776_MOESM29_ESM.zip › SD_ED_9_images/Ext_Data_9_D_G0_SR_BF.tif]

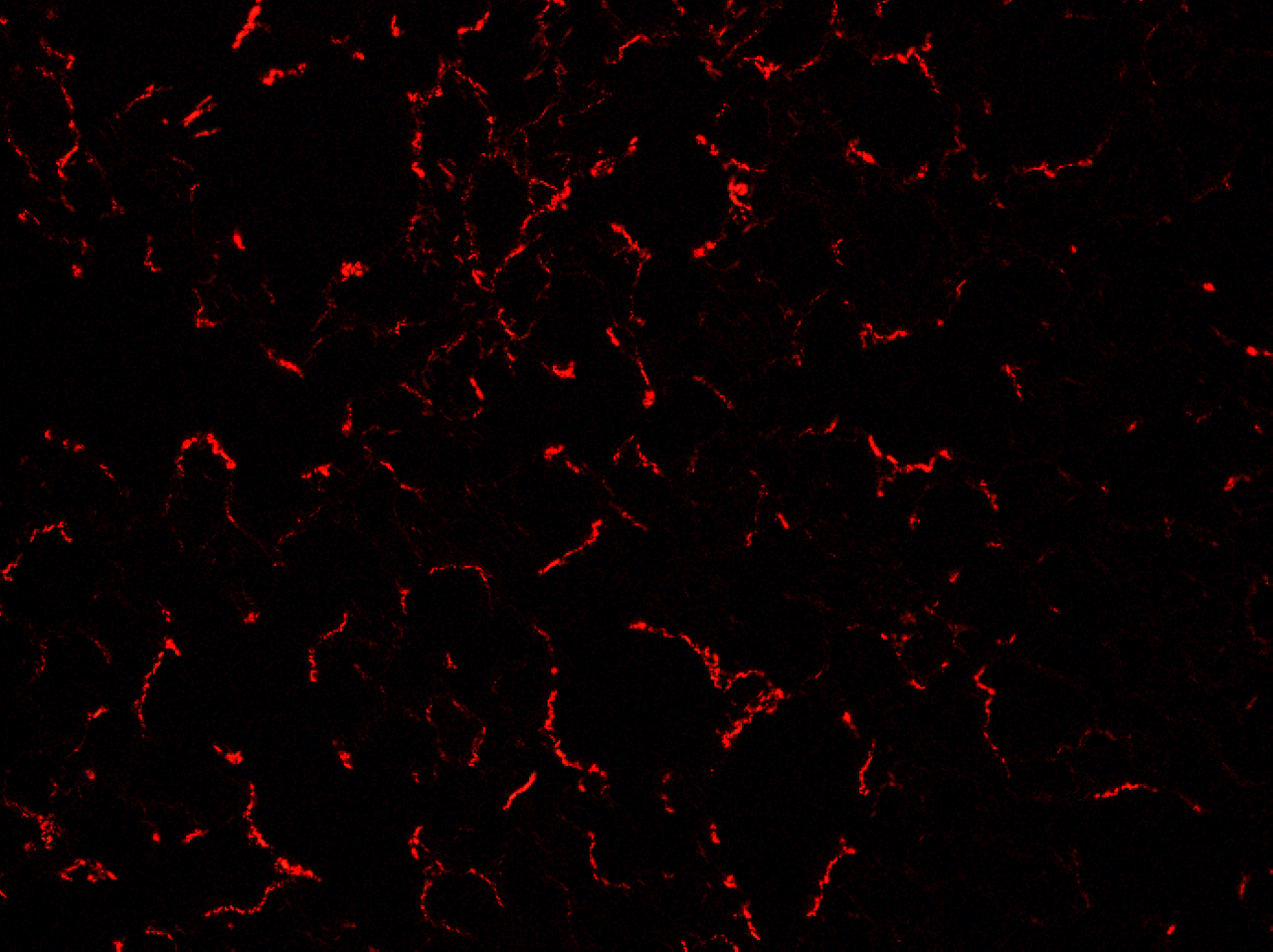

Supplement: Supplementary file 29 — Unprocessed images [file 43587_2024_776_MOESM29_ESM.zip › SD_ED_9_images/Ext_Data_9_D_G4_SR_PL.tif]
