# Supplementary figures and images for: A ganglioside-based immune checkpoint enables senescent cells to evade immunosurveillance during aging (part 2 of 2)
Source: Nat Aging. 2024 Dec 27;5(2):219–36. doi: 10.1038/s43587-024-00776-z (PMC11839482; doi:10.1038/s43587-024-00776-z)

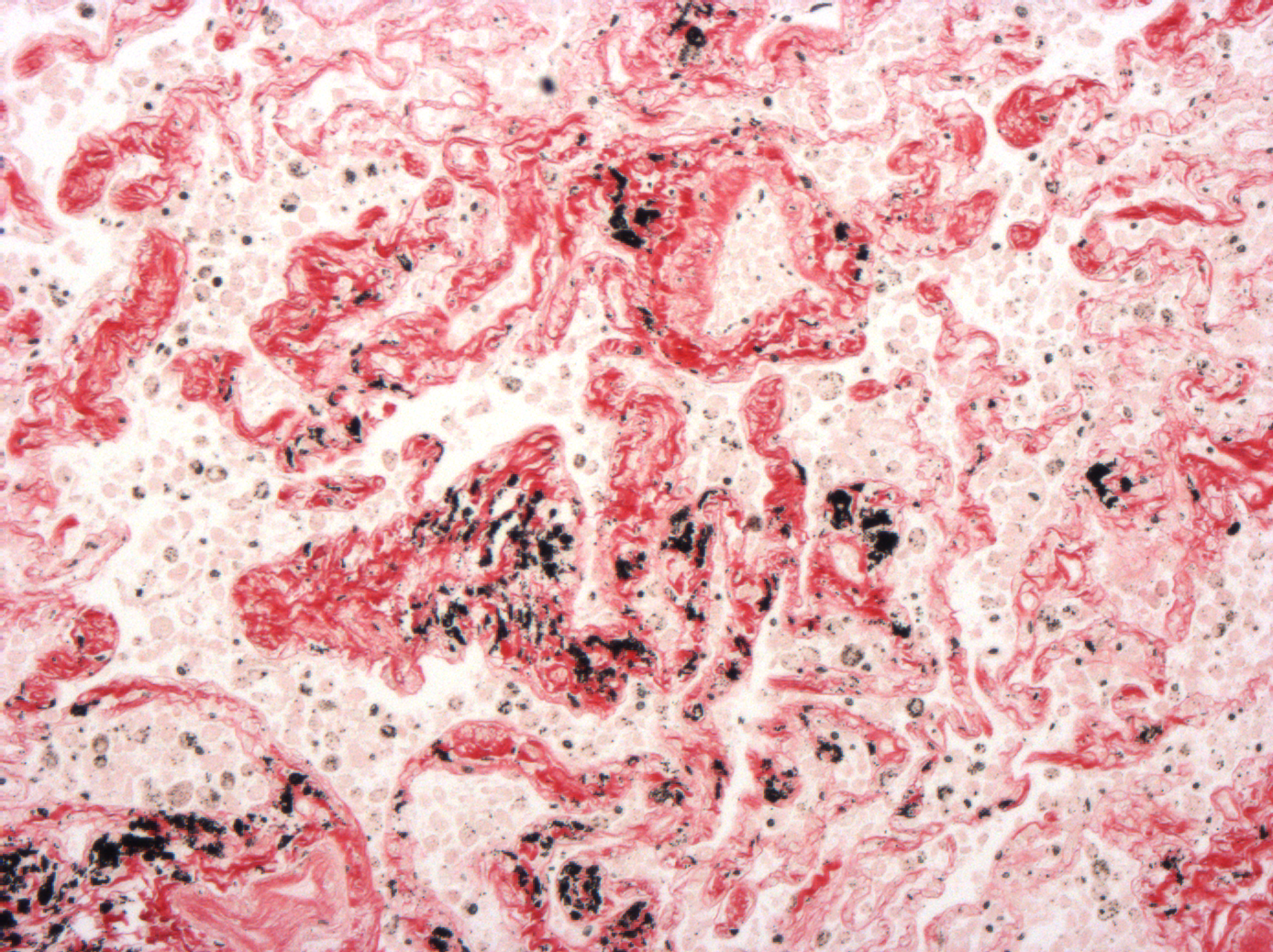

Supplement: Supplementary file 31 — Unprocessed images [file 43587_2024_776_MOESM31_ESM.zip › SD_ED_10_images/Ext_Fig_10_G_High BF SR.tif]

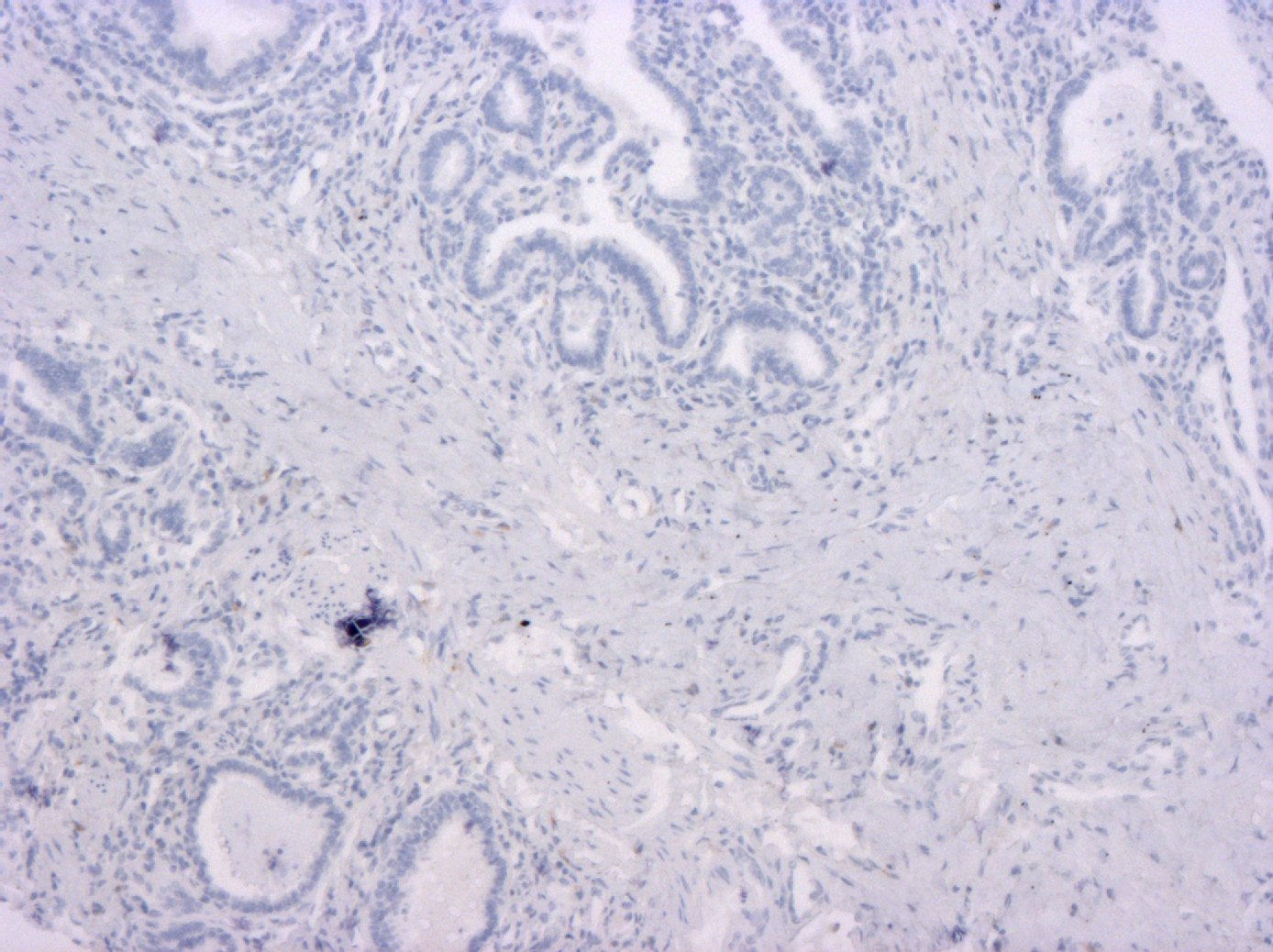

Supplement: Supplementary file 31 — Unprocessed images [file 43587_2024_776_MOESM31_ESM.zip › SD_ED_10_images/Ext_Fig_10_G_Low GD3 IHC.tiff]

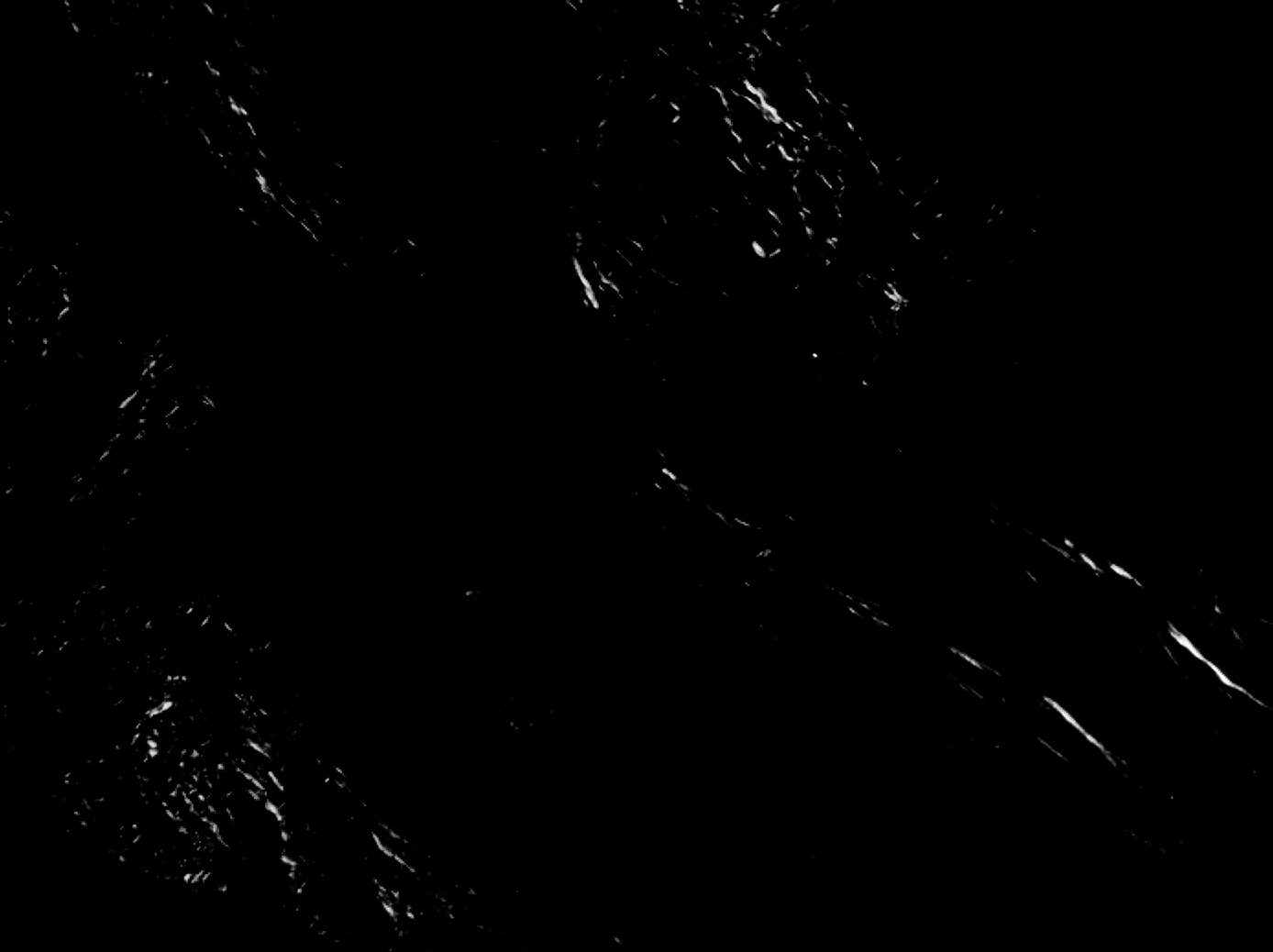

Supplement: Supplementary file 31 — Unprocessed images [file 43587_2024_776_MOESM31_ESM.zip › SD_ED_10_images/Ext_Fig_10_G_Low PL SR.tif]

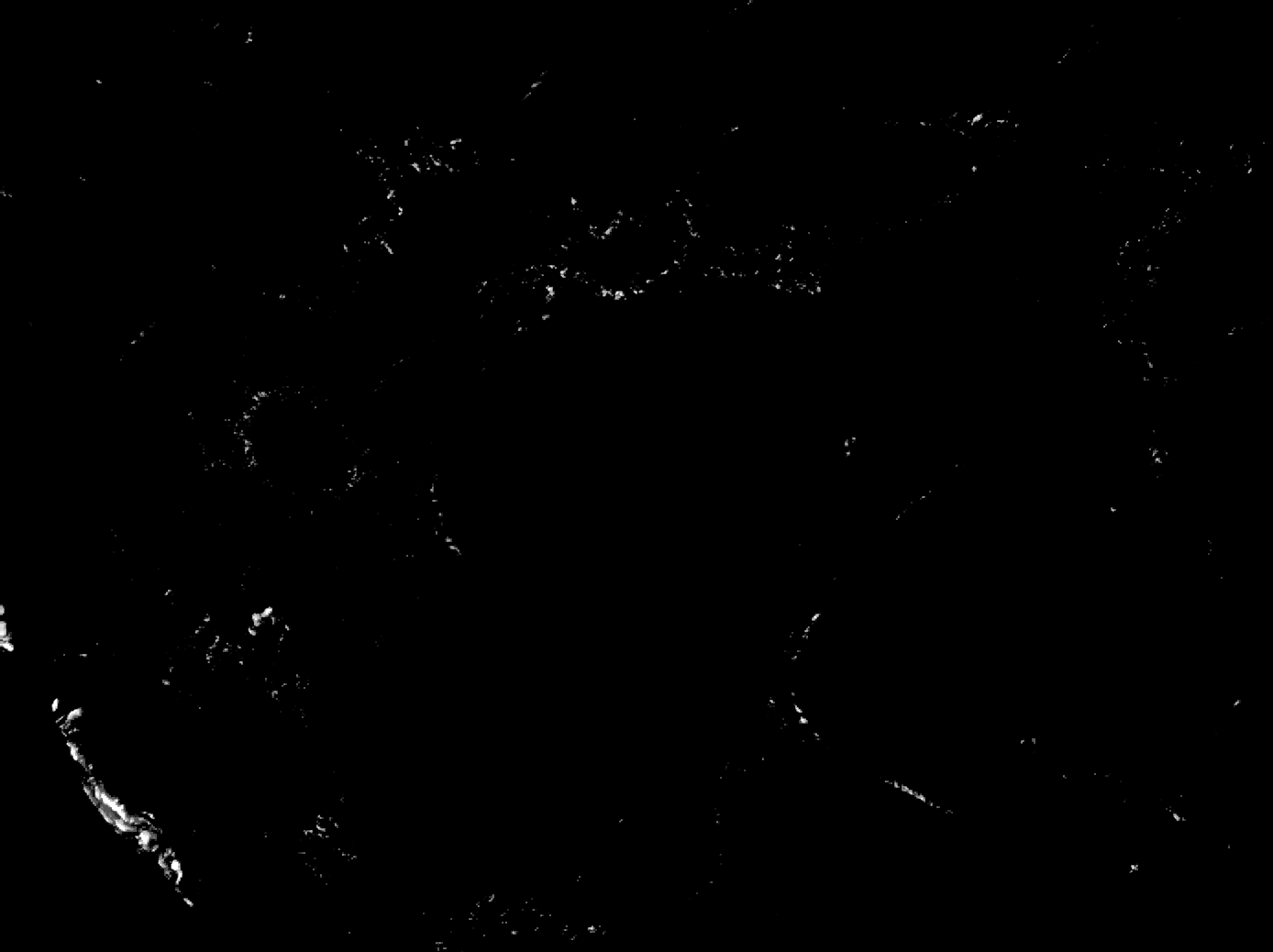

Supplement: Supplementary file 31 — Unprocessed images [file 43587_2024_776_MOESM31_ESM.zip › SD_ED_10_images/Ext_Fig_10_G_Normal PL SR.tif]

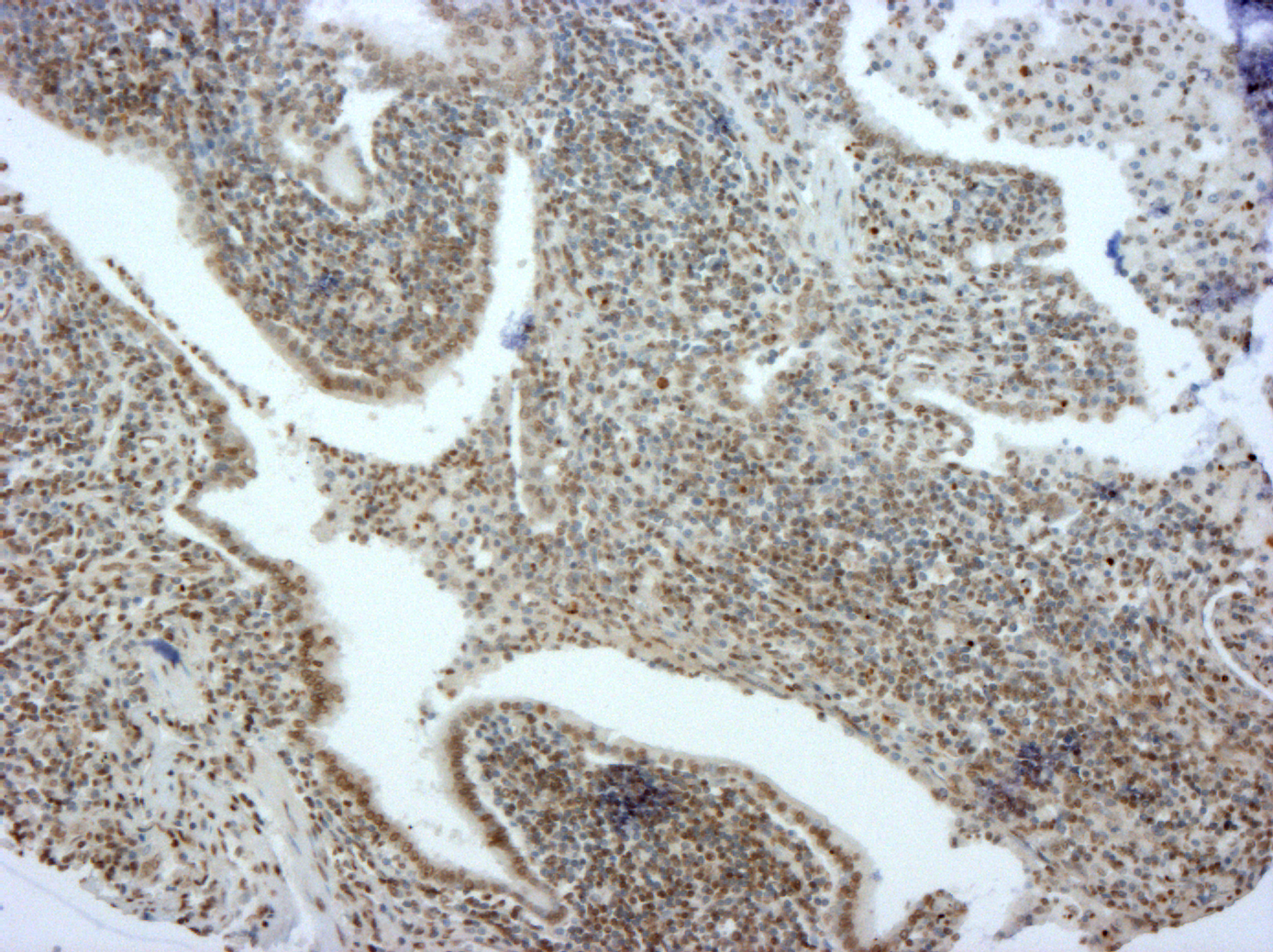

Supplement: Supplementary file 31 — Unprocessed images [file 43587_2024_776_MOESM31_ESM.zip › SD_ED_10_images/Ext_Fig_10_G_High p16 IHC.tiff]

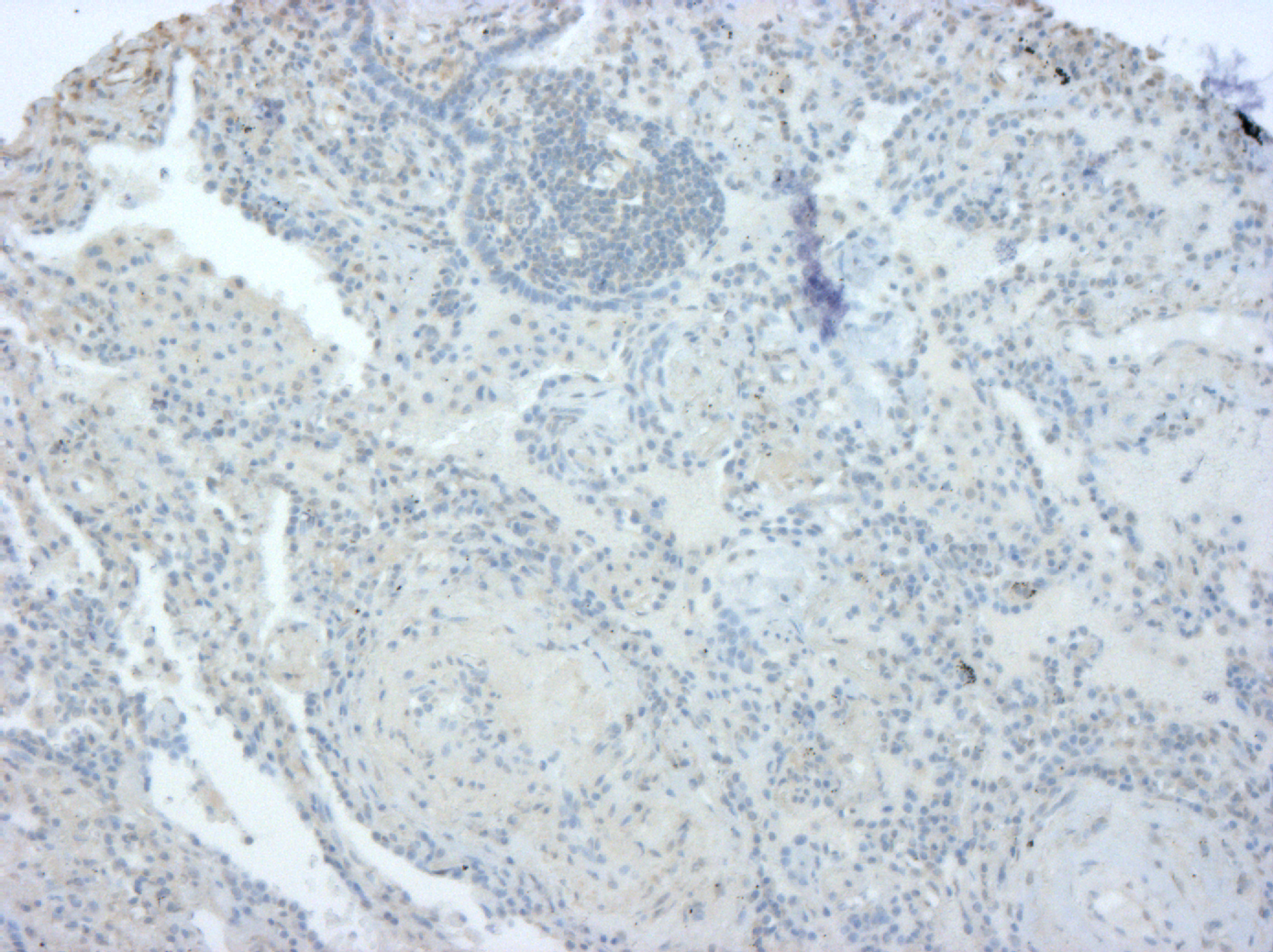

Supplement: Supplementary file 31 — Unprocessed images [file 43587_2024_776_MOESM31_ESM.zip › SD_ED_10_images/Ext_Fig_10_G_Low p21 IHC.tiff]

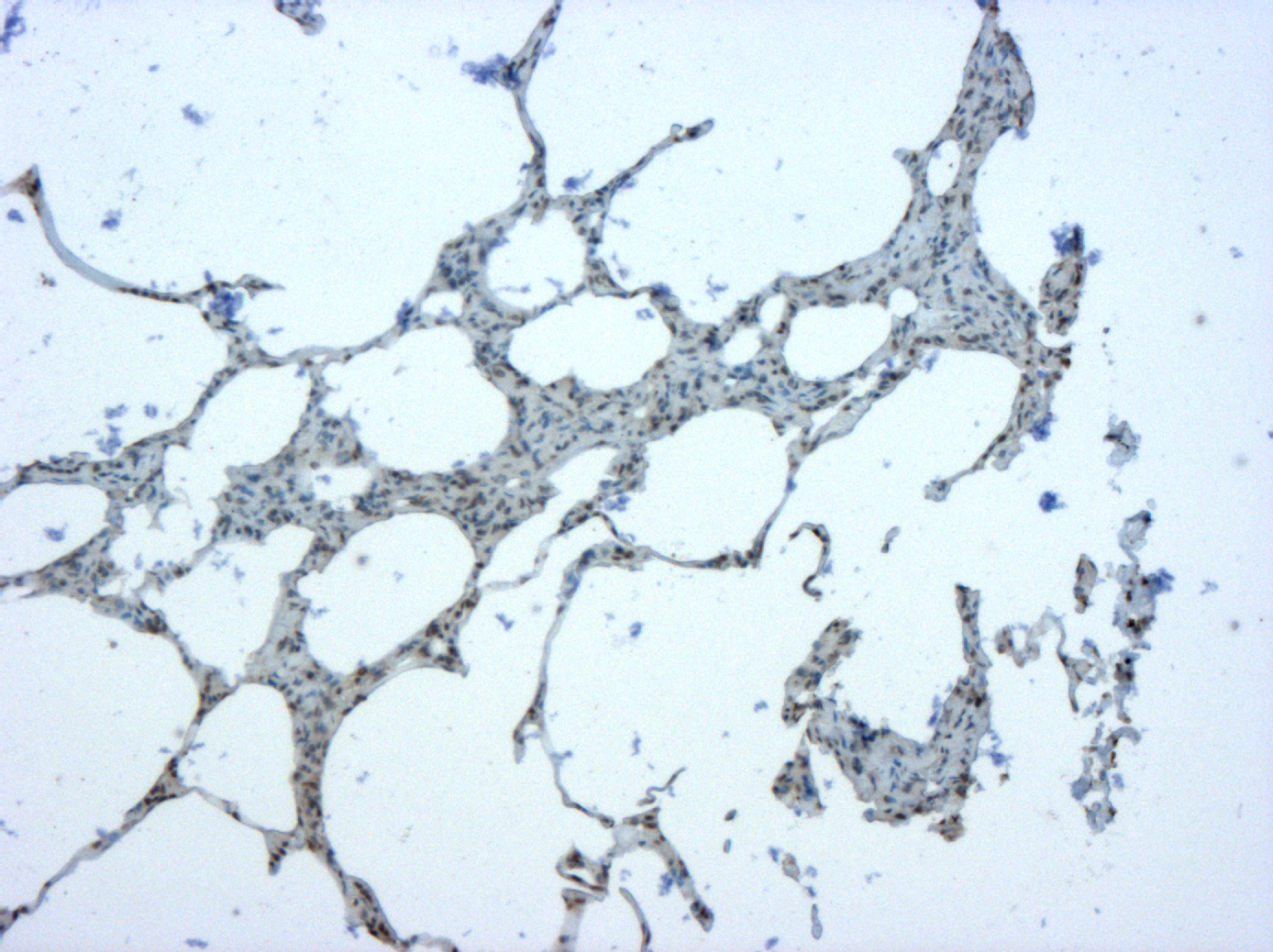

Supplement: Supplementary file 31 — Unprocessed images [file 43587_2024_776_MOESM31_ESM.zip › SD_ED_10_images/Ext_Fig_10_G_Normal p16 IHC.tiff]

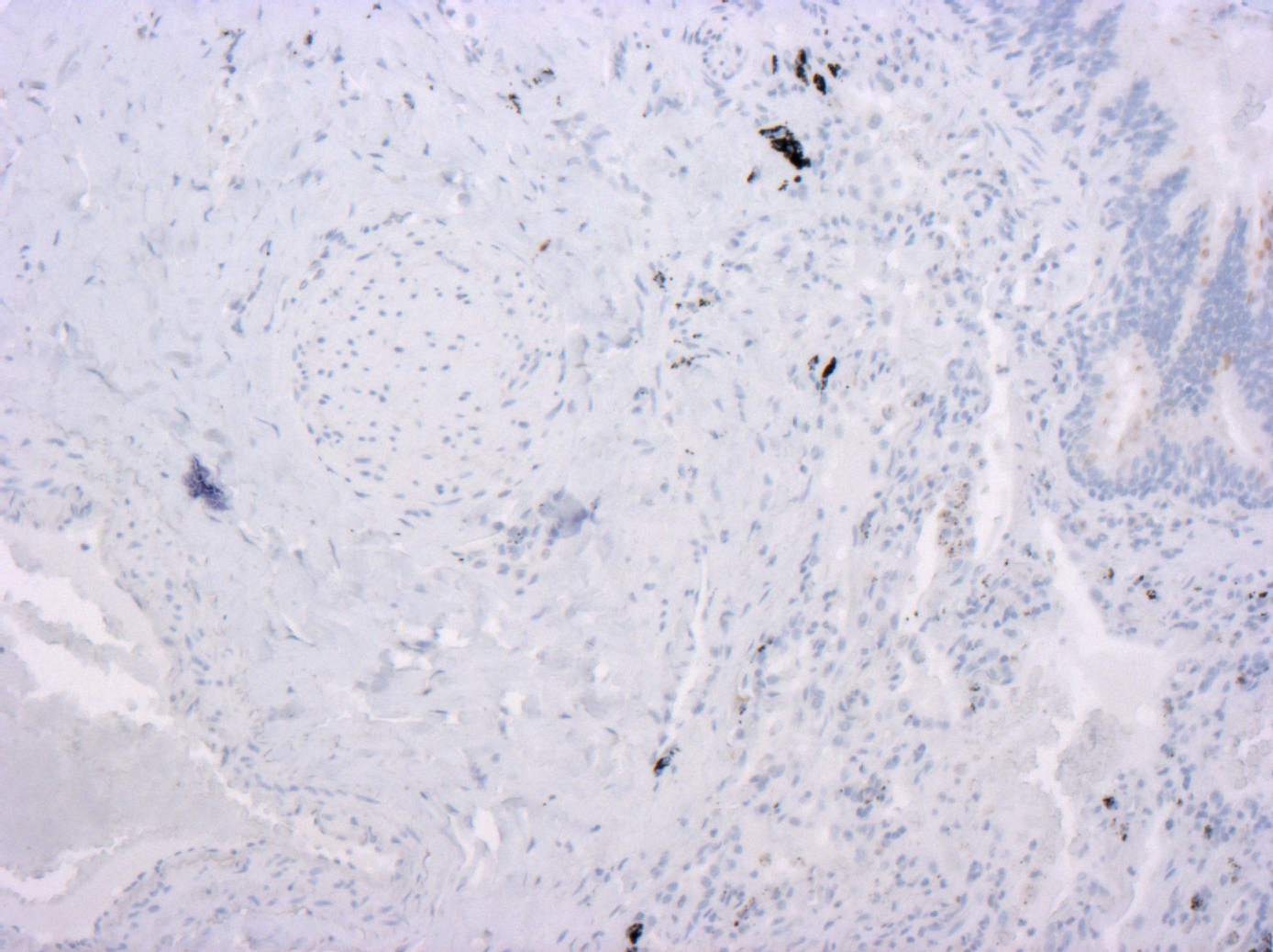

Supplement: Supplementary file 31 — Unprocessed images [file 43587_2024_776_MOESM31_ESM.zip › SD_ED_10_images/Ext_Fig_10_G_High GD3 IHC.tiff]

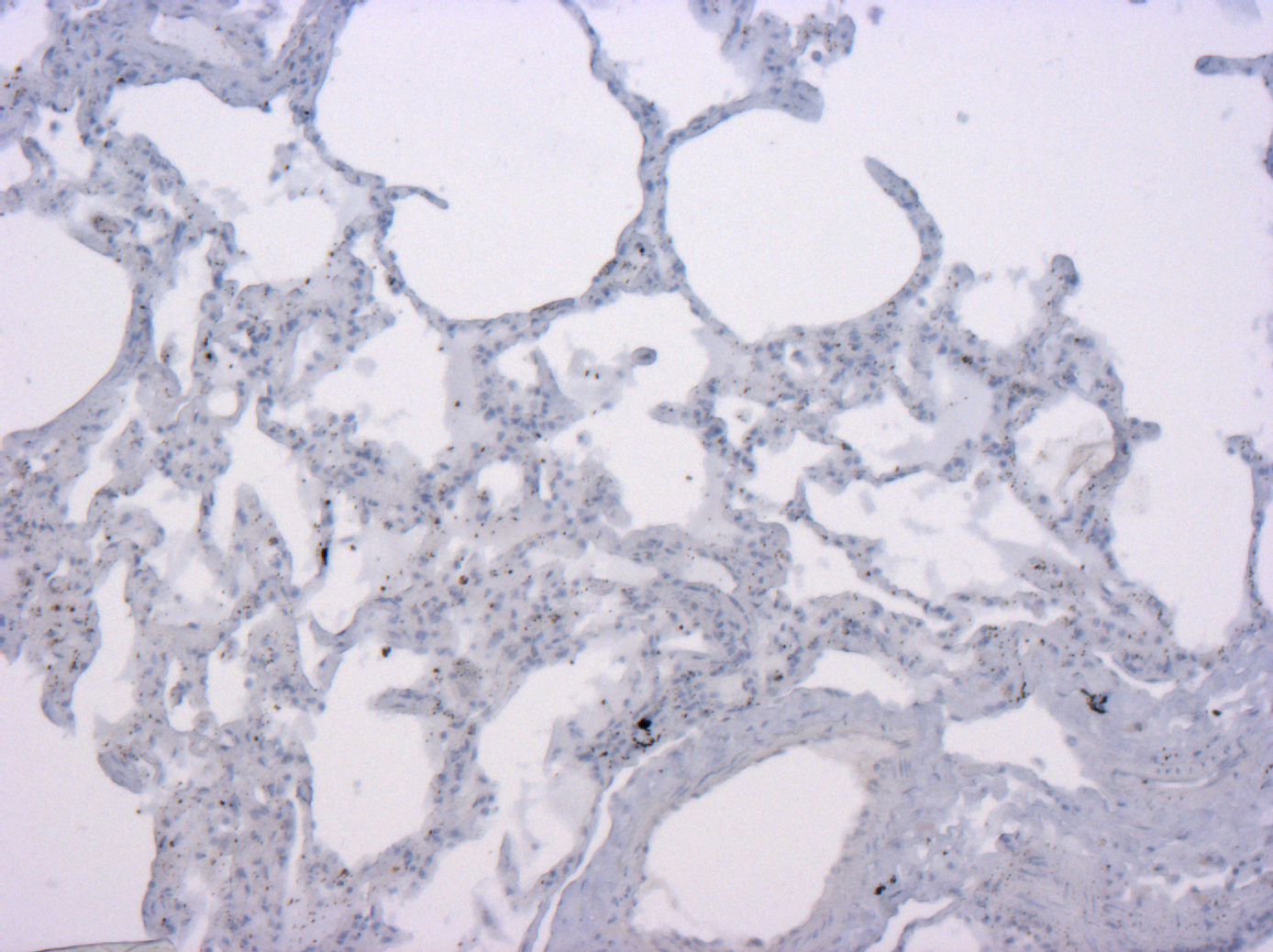

Supplement: Supplementary file 31 — Unprocessed images [file 43587_2024_776_MOESM31_ESM.zip › SD_ED_10_images/Ext_Fig_10_G_Normal GD3 IHC.tiff]

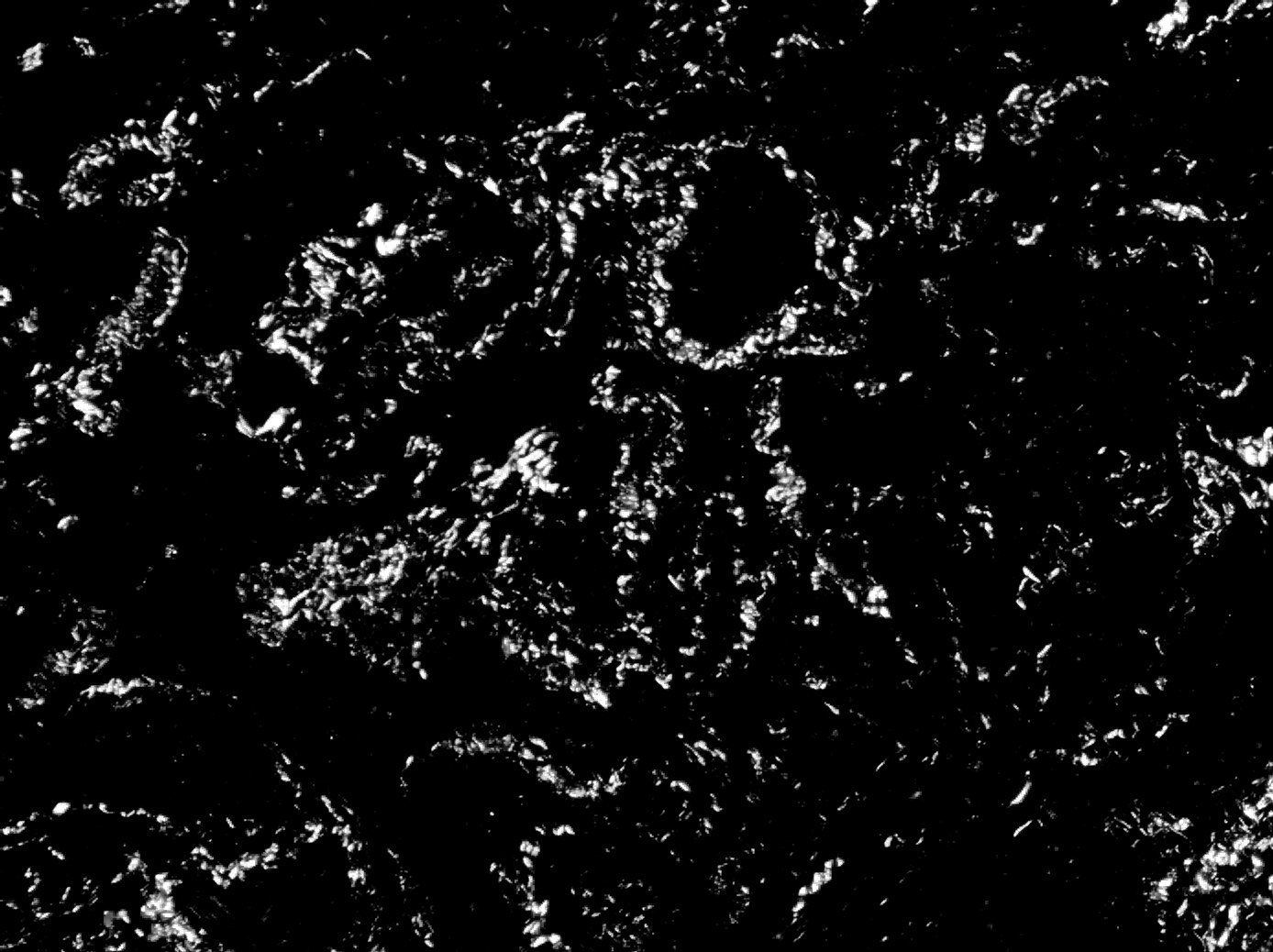

Supplement: Supplementary file 31 — Unprocessed images [file 43587_2024_776_MOESM31_ESM.zip › SD_ED_10_images/Ext_Fig_10_G_High PL SR.tif]

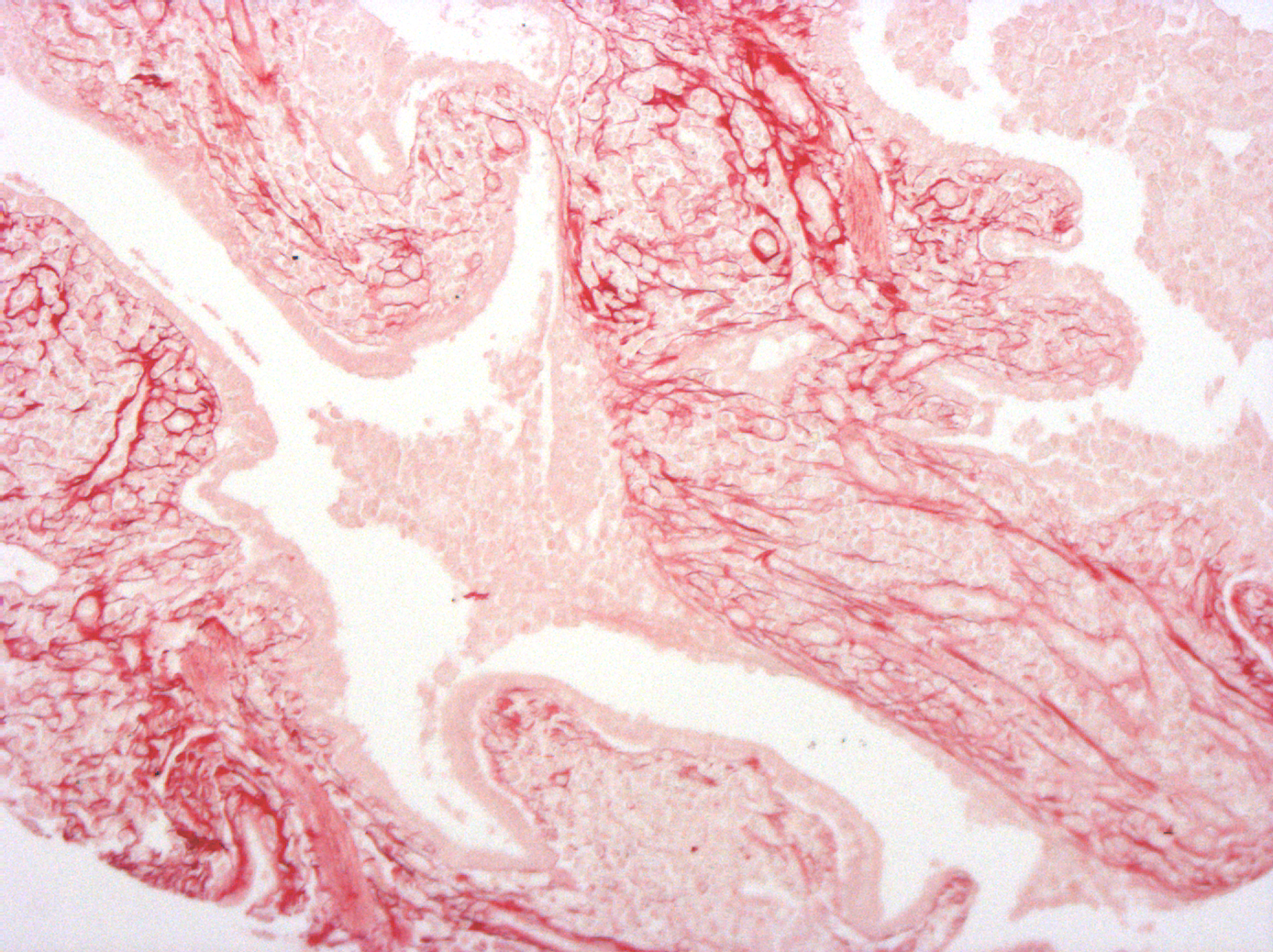

Supplement: Supplementary file 31 — Unprocessed images [file 43587_2024_776_MOESM31_ESM.zip › SD_ED_10_images/Ext_Fig_10_G_Low BF SR.tif]

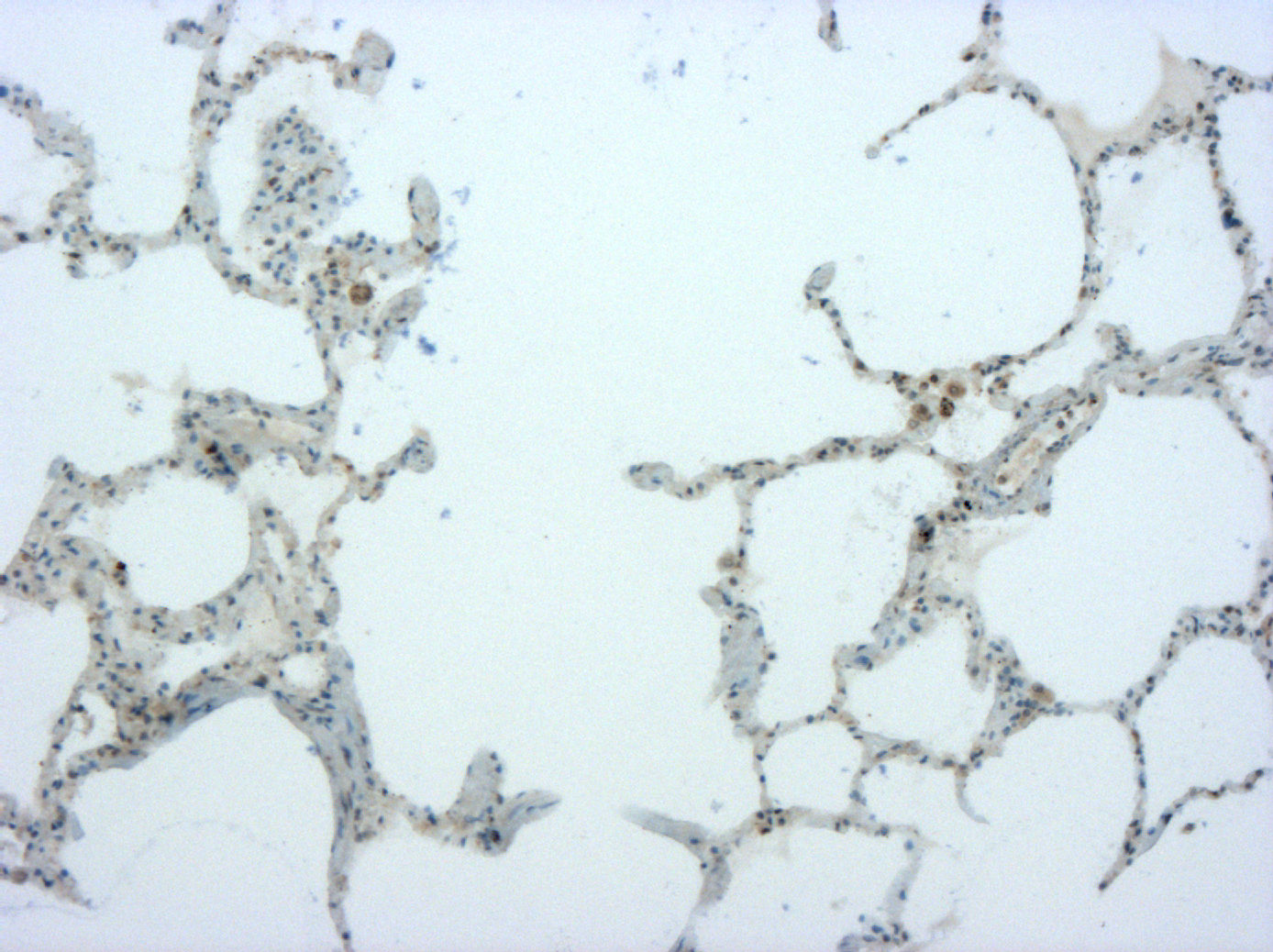

Supplement: Supplementary file 31 — Unprocessed images [file 43587_2024_776_MOESM31_ESM.zip › SD_ED_10_images/Ext_Fig_10_G_Normal p21 IHC.tiff]

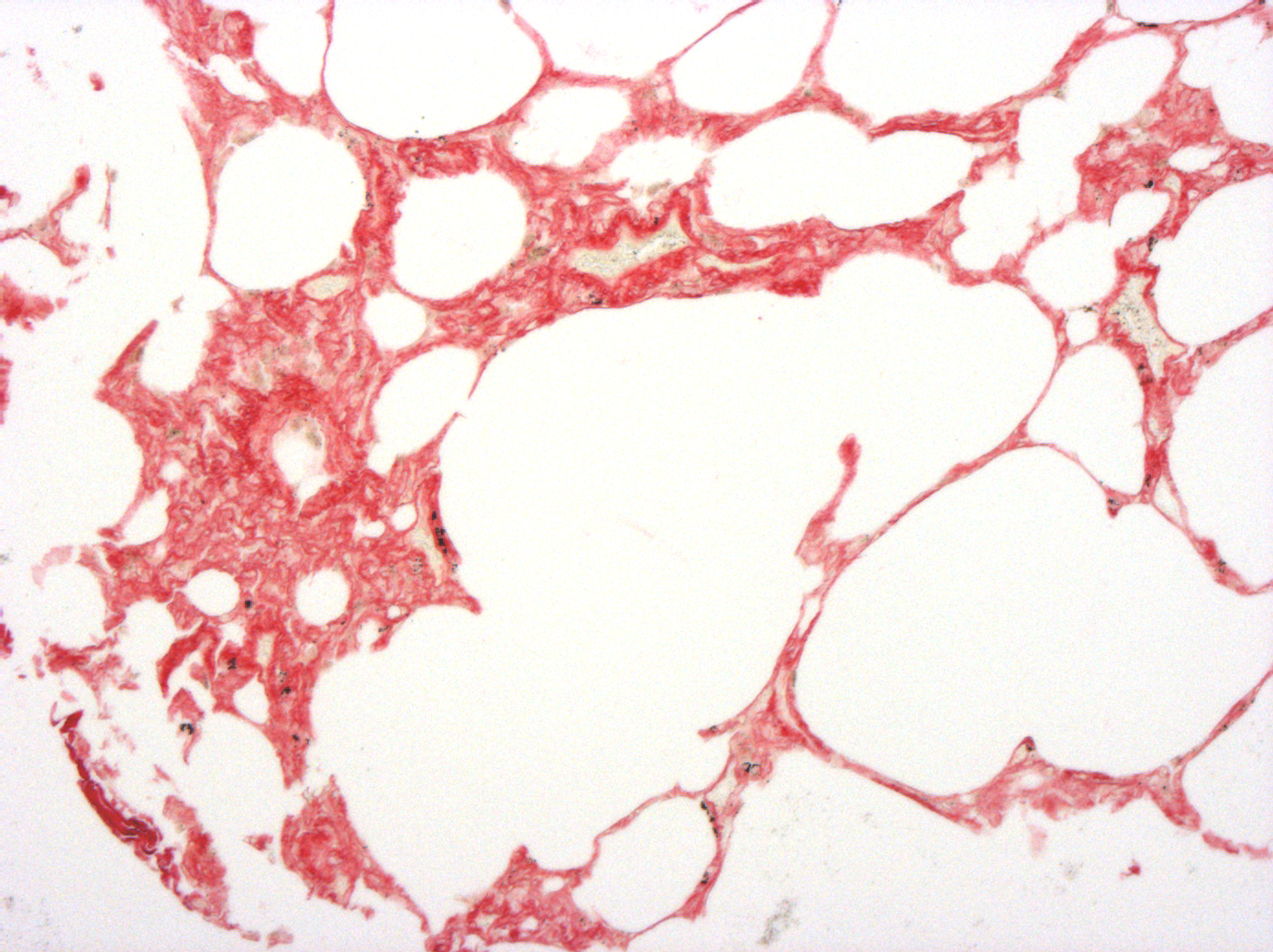

Supplement: Supplementary file 31 — Unprocessed images [file 43587_2024_776_MOESM31_ESM.zip › SD_ED_10_images/Ext_Fig_10_G_Normal BF SR.tif]

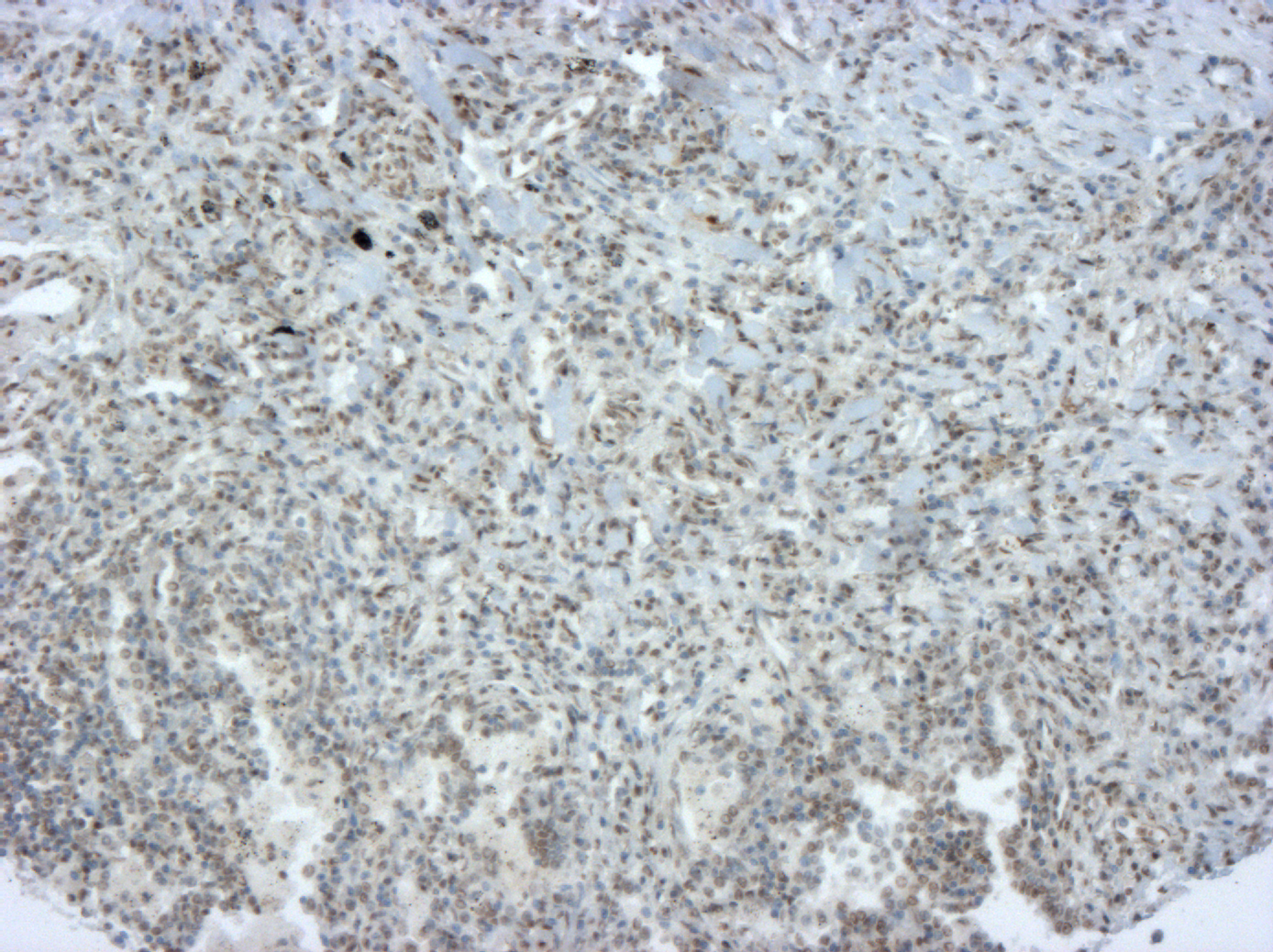

Supplement: Supplementary file 31 — Unprocessed images [file 43587_2024_776_MOESM31_ESM.zip › SD_ED_10_images/Ext_Fig_10_G_Low p16 IHC.tiff]

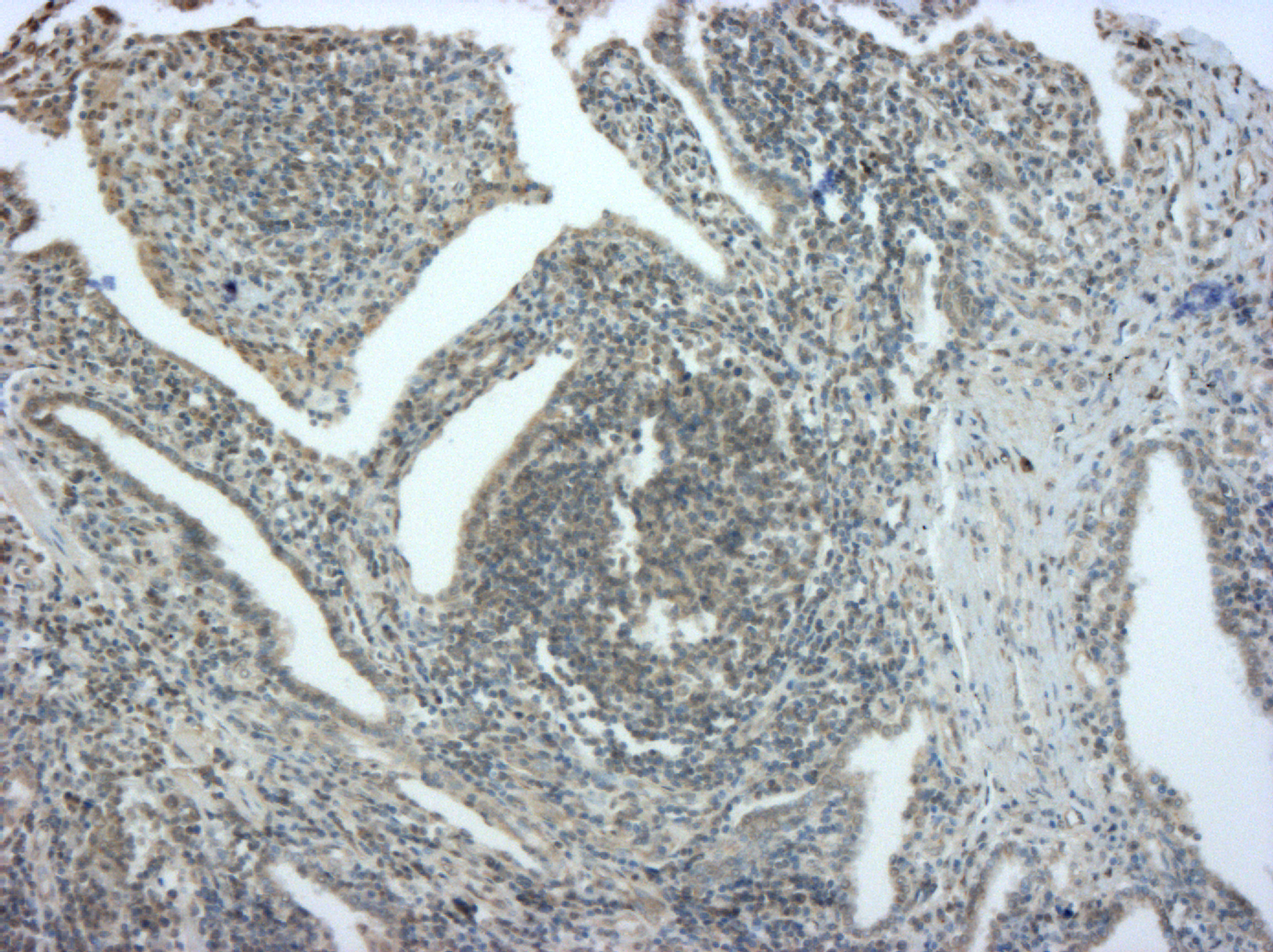

Supplement: Supplementary file 31 — Unprocessed images [file 43587_2024_776_MOESM31_ESM.zip › SD_ED_10_images/Ext_Fig_10_G_High p21 IHC.tiff]
